# Identification of a set of genes showing regionally enriched expression in the mouse brain

**DOI:** 10.1186/1471-2202-9-66

**Published:** 2008-07-14

**Authors:** Cletus A D'Souza, Vikramjit Chopra, Richard Varhol, Yuan-Yun Xie, Slavita Bohacec, Yongjun Zhao, Lisa LC Lee, Mikhail Bilenky, Elodie Portales-Casamar, An He, Wyeth W Wasserman, Daniel Goldowitz, Marco A Marra, Robert A Holt, Elizabeth M Simpson, Steven JM Jones

**Affiliations:** 1 Genome Sciences Centre, British Columbia Cancer Agency, 570 West 7th Ave – Suite 100, Vancouver, BC, V5Z 4E6, Canada; 2 Centre for Molecular Medicine and Therapeutics, Child and Family Research Institute, Department of Medical Genetics, University of British Columbia, 950 West 28th Ave., Vancouver, BC, V5Z 4H4, Canada

## Abstract

**Background:**

The Pleiades Promoter Project aims to improve gene therapy by designing human mini-promoters (< 4 kb) that drive gene expression in specific brain regions or cell-types of therapeutic interest. Our goal was to first identify genes displaying regionally enriched expression in the mouse brain so that promoters designed from orthologous human genes can then be tested to drive reporter expression in a similar pattern in the mouse brain.

**Results:**

We have utilized LongSAGE to identify regionally enriched transcripts in the adult mouse brain. As supplemental strategies, we also performed a meta-analysis of published literature and inspected the Allen Brain Atlas *in situ *hybridization data. From a set of approximately 30,000 mouse genes, 237 were identified as showing specific or enriched expression in 30 target regions of the mouse brain. GO term over-representation among these genes revealed co-involvement in various aspects of central nervous system development and physiology.

**Conclusion:**

Using a multi-faceted expression validation approach, we have identified mouse genes whose human orthologs are good candidates for design of mini-promoters. These mouse genes represent molecular markers in several discrete brain regions/cell-types, which could potentially provide a mechanistic explanation of unique functions performed by each region. This set of markers may also serve as a resource for further studies of gene regulatory elements influencing brain expression.

## Background

The Pleiades Promoter Project (please see Availability & requirements for more information) addresses two major challenges identified in gene therapy – first, the delivery of DNA to specific cell types to reduce side effects from treating healthy cells and second, controlled delivery of DNA to a specific locus in the genome to avoid insertional mutagenesis. The goal for the project is the generation of human DNA promoters less than 4 kb in length (mini-promoters) that drive gene expression in brain regions important in neurological conditions. To achieve this goal, we have first identified genes with enriched expression in different regions of the adult mouse brain. Regional expression patterns within the brain tend to be conserved between orthologous human and mouse genes [1]. Additionally, as regulatory sequences in tissue-specific genes tend to be highly conserved [2], human mini-promoters are expected to drive regional gene expression in transgenic mice based on earlier studies [3]. Therefore, promoter regions from orthologous human genes will be assessed in the mouse brain for the ability to drive regional expression.

Selection of the most optimal genes for promoter design necessitates detailed assessment of gene expression patterns. An invaluable resource to identify genes expressed in the mammalian brain is the serial analysis of gene expression (SAGE) technique [4,5]. A modern improvement of tag-based expression analysis is LongSAGE, which produces longer transcript tags (21-bp) better suited to unique mapping onto cDNA and genome sequences [6]. As part of the Mouse Atlas of Gene Expression project [7], LongSAGE was used to profile transcriptomes of 72 tissues of mouse strain C57BL/6J at various stages of development [8]. For the Pleiades Promoter Project [9], a scion of the Mouse Atlas project, we have generated new LongSAGE data on gene expression in the adult mouse central nervous system to identify genes that display enriched expression in key brain regions.

While LongSAGE provides a rich perspective on gene expression patterns, we extended our data mining efforts to include other large information sources. The PubMed database [10] provides an unparalleled compendium of text from the scientific literature. In order to facilitate extraction of key information from Medline abstracts or full-text articles in PubMed, natural language processing tools are routinely employed to semi-automate the process of literature mining [11,12]. In this study we investigated an approach to specifically and automatically identify associations between genes and brain regions from the literature. We further analysed expression data from the Allen Brain Atlas (ABA; [13]), a high-throughput *in situ *hybridization platform that has assayed expression for ~20,000 genes in the adult mouse brain [14,15]. Here, we report the successful utilization of a combination of gene-finding tools, including SAGE analysis, text mining and ABA expression data, to identify genes displaying regionally enriched expression in surrogate regions of therapeutic interest within the mouse brain.

## Results

### Identification of brain region-enriched gene expression by LongSAGE

**Figure 1:**
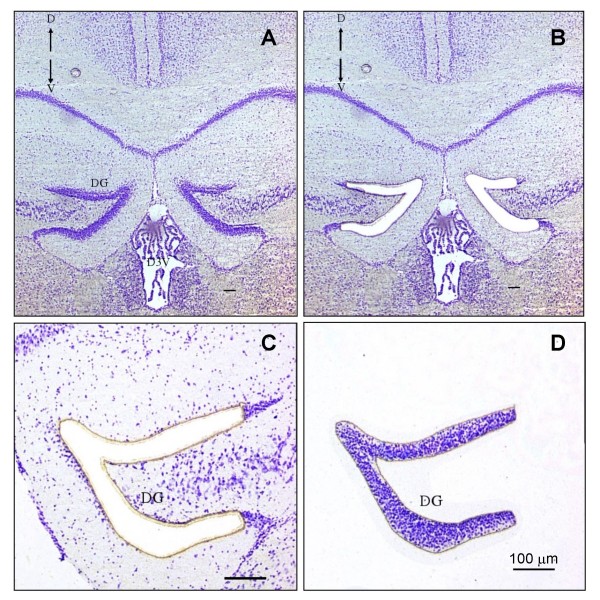
**Use of Laser Capture Microdissection to isolate the hippocampus dentate gyrus from an adult mouse**. A) Intact coronal brain section at ~Bregma -1.35 stained with cresyl violet. B & C) dentate gyrus (DG) has been microdissected with laser. D) dentate gyrus has been isolated and captured for total RNA extraction and construction of SAGE libraries. Images were captured using a Sony DXC-390P 3-CCD color video camera attached to a Nikon Eclipse TE2000-S microscope (10× magnification). Scale bar = 100 μm. D: dorsal; V: ventral.

To identify regionally enriched gene expression within the brain of the adult mouse strain C57BL/6J, we used the precision of Laser Capture Microdissection (LCM; Figure 1) [16] to isolate component tissues and construct SAGE libraries from 17 brain regions as well as the whole adult mouse brain for comparison (Methods). As shown in Table 1, these libraries have been sampled to a depth of > 100,000 tags each, a level shown to be adequate for the discovery of medium-to-high level transcripts [8]. Bioinformatics analysis of differential gene expression was performed as described in Methods. Since the majority of transcripts were detected in multiple libraries, we employed a heuristic approach to identify and rank expression patterns (outlined in Table 2). For each brain region, we ranked genes from 1–91 based on the level and pattern of expression in descending order. Expression specificity of a ranked list of 1999 SAGE-identified genes was then confirmed by examining related literature information and Allen Brain Atlas *in situ *hybridization data. Based on this collective information, region-specific or region-enriched genes were further considered.

**Table 1:** List of adult brain region SAGE libraries

**Name**	**Description**	**No. of Genes**	**Total Tags^b^**
SM098	Whole brain^a^	6893	108441
SM110	Hypothalamus	6676	108882
SM132	Ventral Thalamus	6441	105701
SM137	Hippocampus Dentate Gyrus, dorsal/anterior	5935	104322
SM139	Medial Thalamus	6608	105364
SM147	Visual Cortex Layers II/III/IV	6683	136039
SM152	Substantia Nigra	6584	115991
SM153	Basal Nucleus of Meynert	6581	120997
SM180	Locus Coeruleus	6282	102933
SM181	Raphe Nuclei	6434	104627
SM182	Cerebellum White Matter	5461	107335
SM183	Primary Motor Cortex	6543	115262
SM184	Hippocampus CA1, dorsal/anterior	6331	118198
SM193	Amygdala, basolateral complex	6396	109772
SM194	Amygdala, central nucleus	6451	110056
SM195	Dorsal striatum	6185	105509
SM196	Cerebellum, Purkinje Cell Layer	6604	104850
SM201	Ependymal and Subependymal Layers	6561	107041

^a^Manually dissected; all others were laser capture microdissected

^b^Represents filtered data

**Table 2:** Rank order based on the level and pattern of gene expression

**Rank Order**	**Expression Pattern**
1	1 TL* and 0 OTL* (*P*_*TL*-*OTL *_< = 0.05) (TL tag count > = 5)
2–6	1 TL and 1–5 OTLs (*P*_*TL*-*OTL *_< = 0.05)
7–11	1 TL and 1–5 OTLs (*P*_*TL*-*OTL *_> 0.05) (TL tag count > = 5, OTL tag count: 1–4)
12–17	2 TLs and 0–5 OTLs (*P*_*TL*-*TL *_> 0.05; *P*_*TL*-*OTL *_< = 0.05)
18–22	2 TLs and 1–5 OTLs (*P*_*TL*-*TL *_> 0.05; *P*_*TL*-*OTL *_> 0.05) (TL tag count > = 5, OTL tag count: 1–4)
23–28	3 TLs and 0–5 OTLs (*P*_*TL*-*TL *_> 0.05; *P*_*TL*-*OTL *_< = 0.05)
29–33	3 TLs and 1–5 OTLs (*P*_*TL*-*TL *_> 0.05; *P*_*TL*-*OTL*_> 0.05) (TL tag count > = 5, OTL tag count: 1–4)
34–39	4 TLs and 0–5 OTLs (*P*_*TL*-*TL *_> 0.05; *P*_*TL*-*OTL*_< = 0.05)
40–44	4 TLs and 1–5 OTLs (*P*_*TL*-*TL *_> 0.05; *P*_*TL*-*OTL *_> 0.05) (TL tag count > = 5, OTL tag count: 1–4)
45–55	1 TL and 6–16 OTLs (*P*_*TL*-*OTL *_< = 0.05)
56–65	2 TLs and 6–15 OTLs (*P*_*TL*-*TL *_> 0.05; *P*_*TL*-*OTL *_< = 0.05)
66–74	3 TLs and 6–14 OTLs (*P*_*TL*-*TL *_> 0.05; *P*_*TL*-*OTL *_< = 0.05)
75–82	4 TLs and 6–13 OTLs (*P*_*TL*-*TL *_> 0.05; *P*_*TL*-*OTL *_< = 0.05)
83	1 TL with 4 tags
84	1 TL with 3 tags
85	2 TLs with 4 tags
86	2 TLs with 3 tags
87	1 TL with 2 tags
88	2 TLs with 2 tags
89	3 TLs with 3 tags
90	3 TLs with 2 tags
91	1 TL with 1 tag

* TL = Target library (brain region of interest), OTL = Off-target library (background region)

Of the 237 genes identified as displaying regionally enriched expression in this study, 132 genes [see Additional file 1] displayed expression patterns listed in Table 2. Only 22 genes were found in a single library and five of these (A930006D11Rik, *Chrna6*, *Gdf10*, *Hcrt*, and *Hes3*) were determined to be tissue-specific at a statistically significant level (tag counts > 5, *P *< 0.05).

### Complexity of the adult mouse brain transcriptome and SAGE-based analysis of transcriptome similarity of brain regions

As an indication of complexity of the adult mouse brain transcriptome, within the 18 Pleiades libraries (including whole adult brain library) expression was observed for 11,836 genes of the total 17,098 genes detectable within the Mouse Atlas (total number of tags mapped to the Mouse Atlas libraries was approximately 8.8 million including singletons). In contrast, the Allen Brain Atlas (ABA) contains expression patterns of approximately 16,000 genes across the entire adult C57BL/6J mouse brain (Susan Sunkin, ABA, personal communication); of these genes, roughly 65.5% (10,479/16,000) were detectable in the 18 Pleiades libraries. Furthermore, the Pleiades libraries provided about 8% (1,357/17,357) additional genes to the total number of genes detectable by ABA.

**Figure 2:**
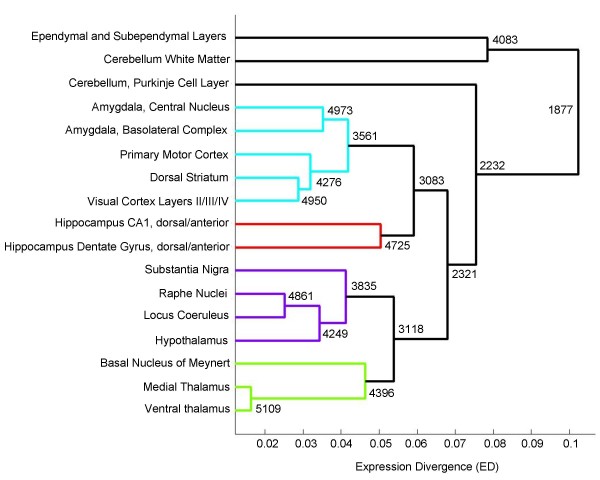
**Transcriptome similarity among 17 brain tissues based on expression divergence at *P *value = 0.01**. Tissues being compared are indicated on the Y-axis, and expression divergence (ED_*P*_) of clusters of tissues is plotted on the X-axis. At each node in the dendrogram, the number of genes shared between libraries in the tissue cluster is indicated. A threshold of 50% of maximum ED_*P *_was chosen for coloring of branch lines in the dendrogram.

We also analyzed SAGE data to measure transcriptome similarity between selected tissues. The premise was that tissues would cluster together or diverge based on the degree to which their genes are differentially expressed. Hierarchical clustering was done based on unweighted average distance between formed clusters (see description in Methods), the results of which are displayed in the form of a dendrogram (Figure 2). A pattern of divergent tissue clusters consistently emerges: a cluster of neuronal tissues and several discrete single tissue clusters including Ependymal Layers, Cerebellum White Matter and Cerebellum Purkinje Cell Layer. Among neuronal tissues, the Ventral and Medial Thalamus consistently clustered tightly together and had the lowest expression divergence between any two pairs of tissues. Additionally, Visual Cortex, Primary Motor Cortex, Amygdala (basolateral), Amygdala (central), and Dorsal Striatum also clustered together. Segregation of the Ependymal tissue into a separate single cluster makes sense given its non-neuronal nature [17], and the Cerebellar White Matter is composed of myelinated axonal processes. Clustering is usually sensitive to the specific expression divergence measure used. However, we tried several empirical measures, as well as different *P *values for selecting differentially expressed genes, and observed that the main pattern of clustering outlined above remains unchanged.

### Literature mining strategy to rapidly identify genes associated with brain regions of interest

**Figure 3:**
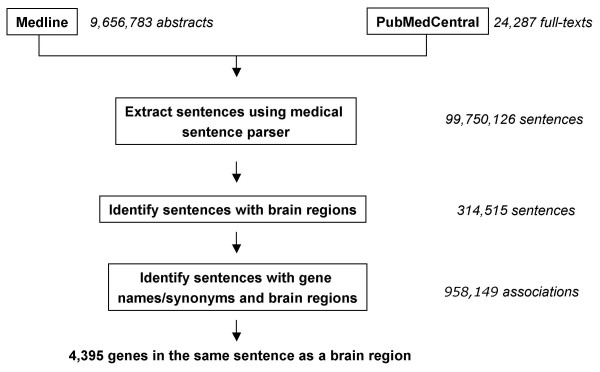
**Text mining data flow**. This shows the steps by which the medical sentence parser retrieves Medline records that contain expression information for a gene in a specific region of the brain.

We included in the present analysis several additional brain regions and cell-types, for example, Blood-Brain Barrier, Barrington's Nucleus, Astroglia etc., for which SAGE libraries had not been constructed. Therefore, to expand our set of genes with regionally enriched expression for all brain regions, we then scrutinized literature from PubMed. We obtained a list of Medline records using Boolean logic with search term combinations indicated in Table 3. To facilitate retrieval of publications from a large literature database such as PubMed, we also developed a semi-automated literature mining strategy (see Methods and Figure 3) based on natural language processing. In this approach we looked for the appearance of a gene name or synonym and a brain region in a sentence. Of the 99.7 million sentences searched, 314,515 occurrences of a brain region term were found; 4,395 mouse genes names, or the names of their human orthologs, were found to appear within the same sentence as a brain region (not shown).

**Table 3:** Boolean search terms to obtain Medline records with information about region-associated expression or promoter characterization

Gene AND brain AND in situ [qualifiers: Mouse/Human]
Gene AND brain region AND in situ
Gene AND regulation
Gene AND promoter
Gene AND promoter AND brain
Gene AND promoter AND brain region
Gene AND promoter AND transgenic mice
Gene AND promoter AND reporter (qualifiers: CAT/Luciferase/Gfp)

The candidature of literature-mined genes was verified by assessing available expression data (reporter gene expression, microarray expression profile, radioactive/non-radioactive *in situ *hybridization) in publications, and confirmed with *in situ *hybridization data from the Allen Brain Atlas (see below). In addition to promoter-reporter fusion data from the literature, reporter expression data for BAC (Bacterial Artificial Chromosome) transgenic mice, when available from the GENSAT database [18], was also considered as complementary evidence of expression [see Additional file 2].

### Data mining genes showing regionally enriched expression from Allen Brain Atlas

The entire Allen Brain Atlas (ABA) data set can be searched via a web-based application [13,14]. We used this feature to examine expression patterns of genes identified as regionally enriched by SAGE and/or the literature. This verification was particularly apt for SAGE because ABA *in situ *hybridization patterns were also derived from the same mouse strain C57BL/6J. We also employed the ABA Anatomic Search tool to identify additional genes whose expression patterns cluster within brain regions of interest. While this approach short-listed genes for major regions (Thalamus, Cerebral Cortex etc.) of the mouse brain listed under Anatomic Search, we also searched within these regions to identify expression in sub-regions of interest, e.g. within Pons for genes expressed in Locus Coeruleus. Recent introduction of the alternative ABA search tool, NeuroBlast, also proved to be useful. We used NeuroBlast to retrieve genes co-expressed with a seeded (query) gene in a region of interest. Identification of regionally enriched co-expressed genes in this manner is indispensable in subsequent identification of shared regulatory elements for efficient mini-promoter design.

Thus, SAGE analysis of the adult mouse brain transcriptome combined with meta-analysis using data mining resources described above identified 237 genes as showing regionally enriched expression (Table 4). A summary of the meta-analysis that supports regionally enriched expression is presented [see Additional file 2]; where available, this file includes examples of supporting ABA images downloaded from the ABA website (please see Availability & requirements for more information)

**Table 4:** List of regionally enriched genes in 30 brain regions and cell-types of therapeutic interest

**Brain Regions/Cell types**	**Example Processes/Disease Associations**	**Genes**
Cortex	Alzheimer Disease, Amyotropic Lateral Sclerosis, Plasticity	*B3galt2, 3110035E14Rik, Ccl27, Ctgf, Emx1, Fhl2, Klf10, Myl4, Rbp4, Rtn4rl2, Stx1a, Tbr1, Vip, Ddit4l, Dkkl1, Rspo2, Ier5, Igfbp6, Ephb6, Mpped1, Pak7, Satb2, Cplx3, E430002G05Rik*
Hippocampus	Alzheimer Disease Adult Neurogenesis, Depression, Plasticity	*Htr1A, Tgfb2, Gria1, Nr3c2*
Hippocampus, Ammon's Horn	Alzheimer Disease Adult Neurogenesis, Depression, Plasticity	*Hunk, Klk8, Gpr161, Arfrp2, C630041L24Rik, Slc9a2, Neurod6, Pkp2, Fibcd1, Sstr4*
Hippocampus, Dentate Gyrus	Alzheimer Disease Adult Neurogenesis, Depression, Plasticity	*Gabrd, Prox1, Dsp, C78409, Lct, Crlf1, Tdo2, A330019N05Rik, Lrrtm4, Htr4, Tspan18*
Neurogenic Regions	Adult Neurogenesis	*Nr2e1, Dcx, Mki67, Vim, Dlx2, Nes, Dlx1, Dscam, Fabp7, Igfbpl1, Lrrn1, Rrm1, Sox2, Thbs4*
Striatum	Huntington Disease, Parkinson Disease, Plasticity in Depression	*Adora2a, Gpr88, Drd1a, Drd2, Gpr6, Rgs9, Adcy5, Crym, Foxp1, Lpl, Pde1b1, Pdyn, Rarb, Rasd2, Tgfa*
Amygdala	Huntington Disease, Depression, Plasticity	*Tac1, Cyp26b1, Hap1, Cdh9, Ptprc, Gabra2, Hgf, Pdzrn3, Plxnd1, Wwox, Rasal1, Dock10, Prkcd*
Amygdala, Basolateral Complex	Huntington Disease, Depression, Plasticity	*Grp, Nov, Nr2f2*
Amygdala, Central Nucleus	Huntington Disease, Depression, Plasticity	*Atp6v1c2*
Thalamus	Huntington Disease	*Ramp3, Rgs16, Slitrk6, Tnnt1, 1110069I04Rik Amotl1, Rab37, Sh3d19, Grid2ip, Lef1, Plekhg1, Syt9, Tcf7l2, Gm804, Gja7, Socs6, Vangl1*
Hypothalamus	Cancer	*Hcrt, Gpx3, Trh, Fezf1, Agrp, Calcr, Ghrh, Npy, Pmch, Pomc*
Cerebellum, Granule Cells	Medulloblastoma, Ataxia, Cerebellar hypoplasia	*Gabra6, Cbln3*
Cerebellum, Purkinje Cell Layer	Spinocerebellar Ataxia, Autism, Plasticity	*Pcp2, Hbegf, Icmt, Atp2a3, Casq2, Gdf10, Grid2, Hes3, Lhx1, Ptprm, A930006D11Rik*
Basal Nucleus of Meynert	Acetylcholine System, Alzheimer Disease	*Gal, Ngfr, Tac2, Lhx8, Ecel1, Gbx1, Lancl3, Ntrk1*
Substantia Nigra	Dopamine System, Parkinson Disease	*Ddc, Slc6a3, Ntsr1, Pitx3, Aldh1a1, Chrna6, Chrnb3, Th*
Raphe Nuclei	Norepinephrine System, Depression	*Fev, Gchfr, Slc6a4, Slc17a8, Tph2, Maob, Esr2*
Locus Coeruleus	Serotonin System, Depression	*Dbh, Maoa, Slc6a2, Slc18a2*
Astroglia	Alzheimer Disease	*Gfap, S100b, Slc1a2, Plaur, Gcm1, Gcm2, Serpina3n*
Microglia (activated)	Alzheimer Disease, Amyotropic Lateral Sclerosis	*Cd68, Aif1, P2rx7, Sulf2*
Microglia (constitutive)	Alzheimer Disease	*Cx3cr1, Itgam*
Oligodendroglia	Alzheimer Disease, Multiple Sclerosis	*Olig1, Ugt8a, Cnp, Gjb1, Klk6, Mag, Apod, Enpp2, Fa2h, Mal, Mbp, Mobp, Mog, Olig2, Pllp, Plp1, Sox10, Tmem63a*
Barrington's Nucleus	Pain	*Crh, Fgfr1*
Brainstem, Pons and Medulla	Pain	*Slc6a5, Glra1, Pogz, Anxa4, Spp1, Esr1, Pou4f1, Slc4a2, Stac*
Cortex, Anterior Cingulate	Pain	*Egr1, Stmn1, Cckbr*,*Adcy1*
Cortex, Somatosensory	Pain	*Rspo1, Cyp39a1, Cartpt, Col5a1, Rorb, Loc433228, Gnb4*
Cortex, Insula	Pain	*Lxn, Ntng2, Nr4a2, Fezf2, Ttc9b*
Hypothalamus, Paraventricular Nucleus	Pain	*Avp, Oxt*
Subthalamic Nucleus	Pain	*Pitx2, Lmx1b*
Blood Brain Barrier	Drug therapy	*Abcb1a, Cldn5, Ednra, Fcgrt, Hspa12b, Lrp10, Lrp8, Rage, Slc2a1, Slc7a5, Slco1c1, Slc6a12, Slc28a2*
GABAergic neurons	Schizophrenia, Bipolar Disorder	Vip*, *Gpr88*^‡^

*also listed as a cortex-specific gene

^‡^also listed as a striatum-specific gene

### Identification of over-represented GO terms among genes with region-enriched expression

The Gene Ontology (GO) resource [19] is a powerful tool to identify common functions shared by genes identified by high-throughput gene expression methods such as SAGE. We searched for over-representation of GO terms among our set of genes from each of three ontology classes: Biological Process, Molecular Function and Cellular Component (Methods). Of 237 genes in our selection, we found annotations for 216 genes in the whole mouse genome set of 18535 annotated genes (as of March 18, 2008). From this list, we determined the top 12 statistically over-represented GO terms [see Additional file 3]. Annotations for the test selection of genes were compared with GO annotations of the whole mouse genome. Significant biological processes involved nervous system development, transmission of nerve impulse, cell-cell signaling, neurogenesis, behavior etc. Significant molecular functions involved neuropeptide hormone activity, sequence-specific DNA binding, neurotransmitter receptor activity, steroid hormone receptor activity, neurotransmitter transporter activity etc. Products of some of these genes also tended to be localized in the extracellular region, plasma membrane, synapse, or within transcription factor complexes. Thus, it appears that many of the genes we identified have established neurological functions, which accounts for their regionally enriched expression. It is noteworthy that we found 28 transcription factor encoding genes representing 16 of 30 regions/cell-types of interest (Table 5). This information combined with identification of regulatory sequences within promoters of selected genes will aid the design of mini-promoters specific for each brain region. Because our selection of the 237 genes was biased towards those with known functions, we also carried out GO analysis on genes expressed in each of 18 SAGE libraries [see Additional file 4]. Specific neurological functions were less apparent among over-represented GO terms for these larger sets than for the 237 genes presented in this study.

**Table 5:** Regionally enriched genes encoding transcription factors

**Gene**	**Transcription Factor Description**	**Associated Brain Region**
*Nr2f2*	Nuclear receptor subfamily 2, group F, member 2	Amygdala, Basolateral Complex
*Gbx1*	Gastrulation brain homeobox 1	Basal Nucleus of Meynert
*Lhx8*	LIM homeobox protein 8	Basal Nucleus of Meynert
*Esr1*	Estrogen receptor 1	Brainstem (Pons and Medulla)
*Pou4f1*	POU domain, class 4, transcription factor 1	Brainstem (Pons and Medulla)
*Lhx1*	LIM homeobox protein 1	Cerebellum, Purkinje Cell Layer
*Emx1*	Empty spiracles homeobox 1	Cortex
*Tbr1*	T-box brain gene 1	Cortex
*Egr1*	Early growth response 1/Zinc finger protein 225	Cortex, Anterior Cingulate
*Nr4a2*	Nuclear receptor subfamily 4, group A, member 2	Cortex, Insula
*Nr3c2*	Nuclear receptor subfamily 3, group C, member 2	Hippocampus
*Neurod6*	Neurogenic differentiation 6; Basic HLH transcription factor	Hippocampus, Ammon's Horn
*Dlx1*	Distal-less homeobox 1	Neurogenic
*Dlx2*	Distal-less homeobox 2	Neurogenic
*Nr2e1*	Nuclear receptor subfamily 2, group E, member 1	Neurogenic
*Sox2*	SRY (sex determining region Y)-box 2	Neurogenic
*Esr2*	Estrogen receptor 2	Raphe Nuclei
*Foxp1*	Forkhead box P1	Striatum
*Rarb*	Retinoic acid receptor, beta:	Striatum
*Pitx3*	Paired-like homeodomain transcription factor 3	Substantia Nigra
*Lmx1b*	LIM homeobox transcription factor 1, beta	Subthalamic Nucleus
*Pitx2*	Paired-like homeodomain transcription factor 2	Subthalamic Nucleus
*Lef1*	Lymphoid enhancer binding factor 1	Thalamus
*Tcf7l2*	Transcription factor 7-like 2 (T-cell specific, HMG-box)	Thalamus
*Gcm1*	Glial cells missing homolog 1	White Matter – Glia, Astrocytes
*Gcm2*	Glial cells missing homolog 2	White Matter – Glia, Astrocytes
*Olig1*	Oligodendrocyte transcription factor 1	White Matter – Glia, Oligodendroglia
*Olig2*	Oligodendrocyte transcription factor 2	White Matter – Glia, Oligodendroglia
*Sox10*	SRY (sex determining region Y)-box 10	White Matter – Glia, Oligodendroglia

## Discussion

Targeting gene therapy to specific regions of the brain requires the application of well-defined promoters that can drive expression in a region-specific manner. In this study our goal was to identify regionally enriched transcripts in sub-structures/cell-types of the mouse brain with a particular focus on those brain regions associated with diseases. We were encouraged by findings from the ABA project that above background level expression was found for ~80% of genes assayed – and approximately 70% of genes have been localized to fewer than 20% of all brain cells – suggesting that gene expression is clustered in small brain regions [14]. For a variety of reasons we believe that human orthologs of regionally enriched mouse genes would be good candidates to design promoters from. First, at the genomic level, approximately 99% of mouse genes have an ortholog in the human genome [20]. Second, it has been shown that 84% of human-mouse orthologous gene pairs show significantly lower expression divergence than that of random gene pairs [21]. In another comparable study within the milieu of neurogenomics, it was demonstrated that there are significant constraints on the evolution of gene expression and nucleotide sequence of region-specific genes in the brains of humans and mice [1]. In general, transcripts that are regionally enriched in mice also appear to be regionally enriched in humans – further emphasizing conservation of mammalian brain gene expression. Nonetheless, we are exercising caution in assuming global conservation of expression across species as divergent as mouse and human, and will be testing multiple candidate genes for each region.

Our study profiles region-enriched gene expression within 17 key areas of the adult mouse brain by LongSAGE analysis. For the small number of brain regions for which we had no SAGE data we interrogated the literature and the ABA directly. We used several expression indicators including SAGE tag abundance and specificity, *in situ *hybridization, promoter-reporter fusion data etc. to assess candidacy of genes. Our data mining strategy was to start with SAGE-identified genes ranked on the basis of specificity and expression level, confirmed with supporting evidence from the literature, ABA or GENSAT. Although we prioritized finding genes displaying absolute regional specificity (no detectable background expression), for our data mining strategy to be practicable we did not limit ourselves to this level of stringency – especially for the brain nuclei e.g. Basal Nucleus of Meynert, Barrington's Nucleus etc. Therefore, we also selected genes that displayed the highest level of regional enrichment with the idea that promoters of such genes can be manipulated to produce desired specificity of expression, as reported by Machon et al. for the mouse *Dach1 *gene [22]. Compared to ubiquitous expression of the native *Dach1 *gene, a transgene with 5.8 kb of *Dach1 *regulatory sequence restricts β-galactosidase reporter expression within the mouse brain to the neocortex. Deletion analysis of this 5.8 kb fragment further delimited cortex-specific activity to a minimal 2.5 kb promoter region. From a total of about 30,000 mouse genes [20], we have identified a set of 237 genes displaying regional enrichment of expression.

Analysis of SAGE data to delineate transcriptome similarity among 17 selected brain tissues revealed segregation of a large cluster of neuronal tissues from discrete single clusters of non-neuronal tissues (Ependymal tissue and the highly myelinated Cerebellar White Matter tissue) and the neuronal outlier Cerebellar Purkinje Cell Layer. This pattern of tissue clustering appears to be borne out by unique tissue composition at the very least. Among neuronal tissues, tight clustering of the Ventral and Medial Thalamus regions is possibly a reflection of common diencephalic origin, although from a functional standpoint the two tissues can be considered to be different. The expression signature of a tissue may either independently confer tissue uniqueness, or itself depend on unique tissue composition, the surrounding cellular environment, or a combination of factors.

Other studies have also demonstrated the utility of gene expression patterns in assessing cytoarchitectural distinctness of rodent brain regions. During review of this manuscript another study was published that employed SAGE gene expression profiling to identify region expression in 11 regions of the adult mouse brain [23]. Interestingly, regional enrichment of some transcripts was found to be conserved in the human brain. Microarray analysis of gene expression patterns in 24 neural tissues in the mouse central nervous system has mapped discrete brain domains based on such expression patterns [24]. Importantly, it was revealed that embryological imprinting is still evident in the adult brain. Microarray analysis has similarly identified molecular markers for neuronal subtypes in the adult mouse forebrain [25], in brain regions in each of eight strains of inbred mice [26], as well as in the adult rat CNS [27,28]. Fang et al. have shown that the most regionally discriminative genes are associated with one of four specific factors: regional myelin/oligodendrocyte levels, resident neuron types, neurotransmitter innervation profiles, and Ca^+2^-dependent signaling and second messenger systems [28].

By assessing over-representation of GO terms within our set of regionally expressed genes, we identified commonalities in molecular functions, cellular locations and involvement in key biological processes. This offers the promise of a unique set of molecular markers for each region/cell-type, and could potentially provide a mechanistic explanation of unique functions performed by discrete brain regions. Because of the disease application of our work, we were assured by the over-representation of genes involved in neurotransmitter synthesis, reception and degradation. Importantly, we have also identified many regionally expressed transcription factor-encoding genes. This is consistent with previous findings of Suzuki et al. who have identified region-specific transcription factors in 11 mouse brain regions by using medium-scale real-time RT-PCR [29].  They reported that 90% of known transcription factors display significant expression in at least one brain region. Additionally, it was found that 349 of over 1000 transcription factor and co-regulator genes, mapped by *in situ *hybridization in the brains of developing mice, show restricted expression patterns adequate to describe the anatomical organization of the mouse brain [30]. 

The identification of brain region-specific transcription factors is a prelude to explaining expression patterns of similarly enriched genes regulated by these factors. Armed with this knowledge, we can now search for evidence of transcription factor co-regulation of genes by availing of existing repositories of regulatory sequence collections [31-33]. In particular, the PAZAR system [33] has been employed to integrate transcription factor data and annotated regulatory sequences from the Pleiades Promoter Project. Additionally, given that much is already known about pathways that activate transcription factors, it would now be possible to identify pathways with which genes regulated by these transcription factors are associated. Indeed, a regulatory network comprising 15 important basic helix-loop-helix transcription factors and 153 target genes within the mouse brain has now been constructed [34]. From the perspective of the Pleiades Promoter Project, the identification of DNA-binding elements, transcription factors and pathways influencing their interaction will stand in good stead for efficient mini-promoter design.

We encountered challenges during in this study that are deserving of mention. In literature mining, curation was obfuscated by the existence of numerous synonyms for either mouse or human genes, references to a single protein rather than two distinct isoforms, or different genes with the same synonym. Furthermore, where genes were not represented on either ABA or GENSAT it was not possible to confirm expression, but nonetheless such genes were retained based on level and specificity of expression indicated by the literature or SAGE. Additionally, for a good number of genes there was low correlation between expression detected by SAGE and *in situ *hybridization. Despite the depth of sampling, expression of many genes was not detected by our SAGE procedure; for e.g *Pde1b1*, which has been shown to be strongly expressed in the striatum by *in situ *hybridization on ABA and in the literature [35]. Also, *Hcrt *appeared to be Hypothalamus-specific by SAGE but ABA indicated enrichment in the Hypothalamus with low level, widespread background expression. Although our SAGE procedure and ABA *in situ *hybridization profiled gene expression from the same mouse strain C57BL/6J, lack of correlation between the two could be due to inherent differences in the way RNA is processed and/or detected in these procedures. Nonetheless, *Hcrt *was retained in our study after considering significance of expression in SAGE analysis (*P *value = 0) and the description of minimal promoters in the literature [36,37].

## Conclusion

We have successfully identified genes displaying region-enriched expression in the mouse brain by the application of SAGE and data mining from a variety of publicly available sources. These genes represent useful molecular markers that could potentially aid in unraveling the functions of representative brain regions/cell-types. Importantly, for the Pleiades Promoter Project, identification of these genes has brought us closer to our goal of designing well-defined human promoters for gene therapy. Indeed, we have further identified promoters of human orthologs of a subset of these mouse genes, and are now gearing up to test expression of reporter genes in transgenic mice (unpublished data). Ultimately, it will be of great interest to determine for how many of these promoters the mouse pattern of regional enrichment is recapitulated within the human brain, and which of these successfully remediate the disorders they may be designed for.

## Methods

### Mice

Mice used in our experiments were all adult male C57BL/6J mice (12-week old post-natal). All procedures used in these experiments were in accordance with the Canada Council on Animal Care and approved by the University of British Columbia Animal Care Committee (A05-1748). All experiments were conducted in accordance with Canadian and International standards for animal care. All efforts were made to minimize the number and suffering of any animals used in these experiments.

### Whole brain manual dissection and RNA extraction

Whole brains were manually dissected at room temperature from the intact bodies of mice. To minimize the effects of stress on gene expression, the mother, and the entire litter remained in the family cage until harvest. Mice were removed, one at a time and killed in a separate room, by cervical dislocation. Tissue was immediately flash frozen in liquid nitrogen and stored at -80°C until further processing. Frozen tissue was disrupted and homogenized for 30 seconds with a Polytron^® ^PT 1200CL hand-held homogenizer (Kinematica AG, through Brinkmann™ Instruments Inc, Mississauga, Canada) at a setting of 3 (~13,000 RPM), which had been equipped with a 7-mm easy-care generator (PT-DA 1207/2EC). Total RNA was extracted using RNeasy Lipid Tissue Mini Kit (Qiagen Inc., Missisauga, Canada), following the manufacturer's protocol with the modification of using 1.5-ml Phase Lock Gel™ Heavy Tube (Eppendorf Scientific, through Fisher Scientific, Ottawa, Canada) for more robust phase separation. Also, while on the column, samples underwent DNase I treatment during RNA extraction. Standard care was used to avoid RNA degradation: reagents were prepared with diethyl pyrocarbonate (DEPC)-treated water and all surfaces and equipment were treated with an RNase decontamination solution (RNaseZap^® ^and RNaseZap^® ^Wipes; Ambion Inc., Austin, Texas, USA). The quality and quantity of the RNA samples were tested on an Agilent 2100 Bioanalyzer with the RNA 6000 Nano LabChip^® ^Kit (Agilent Technologies Canada Inc., Mississauga, Canada).

### Harvesting adult brain regions by Laser Capture Microdissection (LCM)

Brains (1–3 per region; exception: 7 per Ependymal and Subependymal Layers), recovered as above, were immediately frozen on dry ice and mounted in OCT (Optimal Cutting Temperature) embedding medium. For the Visual Cortex (SM147), Cerebellar White Matter (SM182), Dorsal Striatum (SM195), and Cerebellar Purkinje cells (SM196) sagittal sections were processed, while coronal sections were used for the remaining tissues. Cryosections (20 μm) of fresh-frozen tissues were mounted onto RNase-free membrane slides (Molecular Machines & Industries AG (MMI), Glattbrugg, Switzerland) manufactured for LCM. To identify the desired regions for processing by LCM, each slide was individually stained with a modified Nissl-substance stain using cresyl violet (CV) dye (Polysciences, Inc., Warrington, PA) as follows: Slide-mounted sections were air-dried for 2–3 min and the surrounding OCT medium was rinsed off with 1× PBS (made with DEPC water). Tissue was fixed for 30 sec with 75% ethanol, stained for 1 min with 0.5% CV, then sequentially rinsed for 5–10 sec with 75%, 95%, and 100% ethanol. After air-drying for 2–3 min, sections were immediately dissected with the SL μCUT system (MMI AG; Glattbrugg, Switzerland) under the 10× objective of a Nikon Eclipse TE2000-S, at laser power < 70 mV, for no longer than 15 min. The cut regions were collected onto the adhesive cap of a 500-μl microfuge tube (MMI AG, Glattbrugg, Switzerland) designed for the SL μCUT system, digested with 30 μl lysis buffer RLT (RNeasy Micro Kit; Qiagen Inc., Missisauga, Canada), and transferred from the cap to the vial. The samples were vortexed, centrifuged for 5 sec, and then stored at -80°C until RNA extraction (as above). High-quality samples were pooled within groups for SAGE library generation.

### SAGE library preparation

The LongSAGE-Lite method was used to construct the libraries as previously described [5]. In brief, first strand cDNA was synthesized with Powerscript Reverse Transcriptase (Clontech, BD Biosciences, Mississauga, Canada) and LITE1/LITE TS primer mix (Invitrogen, Carlsbad, CA) using 15–120 ng of DNase-treated total RNA, and amplified by a 20-cycle PCR according to the SAGE-Lite method [38]. SAGE-Lite biochemistry for the generation of full-length cDNA libraries is based upon the SMART (Switching Mechanism At the 5' end of RNA Transcripts) cDNA synthesis strategy (Clontech, BD Biosciences, Mississauga, Canada). Following amplification, the cDNA were processed according to an adaptation of the standard LongSAGE protocol using the I-SAGE Long kit (Invitrogen, Carlsbad, CA). The SAGE protocol includes steps of anchoring by *Nla*III, tagging by *Mme*I, and generating 131 bp ditags by T4 DNA ligase. The 131 bp ditags were amplified using the scale-up PCR varying from 23–27 cycles depending on the optimal scale up condition as described in the protocol, and were digested with *Nla*III to remove adapter sequences. Purified 36-bp ditags were ligated to form concatemers that were cloned into *Sph*I-digested pZErO-1 vector (Invitrogen, Carlsbad, CA), and transformations were done using One Shot DH10B T1 electrocompetent *E. coli *(Invitrogen, Carlsbad, CA).

After transformants had been screened by colony PCR, the fraction containing concatemers of sizes ranging from 900 bp-1300 bp was chosen for sequencing. Colonies were picked using a Q-Pix robot (Genetix, Beaverton, OR) and inoculated into 2xYT media with Zeocin (50 μg/ml) and glycerol (7.5%). After overnight culture, glycerol stocks were used to inoculate larger volume cultures for plasmid preparation, carried out using a standard alkaline-lysis procedure adapted for high-throughput processing with microtiter plates. DNA sequencing was performed with BigDye v3.1 dye terminator cycle sequencing reactions run on Tetrad thermal cyclers (MJ Research, Waltham, MA). Products from the sequencing reaction were purified by ethanol precipitation and then run on capillary DNA sequencers (Model 3730xl, Applied Biosystems, Foster City, CA).

Following inspection of data quality from a first 384-well sequencing plate, each library was sequenced to a depth of > 100,000 raw tags. The resulting sequence data were collected automatically and processed by both trimming the reads for sequence quality and removing sequences from non-recombinant clones, vector DNA and linker-derived tags. Processed data can be found on the Mouse Atlas website (please see Availability & requirements for more information)

### SAGE data analysis

To obtain high quality SAGE tags for this study, all raw SAGE tags underwent a three-step cluster modification process developed by Siddiqui et al. [8]. In the first step, we calculated for each tag a *P *value based on the *Phred *quality score [39] to identify single nucleotide variants likely to originate from sequencing error. In the second step, we used tag sequence clustering to group such variants to combine tags likely to originate from a common transcript. Thus, some singletons were clustered and counted as a more abundant tag. The third step was to filter out low quality tags and compare each *P *value to a meta-library *P *value calculated from all SAGE libraries. Tag-to-gene-mapping was then carried out using DiscoverySpace 4.0 application [40]. All cluster-modified tags were then mapped to transcripts in the NCBI Reference Sequence Collection [41]. The remaining unmapped tags were mapped to transcripts in the Mammalian Gene Collection [42], followed by the Ensembl database [43]. Only sense transcripts and unique mappings were considered, and tags that mapped to more than one transcript in any of the three transcript databases were discarded. The three mapping results were subsequently merged based on gene symbol.

For each gene, a *P *value was assigned to each target (TL; brain region of interest) and off-target (OTL; background region) library pair using the *P *value option in DiscoverySpace. The *P *value was computed based on Audic-Claverie algorithms [44] to assess confidence level of differential expression between two transcript libraries. A ranking system was implemented to facilitate selection of candidate genes with specific or enriched expression in each target library (Table 2). Region-specific transcripts were obtained by selecting transcripts detected with 5 tags or more only in one target library. To identify region-enriched transcripts, those detected in one target library and one off-target library (*P*_*TL*-*OTL *_value < = 0.05) were selected. Transcripts detected in multiple libraries were ranked based on pre-defined *P *value limits of differential expression (*P*_*TL*-*TL*_, *P*_*TL*-*OTL*_), as well as additional criteria such as target and off-target library counts. Transcripts whose expression patterns did not fit these criteria were not ranked.

To analyze transcriptome similarity of tissues, a dendrogram was generated using MATLAB 7 (The MathWorks, Natick, MA) based on hierarchical clustering using the Unweighted Pair Group Method with Arithmetic Mean (UPGMA). The input data is a list of objects (tissue SAGE libraries) with their pair-wise distances (expression divergence ED; see below), and the output is a dendrogram. Initially, each object is in its own cluster; then, at each step of the hierarchical clustering the nearest two clusters are combined into a higher-level cluster. The distance between any two clusters A and B is taken to be the average of all distances between pairs of objects in A and B. Thus, we defined pair-wise distance or expression divergence (ED) between any two tissues as the fraction of differentially expressed genes in their corresponding SAGE libraries, using the formula:

ED_(*p*) _= N_diff(*p*)_/N

(N_diff(*p*) _= number of differentially expressed genes for a given *P *value, N = number of shared genes between two corresponding libraries).

### Semi-automated Literature mining

All synonyms for 28,000 mouse genes were obtained from Entrez (RefSeq release 14) combined with Ensembl (build 34) of the mouse genome. Synonyms for the human orthologs were obtained using Compara (Ensembl build 34) to identify similarities between human and mouse together with Homologene (version 47) for homolog detection. In each case, Ensembl and Entrez were used as cross-references for gene identifiers. From these search strings, all names found in the English dictionary were subtracted to remove obfuscating gene terms such as "Ice". Abstracts were parsed from Medline (extraction performed September 7, 2006) and the complete text of articles were parsed from PubMed Central [45], and converted into individual sentences using the medical sentence parser [46]. Each sentence was searched for the co-occurrence of gene names with brain regions of interest. For each brain region, expanded search terms were applied referring to finer structures appropriate to the region as defined by the ontology available from the Allen Brain Atlas website [13]. The number of sentences with gene names and brain regions obtained is greater than the number of sentences with only brain regions because of the plural nature of both search terms. We scrutinized retrieved publications for details indicating regionally enriched/specific expression in a brain region.

### Gene Ontology over-representation analysis

Gene Ontology [19] over-representation analysis was performed for the 237 genes using the BiNGO [47] plug-in for the Cytoscape [48] software package. Significance of over-representation of GO terms was calculated using the hypergeometric test, corrected for multiple testing with a Benjamini & Hochberg false discovery rate correction [49], and a cut-off of 0.05 was applied to the result. The test selection of 237 genes was compared to all GO annotated genes in the mouse genome (18535 genes, as of March 18, 2008).

## Abbreviations

SAGE: Serial Analysis of Gene Expression; LCM: Laser Capture Microdissection; OCT: Optimal Cutting Temperature; CV: Cresyl violet; DEPC: Diethyl Pyrocarbonate; ABA: Allen Brain Atlas; BAC: Bacterial Artificial Chromosome; GENSAT: Gene Expression Nervous System Atlas; UPGMA: Unweighted Pair Group Method with Arithmetic Mean; ED: Expression Divergence; GO: Gene Ontology.

## Availability & requirements

The Pleiades Promoter Project: 

ABA website: ; Seattle (WA): Allen Institute for Brain Science ^© ^2004–2007; in accordance with ABA Terms of Use and Citation Policy.

Mouse Atlas website: 

## Authors' contributions

CAD analyzed SAGE data, ABA *in situ *hybridization data and mined the PubMed database to identify region-enriched genes, carried out GO analysis, and drafted this manuscript. VC analysed SAGE data, ABA *in situ *hybridization data, mined the PubMed database to identify region-enriched genes, and contributed to the compilation of gene expression summaries in Additional file 2. DG confirmed candidature of SAGE and literature mined genes by inspecting ABA images. RV and LLCL performed bioinformatics analysis of SAGE data. Y–YX and SB laser-microdissected tissues for construction of SAGE libraries. YZ participated in SAGE library construction. MB did the hierarchical clustering analysis of tissue transcriptomes utilizing the java script written by AH. EP–C participated in data mining and selection of region-enriched genes. EMS conceived of the study, and participated in its design and coordination along with WWW, DG, MAM, RAH, and SJMJ. All authors read and approved the final manuscript.

## Supplementary Material

Additional file 1 Compilation of SAGE data for 237 regionally enriched genes. Click here for file

Additional file 2 Summary of expression profiles of region-specific or enriched genes by subanatomical region. Click here for file

Additional file 3 Top 12 over-represented GO terms in each ontology category among the 237 regionally enriched genes. Click here for file

Additional file 4 Top 10 over-represented GO terms in each ontology category among the genes in each of 18 SAGE libraries. Click here for file

## References

[B1] Strand AD, Aragaki AK, Baquet ZC, Hodges A, Cunningham P, Holmans P, Jones KR, Jones L, Kooperberg C, Olson JM (2007). Conservation of regional gene expression in mouse and human brain. PLoS genetics.

[B2] Wasserman WW, Palumbo M, Thompson W, Fickett JW, Lawrence CE (2000). Human-mouse genome comparisons to locate regulatory sites. Nature genetics.

[B3] Gotz J, Probst A, Spillantini MG, Schafer T, Jakes R, Burki K, Goedert M (1995). Somatodendritic localization and hyperphosphorylation of tau protein in transgenic mice expressing the longest human brain tau isoform. Embo J.

[B4] Velculescu VE, Zhang L, Vogelstein B, Kinzler KW (1995). Serial analysis of gene expression. Science.

[B5] Khattra J, Delaney AD, Zhao Y, Siddiqui A, Asano J, McDonald H, Pandoh P, Dhalla N, Prabhu AL, Ma K (2007). Large-scale production of SAGE libraries from microdissected tissues, flow-sorted cells, and cell lines. Genome research.

[B6] Saha S, Sparks AB, Rago C, Akmaev V, Wang CJ, Vogelstein B, Kinzler KW, Velculescu VE (2002). Using the transcriptome to annotate the genome. Nat Biotechnol.

[B7] The Mouse Atlas of Gene Expression Project. http://www.mouseatlas.org.

[B8] Siddiqui AS, Khattra J, Delaney AD, Zhao Y, Astell C, Asano J, Babakaiff R, Barber S, Beland J, Bohacec S (2005). A mouse atlas of gene expression: large-scale digital gene-expression profiles from precisely defined developing C57BL/6J mouse tissues and cells. Proceedings of the National Academy of Sciences of the United States of America.

[B9] The Pleiades Promoter Project. http://www.pleiades.org/.

[B10] The NCBI PubMed Database. http://www.pubmed.com.

[B11] De Bruijn B, Martin J (2002). Getting to the (c)ore of knowledge: mining biomedical literature. Int J Med Inform.

[B12] Scherf M, Epple A, Werner T (2005). The next generation of literature analysis: integration of genomic analysis into text mining. Brief Bioinform.

[B13] The Allen Brain Atlas Database. http://www.allenbrainatlas.org/.

[B14] Lein ES, Hawrylycz MJ, Ao N, Ayres M, Bensinger A, Bernard A, Boe AF, Boguski MS, Brockway KS, Byrnes EJ (2007). Genome-wide atlas of gene expression in the adult mouse brain. Nature.

[B15] McCarthy M (2006). Allen Brain Atlas maps 21,000 genes of the mouse brain. Lancet Neurol.

[B16] Simone NL, Bonner RF, Gillespie JW, Emmert-Buck MR, Liotta LA (1998). Laser-capture microdissection: opening the microscopic frontier to molecular analysis. Trends Genet.

[B17] Nakai J, Fujita S (1994). Early events in the histo- and cytogenesis of the vertebrate CNS. The International journal of developmental biology.

[B18] The GENSAT Database. http://www.gensat.org.

[B19] Ashburner M, Ball CA, Blake JA, Botstein D, Butler H, Cherry JM, Davis AP, Dolinski K, Dwight SS, Eppig JT (2000). Gene ontology: tool for the unification of biology. The Gene Ontology Consortium. Nature genetics.

[B20] Waterston RH, Lindblad-Toh K, Birney E, Rogers J, Abril JF, Agarwal P, Agarwala R, Ainscough R, Alexandersson M, An P (2002). Initial sequencing and comparative analysis of the mouse genome. Nature.

[B21] Liao BY, Zhang J (2006). Evolutionary conservation of expression profiles between human and mouse orthologous genes. Molecular biology and evolution.

[B22] Machon O, Bout CJ van den, Backman M, Rosok O, Caubit X, Fromm SH, Geronimo B, Krauss S (2002). Forebrain-specific promoter/enhancer D6 derived from the mouse Dach1 gene controls expression in neural stem cells. Neuroscience.

[B23] Brochier C, Gaillard MC, Diguet E, Caudy N, Dossat C, Segurens B, Wincker P, Roze E, Caboche J, Hantraye P (2008). Quantitative gene expression profiling of mouse brain regions reveals differential transcripts conserved in human and affected in disease models. Physiological genomics.

[B24] Zapala MA, Hovatta I, Ellison JA, Wodicka L, Del Rio JA, Tennant R, Tynan W, Broide RS, Helton R, Stoveken BS (2005). Adult mouse brain gene expression patterns bear an embryologic imprint. Proceedings of the National Academy of Sciences of the United States of America.

[B25] Sugino K, Hempel CM, Miller MN, Hattox AM, Shapiro P, Wu C, Huang ZJ, Nelson SB (2006). Molecular taxonomy of major neuronal classes in the adult mouse forebrain. Nat Neurosci.

[B26] Letwin NE, Kafkafi N, Benjamini Y, Mayo C, Frank BC, Luu T, Lee NH, Elmer GI (2006). Combined application of behavior genetics and microarray analysis to identify regional expression themes and gene-behavior associations. J Neurosci.

[B27] Stansberg C, Vik-Mo AO, Holdhus R, Breilid H, Srebro B, Petersen K, Jorgensen HA, Jonassen I, Steen VM (2007). Gene expression profiles in rat brain disclose CNS signature genes and regional patterns of functional specialisation. BMC genomics.

[B28] Fang H, Tong W, Shi L, Jakab RL, Bowyer JF (2004). Classification of cDNA array genes that have a highly significant discriminative power due to their unique distribution in four brain regions. DNA and cell biology.

[B29] Suzuki H, Okunishi R, Hashizume W, Katayama S, Ninomiya N, Osato N, Sato K, Nakamura M, Iida J, Kanamori M (2004). Identification of region-specific transcription factor genes in the adult mouse brain by medium-scale real-time RT-PCR. FEBS letters.

[B30] Gray PA, Fu H, Luo P, Zhao Q, Yu J, Ferrari A, Tenzen T, Yuk DI, Tsung EF, Cai Z (2004). Mouse brain organization revealed through direct genome-scale TF expression analysis. Science.

[B31] Sandelin A, Wasserman WW (2004). Constrained binding site diversity within families of transcription factors enhances pattern discovery bioinformatics. Journal of molecular biology.

[B32] Montgomery SB, Griffith OL, Sleumer MC, Bergman CM, Bilenky M, Pleasance ED, Prychyna Y, Zhang X, Jones SJ (2006). ORegAnno: an open access database and curation system for literature-derived promoters, transcription factor binding sites and regulatory variation. Bioinformatics (Oxford, England).

[B33] Portales-Casamar E, Kirov S, Lim J, Lithwick S, Swanson MI, Ticoll A, Snoddy J, Wasserman WW (2007). PAZAR: a framework for collection and dissemination of cis-regulatory sequence annotation. Genome Biol.

[B34] Li J, Liu ZJ, Pan YC, Liu Q, Fu X, Cooper NG, Li YX, Qiu MS, Shi TL (2007). Regulatory module network of basic/helix-loop-helix transcription factors in mouse brain. Genome Biol.

[B35] Polli JW, Kincaid RL (1994). Expression of a calmodulin-dependent phosphodiesterase isoform (PDE1B1) correlates with brain regions having extensive dopaminergic innervation. J Neurosci.

[B36] Sakurai T, Moriguchi T, Furuya K, Kajiwara N, Nakamura T, Yanagisawa M, Goto K (1999). Structure and function of human prepro-orexin gene. The Journal of biological chemistry.

[B37] Waleh NS, Apte-Deshpande A, Terao A, Ding J, Kilduff TS (2001). Modulation of the promoter region of prepro-hypocretin by alpha-interferon. Gene.

[B38] Peters DG, Kassam AB, Yonas H, O'Hare EH, Ferrell RE, Brufsky AM (1999). Comprehensive transcript analysis in small quantities of mRNA by SAGE-lite. Nucleic acids research.

[B39] Ewing B, Green P (1998). Base-calling of automated sequencer traces using phred. II. Error probabilities. Genome research.

[B40] Robertson N, Oveisi-Fordorei M, Zuyderduyn SD, Varhol RJ, Fjell C, Marra M, Jones S, Siddiqui A (2007). DiscoverySpace: an interactive data analysis application. Genome Biol.

[B41] The NCBI Reference Sequence Collection. http://www.ncbi.nlm.nih.gov/RefSeq.

[B42] The Mammalian Gene Collection. http://mgc.nci.nih.gov.

[B43] The Ensembl Database. http://www.ensembl.org.

[B44] Audic S, Claverie JM (1997). The significance of digital gene expression profiles. Genome research.

[B45] Greenberg DS (1999). National Institutes of Health moves ahead with "PubMed Central". Lancet.

[B46] Berman JJ (2003). Improved Medical Sentence Parser. Arch Pathol Lab Med.

[B47] Maere S, Heymans K, Kuiper M (2005). BiNGO: a Cytoscape plugin to assess overrepresentation of gene ontology categories in biological networks. Bioinformatics (Oxford, England).

[B48] Shannon P, Markiel A, Ozier O, Baliga NS, Wang JT, Ramage D, Amin N, Schwikowski B, Ideker T (2003). Cytoscape: a software environment for integrated models of biomolecular interaction networks. Genome research.

[B49] Benjamini Y, Hochberg Y (1995). Controlling the False Discovery Rate: a Practical and Powerful Approach to Multiple Testing. Journal of the Royal Statistical Society, Series B.

