# Supplementary material for: Identification of a set of genes showing regionally enriched expression in the mouse brain
Source: BMC Neurosci. 2008 Jul 14;9:66. doi: 10.1186/1471-2202-9-66 (PMC2483290; doi:10.1186/1471-2202-9-66)
Supplement: Additional file 2 — Summary of expression profiles of region-specific or enriched genes by subanatomical region. [file 1471-2202-9-66-S2.doc]

**Summary of expression profiles of region-specific or enriched genes by subanatomical region**

**Amygdala……………………………………………………………………………………..3**

**Amygdala, Basolateral Complex…………………………………………………..17**

**Amygdala, Central Nucleus………………………………………………………...21**

**Barrington’s nucleus………………………………………………………………….22**

**Basal Nucleus of Meynert.……..…………………………………………………...25**

**Blood brain barrier...………………………………………………………………….36**

**Brainstem, Pons and Medulla………………………………………………………52**

**Cerebellum…………………………………………………………………………………61**

**Cerebellum Granule Cells……………….………………………………………….61**

**Cerebellum, Purkinje Cell Layer…………..……………………………………….64**

**CEREBRAL CORTEX…………………………………………………………………………76**

**Cortex, Anterior Cingulate…………………………………….………………...........…..104**

**Cortex, Insula…………………………………………...…………………………...111**

**Cortex, Somatosensory……………………………………………………………120**

**Glia……………………………………………………………………………………………128**

**Astroglia………………...……………………………………………………………128**

**Microglia (constitutive)………………………………………….…………………137**

**Microglia (activated)……………………………………………..…………………139**

**Oligodendroglia……………………………..………………………………………144**

**Hippocampus…………………………………...………………………………………….162**

**Hippocampus, Ammon’s Horn.......................................................................167**

**Hippocampus, Dentate Gyrus........................................................................179**

**Hypothalamus…………………………………....……………………………………...190**

**Hypothalamus, Paraventricular Nucleus…………………………………..…...203**

**Locus Coeruleus……………………………………......……………………………...205**

**Neurogenic regions…………………………………………...……………………...210**

**Raphe Nuclei………………………………………………………………………………230**

**Striatum…………………………………………………………………………………….239**

**Substantia Nigra…………………………………………………………...…………..259**

**Subthalamic Nucleus…………………………………………………………………269**

**Thalamus…………………………………………………………………………………...272**

**REFERENCES……………………………………………………………………………......289**

**Subanatomical region: Amygdala, Therapeutic interest: Huntington Disease, Depression, Plasticity**

The amygdalae are almond-shaped groups of neurons located deep within the medial temporal lobes of the brain in complex vertebrates. The regions described as amygdalae encompass several nuclei with distinct functional traits [1]. Among these nuclei are the basolateral complex, the centromedial nucleus and the cortical nucleus. The amygdalae are considered part of the limbic system, and therefore, perform a primary role in the processing and memory of emotional reactions; abnormalities of the amygdala result in anxiety disorders. As mentioned in the main text of this manuscript, the amygdala was a particularly difficult region to find region-specific genes, and for that reason, genes with region-enriched expression were included.

**1. *Cyp26b1* (Cytochrome P450, Family 26, Subfamily b, Polypeptide 1)**

**ABA review:** This gene is expressed in the frontal cortex region including larger neurons in deep layers of motor cortex and smaller neurons of superficial layers of orbital cortex, and agranular insular cortex at rostral and posterior levels. Other regions include neurons in bed nucleus of stria terminalis, amygdala (basomedial, lateral, basolateral and central nuclei and amygdalopiriform transition area, CA3c, hilar pyramidal cells, and subiculum and entorhinal cortex of hippocampal system, brainstem motor and sensory nuclei (vestibular, cochlea, hypoglossal), cerebellar internal granule cell layer and nuclear region, and the choroid plexus. In an independent ABA review, low expression was found in the piriform area of the cortex. In the second set of sections, there was moderate expression in the Purkinje cell layer. In addition, there was significant expression throughout the dentate gyrus and in the subiculum. There was also some diffuse labeling in the midbrain.


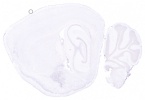

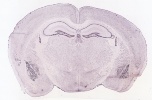

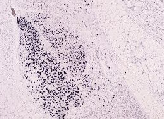


Sagittal section Coronal section Coronal (zoomed)

**GENSAT:** No information available

**Literature:** In the adult murine brain *Cyp26b1* is expressed in the CA3 region of hippocampus, subiculum and amygdaloid nuclei [2].

**2. *Hap1* (Huntingtin-Associated Protein 1)**

**ABA review:** In the main olfactory bulb moderate-to-high expression was observed in the glomerular, mitral and granular layers; similar levels of labeling could also be seen in layer 2 of the dorsal peduncular area and the taenia tecta. In the anterior olfactory nucleus, there was high expression in the granular layer. In the cerebral cortex moderate labeling could be found in layers 2/3 and 4; in particular there was high expression in layer 2 of the posterolateral visual area. Moderate-to-high expression could be found in the rostroventral segment of the lateral septal nucleus, rostral. In the thalamus, there was also moderate-to-high staining in the paraventricular nucleus, intermediodorsal nucleus, medial habenula, and subparafascicular area. Similar levels of labeling could be found throughout the hypothalamus. However, high expression levels were present in the arcuate hypothalamic nucleus. Moderate-to-high labeling was found in the central amygdalar nucleus and medial amygdalar nucleus whereas moderate amounts were observed in the basolateral amygdalar nucleus. In the hippocampal formation there was moderate-to-high expression in the granule cell layer of the dentate gyrus. Slightly lower levels of staining seen along the length of the pyramidal cell layer of Ammon’s horn. In the midrain there was moderate-to-high expression in the periaqueductal gray. In the pons there was strong labeling for *Hap1* in the nucleus of the lateral lemniscus and in the locus coeruleus. In the medulla there were moderate-to-high levels of expression in the nucleus of the solitary tract dorsal motor nucleus of the vagus nerve, ventral part of the anteromedial nucleus, and lateral reticular nucleus. In addition, moderate levels of expression were present in the spinal tract of the trigeminal nerve. It should be noted that there was significant background in the midbrain and brainstem.

An independent review indicated expression throughout the hypothalamus and also in the amygdala (preferential to central nuclei), nucleus of the solitary tract and other brainstem regions.


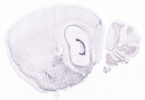

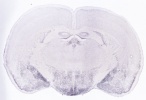

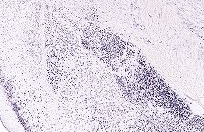


Sagittal section Coronal section Coronal (zoomed)

**GENSAT:** There is no expression data for this gene in the adult; images were only available for stage P7. The GENSAT BAC data was consistent with the literature and the *in situ* data from Allen database.

**Literature:** Hap1-B immunostaining of the adult mouse brain shows abundant expression in the hypothalamus, accessory olfactory bulb, superior and inferior colliculi, pedunculopontine nucleus, and brain stem [3]. A similar expression pattern in the adult mouse brain was shown by *in situ* hybridization [4].

**3. *Cdh9* (Cadherin 9)**

**ABA review:** There was strong expression in deep cortex, striatum, amygdaloid region, all hippocampal neurons and in the inferior olivary complex of the brainstem. An independent review indicated high expression in the hippocampal formation and in the striatum-like amygdalar nuclei. *Cdh9* also appeared to be expressed at a moderate level in the nucleus accumbens and to some extent the lateral septal complex.


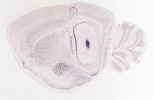

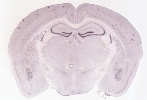

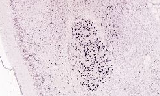


Sagittal section Coronal section Coronal (zoomed)

**GENSAT:** No information available

**Literature:** This gene was associated with expression in the amygdala in a microarray study [5].

**4. *Ptprc* (Protein Tyrosine Phosphatase, Receptor Type C)**

**ABA review:** Moderate but highly specific expression in the striatum-like amygdalar nuclei. An independent review indicated very discrete expression pattern in amygdaloid region, clearly marking the basolateral amygdala. Expression was found to be very specific and there was almost no expression elsewhere.


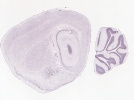


Sagittal section

**GENSAT:** No information available

**Literature:** Amygdala basolateral specific expression has been shown in a microarray study [5].

**5. *Gabra2* (Gamma-Aminobutyric acid (GABA) A Receptor, Alpha 2)**

**ABA review:** Low-to-moderate expression was observed in the anterior and posterior parts of the basomedial amygdalar nucleus and in the posterior amygdalar nucleus. Slightly lower levels of labeling were also observed in the CA3 pyramidal layer, the facial motor nucleus, and the spinal nucleus of the trigeminal (caudal portion).

An independent review indicated discrete, light punctate label in what appeared to be all cells of the amygdala. The amygdala positive cells appeared to include the medial to lateral and anterior to posterior extents of the structure. This characteristic staining pattern was the same for hippocampus pyramidal cells, layer 2 cortical neurons and a few other neuronal classes, including the cerebellar nuclear neurons, but still rather specific.


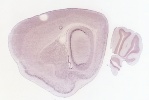

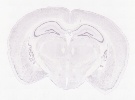

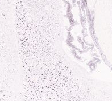


Sagittal section Coronal section Coronal section (zoomed)

**GENSAT:** No information available

**Literature:** In the adult rat brain, *Gabra2* mRNA is found only in the olfactory bulb, cerebral cortex, caudate putamen, hippocampal formation, and certain lower brain stem nuclei [6].

**6. *Hgf* (Hepatocyte Growth Factor**)

**ABA review:** Labeling in the ABA images is very faint for *Hgf*. However, there was low but specific expression in the striatum-like amygdalar nuclei. An independent review indicated that there was expression in the amygdala (lateral and basomedial), CA3 pyramids, layer 4 cortex, posteriorly in parabigemical nucleus and a smattering of cells in agranular insular cortex.


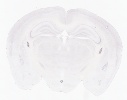

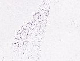


Coronal section Coronal section (zoomed)

**GENSAT:** In E15.5 telecephalon, the BAC produces staining in the outer layer of the cortical plate, whereas the literature claims that both the ventricular zone and the cortical plate are labeled. During postnatal development, the BAC produces scattered, stained interneurons in the cortex, which is in agreement with the role of Hgf as a motogen for cortical interneurons. There are three regions of moderate-to-strong expression in the adult, namely, the amygdala, cerebellum and hippocampus.

**Literature:** This product is also called hepatopoietin A or scatter factor. *In situ* hybridization localized *Hgf* mRNA in cerebral cortex, hippocampus and amygdala in the rat brain [7]. Consistently, specific localization of Hgf protein in neurons of these regions was detected by immunohistochemical analysis, and non-neuronal glial cells in cingulum, cerebellum, pons and medulla were also specifically stained. Another study indicated that in the adult rat brain, there is strong expression in the hippocampus, particularly in the dentate gyrus [8]. Lower signals were detected in all cortical areas, most prominently in the superficial layers II-m and layer V. A strong signal was seen in the ependymal cells lining the brain ventricles, as well as in the chorioid plexus. In the hindbrain, *Hgf* mRNA is expressed in the pontine nuclei and in the deep cerebellar nuclei. A relatively strong signal was seen in cerebellar granule neuron.

**7. *Pdzrn3* (PDZ Domain Containing Ring Finger 3)**

**ABA review:** *Pdzrn3* was expressed at moderate levels within layers 1 and 2 of the cerebral cortex.


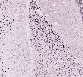

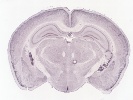

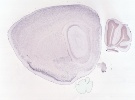


Sagittal section Coronal section Coronal (zoomed)

**GENSAT:** No information available

**Literature:** Microarray analysis revealed *Pdzrn3* is regionally enriched in the adult rat cerebral cortex [9].

**8. *Plxnd1***

**ABA review:** There was significant expression in layers 2 and 4 of the cerebral cortex. In addition, there was high expression throughout the caudoputamen and nucleus accumbens. There was also significant expression in the striatum-like amygdalar nuclei and the magnocellular nucleus of the pallidum.

An independent review indicated very nice expression in the caudate/accumbens, cortex layers 2 and 4, CA1 pyramidal cells, and amygdala, with very discrete label in the lateral and posterior amygdaloid nuclei.


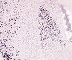

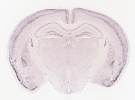

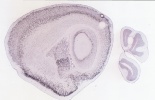


Sagittal section Coronal section Coronal (zoomed)

**GENSAT:** BAC data is consistent with the literature and the *in situ* data from Allen Brain Atlas database. The BAC produces expression in the endothelial cells of blood vessels as well as in cortex and striatum.

**Literature:** In the literature, expression of this gene in the amygdala was indicated in a microarray experiment [5].

**9. *Wwox* (WW Domain Containing Oxidoreductase)**

**ABA review:** Moderate levels of punctate expression could be seen in layers 3, 4, 5, 6a of the cerebral cortex. Similar levels of expression were seen in the anterior olfactory nucleus, posteroventral part and the substantia innominata. Moderate staining was also seen in the lateral amygdalar nucleus and basolateral amygdalar nucleus. Moderate expression was seen in the layer 2 of the dorsal zone of the medial segment of the entorhinal area. Low-to-moderate labeling could also be seen in the layers2a/2b of the lateral segment of the entorhinal area. In the medulla there was moderate staining in dorsal motor nucleus of the vagus nerve and the magnocellular part of the lateral reticular nucleus. Finally, moderate-to-high expression was observed in the cerebellar Purkinje cell layer.

An independent review indicated that expression is specific for amygdala especially in the basomedial and lateral complexes, also layer VI cortex.

**
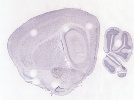

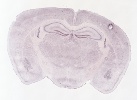

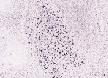
**

Sagittal section Coronal section Coronal (zoomed)

**GENSAT:** No information available

**Literature:** No relevant brain expression studies have been reported.

**10. *Rasal1* (Ras Protein Activator Like 1)**

**ABA review:** *Rasal1* was found to be expressed at high levels within the cortex, amygdala and hippocampal formation with the exception of the striatum.


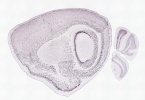

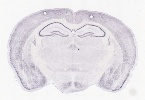

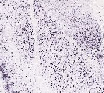


Sagittal section Coronal section Coronal (zoomed)

**GENSAT: T**he BAC data overlaps with BGEM *in situ* hybridization data. There are some minor discrepancies:

1. In cerebellum, the BAC reproducibly produces expression in interneurons of the internal granule cell layer.

2. By epifluorescence, the dentate gyrus is the site of highest expression in P7 BAC transgenic mice. 3. At E15-5, the *in situ* data shows expression primarily around the ventricular zones, which is not clearly seen in the BAC data. The BAC data overlaps with *in situ* data from genepaint.org. However, the BAC detects extra expression in dorsal medulla and spinal cord. This could be due to the different time points used in the two studies (E15-5 vs E14-5 (*in situ*).

**Literature:** SAGE analysis revealed regional enrichment in the orbital cortex of adult C57BL/6 mice [10].

**11. *Dock10* (Dedicator of Cytokinesis 10)**

**ABA review:** *Dock10* is expressed at high levels in the hippocampal dentate gyrus and the granule cell layer, and in the lateral and medial parts of the central amygdalar nucleus. Moderate expression levels were present in the glomerular and granular layers of the main olfactory bulb. Low-to-moderate expression could be found throughout the striatum; however, there appeared to be moderate-to-high expression in the rostral (rostroventral) part of the lateral septal nucleus. In the pallidum there was high expression in the bed nuclei of the stria terminalis, anterior division and the oval nucleus. Moderate labeling could also be seen in the rostral portion of the bed nuclei of the stria terminalis. In the hypothalamus there was expression in the medial preoptic nucleus. Finally, there was some enrichment of expression in the islands of Calleja in the olfactory tubercule.

**
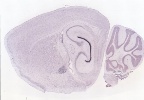

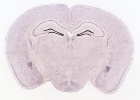

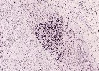
**

Sagittal section Coronal section Coronal (zoomed)

**GENSAT:** No information available

**Literature:** No relevant brain expression studies have been reported.

**12. *Prkcd* (Protein Kinase C, Delta)**

**ABA review:** There was very high level of expression in the striatum-like amygdalar nuclei, thalamus, and globus pallidus. In addition, there was also moderate-to-high level expression in medulla and the Purkinje cells.


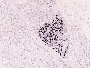

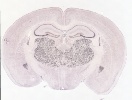

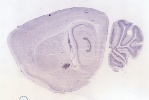


Sagittal section Coronal section Coronal (zoomed)

**GENSAT:** The BAC data matches both literature and BGEM *in situ* data. There are 11 regions of moderate-to-strong expression in the adult: amygdala, basal forebrain, cerebellum, hippocampus, hypothalamus, medulla, pons, septum, spinal cord dorsal horn, spinal cord ventral horn and thalamus.

**Literature:** SAGE analysis revealed regional enrichment of *Prkcd* in the thalamus of the adult C57BL/6 mouse [10]. Microarray analysis revealed *Prkcd* is regionally enriched in the adult mouse midbrain [11]. Immunostaining detected protein in thalamocortical systems, parietal cortex, cerebellar Purkinje neurons, and in the caudate-putamen of the rat brain [12].

**13. *Tac1* (Tachykinin 1)**

**ABA review:** One review found moderate expression in the posteroventral and posterodorsal portions of the medial amygdalar nucleus. There is very high expression in the olfactory tubercule, caudoputamen, nucleus accumbens and fundus of striatum, arcuate hypothalamic nucleus and tuberal nucleus. There also seems to be significant expression in the medulla, the spinal tract of the trigeminal nerve and ventral spinocerebellar tract. *Tac1* expression is also seen in the midbrain but does not seem to be present in the superior or inferior colliculus. Another review indicated particularly strong expression in striatum and nucleus accumbens. Expression is also found in clustered cells just posterior to accumbens, most likely the medial amygdalar region, deep part of colliculus, some brainstem nuclei, periacqueductal grey area, habenula, layer 5-6 cortical neurons, VMH hypothalamus, and striatum.


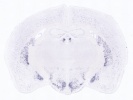

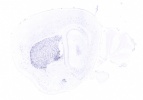


Sagittal section Coronal section

**GENSAT:** Striatal expression in BAC transgenic mice is not as strong as the literature suggested. In E15-5 BAC embryos, expression in the caudate putamen is almost undetectable. BAC reveals distinct cerebellar expression at P7, not supported by the literature; other extra expression sites also include thalamus.

**Literature:** The protein encoded by this gene is also called substance P. SAGE analysis revealed regional enrichment of *Tac1* in the caudate-putamen of adult C57BL/6 mice [10]. Among the areas of the rodent brain examined, only the striatum contained neurons labelled with the antisense tachykinin RNA [13]. A remote and highly conserved enhancer supports amygdala specific expression (medial amygdaloid nucleus and in the central amygdaloid nucleus) [14]. Microarray analysis revealed enrichment of *Tac1* in the adult rat striatum [15].

**Subanatomical region: Amygdala, BASOLATERAL COMPLEX. Therapeutic interest: Huntington Disease, Depression, Plasticity**

(See description of Amygdala under Subanatomical region:
Amygdala)

**1. *Grp* (Gastrin-Releasing Peptide)**

**ABA review:** *Grp* is expressed in the lateral and basolateral amygdala, lateral hippocampus, subiculum, the nuclei of lateral lemniscus, and sporadic, but specific, cell groups in cortex, forebrain, brainstem, etc. An independent review of the ABA images found expression in the striatum-like amygdalar nuclei. Thus, the strongest data suggests *Grp* mRNA is enriched in amygdala - but not specific.

**
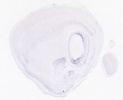

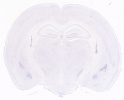

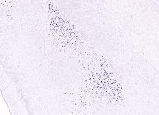
**

Sagittal section Coronal section Coronal (zoomed)

**GENSAT:** There are 15 regions of moderate-to-strong expression in the adult: amygdala, cerebellum, cerebral cortex, entorhinal cortex, hippocampus, hypothalamus, medulla, midbrain, olfactory bulb, piriform cortex, pons, spinal cord dorsal horn, spinal cord ventral horn, subicular cortex, and thalamus. Two BAC transgenic lines have matching expression at P7. The BAC data is consistent with the literature in general. Expression in cerebellum and striatum has not been reported in the literature. The level of expression decreases in adult BAC mice.

**Literature:** *In situ* hybridization detected enrichment of *Grp* mRNA in the lateral nucleus and dorsomedial subnuclei of the amygdala in the mouse brain. In addition, strong expression was observed in the medial, ventral, and dorsal subdivisions of the medial geniculate body, the posterior intralaminar nucleus (PIN) of the auditory thalamus, the TE3 subregion of the auditory cortex, and the perirhinal cortex [16].

**2. *Nov* (Nephroblastoma Overexpressed Gene)**

**ABA review:** In one review of ABA images, it was found that *Nov* expression is extensive in superficial, mid-level and deep cortex, piriform cortex, hippocampus CA1 and strong in basal and lateral nuclei of amygdala, the continuum between hippocampal CA1 region into amygdaloid-hippocampal region, posteromedial cortical amygdala and the olfactory bulb. An independent review indicated that *Nov* is expressed at high levels throughout the brain with the exception of the striatum and the hippocampal formation.


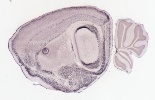

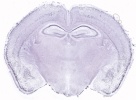

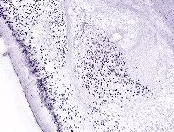


Sagittal section Coronal section Coronal (zoomed)

**GENSAT:** No information available

**Literature:** This gene is also called Ccn3. SAGE analysis revealed regional enrichment of *Nov* in the entorhinal cortex of adult C57BL/6 mice [10]. In situ hybridization detected Nov expression in the cerebral cortex, especially in the temporal cortex (auditory area) and piriform cortex [17]. The striated cortex showed several weakly labeled neurones. Many cells showing strong positive labelling were detected in the CA1 to CA3 regions of the hypocampus, whereas the CA4 region had moderate labelling. The strongest labelling was seen in the amygdala, particularly in the basomedial amygdaloid nucleus. Additonally, there was moderate-to-high labelling of thalamic and hypothalamic neurons.

**3. *Nr2f2* (nuclear receptor subfamily 2, group F, member 2)**

**ABA review:** One review found low-level expression in the striatum-like amygdalar nuclei. Another review indicated expression in the claustrum, cells of the bed nucleus anterior hypothalamus, reticular nucleus of thalamus and then much of amygdala (lateral, basolateral, basomedial, anterior cortical, medial) except central nuclear region. This gene is also expressed in VPL/VPM thalamus, Purkinje cells and periaqueductal regions


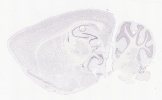

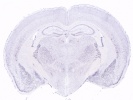


Sagittal section Coronal section

**GENSAT:** GENSAT describes 20 regions of strong to moderate expression in the adult: amygdala, anterior olfactory nucleus, caudate putamen, cerebral cortex, corpus callosum, entorhinal cortex, fornix, hippocampus, hypothalamus, inferior colliculus, longitudinal fasciculus of pons, medulla, midbrain, olfactory bulb, piriform cortex, pons, spinal cord dorsal horn, stria terminalis, thalamus, and ventral striatum. Two BAC transgenic mice lines have very similar expression at P7. The data is consistent with the literature and in situ data. However, the choroid plexus and ventrolateral thalamic area are not stained in adult BAC transgenic mice. Absence of expression in choroid plexus contrasts with the literature, but is consistent with the in situ data. In addition, the BAC transgene reveals extra expression sites in cortex and olfactory bulb; these sites are confirmed by in situ data. The P7 in situ data detects stronger hybridization signals in thalamus.

**Literature:** This gene is also called *ARP-1*. In the adult mouse brain, high expression of *ARP-1* was detected in the reticular, the ventral lateral and the gelatinosus thalamic nuclei [18]. Other hot spots of *ARP-1* mRNA expression were the amygdaloid nucleus and the arachnoid membranes.

**Subanatomical region: Amygdala, Central Nucleus. Therapeutic interest: Huntington Disease, Depression, Plasticity**

(See description of Amygdala under Subanatomical region:
Amygdala)

**1. *Atp6v1c2* (Vacuolar H+ ATPase C2 Isoform B)**

**ABA review:** There was very limited expression in the brain. The central and medial nuclei of the amygdaloid nucleus are the principle cell groups that are labeled. In more anterior sections scattered cells in the accumbens are also discretely labeled. According to an independent ABA review, the images for Atp6v1c2 did not show any convincing expression.


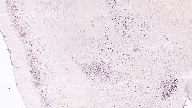

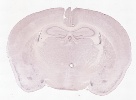

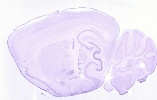


Sagittal section Coronal section Coronal (zoomed)

**GENSAT:** No information available

**Literature:** No relevant brain expression studies have been reported.

**Subanatomical region: Barrington’s nucleus, Therapeutic Interest: Pain**

Barrington’s nucleus (BN) in the pons is a small group of neurons located bilaterally in the pontine tegmentum, immediately ventromedial to the locus coeruleus (LC) [19]. BN neurons are highly responsive to a variety exteroceptive and/or interoceptive stressors [20]. BN is known to be the micturition center that acts as a switch to induce bladder contraction in response to distention [21]. In addition, BN activation has been shown to induce increases in colonic intraluminal pressure [22].

**1) *Crh* (Corticotropin Releasing Hormone)**

**ABA review:** Moderate-to-high expression was seen in Barrington’s nucleus and in the locus coeruleus. Within the medulla high expression was seen in the inferior olivary complex, the magnocellular reticular nucleus, nucleus raphe (magnus and pallidus), and the paragigantocellular reticular nucleus. In addition, significant expression was also observed in the medial and lateral portions of the superior central nucleus raphe. As in *Fgfr1*, low diffuse expression of *Fgfr1* was observed throughout the brain.

**
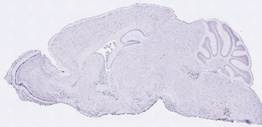

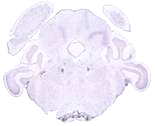
**

Sagittal section Coronal section

**GENSAT:** Strong to moderate expression is found in 8 brain regions: amygdala, anterior olfactory nucleus, cerebral cortex, hippocampus, olfactory bulb, piriform cortex, pons, and septum. The BAC data is consistent with the literature and the *in situ* data from ABA. Confirmed expression is observed in brainstem, hypothalamus, main olfactory bulb, cortex and piriform cortex. At P7, the BAC expresses in colliculi and cerebellum. But, these sites contain mostly stained fibers. The cerebellar expression with stained projection fibers persists into adulthood. BGEM data failed to detect any specific hybridization signals.

**Literature:** *In situ* hybridization detected *Crh* mRNA in numerous regions of the adult mouse brain, including most prominently the paraventricular nucleus of the hypothalamus, the inferior olivary nucleus, and Barrington's nucleus [23].

**2) *Fgfr1* (Basic Fibroblast Growth Factor Receptor 1 Precursor)**

**ABA review:** Moderate-to-high expression was seen in Barrington’s nucleus and the laterodorsal tegmental nucleus. Slightly lower levels of expression were also observed in the medial mammillary nucleus of the hypothalamus and in the hippocampal formation where it was highest in the CA3. In addition, significant expression was also observed in the medial and lateral portions of the superior central nucleus raphe and the pontine gray of the pons. Low diffuse expression of *Fgfr1* could also be seen throughout the brain.


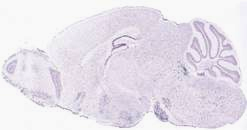

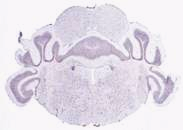


Sagittal section Coronal section

**GENSAT:** In the adult mouse strong to moderate expression is found in 17 brain regions: amygdala, basal forebrain, caudate putamen, cerebellum, cerebral cortex, hippocampus, hypothalamus, leptomeninges, medulla, midbrain, olfactory nucleus, pons, rostral migratory stream, septum, thalamus, ventral horn, and ventral striatum. There are papers reporting expression of *Fgfr1* mRNA in both neurons and glia. Two BAC lines have matching expression at P7. The BAC expresses in both neurons and glia. The overall expression pattern is consistent with the literature and ABA *in situ* hybridization data. At E15-5, scattered cells are labeled in the ventral zone. The post-mitotic cells are prominently stained due to the stability of EGFP protein.

**Literature:** *In situ* hybridization and immunohistochemistry localized *Fgfr1* mRNA and protein to cells in the normal adult human hippocampus and caudal entorhinal cortex [24]. *Fgfr1* is ubiquitously expressed in the adult rat brain [25].

**Subanatomical region: Basal Nucleus of Meynert, Therapeutic Interest: Acetylcholine System, Alzheimer Disease**

The basal nucleus of Meynert (NBM) is a group of neurons located in the substantia innominata and provides the major source of cholinergic input to the cerebral cortex. Acetylcholine (ACH) release in the brain has been hypothesized to mediate attentional processes (arousal, alertness, wakefulness) as well as learning and memory [26]. In late stages of Alzheimer disease, the NBM undergoes degeneration resulting in a severe reduction in ACH levels, which is associated with a general decrease of mental capacity and learning. The majority of pharmacological treatments of dementia attempt to compensate for a faltering NBM function by artificially increasing acetylcholine levels [27]. In Parkinson disease, Lewy body formation and neurodegeneration has been observed in the NBM [28].

**1) *Gal* (Galanin)**

**ABA review:** Very high expression of *Gal* was present at the border between the substantia innominata and the bed nucleus of the stria terminalis. Similar levels of labeling were located in the 3 distinct regions of the hypothalamus: the medial preoptic area, lateral hypothalamic area and the posterior hypothalamic area. In the brainstem very high levels of expression could be found in the pontine central gray, nucleus of the solitary tract and paragigantocellular reticular nucleus, lateral part. Moderate, but significant, expression was seen throughout the main olfactory bulb. In an independent review of the ABA images, it was noted that *Gal* was present in discrete populations of neurons including larger neurons of the vertical limb of the diagonal band, and a few large cells in the medial aspect of the globus pallidus where cells of the basal nucleus of Meynert partly reside, periglomerular (possibly tufted) cells in the olfactory bulb, cells in anterior/medial segments of the hypothalamus, parts of the bed nucleus of the stria terminalis, and cells of the locus coeruleus. In addition, the cells of the inferior olive, nucleus of the solitary tract, anterior thalamus, supraoptic region and parts of the paraventricular nucleus are also stained.


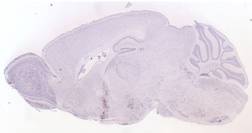

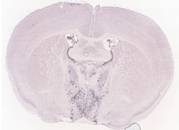


Sagittal section Coronal section

**GENSAT:** In the adult mouse moderate-to-strong expression is found in 9 brain regions: amygdala, basal forebrain, cerebellum, hypothalamus, lateral olfactory tract, medulla, olfactory bulb, piriform cortex, and pons. The BAC data is consistent with the literature and the *in situ* data from Allen database. Correct expression sites include hypothalmus, basal forebrain, amygadala, locus coeruleus, raphe nuclei and the nucleus tractus solitarii. There are papers reporting *Gal* mRNA is expressed in cerebellar Purkinje cells. The BAC data shows EGFP transgene expression predominantly in cerebellar granule neurons. The BAC also detects expression in main olfactory bulb, dentate gyrus (mainly at P7) and scattered cells in cortex at P7. BGEM *in situ* failed to detect any specific hybridization signals. At E15-5, expression in trigeminal ganglia and dorsal spinal cord is confirmed by the E14-5 in situ data from GenePaint.

**Literature:** In the human brain, *GAL*-immunoreactive cells are restricted largely to the basal nucleus of Meynert and to the supraoptic, ventromedial and posterior areas of the hypothalamus [29]. Fibre staining is seen throughout the hypothalamus and in the diagonal band, septum, amygdala, hippocampus and scattered throughout the cortex. Galanin fiber hypertrophy within the anterior nucleus basalis subfield is a late-stage Alzheimer disease response, which may play a role in regulating the cholinergic tone of remaining basocortical projection neurons [30].

**2) *Ngfr* (Nerve Growth Factor Receptor)**

**ABA review:** Very strong punctuate labeling of *Ngfr* was observed along the substantia innominata. However, there were also moderate levels of expression throughout the hypothalamus, brain stem, cerebral cortex and cerebellum. In an independent review, it was noted that there was quite a lot of expression in anterior forebrain regions and layer 2-3 of all cortex. Very discrete and robust staining could be seen in both the vertical and horizontal limbs of the diagonal band. In addition, there was labeling of Purkinje cells and scattered cells in brainstem.


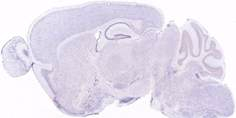

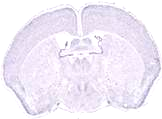

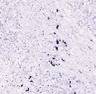


Sagittal section Sagittal (zoomed) Coronal section

**GENSAT:** In the adult mouse strong to moderate expression is found in 14 brain regions: basal forebrain, cerebellum, cerebral cortex, dorsal column fasciculus, dorsal funiculus, endopiriform nucleus, entorhinal cortex, fornix, lateral lemniscus, olfactory bulb, optic tract, pons, spinal trigeminal tract, and subicular cortex. The BAC data is reproducible and matches BGEM *in situ* data and the literature. There are a few additional expression sites in the BAC data that are not reported by the literature, but are reproducible among different BAC founder lines.

**Literature:** Also called p75NTR (low-affinity nerve growth factor receptor). By immunohistochemistry it was shown that in normal aged human brain, NGFR+ neurons were located within the medial septum, vertical and horizontal limb nuclei of the diagonal band, and nucleus basalis [31]. Positively stained cells were also seen within the bed nucleus of the stria terminalis, the anterior commissure, the internal capsule, and the internal and external medullary laminae of the globus pallidus. Greater than 95% of NGFR+ neurons colocalize with the specific cholinergic marker - choline acetyltransferase. Using RNA amplification from anterior nucleus basalis (NB) neuron and cDNA arrays, it was shown that while *TRKB* and *TRKC* mRNAs were selectively down-regulated in the NB neurons, p75NTR mRNA levels remained stable in end stage Alzheimer disease [32].

**3) *Tac2* (Tachykinin 2)**

**ABA review:** High levels of expression of *Tac2* could be seen in the basal nucleus of Meynert and the globus pallidus. Very high levels of staining were present in the third ventricle. High expression could also be identified along the region bordering the rostroventral portion of the lateral septal nucleus and the nucleus accumbens. Similar levels of labeling were found in the anterior division of the bed nuclei of the stria terminalis including the oval nucleus, ventral nucleus, rhomboid nucleus, anterolateral area and the anteromedial area. In the hypothalamus, high expression was noted in the lateral hypothalamic area. Somewhat lower and sparse expression was also observed in the hypothalamic medial preoptic area, arcuate nucleus, zona incerta and the posterior segment of the periventricular nucleus. In addition, there was moderate staining in the glomerular layer of the main olfactory bulb. Finally, there was sparse moderate-to-high punctate labeling throughout the cerebral cortex.


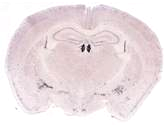

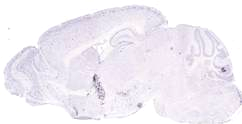


Sagittal section Coronal section

**GENSAT:** In the adult mouse strong to moderate expression is found in 13 brain regions: amygdala, anterior olfactory nucleus, basal forebrain, claustrum, dorsal horn, entorhinal cortex, habenular nucleus, hypothalamus, medulla, midbrain, pons, substantia nigra, and thalamus. The BAC data is consistent with the literature and the *in situ* data from both BGEM and ABA. The adult BAC data also expresses in hippocampus and claustrum that is not obvious in the *in situ* data.

**Literature:** Also called *Nkb*. In the rat brain, *Nkb* immunoreactivity and mRNA are present in many areas including cerebral cortex, hippocampal formation, amygdaloid complex, bed nucleus of the stria terminalis, ventral pallidum, habenula, medial preoptic area, arcuate nucleus, and lateral mammillary bodies [33]. In the human brain, *Nkb* mRNA is detected in the rostral hypothalamus, magnocellular basal forebrain, the bed nucleus of stria terminalis, and the anterior hypothalamic area [34]. Scattered Nkb*+* neurons are present in the infundibular and paraventricular nuclei, paraolfactory gyrus, posterior hypothalamic area, lateral division of the medial mammillary nucleus, and amygdala.

**4) *Lhx8* (LIM Homeobox 8)**

**ABA review:** *Lhx8* expression was observed in the substantia innominata at low-to-moderate levels. Similar levels of expression were also found in the medial septal nucleus and in the diagonal band nucleus. In addition, moderate levels of expression were present in the hippocampal formation. An independent review indicated that there was virtually no expression anywhere in brain other than in isolated cells in forebrain and what were likely the basal nucleus of Meynert and the cells of the diagonal band.


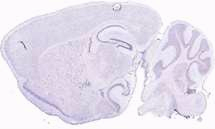

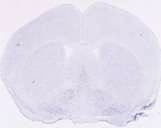

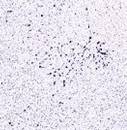


Sagittal section Sagittal (zoomed) Coronal section

**GENSAT:** No information available.

**Literature:** The gene is also called *Lhx7*. In the septum, the most prominent *Lhx7* expression is present in the nucleus of the diagonal band of Broca and the medial septal nucleus of the adult rat brain [35]. *Lhx7* is also detected in all structures of the striatum and the pallidum: the caudate–putamen (punctate pattern), nucleus accumbens, substantia innominata and globus pallidus. In addition, *Lhx7* expression is present in the magnocellular preoptic area, the anterodorsal preoptic area and the lateral preoptic nucleus. A null mutation of *Lhx8* is deficient in the development of forebrain cholinergic neurons; *Lhx8* mutants lack the nucleus basalis, a major source of the cholinergic input to the cerebral cortex [36]. A targeted allele of *Lhx7*was generated by replacing exons 3-5 with a *lacZ* reporter [37]. In heterozygous animals, which were healthy, fertile and had no apparent cellular deficit in the forebrain, β-galactosidase activity reproduced the pattern of expression of the wild-type *Lhx7* locus. However, homozygous mutant mice showed severe deficits in forebrain cholinergic neurons.

**5) *Ecel1* (Endothelin Converting Enzyme-Like 1)**

**ABA review:** There was moderate punctuate labeling of *Ecel1* in the globus pallidus and fundus of striatum. There was strong labeling in the caudoputamen, lateral septal nucleus (rostroventral), bed nuclei of the stria terminalis and substantia innominata. *Ecel1* was expressed at high levels throughout the intercalated amygdalar nucleus and in the capsular and lateral portions of the central amygdalar nucleus. Similar levels of expression were also observed in the layer 3 of the posterior portion (medial zone) of the cortical amygdalar area. Within the hypothalamus, several nuclei displayed high expression levels of *Ecel1* including the zona incerta, anterior hypothalamic nucleus dorsomedial nucleus of the hypothalamus posterior hypothalamic nucleus dorsal and ventral premammillary nuclei. High levels of staining were also seen in cuneiform nucleus of the midbrain and in the motor nucleus of trigeminal of the brainstem. In an independent review of the ABA images, it was noted that there is nice labeling of cells in lateral septum extending into the lateral preoptic and other basal forebrain structures. In addition, there is significant staining in the anterior hypothalamus and bed nucleus of stria terminalis, zona incerta and a band about anterior thalamus. Finally, there is some of sporadic expression in cells of the striatum.


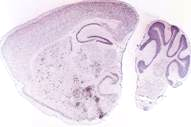

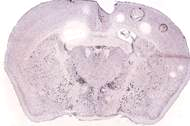


Sagittal section Coronal section

**GENSAT:** No information available.

**Literature:** The gene is also called *Dine*. A discrete but strong hybridization signal is observed in rat striatal (probably cholinergic) interneurons, basal forebrain neurons, and in motoneurons of brainstem (e.g., facial nucleus) and spinal cord. A moderate level of hybridization signal is present in certain thalamic nuclei, zona incerta and spinal cord dorsal horn [38]. In the rat brain, *Dine* expression is detected in the caudate putamen, diagonal band, paraventricular nucleus of the thalamus, hypothalamus, cranial motor nuclei, inferior olive, and substantia gelatinosa of the spinal tract trigeminal nucleus [39]. *Dine* is normally expressed in a group of neurons mainly in the hypothalamus, but not in the cerebral cortex. In the transgenic mice, the expression level of transgenic *Akt* and GFP in normal cortex is low, but can be markedly induced in response to ischemic damage, in a pattern very similar to that of endogenous *Dine* [40].

**6) *Gbx1* (Gastrulation and Brain-specific Homeobox Protein 1)**

**ABA review:** No images were available for *Gbx1.*

**GENSAT:** No information available.

**Literature:** *Gbx1* and *Lhx7* are both expressed in those adult rat brain nuclei that collectively form the basal forebrain cholinergic system, a prime target of neurodegeneration in Alzheimer disease. *Gbx1* expression is restricted to the septum, pallidum and, weakly, the thalamus. Most prominent *Gbx1* signals are detected in the nucleus of the diagonal band of Broca, the medial septal nucleus, the magnocellular preoptic area, the anterodorsal preoptic area and the lateral preoptic nucleus. Weak expression is also observed in the substantia innominata [35].

**7) *Lancl3* (LanC Lantibiotic Synthetase Component C-Like 3)**

**ABA review:** Low-to-moderate expression of *Lancl3* was observed in the medial septal nucleus and substantia innominata. High expression was observed in the mitral layer of the main olfactory bulb. Moderate expression was found in the cerebellar granule layer. Expression seems to be somewhat enriched in the ventral cochlear nucleus of the medulla and the reticular nucleus of the thalamus. Widespread punctuate staining throughout the brain although is reduced in the caudoputamen.


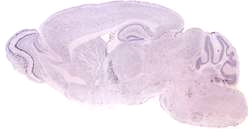

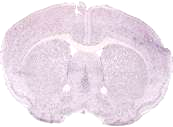


Sagittal section Coronal section

**GENSAT:** No information available.

**Literature:** No expression data for *Lancl3* was found in the literature.

**8) *Ntrk1* (Neurotrophic Tyrosine Kinase, Receptor, Type 1)**

**ABA review:** Moderate punctate staining for *Ntrk1* in the substantia innominata and throughout the caudoputamen. Similar levels of expression are also seen in the diagonal band nucleus and to some extent the olfactory tubercule. Within the midbrain there was moderate-to-high expression in the interpeduncular nucleus. In the brainstem some faint expression was seen in the medial vestibular nucleus. Finally, the cerebellar fiber tracts vestibulocochlear nerve, vestibular nerve trapezoid body had *Ntrk1* labeling. In an independent review of the ABA images, it was noted that rather robust expression in scattered cells in rostral striatum and basal forebrain. In addition, there was labeling of cells in the thalamic paraventricular nucleus and in the horizontal limb of the diagonal band. Finally, sporadic expression could be seen in the basal nucleus of Meynert.


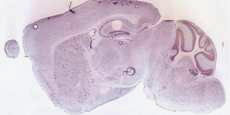

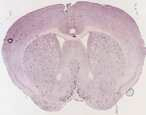

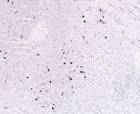


Sagittal section Sagittal (zoomed) Coronal section

**GENSAT:** At P7, the BAC expresses in the visual cortex, septum, basal forebrain, striatum, interpeduncular nucleus and vestibular nucleus of brainstem. At E15-5, the trigeminal ganglia and dorsal root ganglion are labeled. All these expression sites are in agreement with the literature. However, the BAC does produce staining in the prepositus hypoglossal nuclei as the literature indicated. BGEM *in situ* data shows no specific hybridization signals.

**Literature:** In the human brain using immunocytochemical staining, it was shown that *TRKA* (*NTRK1*) was restricted to the basal nucleus of Meynert and was not expressed in the adjacent hypothalamic nuclei [41]. In contrast, *TRKB* and *TRKC* were expressed in basal nucleus of Meynert neurons and in hypothalamic nuclei, including the supraoptic nucleus and tuberomammillary nucleus. This was consistent with previous observations on the anatomical distribution of these receptors in the rat. The immunoreactivity of all three trk receptors was dramatically reduced in the basal nucleus of Meynert of Alzheimer disease brains compared with non-demented controls. In addition to cholinergic neurons in the basal forebrain and neostriatum, *TrkA* expression is present in non-cholinergic neurons in the paraventricular anterior and reuniens thalamic nuclei, the rostral and intermediate subnuclei of the interpeduncular nucleus, scattered neurons in the ventrolateral and paramedian medulla, the prepositus hypoglossal nucleus, and the area postrema [42].

**Subanatomical region: Blood brain barrier, Therapeutic Interest: Drug therapy**

The blood-brain barrier (BBB) acts as a functional interface between the circulatory system and the parenchyma of the brain. The permeability properties of the BBB are largely regulated by the capillary endothelial cells [43]. However, several other cell types make up the brain microvasculature and thereby indirectly influence BBB permeability. Pericytes share a common capillary basement membrane with the endothelial cells. The brain surface of the capillaries is surrounded by astrocytic foot processes. Finally, the capillaries are innervated by axons which may be of intracerebral and extracerebral origin [43]. Disruption of the BBB has been linked to neuroinflammatory diseases of the brain such as multiple sclerosis or meningitis [44]. A number of neurological disorders not normally associated with inflammation have also been shown to have disruptions in the BBB, including Alzheimer, Parkinson and Huntington disease [44, 45]. Successful delivery of therapeutic agents through the BBB remains a major challenge for the treatment of many brain disorders.

**1) *Abcb1a* (ATP-binding cassette, sub-family B (MDR/TAP), member 1A)**

**ABA review:** No reliable expression of Abcb1 could be found in the brain.


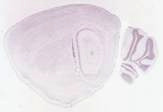


Sagittal section

**GENSAT:** In the adult mouse strong to moderate expression is found in 22 brain regions: amygdala, anterior olfactory nucleus, basal forebrain, caudate putamen, cerebellum, cerebral cortex, dorsal horn, entorhinal cortex, globus pallidus, hippocampus, hypothalamus, medulla, midbrain, olfactory bulb, piriform cortex, pons, septum, subicular cortex, substantia nigra, thalamus, ventral horn, and ventral striatum. Two BAC lines share very similar expression patterns at P7 and adult. The expression sites, such as pia, blood vessels and thalamus, are confirmed by the literature and BGEM *in situ*. At P7, the BAC mice show additional expression in anterior olfactory bulb, brainstem and spinal cord, which cannot be determined from the in situ data.

**Literature:** Also referred to as P glycoprotein 1 (*P-gp*) and *Mdr1*. Using immunogold cytochemistry, it was shown that *P-gp* localizes to both the luminal and abluminal membranes of capillary endothelial cells as well as to adjacent pericytes and astrocytes in rat and human brain [46]. Subcellularly, *P-gp* was distributed along the nuclear envelope, in caveolae, cytoplasmic vesicles, Golgi complex, and rough endoplasmic reticulum. Its presence at the luminal and abluminal poles of the BBB suggests that it may regulate drug transport processes in the entire central nervous system BBB at both the cellular and subcellular level. Using an *in vitro* model of the BBB formed by co-culture of bovine brain capillary endothelial cells (BBCEC) with astrocytes, it was shown that *P-gp* expressed at the BBB is mainly localized in caveolae and its activity may be modulated by interaction with caveolin-1 [47].

**2) *Cldn5* (Claudin 5)**

**ABA review:** Very high levels of expression were seen in the olfactory nerve layer of the main olfactory bulb. Low, moderate and high punctate staining was also observed throughout the brain with no obvious enrichment in any particular subregion. However, punctate staining was somewhat less in the pons and medulla. In an independent review, it was noted that at higher magnification, *Cldn5* staining was exclusive found in the capillary bed of the central nervous system with little to no off target label in other tissue such as the choroid plexus.


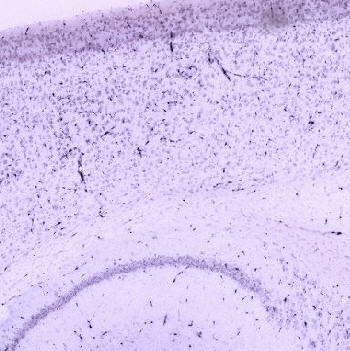

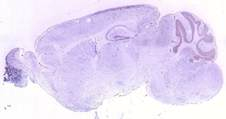


Sagittal section Sagittal (zoomed)

**GENSAT:** In the adult mouse, moderate-to-strong expression is found in 22 brain regions: amygdala, anterior olfactory nucleus, basal forebrain, caudate putamen, cerebellum, cerebral cortex, dorsal horn, entorhinal cortex, globus pallidus, hippocampus, hypothalamus, medulla, midbrain, olfactory bulb, piriform cortex, pons, septum, subicular cortex, substantia nigra, thalamus, ventral horn, and ventral striatum. The BAC data matches the literature with expression in blood vessels and pia only.

**Literature:** Also called *Mbec1* or *Tmvcf*. Tight junctions (TJs) in endothelial cells are thought to determine vascular permeability. Recently, claudins were identified as major components of TJ strands. *Tmvcf* is one of the genes deleted at 22q11.2 and linked to DiGeorge or velocardiofacial syndrome (VCFS), whose hallmarks include heart, limb, and craniofacial anomalies, as well as learning disabilities and increased incidence of schizophrenia [48]. In the adult mouse brain, *Tmvcf* expression is present in all major forebrain subdivisions: the neocortex, hippocampus, basal ganglia, amygdala/basal forebrain, and olfactory bulb, as well as all other CNS regions. *Mbec1* is expressed in cultured mouse brain embryonic cells and in freshly isolated *Mbec1* as early as embryonic Day 7 [49]. *In situ* hybridization and immunocytochemical analyses revealed the presence of the *Mbec1* mRNA and its protein product in brain capillary endothelial cells, as well as in a subset of other endothelial and epithelial cells. In the brain and lung, immunofluorescence microscopy showed that *Cldn5*/*Tmvcf* was exclusively concentrated at cell-cell borders of endothelial cells of all segments of blood vessels, but not at those of epithelial cells [50].

**3) *Ednra* (Endothelin Receptor Type A)**

**ABA review:** Images are not available for Ednra.

**GENSAT:** No information available.

**Literature:** Also referred to as *ETa*. The properties of brain capillary endothelial cells (BCECs) have been analyzed [51]. BCECs were found to express two types of receptor sites for endothelins (ETs), an *ETa* -like receptor and an *ET*b-like receptor. Infusion with S-0139, an *ETa* antagonist, has been shown to result in significant reduction of brain injury and plasma extravasation after transient middle cerebral artery occlusion [52]. Thus, ETa may contribute to cerebral ischemia/reperfusion injury at least partly by increasing the BBB permeability.

**4) Fcgrt (IgG Receptor FcRn Large Subunit p51 Precursor)**

**ABA review:** Images are not available for *Fcgrt*.

**GENSAT:** No information available.

**Literature:** Also referred to as neonatal *FcR* (*FcRn*). Immunocytochemical studies using an antibody to the rat neonatal FcRn, determined that protein was located at the brain microvasculature and choroid plexus epithelium [53]. Co-localization with the *Glut1* glucose transporter indicated that the brain microvascular *FcRn* was expressed in the capillary endothelium. It has been suggested that the capillary endothelial *FcRn* may mediate the reverse transcytosis of IgG in the brain to blood direction. There is an age-dependent increase in IgG-assisted clearance of amyloid beta peptide by the blood-brain barrier *FcRn* in Alzheimer disease [54]. Inhibition of the FcRn pathway in older Alzheimer amyloid model APPsw^+/-^ mice blocked clearance of endogenous beta peptide by centrally administered Abeta immunotherapy. Moreover, deletion of the *FcRn* gene in wild-type mice inhibited clearance of endogenous mouse beta peptide by systemically administered anti-Aβ. This data suggests that the *FcRn* pathway at the BBB plays a crucial role in IgG-assisted Aβ removal from the aging brain.

**5) *Hspa12b* (Heat shock 70kD protein 12B)**

**ABA review:** Moderate expression was present throughout the brain and including the lateral ventricle. However, in the cerebellum there moderate-to-high expression in the cerebellar Purkinje and underlying granular cell layers. In some slides there appears to be moderate-to-high expression in the mitral and granular cell layers of the main olfactory bulb. In an independent review of the ABA images, it was noted that several populations of neurons in brain labeled with *Hspa12b* including the cerebellar Purkinje cell and hippocampal neurons. However, there did not appear to be any significant staining of the vasculature.


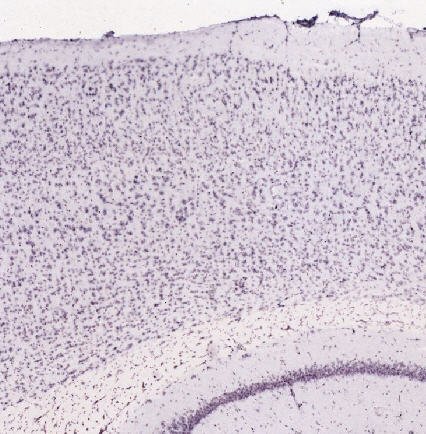

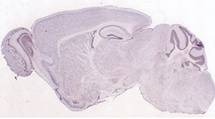


Sagittal section Sagittal (zoomed)

**GENSAT:** In the adult mouse strong to moderate expression is found in 22 brain regions: amygdala, anterior olfactory nucleus, basal forebrain, caudate putamen, cerebellum, cerebral cortex, dorsal horn, entorhinal cortex, globus pallidus, hippocampus, hypothalamus, medulla, midbrain, olfactory bulb, piriform cortex, pons, septum, subicular cortex, substantia nigra, thalamus, ventral horn, and ventral striatum. Two BAC lines are identical at P7. Our BAC shows strong and exclusive expression in pia and blood vessels. The BAC data is also consistent with BGEM *in situ* hybridization data. However, the P7 *in situ* data in BGEM shows presence of *Hspa12b* mRNA in restricted thalamic nuclei, which is not detectable in the BAC data.

**Literature:** *HSPA12B* transcript is only sparsely present in the human brain, and the expression levels are uniform across the investigated brain regions including cerebellum, medulla, spinal chord, putamen, temporal, frontal and occipital cortices [55]. Evidence from, *in situ* hybridization, western blot, immunohistochemistry, and *HSPA12B*-BAC-EGFP transgenic mice is consistent with HSPA12B being predominantly expressed in endothelial cells lining blood vessels [56]. These results suggest that *HSPA12B* may play a role in angiogenesis.

**6) *Lrp10* (Low-Density Lipoprotein Receptor-Related Protein 10)**

**ABA review:** Moderate-to-high expression of *Lrp10* was present in the lateral ventricle and the lateral recess. Similar levels of expression could be seen in the in the cerebellar Purkinje cell layer and the underlying granular layer. Moderate expression was present in the mitral and granular cell layers of the main olfactory bulb. In the hippocampal formation there was moderate labeling in the granular layer of the dentate gyrus and in the pyramidal layer of the CA1, CA2 and CA3 fields. Finally, low level punctate staining could also be seen throughout the brain.


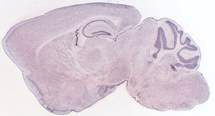


Sagittal section

**GENSAT:** In the adult mouse strong to moderate expression is found in 2 brain regions: cerebral cortex and olfactory bulb. No detailed central nervous system expression literature was found. One paper reports that the mRNA is localized in pia, ependyma of the third ventricle and choroid plexus in mouse brains. These sites are also EGFP+ in BAC mice.

**Literature:** This gene is also referred to as *Lrp9*. *In situ* hybridizations detected *Lrp9* transcripts in peritubular capillaries, choroid plexus, ependyma of the third ventricle, pia matter, and hippocampus. In particular, high levels of expression were observed in the vascular walls [57].

**7) *Lrp8* (Low-Density Lipoprotein Receptor-Related Protein 8)**

**ABA review:** There were moderate levels of expression throughout the cerebral cortex, cortical subplate and brainstem. Virtually no staining was found in the striatum and pallidum. High levels of staining could be seen in cells lining the lateral ventricle, third ventricle and the lateral recess. In the thalamus, moderate-to-high level of expression was present in the medial habenula. In the hippocampal formation there was moderate-to-high labeling in the granular layer of the dentate gyrus and in the pyramidal layer CA3 field. Moderate levels of staining could also be found in the CA1 and CA2 fields. In addition, there was very high *Lrp8* expression in the cerebellar Purkinje cell layer. Finally, moderate punctate labeling was present in the underlying granular cell layer.


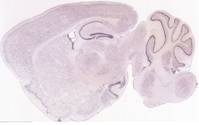

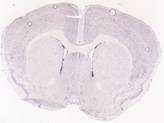


Sagittal section Coronal section

**GENSAT:** No information available.

**Literature:** Also referred to as *ApoER2*. *In situ* hybridization studies of adult rat brain showed that the apoER2 transcripts were detectable most intensely in the cerebellar cortex, choroid plexus, ependyma, hippocampus, olfactory bulb and, to a much lesser extent, in the cerebral cortex [58]. In the cerebellar cortex, the receptor transcripts were densely deposited in Purkinje cell somata. Four exons of the *APOER2* mRNAs are alternately spliced in both human fetal and adult brain tissue [59]. No differences are seen in the pattern of apolipoprotein E receptor 2 splicing between control and Alzheimer brains. Immunohistochemistry of mouse brain showed *Apoer2* is expressed in neurons throughout the brain, with strong expression in pyramidal neurons of the hippocampus, granule cells of the dentate gyrus, cortical neurons and Purkinje cells of the cerebellum. Using human riboprobes for *in situ* hybridization studies on rhesus monkey brain, it was found that in vessels, the endothelium expressed all three splice variants of *ApoER* [60]. In the cerebellum Purkinje cells, the large neurons located at the border of the granular and the molecular layer expressed high levels, whereas the basket cells and the granule cells showed low levels of two *ApoER2* mRNA variants. Furthermore, the oligodendrocytes in the white matter expressed low levels of two splice variants of the *ApoER2* mRNA.

**8) *Ager/Rage* (Advanced Glycosylation End Product-Specific Receptor)**

**ABA review:** Sparse low-to-moderate expression of *Ager* could be seen throughout the brain. In some slides there was moderate expression in the olfactory nerve layer of the main olfactory bulb. In addition, there was also high but inconsistent expression in the granular cell layer of the cerebellum.


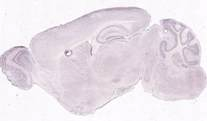


Sagittal section

**GENSAT:** No information available.

**Literature:** Is also referred to as *Mok*. Rage mediates binding of Alzheimer Disease (AD) amyloid-beta (1-40) peptide (sAbeta1-40) at the apical side of human BBB. In addition, Rage is involved in sAβ1-40 transcytosis [61]. Systemic amyloid beta (Aβ) infusion and studies in genetically manipulated mice have shown that Aβ interaction with RAGE-bearing cells in the vessel wall results in transport of Aβ across the blood-brain barrier [62]. Inhibition of the Rage-ligand interaction suppressed the accumulation of Aβ in brain parenchyma in a mouse transgenic model. These findings suggested that vascular Rage is a target for inhibiting pathogenic consequences of Aβ -vascular interactions, including development of cerebral amyloidosis. In human hippocampi there is robust *RAGE* immunoreactivity in neurons, but barely detectable staining in the microvascular [63]. In Alzheimer disease (AD) cases, neuronal *RAGE* immunoreactivity is significantly decreased. However, AD cases display the strongly positive microvascular *RAGE* immunoreactivity. Western blot analysis shows a much higher concentration of *RAGE* protein in AD hippocampi as compared with controls.

**9) *Slc28a2* (Solute Carrier Family 28 (Sodium-Coupled Nucleoside Transporter), Member 2)**

**ABA review:** Moderate-to-high expression of *Slc28a2* was present in the choroid plexus within the lateral ventricle and the lateral recess. Low-to-moderate staining could be seen in pallidum, thalamus, and throughout the cerebral cortex. In the main olfactory bulb, moderate expression was observed in the mitral, glomerular and granular layers. In the hippocampal formation there was moderate labeling in the granular layer of the dentate gyrus and in the pyramidal layer of the CA1, CA2 and CA3 fields. Moderate staining was present throughout the brainstem. However, a greater density of expression could be seen in the motor nucleus of trigeminal and the facial motor nucleus of the pons and medulla respectively.


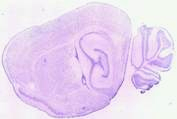


Sagittal section

**GENSAT:** No information available.

**Literature:** Also called *Cnt2* or *Spnt1*. Adenosine transport into brain is regulated by the activity of the adenosine transporter located at the brain capillary endothelial wall, which forms the blood-brain barrier *in vivo* [64]. The pattern of sodium dependency and NBTI inhibitor insensitivity of the cloned rat *Cnt2* were identical to patterns of adenosine transport across the BBB *in vivo.*  *In situ* hybridization on rat brain revealed that *Cnt2* mRNA was most prevalent in the amygdala, the hippocampus, specific neocortical regions and the cerebellum; most of the cells labeled were neurons [65]. Total sleep deprivation dramatically diminished the amounts of *Cnt2* mRNA.

**10) *Slc2a1* (Solute Carrier Family 2 (Facilitated Glucose Transporter), Member 1)**

**ABA review:** Moderate-to-high staining for *Slc2a1* was seen in the cells lining the lateral ventricle, third and fourth ventricles, cerebral aqueduct and the lateral recess. Moderate punctate staining was present throughout the brain with a slight enrichment of in the main olfactory bulb. At higher magnification it was noted that *Slc2a1* expression was in blood vessel and in the ependymal cells lining the lateral ventricle.


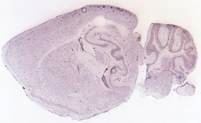

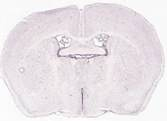

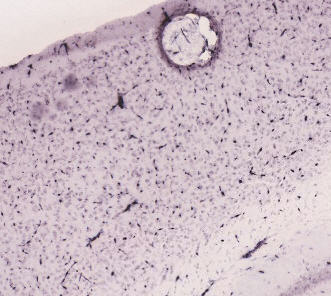


Sagittal section Sagittal (zoomed) Coronal section

**GENSAT:** No information available.

**Literature:** Also called *Glut1*. Immunogold electron microscopy localized *GLUT1* to the human brain capillary endothelia, with < 0.25% of the particles beyond the capillary profile [66]. Erythrocyte membranes were also highly immunoreactive, whereas macrophage membranes were *GLUT1*-negative. *GLUT1* glucose transporter was up-regulated in seizures, and this elevated transporter activity was characterized by increased *GLUT1* transporters, particularly on the luminal capillary membranes. Human *GLUT1* is present both in endothelium of the blood-brain barrier and in astrocytes surrounding gray matter blood vessels and synapses [67]. Furthermore, the form present in astrocytes has a lower molecular weight than the form found in cerebral endothelium.

**11) *Slc6a12* (Solute Carrier Family 6 (Neurotransmitter Transporter, Betaine/GABA), Member 12)**

**ABA review:** Images are not available for *Slc6a12*.

**GENSAT:** In the adult mouse, moderate-to-strong expression is found in 11 brain regions: amygdala, basal forebrain, cerebellum, dorsal horn, entorhinal cortex, hypothalamus, leptomeninges, medulla, olfactory bulb, pons, and thalamus. No detailed brain expression literature found. The BAC data is reproducible and consistent with the available literature. Confirmed expression sites are observed in pia, blood vessels and choroid plexus. The BAC produces expression in granule cells and cerebellar Golgi cells; expression in hippocampus and dorsal brainstem needs to be confirmed by *in situ*.

**Literature:** Also referred to as *Gat2* or *Bgt1*. In the mouse brain, *in situ* hybridization detected *GAT2* mRNA only in proliferating and migrating cerebellar granule cells and to some degree in the leptomeninges [68]. Expression was almost entirely restricted to the pia-arachnoid. RT-PCR analysis showed that *Gat2*/*Bgt-1* mRNA was expressed in mouse brain capillary endothelial cells, whereas Western blot analysis showed that these cells and mouse brain capillaries express *Gat2*/*Bgt-1* protein [69]. Moreover, confocal immunofluorescent microscopy of dual-labeled mouse brain sections demonstrated the colocalization of *Gat2*/*Bgt-1* and P-glycoprotein, a BBB-specific marker, on brain capillaries labeled with anti- *Gat2*/*Bgt-1* antibody and anti-P-glycoprotein antibody, respectively. These results suggested *Gat2*/*Bgt-1* was expressed at the BBB and was involved in GABA transport across the BBB.

**12) *Slc7a5* (Solute carrier family 7)**

**ABA review:** Moderate expression was present in the lateral ventricle in both the cells lining the ventricle and in the choroid plexus. There was a higher density of expression in the main olfactory bulb, cerebellum and the brainstem. In particular, there was moderate-to-high labeling in the pontine central gray. In some slides, there was high expression in the paraventricular hypothalamic nucleus. In the hippocampal formation there was moderate-to-strong labeling of the CA3 pyramidal layer. *Slc7a5* staining was very much reduced in the striatum and pallidum. In an independent review, it was noted that *Slc7a5* was highly and specifically expressed in endothelial cells and blood vessels of the brain.


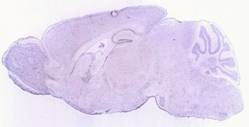

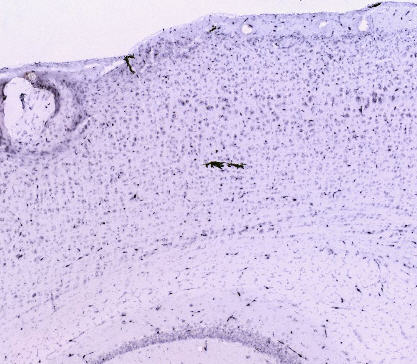


Sagittal section Sagittal – zoomed

**GENSAT:** No information available.

**Literature:** This gene is also referred to as *Lat1*. *Lat1* was shown to be expressed in the brain capillary endothelial cells in rats [70]. Both *Lat1* and *4F2hc* immunoreactivity were detected in a double line appearance surrounding endothelial cell nuclei, suggesting both proteins are present in the luminal and abluminal membranes. Northern hybridization and immunoblotting revealed that both *4F2hc* and *Lat1* were expressed and formed a heterodimer in mouse brain capillary endothelial cell line as an *in vitro* BBB model [71]. *Lat1* was predominantly expressed in the microvessels of the central nervous system, and the *4F2hc/LAT1* complex participates in L-DOPA transport across the BBB.

**13) *Slco1c1* (Solute Carrier Organic Anion Transporter Family, Member 1C1)**

**ABA review:** Moderate-to-high expression was observed in the lateral ventricle and the lateral recess. However, there was also expression in the choroid plexus. Moderate-to- high levels to staining could be seen in the rostral migratory stream. Low-to-moderate punctate staining was found throughout the brain. However, the density of expression was higher throughout the main olfactory bulb. Similarly, in the cerebellum there was moderate but significant expression in the Purkinje and granular cell layers. Additionally, there was also significant expression in the brainstem. In particular, there was moderate, but very dense expression, in the superior olivary complex of the pons and in the interpolar and oral segments of the spinal nucleus of the trigeminal of the medulla.


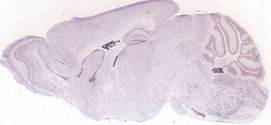

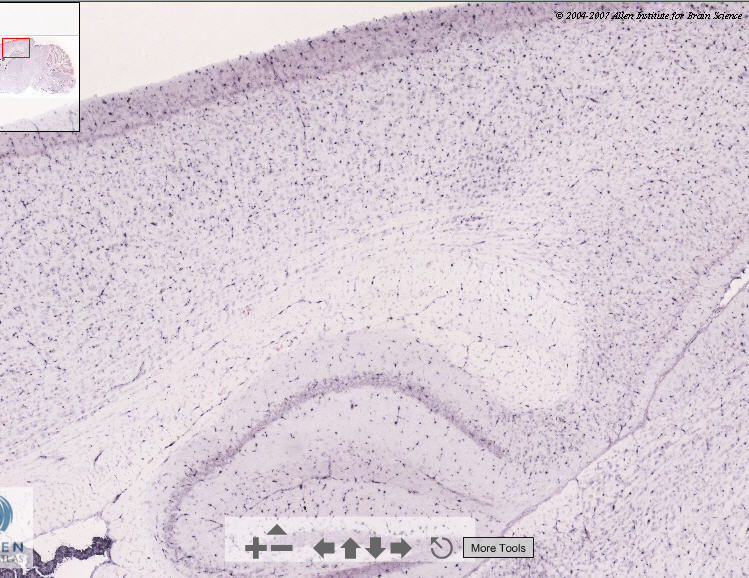


Sagittal section Sagittal (zoomed)

**GENSAT:** The EGFP transgene is primarily expressed in blood vessels.

**Literature:** This gene is variousl called *Bsat1*, *Oatp14*, *OatpF* and *Slc21a14.* A 3.3-kb *OATP-F* mRNA is present in numerous human brain regions with the exceptions of pons and cerebellum [72]. This wide intracerebral distribution of *OATP-F* is indicative of expression in the BBB. Northern blot analyses revealed predominant expression of *Oatp14* in rat the brain, and Western blot indicated expression in the brain capillary and choroid plexus [73]. *Oatp14* is expressed in the border of the brain capillary endothelial cells. *Oatp14* may play a role in transporting thyroid hormone T4 from the circulating blood to the brain.

**Subanatomical region: Brainstem, pons and medulla, Therapeutic Interest: Pain**

The brainstem (BS) comprised of the midbrain, medulla oblongata, and the pons is located caudal to the thalamus and hypothalamus, ventral to the cerebellum, and rostral to the spinal cord. BS plays an essential role in relaying information between the peripheral nervous system and spinal cord to the upper segments of the brain. BS contains the majority of cranial nerves that contain either sensory fibres, motor fibres, or both sensory and motor fibres. In addition, cranial nerves fibres also have a parasympathetic component. BS is involved in a number of integrative functions including arousal, alertness, breathing, blood pressure, heart rate and intestinal motility [74]. BS dysfunction may result severe disturbances of proprioception, vision, audition, vocalization, muscular weakness, and headaches [74].

**1) *Slc6a5* (Solute Carrier Family 6 (Neurotransmitter Transporter, Glycine), Member 5)**

**ABA review:** Strong expression of *Slc6a5* was found throughout the pons and medulla. In addition, there was scattered expression throughout the cerebellar granular layer. Interestingly, in coronal sections there was high and specific expression in the periaqueductal gray. In an independent review of the ABA images, it was noted that there was also labeling of cells in the spinal cord.


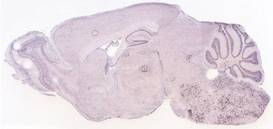

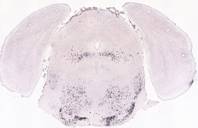


Sagittal section Coronal section

**GENSAT:** In the adult mouse strong to moderate expression is found in 4 brain regions: cerebellum, medulla, midbrain, and pons. The BAC data is reproducible and consistent with the literature. High levels of expression are mostly confined to the brainstem and spinal cord. The gene is also expressed in cerebellum to a lesser extent. Scattered cells in the superior colliculus are labeled, which had not been previously reported. In adult BAC mice, EGFP expression is greatly reduced.

**Literature:** The gene is also referred to as *Glyt2*. Northern hybridization analyses performed on rat brain tissue demonstrated that *Glyt2* mRNA was specifically localized in spinal cord, brain stem, and to a lesser extent in the cerebellum [75].

**2) *Glra1* (Glycine Receptor Alpha-1 Chain Precursor)**

**ABA review:** Moderate-to-high levels of *Glra1* expression were observed throughout the pons and medulla in saggital sections. In addition, there was significant scattered expression throughout the midbrain. In coronal section there was significant expression throughout the medulla, pons and midbrain. Several consecutive slides indicated highest expression in the sensory-related region of the pons.


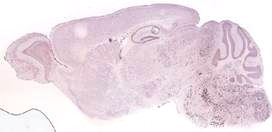

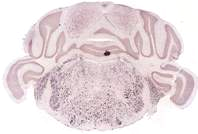


Sagittal section Coronal section

**GENSAT:** No information available.

**Literature:** The glycine receptor alpha 1-subunit is highly abundant in the adult mammalian spinal cord and brainstem [76].

**3) *Pogz* (Pogo Transposable Element with ZNF Domain)**

**ABA review:** We observed high expression throughout the thalamus, globus pallidus, pons and medulla (especially in the lateral reticular nucleus and external cuneate nucleus). In addition, significant expression was also seen throughout the midbrain. In an independent review of the ABA images, it was noted that the Pogz expression was very similar to the *Spp1* gene.


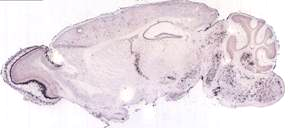

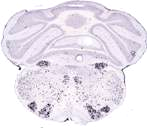


Sagittal section Coronal section

**GENSAT:** No information available.

**Literature:** No adult brain expression studies have been reported.

**4) *Anxa4* (Annexin A4)**

**ABA review:** *Anxa4* was expressed in the pontine gray and tegmental reticular nucleus of the pons. Within the medulla, *Anxa4* staining was enriched in the intermediate reticular nucleus and lateral recticular nucleus and external cuneate nucleus


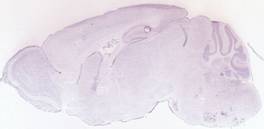

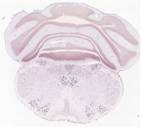


Sagittal section Coronal section

**GENSAT:** No information available.

**Literature:** In the rat annexin IV is primarily expressed dorsal root ganglia and the spinal chord by glial cells and at lower levels in neurons [77].

**5) *Spp1* (Secreted Phosphoprotein 1)**

**ABA review:** *Spp1* was found to be highly expressed throughout the pons and medulla especially in the lateral reticular nucleus and external cuneate nucleus. There was also high expression in the mitral layer of the main olfactory bulb and the reticular layer of the thalamus. Finally, there was moderate expression throughout the midbrain especially in the inferior colliculus and anterior pretectal nucleus.


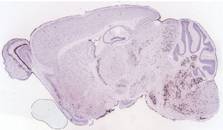

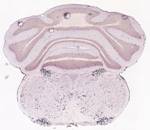


Sagittal section Coronal section

**GENSAT:** In the adult mouse strong to moderate expression is found in 3 brain regions: cochlear nucleus, olfactory nerve layer and pons. Four lines of BAC transgenic mice are identical at P7. The BAC data is consistent with the literature and the *in situ* hybridization data. Confirmed expression sites are observed in olfactory bulb, the lateral ventricles, cerebellum, medulla and spinal cord. The *in situ* data also shows Spp1 expression in particular nuclei in the ventral brainstem at P7, which are absent in the BAC data.

**Literature:** The gene is also referred to as *Opn*. In adult rat brain, using *in situ* hybridization, it was found that *Opn* mRNA was restricted to likely neurons in the olfactory bulb and the brain stem. Northern blot analysis showed a confined expression only in the brain stem with higher level in the pons and the medulla than in the midbrain. In the brain stem, it was found in functionally diverse areas including motor-related areas, sensory system and reticular formation [78]. Expression of *Opn* mRNA expression is present in the developing rat brainstem and cerebellum [79].

**6) *Esr1* (Estrogen Receptor 1 Alpha)**

**ABA review:** Very faint expression of *Esr1* was observed in the medial and posterior amygdalar nucleus, and in the periaqueductal gray.


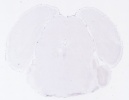

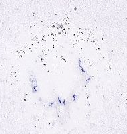


Coronal section Coronal section (zoomed)

**GENSAT:** No information available.

**Literature:**  *Esr1* is localized to the ventromedial hypothalamic nucleus and subfornical organ. Perikarya in other brain regions, including the bed nucleus of the stria terminalis, medial and cortical amygdaloid nuclei, preoptic area, lateral habenula, periaqueductal gray, parabrachial nucleus, locus ceruleus, nucleus of the solitary tract, spinal trigeminal nucleus and superficial laminae of the spinal cord [80]. *In situ* hybridization studies in mouse brain revealed that *Esr1* was expressed in the dorsal raphe nucleus and the periaqueductal grey [81].

**7) *Pou4f1* (POU Domain, Class 4, Transcription Factor 1)**

**ABA review:** High level expression of *Pou4f1* could be seen in the medulla at the inferior olivary complex, nucleus raphe pallidus, and nucleus raphe obscurus. Within the midbrain, there was low-to-moderate expression in the periaqueductal gray and the red nucleus.


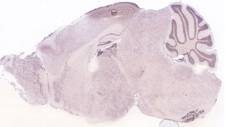

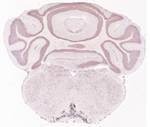


Sagittal section Coronal section

**GENSAT:** No information available.

**Literature:** The gene is also called *Brn3a.* *Brn3a* is expressed in terminally differentiating neurons in the sensory peripheral nervous system and in specific neurons of the midbrain, hindbrain and spinal cord. In transgenic mice, 11 kb of *Brn3a* upstream flanking sequence was sufficient to direct reporter gene expression to the sensory neurons expressing *Brn3a*, but not to *Brn3a* neurons in the central nervous system. In an E13.5 embryo expressing this transgene, ß-gal activity is evident in the trigeminal ganglion, vestibulocochlear ganglion, the IX/X ganglion complex, the dorsal root ganglia, and the central and peripheral axons of these groups of neurons. *Brn3a* negatively regulated its own expression *in vivo* [82]. Transgenic mice were developed where a tau::*lacZ* reporter replaced the entire *Brn3a* coding sequence. Expression of the tau::*lacZ* transgene replicated all known sites of *Brn3a* expression in an E13.5 *Brn3a*tau::*lacZ* embryo. Expression of the tau::*lacZ* transgene was localized in the adult brain to the caudal thalamus and the superior colliculus [83].

**8) *Slc4a2* (Solute Carrier Family 4 (Anion Exchanger), Member 2)**

**ABA review:** *Slc4a2* was expressed at low-to-moderate levels in the pons and medulla. In coronal sections, there was high and specific expression in the periaqueductal gray. Expression could also be observed in the red nucleus of the midbrain. In addition, *Slc4a2* also labeled cells in the cerebellar interposed nucleus.


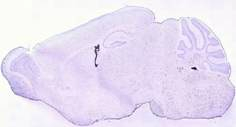

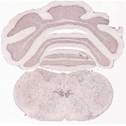


Sagittal section Coronal section

**GENSAT:** In the adult mouse strong to moderate expression is found in the choroid plexus. No brain expression literature was found. The BAC data is reproducible and is consistent with the *in situ* hybridization data from ABA. The gene is strongly expressed in choroid plexus and ependymal cells lining the ventricles. However, the BAC shows much less expression in brainstem at adult. In adult spinal cord, the expression is not restricted to the dorsal horn as seen in P7 and E15-5 BAC mice.

**Literature:** The gene is also referred to *Ae2*. No adult brain expression studies have been reported.

**9) *Stac* (SRC homology 3 and Cysteine-Rich Domain Protein)**

**ABA review:** *Stac* was highly expressed in the cerebellar Purkinje cells and within the medulla it was present in the inferior olivary complex. There was also relatively low level expression throughout the medulla and all layers of the cerebral cortex.

Sagittal section Coronal section

**GENSAT:** No information available.

**Literature:** Northern blotting and *in situ* hybridization analyses demonstrated that 2.7 kb of *Stac* mRNA was expressed predominantly in brain and neurons, especially in hippocampus, cerebellum and inferior olive [84].

**Subanatomical region: cerebellum Granule cells, Therapeutic interest:** **Medulloblastoma, Ataxia, Cerebellar hypoplasia**

Like the cerebrum, cerebellum also contains similar gray and white matter divisions. The cerebellar cortex is divided into three layers namely, the molecular, Purkinje, and granular layers. The cerebellum plays an important role in the integration of sensory perception and motor output, particularly fine motor control and balance. Patients with cerebellar damage have problems with motor coordination and movement.

**1*. Gabra6* (Gamma-Aminobutyric acid (GABA) A Receptor, Alpha 6)**

**ABA review:** Moderate-to-high expression could be seen in the cerebellar Purkinje cell and the underlying granular cell layer. Virtually no expression could be seen in other regions of the brain. An independent review indicated significant expression in the cerebellar granule cells. Some more limited populations of other neurons, such as the hippocampal pyramidal cells were also stained for *Gabra6*.

Sagittal section Coronal section Coronal zoomed

**GENSAT:** Moderate-to-strong expression is present in the cerebellum and cochlear nucleus. The BAC data matches the literature and BGEM in situ data. The database line is a low copy number line. Overall level of expression is weak in BAC transgenic mice.

**Literature:** Microarray analysis revealed *Gabra6* is regionally enriched in the adult mouse cerebellum [11].

*Gabra6* mRNA in the cerebellum increases dramatically during the second postnatal week, reaching maximal levels by postnatal day 21 [85]. No further increase in alpha6 mRNA expression in the adult cortex was observed. Expression was only detected in the internal granule cell layer and not in either the external granule cell layer or in migrating granule cells. In a transgenic mouse line that expresses CRE recombinase under the *Gabra6* promoter, recombination of an R26R reporter allele occurred post-natally in granule cells of the cerebellum and dorsal cochlear nucleus, as well as in a subset of pre-cerebellar nuclei in the brainstem [86]. In α6 -/- mice, the cerebellum expresses only half of the number of GABAA receptors present in wild-type animals [87]. Because these animals have no gross motor deficits, synaptic integration in granule cells is apparently maintained by α1-subunit-containing receptors with an altered overall subunit composition, and/or by changes in the expression of other ligand and voltage gated channels.

**2. *Cbln3* (Cerebellin 3 precursor)**

**ABA review:** There was strong and specific expression in the cerebellar granule cells with no expression elsewhere.

Sagittal section Coronal section

**GENSAT:** Moderate-to-strong expression is present in the cerebellum, medulla and pons of the adult mouse, and BAC data matches BGEM in situ data. The EGFP reporter gene is expressed in neurons in the cerebellar internal granule cell layer. Cerebellar expression is detected as early as E15.5 in BAC mice. In situ data shows no expression at E15.5. This was thought to be due to the difference in determining the gestation date.

**Literature:**  *Cbln3* mRNA was selective to cerebellar granule cells throughout development, and its onset was as late as postnatal day 7-10 [88]. In another study using situ hybridization it was shown that there is robust expression in the cerebellum [89]. In the adult, the internal granule cell layer was the predominant site of *Cbln3* expression. Grain density over Purkinje neurons was indistinguishable from background, suggesting that they did not express *Cbln3*.

**Subanatomical region: cerebellum purkinje cells, Therapeutic interest: Spinocerebellar Ataxia, Autism, Plasticity**

(See description of Cerebellum under Subanatomical region: cerebellum Granule cells)

**1*. Pcp2* (Purkinje Cell Protein 2)**

**ABA review:** Very high expression of *Pcp2* was present in the cerebellar Purkinje cell layer. Virtually no expression could be seen in other regions of the brain.

An independent review indicated very strong and specific expression in the Purkinje cell layer of the cerebellum.

Sagittal section Coronal section Coronal (zoomed)

**GENSAT**: There are three regions of moderate-to-strong expression in the adult: cerebellum, medulla and pons. BAC data is reproducible and matches the literature and BGEM in situ data.

**Literature:** Microarray analysis revealed *Pcp2* is regionally enriched in the adult mouse cerebellum [11]. In a mouse BAC-*Pcp2*-IRES-Cre transgenic line, Cre recombinase activity is detected exclusively in cerebellar Purkinje cells and retina bipolar neurons. Cre recombinase activity was initially detected at P6 before increasing to a plateau around P15 [90]. Expression of *Pcp2*, like development of the Purkinje cell, is only transiently responsive to thyroid hormone stimulation [91]. *Pcp2* expression is stimulated by thyroid hormone during the 2nd and 3rd weeks of postnatal life in the mouse and rat. Conversely, the gene is refractory to thyroid hormone stimulation in the fetus and early neonate. This is because COUP-TF expression in the immature Purkinje cell renders the *Pcp2* gene refractory to thyroid hormone stimulation during the early period of Purkinje cell development. The presence of elements lying within the 3.1 kb upstream region of the *Pcp2* gene is thought to confine its expression to the cerebellar Purkinje cells [92].

**2. *Hbegf* (Heparin-Binding EGF-Like Growth Factor)**

**ABA review:**  One interpretation of the ABA images was that there was specific expression in the cerebellar Purkinje cell layer with some sporadic cells labeled as well. In an independent review, it was confirmed that there was moderate expression solely in the Purkinje cell layer of the cerebellum.

Sagittal section Sagittal (zoomed)

**GENSAT:** Moderate-to-strong expression in the adult was seen in the cerebellum and the olfactory bulb. The BAC data is correct but incomplete. Confirmed expression sites include cerebellum and brainstem.

**Literature:** In the adult rat brain, the strongest in situ hybridization signals is seen in the cerebellar cortex, particularly in the granular cell layer and Purkinje cells [93]. Additionally, expression is detected in cerebrum, olfactory bulb, olfactory tubercle, entorhinal cortex, dentate gyrus and brainstem. There was moderate expression in the neocortex, thalamus, subthalamic area and hypothalamus, and weak expression in the caudate putamen.

**3*. Icmt* (Isoprenyl Cysteine Carboxyl Methyltransferase)**

**ABA review:** There was very strong expression in all Purkinje cells and very little else in other brain regions, except for scattered, lightly labeled cells. An independent review indicated very high and specific expression in the cerebellar Purkinje cell layer. Coronal sections indicated low level expression throughout the brain. Some slides inconsistently suggested high density of expression in the olfactory bulb and in the thalamus.

Sagittal section Sagittal section (zoomed)

**GENSAT:** No information available

**Literature:** This gene is also called *Pccmt*. In adult mice and the human brain, *PCCMT* mRNA was highly enriched in the cerebellum, with low levels of expression in other brain regions. Immunohistochemical analysis using an antibody against human Pccmt protein found abundant expression in Purkinje cells and pontine neurons. Gene expression is reduced in spinocerebellar ataxia type 1 (SCA1) transgenic mice until 6 weeks following birth [94]. All knockout embryos (*Icmt*-/-) die by mid-gestation [95].

**4. *Atp2a3* (ATPase, Ca^+2^ Transporting, Ubiquitous)**

**ABA review:** There was very specific, high level expression limited to the Purkinje cells. However, two non-consecutive slides displayed moderate labeling throughout the brain stem. An independent review indicated strong expression in Purkinje cells. Expression was also present in cells of choroid plexus and cells of the substantia nigra pars compacta.

Sagittal section Sagittal (zoomed)

**GENSAT:** No information available

**Literature:** This gene is also referred to as *Serca3*. In situ hybridization revealed *Serca3* transcripts in cells of the intestinal crypt, the thymic cortex, and Purkinje cells in the rat cerebellum [96]. In another study it was shown that *Serca3* is specifically expressed in rat Purkinje neurons based on immunocytochemistry, in situ hybridization and single-cell RT-PCR. Immunocytochemistry revealed expression of Serca3 in the cell body and in the dentritic processes of the Purkinje neurons of the rat brain [97].

**5. *Casq2* (Calsequestrin 2; Cardiac muscle)**

**ABA review:** There was strong and specific in all Purkinje cells – filling the cytoplasm robustly. A class of small cells in olfactory bulb and tubercle, striatum, and cortex are also labeled but this is a small population of cells. An independent review also indicated strong and specific expression in the Purkinje cell layer. Low-to-moderate expression was observed throughout the medulla, caudoputamen, ventro-postero-medial nucleus of the thalamus and the olfactory nerve layer of main olfactory bulb.

Sagittal section Sagittal (zoomed)

**GENSAT:** No information available

**Literature:** In the chicken brain, the gene for *CASQ2* was found to be selectively expressed in Purkinje neurons, as judged by Northern blotting, in situ hybridization and immunocytochemistry [98]. Northern blot did not detect any transcript in mouse brain [99].

**6. *Gdf10* (Growth Differentiation Factor 10)**

**ABA review:** There was very nice expression in cerebellar Purkinje cells, as well as in the olfactory bulb mitral cells, and polymorph cells in the dentate hilus. There was low level in situ staining in general and it is not clear if this is real or just background. An independent review indicated high expression in the cerebellar Purkinje cells. There also appeared to be low-to-moderate expression in the mitral layer of the main olfactory bulb, and sparse punctuate labeling could be seen throughout the brain.

Sagittal section Sagittal (zoomed)

**GENSAT:** Images were only available for developmental stage P7. The BAC produces expression predominantly in Bergmann glial cells in cerebellum, which is confirmed by ABA in situ data. Because Purkinje cells, Bergmann glia and cells surrounding them are all located at the junction of molecular layer and internal granule layer, it is sometimes hard to determine the cell types from in situ data. Besides cerebellum, the BAC is expressed in main olfactory bulb, piriform cortex, dentate gyrus (scattered cells) and cortex (very weak expression). These sites are all confirmed by the in situ data. There are scattered astrocytes labeled across the central nervous system in the BAC data.

**Literature:** The human *GDF10* gene is also called *BMP3b*. In mice, *Gdf10* mRNA is primarily localized to cells in the Purkinje cell layer of the cerebellum, and *Gdf10*-expressing cells are unlikely to correspond to Bergmann glia [100]. Microarray analysis revealed *Gdf10* is regionally enriched in the adult mouse cerebellum [11]. In the adult rat brain one study reported that *Gdf10* is only expressed in the granule cells of the dentate gyrus and in the pyramidal cells of the hippocampal CA3 layer [101]. Another study reported that *Gdf10/Bmp3b* is expressed almost exclusively in the cerebellum (region not indicated) [102].

**7*. Grid2* (Glutamate Receptor, Ionotropic, Delta 2)**

**ABA review:** Very high expression could be found in the cerebellar Purkinje cell layer. Sparse, scattered, and punctate staining was present throughout the brain. In particular, moderate, punctate staining was seen in layers 2/3, 4, 5, 6 of the cerebral cortex.

Sagittal section Coronal section

**GENSAT:** No information available

**Literature:** RNA blot and in situ hybridization show that the *Grid2* subunit mRNA is localized selectively in mouse cerebellar Purkinje cells [103]. Microarray analysis revealed *Grid2* is regionally enriched in the adult mouse cerebellum [11]. Lurcher (Lc) is a gain-of-function mutation in *Grid2* that results in the cell-autonomous death of cerebellar Purkinje cells in heterozygous lurcher (+/Lc) mice [104].

**8. *Hes3* (Hairy and Enhancer of Split 3)**

**ABA review:** There was moderate-to-high expression in the cerebellar Purkinje cells. In addition there appeared to be low-to-moderate throughout the cerebral cortex but especially high in layer 2 of cerebral cortex in piriform area. Higher density of labeling was also seen in layer 2/3 and 5 of the cortex. In the hippocampal formation, significant levels of expression were observed in the pyramidal layer of the CA1. Scattered expression was also seen throughout the thalamus, brainstem and cerebellar interposed nucleus, and in the main olfactory bulb. An independent review indicated Purkinje cells were labeled but not very robustly. Cells of piriform cortex were labeled more intensely. Superficial layer V pyramidal neurons are positive. In addition other large cell populations appeared to have low-level positivity.

Sagittal section Sagittal (zoomed)

**GENSAT:** No information available

**Literature:** In the rat brain, *Hes3* is a cerebellar Purkinje cell-specific transcription factor [105]. For the mouse *Hes3* gene, two transcriptional initiation sites in exon 1a and exon 1b used [106]. Exon 1a is transcribed only in postnatal cerebellum, whereas exon 1b is only in embryos.

**9. *Lhx1* (LIM Homeobox Protein 1)**

**ABA review:** Expression is very specific to Purkinje cells and nowhere. An independent review indicated moderate but very specific expression in the Purkinje cells. In some coronal sections high expression was observed in the medial preoptic nucleus.

Sagittal section Sagittal (zoomed)

**GENSAT**: The BAC data at E15.5 and P7 is consistent with in situ data. There are 14 regions of moderate-to- strong expression in the adult: amygdala, anterior olfactory nucleus, cerebellum, cerebral cortex, entorhinal cortex, fornix, hippocampus, hypothalamus, medulla, olfactory bulb, piriform cortex, septum, subicular cortex, and ventral striatum.

**Literature:** This gene is also called *Lim1* in the literature. In situ hybridization revealed that only the Purkinje cell layer of the adult rat cerebellum strongly expressed *Lim1* [107].

**10*. Ptprm* (Protein Tyrosine Phosphatase, Receptor Type M)**

**ABA review:** It is not clear that the signal in cerebellum is Purkinje cells; it could be very hot Bergmann glia. This would fit with the spotty in situ hybridization positivity that looks to be in glia. An independent review indicated moderate expression exclusively in the cerebellar Purkinje cells.

Sagittal section Sagittal (zoomed)

**GENSAT:** No information available

**Literature:** This gene is also called *Rptpm* or *RPTPmu*. In the adult mouse brain, RPTPmu is expressed at high levels in the Purkinje cells, and at much lower levels in Golgi, stellate and basket cells [108]. *RPTPmu*::*lacZ* knock-in mice have been generated that express the *lacZ* reporter gene under the control of the *RPTPmu* promoter [109]. Analysis of beta-galactosidase activity revealed *RPTPmu* expression in Purkinje cells and other neurons in the adult mouse brain.

**11. *A930006D11Rik* (Hypothetical Protein)**

**ABA review:** Very high expression was observed in the cerebellar Purkinje cell layer. In the underlying granular layer there was also sparse, moderate-to-high level punctate staining. Moderate level expression could be seen in the lateral ventricle. Similar levels of labeling were present in the glomerular layer of the main olfactory bulb.

An independent review indicated very nice, specific and intense Purkinje cell label as well as interneurons in the internal granule cell layer (Golgi type II cells, likely).

Sagittal section Coronal section

**GENSAT:** No information available

**Literature:** No relevant brain expression studies have been reported.

**Subanatomical region: cerebral Cortex, Therapeutic interest: Alzheimer disease, plasticity, amyotrophic lateral sclerosis**

The mammalian cerebral cortex is a complex, highly organized, six-layered structure that contains hundreds of different neuronal cell types and glia. It is the region of the brain responsible for cognitive function, sensory perception and consciousness, and as such it has undergone pronounced expansion and development during evolution. Differences in intellectual abilities of humans and other mammals are due to the extent of development of the cerebral cortex. Most of the actual information processing within the brain takes place in the cerebral cortex. Neurodegenerative diseases that affect cortical and subcortical areas of the brain include Alzheimer disease, amyotrophic lateral sclerosis, progressive supranuclear palsy and Huntington disease.

**1. *B3galt2* (UDP-Gal:betaGlcNAc beta 1,3-Galactosyltransferase, Polypeptide 2)**

**ABA review:** Moderate but specific expression was observed throughout layers 5 and 6 of the cerebral cortex. Similar levels of expression were present in layer 2 of the piriform and cortical amygdalar areas of the cortex. High expression could be seen in the dorsal and ventral segments of the taenia tecta. The glomerular, mitral and granular cell layers of the main olfactory bulb displayed low-to-moderate expression. In the hippocampal formation, there was high expression in the in CA1, CA2, CA3 fields and in the dentate gyrus. In the cortical subplate there was high expression in the layer 6b (isocortex), claustrum and the dorsal/ventral segments of the endopiriform nucleus. Lower levels of staining could also be seen scattered throughout the basolateral amygdalar nucleus. Within the retrohippocampal region, there was high expression in layers 5/6 of the entorhinal area and moderate expression throughout the subiculum. There was low level of labeling throughout the ventral group of the dorsal thalamus, brainstem and the cerebellar Purkinje cell layer. An independent review indicated that the deep cortex in all areas was labeled fairly nicely; the dentate gyrus, some hippocampal pyramidal cell, and cells in lateral thalamus also had the same intensity of label.

Sagittal section Coronal section

**GENSAT:** No information available.

**Literature:** Northern detected mRNA in the human and mouse brain [110, 111].

**2*. 3110035E14Rik* (Hypothetical Protein)**

**ABA review:** In the cerebral cortex there was moderate-to-high expression in layers 2, 3, 5, and 6; expression appeared to be highest in layers 5 and 6, and virtually no expression was observed in layer 4. A similar level of expression could be seen in the primary and secondary motor areas of layer 2. In the cortical subplate there was high staining in layer 6b and the anterior part of the basolateral amygdalar nucleus. Moderate expression was also present in the ventral and posterior segments of the basolateral amygdalar nucleus. There was very high labelling in the lateral, medial, and posteroventral segments of the anterior olfactory nucleus. In the striatum there was moderate-to-strong expression in the lateral septal complex, and olfactory tubercle. In the hippocampal formation there was moderate-to-high expression in the CA1 and CA3 fields’ pyramidal layer. In addition, there was high expression along the length of the stratum oriens, and some faint scattered expression was also observed inconsistently in the thalamus, hypothalamus and brainstem.

Sagittal section Coronal section

**GENSAT:** No information available

**Literature:** SAGE analysis revealed regional enrichment in the cingulate cortex of adult C57BL/6 mice [10].

**3. *Ccl27* (Chemokine (C-C motif) Ligand 27)**

**ABA review:** Moderate-to-high expression could be seen in layers 2 to 6 of the cerebral cortex. Little or no expression was observed in the olfactory and retrohippocampal areas. In the cortical subplate there was moderate expression in the subiculum. In the main olfactory bulb, there was sparse moderate expression in the olfactory nerve layer of main olfactory bulb. In an independent review, it was observed that expression was found in about 50% of cortical cells with less expression in layer 4.

Sagittal section

**GENSAT:** No information available

**Literature:** This gene is also called ESkine, CTACK or ILC. Murine ESkine is produced as two splice variants. Differential splicing arises as a result of alternative 5' exon usage; ESkine is highly expressed in the placenta while PESKY is mainly expressed in the testes and brain (region not indicated) [112]. Microarray analysis revealed *Ccl27* is regionally enriched in the adult mouse cerebral cortex [11].

**4. *Ctgf* (Connective Tissue Growth Factor)**

**ABA review:** There was strong expression along the corpus callosum, although by SAGE the gene is expected to be present in the amygdala basolateral complex.

An independent review of the ABA images also indicated very specific expression in the cells dorsal to the corpus callosum, and also in the olfactory bulb.

Sagittal section Coronal section

**GENSAT:** There is moderate-to-strong expression in the amygdala, caudate putamen, cerebral cortex, entorhinal cortex, globus pallidus, hippocampus, internal capsule, leptomeninges, olfactory bulb, piriform cortex, pons, and thalamus. The BAC data is consistent with the literature and in situ data from Allen Brain Altlas. In adult BAC transgenic mice, layer VIb neurons of the cortex are labeled; other confirmed expression sites include ependymal cells lining the lateral ventricles and blood vessels.

**Literature:** Astrocytic Ctgf immunoreactivity is detected in the cerebral cortex and white matter of the spinal cord [113]. A very strong and selective expression of *Ctgf* mRNA is found on the band of layer VII neurons throughout the adult rat cerebral cortex [114].

**5. *Emx1* (Empty Spiracles Homeobox 1)**

**ABA review:** This gene had no images available for reviewing on ABA

**GENSAT:** There is moderate expression in layers 1, 2, and 5 of the cerebral cortex. Slightly higher levels of expression were found in layer 1/2 of the piriform cortex. Moderate levels of labeling could also be seen in the mitral and glomerular layers of the main olfactory bulb, and along the length of the corpus callosum. In the hippocampal formation, there is moderate-to-strong expression in the dentate gyrus; however, some expression could also be seen in the CA1, CA2 and CA3 fields. Cells were also stained in the bed nucleus of the stria terminalis portion of the pallidum, and there is moderate-to-strong expression throughout the hypothalamus. The BAC data is consistent with the literature.

**Literature:** *Emx1* is a marker for pyramidal neurons of the cerebral cortex [115]. Specifically, it was found that, similar to pyramidal neurons, cells expressing *Emx1* are distributed in all rat cortical layers, except layer I. *LacZ* and *Cre* genes have been inserted directly into the exon 1 of the *Emx1* gene. The distribution of β-galactosidase activity in the transgenic mice was consistent with endogenous gene expression in the developing and adult cerebral cortex and hippocampus [116]. In another study, Cre recombinase gene was inserted into the PAC Emx1-locus clone and utilized to generate three transgenic mouse lines [117]. Dense beta-galactosidase staining, indicative of many cells with Cre-mediated recombination, was detected in the olfactory bulb, neocortex, piriform cortex, hippocampus, and amygdala in each of transgenic mice. In contrast, there were no LacZ+ cells in other regions of the brain.

**6. *Fhl2* (Four And A Half LIM Domains 2)**

**ABA review:** There was moderate-to-high labeling throughout the cerebral cortex. Expression appeared to somewhat higher in layer 1 of the anterior olfactory nucleus, the pyramidal layer of the piriform area, the islands of Calleja in the olfactory tubercule, and the dorsal/ventral segments of the taenia tecta. In the cortical subplate there was high expression in the claustrum, the dorsal endopiriform nucleus and the anterior part of the basomedial amygdalar nucleus. Within the hippocampal formation there was very high expression in CA1, CA2 and CA3 fields. Some moderate punctate expression was also present in the dentate gyrus. In the main olfactory bulb there was low-to-moderate expression in the glomerular, mitral, and granular cell layers.

Sagittal section Coronal section

**GENSAT**: No information available

**Literature:** *Fhl2* was identified in a microarray study to identify genes differentially expressed along the A-P axis of the developing cerebral cortex of E12.5 mouse; this was confirmed by in situ hybridization [118]. SAGE analysis revealed regional enrichment of *Fhl2* in the prelimbic cortex of adult C57BL/6 mice [10]. Mice deficient for the transcriptional cofactor Fhl2 exhibit a dramatic decrease of bone mass in both genders [119]. In human mesial temporal lobe epilepsies (MTLE), the *FHL2* gene is downregulated in the entorhinal cortex of MTLE patients, as compared with non-epileptic autopsy controls [120].

**7. *Klf10* (Kruppel-Like Factor 10)**

**ABA review:** No expression was observed in layer 4 of the cerebral cortex; otherwise, there was moderate expression throughout the remainder of the cortex. Labeling was higher in the visceral and gustatory areas and in the dorsal/ventral segments of the agranular insular area. In the main olfactory bulb there was moderate expression in the granular cell layer, anterior olfactory nucleus and the taenia tecta. In the hippocampal formation there was moderate-to-high expression in the in CA1, CA2 and CA3 fields. Somewhat lower levels of expression were also present in the dentate gyrus.

Sagittal section Coronal section

**GENSAT:** The BAC data grossly matches the in situ data from BGEM and Allen Brain Atlas

**Literature:** This gene is also called *Tieg1*, *mGIF* and *EGRalpha*. In the adult mouse brain, *mGIF* is abundantly expressed in hippocampus, cerebral cortex, cerebellum, and amygdala with lower amounts in striatum, nucleus accumbens, olfactory tubercle, thalamus, and substantia nigra [121].

**8. *Myl4* (Myosin, Light Polypeptide 4)**

**ABA review:** There was moderate-to-high expression throughout layers 3 to 6 of the cerebral cortex; staining appeared to be slightly higher in layer 4. In the cortical subplate, similar levels of labeling were also observed in the ventral part of the lateral amygdalar nucleus and endopiriform nucleus. Some low-to-moderate, punctate expression could also be seen in layer 2 of the piriform area and throughout the brainstem. However, in the medulla there was moderate expression in the dorsal motor nucleus of the vagus nerve and the hypoglossal nucleus.

Sagittal section Coronal section

**GENSAT:** No information available

**Literature:** This gene is also called *Mlc1a* (*MLC1emb* in human). No brain expression has been reported.

**9. *Rbp4* (Retinol Binding Protein 4, Plasma)**

**ABA review:** There was moderate expression throughout layers 2 to 6. However, density of staining appeared to be higher in layers 5 and 6. Within the cortical subplate, similar levels and density of labeling was present throughout the subiculum. There was moderate expression in the olfactory nerve layer of main olfactory bulb. There was also low-to-moderate staining throughout the caudodorsal and rostroventral portions of the lateral septal nucleus, nucleus accumbens, and caudoputamen. Within the pons there was moderate labeling of the locus coeruleus, and to some extent, the sublaterodorsal nucleus. In addition, in some coronal sections there appeared to be moderate punctate expression throughout the medulla. There was moderate staining in the cerebellar Purkinje cell layer.

Sagittal section Coronal section

**GENSAT:** No information available

**Literature:** There is a significant association between the polymorphic marker D10S583 (*RBP4*) and Alzheimer disease [122]. Mice lacking plasma RBP display impaired vision at the time of weaning but are otherwise phenotypically normal [123]. No brain expression has been reported.

**10. *Rtn4rl2* (Reticulon 4 Receptor-Like 2)**

**ABA review:**  There was low-to-moderate expression in layers 5 and 6a of the cerebral cortex. In the cortical subplate, moderate-to-high expression was present in the claustrum, the anterior part of the basolateral amygdalar nucleus, and to a lesser extent also in the dorsal segment of the endopiriform nucleus. In the hippocampal formation there was moderate-to-high expression in the CA1, CA2 and CA3 pyramidal cell layer. In addition, there was moderate expression in the mitral layer of the main olfactory bulb.

Sagittal section Coronal section

**GENSAT:** No information available

**Literature:** The gene is also called *Ngrh1* or *Ngrl3*. Northern blot analysis showed predominant expression of *NGRH1* mRNA in the human brain [124]. It is abundantly expressed throughout the cerebral cortex without obvious differences between the frontal, parietal, occipital and temporal lobe and the paracentral gyrus. Expression at a level comparable to the cortex is also found in the amygdala, the hippocampus and nucleus accumbens. In the rat brain, in situ hybridization detected expression in all cortical layers except layer 1, and in the hippocampus. In the adult mouse brain, the gene is highly expressed in the cerebral cortex (layers II–VI), hippocampal formation, amygdala, habenula, mitral cell layer of the olfactory bulb, and in anterior olfactory nucleus [125].

**11. *Stx1a* (Syntaxin 1A)**

**ABA review**: There was moderate-to-high expression in layers 2 to 4 of the cerebral cortex; low-to-moderate labeling was also observed in layers 5 and 6a. High density, moderate level expression was present in the medial, dorsal, lateral and posteroventral segments of the anterior olfactory nucleus. In addition, there was moderate labeling of cells in layer 1 of the olfactory tubercule. Low level staining was also present throughout the lateral septal nucleus. In the hippocampal formation there was moderate-to-high expression in the CA1, CA2 and CA3 pyramidal cell layer. Moderate levels can be seen in the medial portions of the thalamus including the central medial nucleus, nucleus of reunions and paraventricular nucleus. In the main olfactory bulb there was moderate expression in the mitral, glomerular, granular and outer plexiform layers.

Sagittal section Coronal section

**GENSAT:** The BAC data grossly matches literature and BGEM in situ data. There are 14 regions of strong to moderate expression in the adult: amygdala, cerebral cortex, entorhinal cortex, hippocampus, hypothalamus, inferior cerebellar peduncle, medulla, midbrain, olfactory bulb, pons, pyramidal tract, spinal cord dorsal horn, spinal cord ventral horn and ventral striatum.

**Literature:** This gene is also called *Hpc-1*. The *HPC-1*/syntaxin 1A (*STX1A*) gene is commonly deleted in Williams syndrome (WS). In the human brain, marked expression is observed in cerebellum and cerebral cortex. STX1A protein is distributed in the molecular layer of the cerebellar cortex, with no significant difference among frontal, temporal, and occipital poles of the human adult cortex in the two hemispheres [126]. Microarray analysis revealed *Stx1a* is regionally-enriched in the adult mouse and rat cerebral cortex [9, 11, 15]. SAGE analysis revealed regional enrichment in the somatosensory cortex of adult C57BL/6 mice [10]. In the rat brain, immunohistochemistry detected *Hpc-1* in the matrices of the cerebral cortex and hippocampus, the molecular layer, membranes of granular cell somas and glomeruli in the cerebellum [127]. HPC-1/syntaxin 1A, which has been identified as a presynaptic membrane protein, is believed to regulate synaptic exocytosis. The distribution of the protein, however, is not restricted to the synaptic terminal, but is also found on the axonal membrane. When the expression of *Hpc-1* was suppressed, neurite sprouting was enhanced in cultured neurons [128].

**12. *Tbr1* (T-box Brain Gene 1)**

**ABA review:** There was moderate expression in layers 6a-b in the cerebral cortex, as well as in layers 2 and 3 of the piriform cortex area. Some scattered, moderate labeling was also observed in the glomerular layer of the main olfactory bulb. In some slides, low-to-moderate staining could be seen in the granule cell layer of the dentate gyrus and the CA1, CA2 and CA3 pyramidal cell layer.

Sagittal section Coronal section

**GENSAT:** No information available

**Literature:** In normal human controls, *TBR1* labeling in the prefrontal cortex is diffuse and present in all layers, being higher in layer VI [129]. The finding that *TBR1* expression is increased in bipolar patients, but not in major depressed patients, suggests abnormalities of specific genes related to a major cortical cell type and its connectivity. Within the postnatal neocortex of the mouse, *Tbr1* expression is found at different levels in distinct layers [130]. *Tbr1* is robustly expressed in the superficial zones corresponding approximately to layers 1, 2, and 3. Between about Pl and P7, thalamic expression of *Tbr1* begins; weak thalamic expression continues to be present at P17 and in the adult. Microarray analysis revealed *Tbr1* is regionally enriched in the adult mouse cerebral cortex [11]. A mouse *Tbr1* promoter with downstream sequences was used to generate Gfp reporters that were incorporated into transgenic mice [131]. The *Tbr1*-driven transgene is expressed in cortical neurons expressing the native *Tbr1* gene in a temporal pattern that mimicked the endogenous gene's expression. The *Tbr1*-driven transgene, however, was selectively expressed by just a subset of Tbr1+ cortical cells namely neurons of the deep cortical plate and subplate. Transgene-positive neurons are generated early in corticogenesis and persisted into adulthood.

**13. *Vip* (Vasoactive Intestinal Polypeptide)**

**ABA review:** There was moderate-to-high punctate staining throughout the cerebral cortex, the glomerular and olfactory nerve cell layers of the main olfactory bulb. Interestingly, there was very high labeling of cells in the optic chiasm. In one slide, there was high level of expression in the ventral portion of the periaqueductal gray and possibly the adjacent medial longitudinal fascicle. An independent review of the ABA images indicated very specific expression in the VIPergic neurons of cortex that are few in number and dispersed in the upper layers of cortex.

Sagittal section

**GENSAT:** Two BAC transgenic lines have identical expression at P7. The BAC data is correct but incomplete. Correct expression is observed in suprachiasmatic nuclei, optic tract, superior colliculus and lateral geniculate nuclei.

**Literature:** In the adult mouse brain, *Vip* mRNA is very dense in the raphe and vestibular nuclei and prominent in the olfactory bulb, hippocampus, and orbital, parietal, and insular cortices [132]. In these regions, there is significantly more mRNA in the segmental trisomy mouse (model of Down Syndrome) compared with wild-type littermates.

**14. *Ddit4l* (DNA-Damage-Inducible Transcript 4-Like)**

**ABA review:** Within the cortex there was very high expression along the entire length of the layer 2/3. In some section there appeared to be higher expression in the orbital and the secondary somatosensory areas. Moderate expression levels were observed in the glomerular layer of the main olfactory bulb. In the cortical subplate there was moderate labeling of cells in the lateral amygdalar nucleus and the ventral portion of the basolateral amygdalar nucleus. Moderate-to-strong expression could be seen in the hippocampal CA1, CA2, CA3 fields. In the retrohippocampal formation there was very strong labeling in layer 2 of the pre and post subiculum. Low punctate staining for *Ddit4l* was present throughout the caudoputamen. Finally, in the pons there appeared to be low but specific expression in the motor nucleus of trigeminal and in the medial segments of the superior olivary complex. There was very high and specific level of expression in layer 2 (and possibly 3) of the cerebral cortex. The staining extends from infralimbic area to the agranular insular area ventral part. An independent review indicated limited number of cell populations in layers 2-3 of cortex and CA1 (deep neurons). There was nice expression in lateral amygdaloid nuleus– but neither the central or basal groups have any signal.

Sagittal section Coronal section

**GENSAT**: No information available

**Literature**: No expression studies have been reported.

**15. *Dkkl1* (Dickkopf-Like 1)**

**ABA review:** Moderate-to-high expression of *Dkkl1* was observed throughout the cortex. There was high expression in layer 2/3 of the primary, anteromedial and anterolateral visual areas. The adjacent retrosplenial area displayed slightly higher levels of staining for *Dkkl1*. Expression was found to be slightly higher in the piriform area, layers 4 and 5 of the ventral/dorsal segments of the anterior cingulate area, and the primary/secondary motor cortex. Moderate-to-high expression was also observed in the glomerular and mitral layers of the main olfactory bulb and the anterior olfactory nucleus. In addition, there were very high levels of staining in the accessory olfactory bulb. Within the hippocampal formation there was moderate-to-high expression in the granular layer of the dentate gyrus and moderate expression in the pyramidal layer of the CA1, CA2 and CA3 fields. There was significant moderate-to-high punctate expression throughout the midbrain, brainstem and cerebellar Purkinje and granular cell layers. Within the medulla there was somewhat higher expression in the hypoglossal nucleus and the magnocellular reticular nucleus.

Sagittal section Coronal section

**GENSAT**: No information available.

**Literature:** This gene is also called *Soggy*. SAGE analysis revealed regional enrichment in the cingulate cortex of adult C57BL/6 mice [10]. No in situ hybridization or transgene studies have been reported.

**16. *Rspo2* (R-spondin 2)**

**ABA review:** There was moderate-to-strong expression in layers 3 and dorsal part of the layer 5 along the prelimbic, primary/secondary motor, orbital (medial), and anterior cingulate (dorsal) areas. Moderate punctate staining could be seen throughout layer 6a and to a lesser extent in the claustrum and dorsal endopiriform nucleus. Lateral to the claustrum, there was moderate labeling of cells in layer 5 and 6a of the perirhinal and ectorhinal areas. Slightly higher expression levels were found in layers 2/3, 4, and 5 of the lateral part of the entorhinal cortex. Moderate staining was present in the lateral amygdalar nucleus and the anterior portion of the basolateral amygdalar nucleus. Within the hippocampal formation there was moderate-to-high expression in CA1 and CA3 pyramidal cell layers. In the retrohippocampal region there were similar levels of staining of cells in the pyramidal layer in the dorsal and ventral parts of the subiculum. In the pons, there was also moderate-to-high staining in the motor nucleus of trigeminal and the facial motor nucleus. Similar levels of expression were also found in the hypoglossal nucleus of the medulla.

An independent review indicated expression mainly in anterior somatosensory cortex, hippocampal/olfactory-related cortices, and in the deeper cortical cell populations (layers V-VI, mainly V). There was very limited, if any, expression elsewhere.

Sagittal section Coronal section

**GENSAT**: No information available

**Literature:** No relevant information found in the literature.

**17. *Ier5* (Immediate Early Response Gene 5 Protein)**

**ABA review:** Moderate levels of expression were found in the layers 2/3, 5 and 6 of the cerebral cortex. Virtually no *Ier5* staining could be seen in the layer 4 and white matter tracts. Moderate staining was observed in the anterior olfactory nucleus. Low levels of expression could be seen in the caudoputamen and to a lesser extent the thalamus. In the hippocampal formation, there was moderate-to-high expression in CA1 and CA2 field pyramidal layer; slightly lower levels of expression were found in the CA3 field. In the dentate gyrus there was moderate-to-high expression in the granule cell layer. In the pons there was moderate expression in the pontine gray. An independent review indicated moderate expression in about 50% of the cells in almost all layers of cortex (fewer in layer 4). Only sporadic cells in the hippocampal formation were labeled.

Sagittal section Coronal section

**GENSAT:** BAC data matches the in situ data from BGEM and Allen Brain Atlas. The BAC produces strong expression in the dentate gyrus, cortex, inferior colliculus and cerebellum external granule cell layer, as measured by epifluorescence.

**Literature:** To investigate gene expression in the brain across the sleep-waking cycle, mRNA differential display and cDNA microarrays was used screen genes expressed in the cerebral cortex of rats; *Ier5* was one of 44 genes upregulated in waking and sleep deprivation [133].

**18. *Igfbp6* (Insulin-like Growth Factor-Binding Protein 6 Precursor)**

**ABA review:** Moderate expression was present in layers 2, 5, and 6a of the cortex. A band of cells along the length of layers III/IV did not show any *Igfbp6* staining. Moderate labeling was present in layer 2 of the piriform, piriform-amygdalar area and the medial and lateral zones of the posterior part of the cortical amygdalar area. Similar levels of expression could also be seen in basolateral, basomedial, and posterior amygdalar nuclei. Low-to-moderate levels of expression could be found in granular layer of the main olfactory bulb. Moderate-to-high levels of expression were found in cells in the pyramidal layer of the dorsal portion of the subiculum. In the pons there was moderate expression in the pontine gray and tegmental reticular nucleus. In the medulla there was moderate-to-high expression in the facial motor nucleus and lateral reticular nucleus. Strong expression was seen in the cerebellar Purkinje cell layer.

Sagittal section Coronal section

**GENSAT:** No information available

**Literature:** SAGE analysis revealed regional enrichment of *Igfbp6* in the somatosensory cortex of adult C57BL/6 mice [10]. Highest levels of expression in the adult rat brain are in the hindbrain, spinal cord, cranial ganglia, and dorsal root ganglia; these nuclei in the hindbrain and periphery that express *Igfbp6* are all associated with the coordination of sensorimotor function in the cerebellum [134]. Overexpression of *Igfbp6* in the brain under the control of *Gfap* promoter results in dysregulation of energy homeostasis in mice [135].

**19. *Ephb6* (Ephrin Type-B Receptor 6 Precursor)**

**ABA review:** Moderate-to-high levels of expression were found in the layers 2/3, 5 and 6 of the cerebral cortex. Low levels of staining could be seen in the layer 4 of the cortex; similar levels of labeling could be seen in layer 2 of the piriform area and the adjacent lateral zone of the posterior segment of the cortical amygdalar area. In the hippocampal formation moderate-to-high expression was observed in the Ammon’s horn. Moderate-to-high levels of staining levels were found in cells in the pyramidal layer of the dorsal portion of the subiculum. Low level punctate staining could be seen throughout the cerebral cortex. However, moderate levels of expression were observed in the anterior part of the basolateral amygdalar and the lateral amygdalar nuclei. Similar levels of expression could be seen in the paraventricular nucleus of the thalamus. Low-to-moderate punctate staining could be seen throughout the brainstem. However, there was moderate-to-high expression in the superior olivary complex (periolivary region) and facial motor nucleus of the pons and medulla, respectively. In an independent review of the ABA images, moderate label was found throughout cortex in coronal sections, some label was observed in amygdala also.

Sagittal section Coronal section

**GENSAT:** No information available

**Literature:** This gene is also called *Hep*, *Mep* and *Cekl*. Northern analysis and immunoblot indicated *Hep* expression in the normal human brain [136]. Analysis of mRNA levels in a variety of mouse tissues shows that *Mep* is highly expressed in thymus and brain [137].

**20. *Mpped1* (Metallophosphoesterase Domain Containing 1)**

**ABA review:** Moderate-to-high expression levels were present in layers 2-6a of the cerebral cortex. The expression was slightly higher in the dorsal and ventral segments of the anterior cingulate area. Low-to-moderate levels of expression could be found in granular layer of the main olfactory bulb. High expression was observed in the lateral part of the anterior olfactory nucleus. Similar levels of staining were found in the dorsal part of the endopiriform nucleus and claustrum. There may also potentially be some expression in layer 6 of the adjacent of the insula. Very high expression amounts were present in CA1 and CA2 field pyramidal layer of hippocampal formation. In the retrohippocampal region there were high levels of *Mpped1* staining in the lateral segment of the entorhinal area. Moderate expression was also present in bed nuclei of the stria terminalis, posterior division, principal nucleus and in the central amygdalar nucleus. In the brainstem low-to-moderate expression amounts were found in the spinal tract of the trigeminal nerve. An independent review of the ABA images indicated very strong label in all regions and levels of cortex. Additionally hippocampus CA1 was strongly labeled, as well as the dorsal and medial amygdala.

Sagittal section Coronal section

**GENSAT:** No information available

**Literature:** This gene is also called *239AB* or *FAM1A*. A transcript of ~3.6kb is uniquely expressed in the adult human brain [138].

**21. *Pak7* (Serine/Threonine-p21-Activated Kinase 7)**

**ABA review:** Moderate levels of expression were found in the layers 2/3, 4 and 6 of the cerebral cortex. Slightly higher levels of staining could be seen in layer 2 of the piriform area. Moderate but dense labeling was also found in the lateral amygdalar nucleus and the anterior and posterior segments of the basolateral amygdalar nucleus. Moderate-to-high expression was observed in the Ammon’s horn and granule cell layer of the dentate gyrus. Some sparse, low level expression was observed in the main olfactory bulb and brainstem.

Sagittal section Coronal section

**GENSAT:** No information available

**Literature:** This gene is also called *Pak5*. Northern analysis indicated strong expression in the human brain (region not indicated) [139]. In the mouse brain, *Pak5* mRNA was expressed throughout all regions of the brain, with higher levels of expression detected in the cerebellum, cerebral cortex and olfactory bulb. In cerebellum, discrete and intense signals were detected in the granular cell layer, while basal level of expression was detected in Purkinje and molecular cell layers [140].

**22*. Satb2* (Special AT-rich Sequence Binding Protein 2)**

**ABA review:** There was very specific labeling of the cerebral cortex. Moderate-to-high expression could be seen throughout layers 2/3, 4, 5 and 6a; slightly higher levels of expression could be seen in layers 2 and 3 of the taenia tecta and the piriform area. Moderate-to-high expression could be seen along the length of the cortical subplate. Interestingly, there was a small group of cells with very high levels of staining in the claustrum and the dorsal part of the endopiriform nucleus. In some non-consecutive slides, there were low levels of labeling in the brain stem and the granular layer of the cerebellum.

Sagittal section Coronal section

**GENSAT:** Both literature and BGEM in situ hybridization suggest that *Satb2* mRNA is primarily expressed in developing neocortex. Two BAC lines have almost identical expression post-natally. Besides the cortex, additional expression sites are observed at P7 compared with BGEM in situ data. These sites include main olfactory bulb, thalamus, cerebellum, medulla and hippocampus. In the adult, the BAC data matches Allen in situ data. Expression in pons and hippocampus is confirmed.

**Literature:** Using RT-PCR, Western analysis and immunohistochemistry it was shown that *Satb2* expression is restricted to a subset of postmitotic, differentiating neurons in the rat neocortex at ages E16 and P4. The authors suggested that similar to its homologue *Satb1*, *Satb2* is also involved in regulating gene expression through altering chromatin structure in differentiating cortical neurons [141].

**23. *Cplx3* (Complexin 3)**

**ABA review:** Expression of *Cplx3* seemed to be confined primarily to cortical subplate where it was expressed at high levels in layer 6b and the dorsal endopiriform nucleus. There was some moderate punctate staining in the third and lateral ventricle. It should be noted that there was some inconsistent expression in other brain regions. For example, in some slides there was moderate expression in the granular layer of the main olfactory bulb. Also, there appeared to be moderate-to-strong expression in the cerebellar granular cell layer. Finally, some coronal sections suggested moderate punctate staining throughout the medulla. An independent review of the ABA images indicated that in antero-frontal sections only the cells that wrap the anterior forceps of the corpus callosum were labeled, which continues as the deepest layer 6 cells throughout cortex with sporadic cells in reticular nucleus.

Sagittal section Coronal section

**GENSAT:** No information available

**Literature:** This gene is also called *CPXIII*. *CPXIII* mRNA is found in many regions of the adult mouse brain including, pyramidal cells in the hippocampal CA, granule cells in the dentate gyrus, cerebellum granule cell and Purkinje cell layers [142].

**24. *E430002G05Rik* (Hypothetical Protein)**

**ABA review:** Moderate-to-high expression could be seen along the length of layer 2/3 of the cerebral cortex; within this layer, the visual area and adjacent supplemental somatosensory area, a number of cells had high levels of staining. Moderate-to-high expression could also be seen in layer 2 of the taenia tecta. In the main olfactory bulb there was low-to-moderate labeling in the glomerular and mitral layers. In the retrohippocampal area, moderate-to-high staining was found in the ventral part of the pyramidal layer of the subiculum. The thalamus contained a number of regions with moderate expression of *E430002G05Rik* including: central lateral and medial nuclei, paracentral nucleus, paraventricular nucleus, and the medial habenula. High levels of labelling were present in the nucleus incertus of the pons. Moderate-to-high expression could also be seen in the lateral division of the external lateral segment of the parabrachial nucleus. In the medulla there was moderate staining in the area postrema and the commissural part of the nucleus of the solitary tract. Within this region there was also moderate-to high expression in the spinal tract of the trigeminal nerve and the central canal of the spinal cord/medulla.

Sagittal section Coronal section

**GENSAT:** No information available

**Literature:** No brain expression studies have been reported.

**Subanatomical region: Anterior CINGULATE Cortex, Therapeutic interest: Pain alleviation**

The anterior cingulate cortex (ACC) is the frontal part of the cingulate cortex, and includes both the ventral and dorsal areas of the cingulate cortex. It appears to play a role in a wide variety of autonomic functions, such as regulating blood pressure and heart rate, as well as rational cognitive functions, such as reward anticipation, decision-making, empathy and emotion. The ACC is part of a neuronal network involved in processing pain perception from peripheral stimuli. It is hypothesized that potentiation of excitatory responses within the ACC contributes to chronic pain and pain-related mental disorders [143].

**1. *Egr1* (Early Growth Response 1)**

**ABA review:** Moderate-to-high staining was found throughout the cerebral cortex, main olfactory bulb, caudo-putamen, and striatum. Within the cortex there appeared to be higher expression in layer 2/3 of the anterior cingulate area. However, it should be noted that adjacent areas including the retrospenial and visual areas also displayed similar levels of labeling. In the hippocampal formation, there was also very high staining in the pyramidal layer of the CA1, CA2 and CA3 fields. In the remaining brain regions moderate, sparse, punctate expression was observed. An independent review of the ABA images indicated that expression was found throughout the forebrain including, striatum, hippocampus, and olfactory bulb.

Coronal section Coronal (zoomed)

**GENSAT:** Three BAC transgenic mouse lines are identical at P7. The BAC produces expression in both neurons and glia. The overall expression pattern agrees with BGEM in situ hybridization data and the literature.

**Literature:** The gene is also called *Zif268*. In *Egr1*::*lacZ* transgenic mouse brain sections, beta-galactosidase expression was present within a subset of neurons and in a subpopulation of endothelial cells and vascular smooth muscle cells lining the large arteries of the subarachnoid and subpial space [144]. SAGE analysis revealed regional enrichment of *Egr1* in the cingulate cortex of adult C57BL/6 mice [10]. Microarray analysis revealed regional enrichment of *Egr1* in the adult rat parietal cortex [15]. In the neocortex of the rat brain, *Zif268* expression rose sharply in the sensorymotor area between postnatal days (PND) 10 and 12 [145]. In the frontal and occipital cortex, in contrast, an increase in *Zif268* mRNA levels was first seen on PND 14. After PND 17, levels decreased in the sensory-motor and the frontal cortex but remained high in the occipital and the piriform cortex. *Zif268* expression is induced in neurons of the nucleus accumbens shell and the anterior cingulate cortex during the retrieval of contextual but not cued fear memories [146]. *Egr1* null mice display reduced nociceptive behaviors to persistent inflammatory pain and inflammation increased *Egr1* expression in the anterior cingulate cortex of wild-type mice [147].

**2. *Stmn1* (Stathmin 1)**

**ABA review:**  Very high expression of *Stmn1* was observed in the anterior cingulate cortex. However, it should be noted that virtually all of the medial areas also expressed this gene at similar levels. In addition, similar levels of staining were observed in the taentia tecta, anterior olfactory nucleus, and the layer 2 of the piriform area. In the rest of the cerebral cortex very dense moderate-to-high staining was found. In the main olfactory bulb there were similar levels of expression in the glomerular, mitral and granular cell layers. Very high expression in the subependymal zone, high expression in the medial portions of the thalamus, and moderate levels of staining in the remaining regions of the brain were noted.

Sagittal section Coronal section

**Literature:** This gene is also called *p19* or *Op18*. In rat and human central nervous system, stathmin immunoreactivity was localized to the perikaryon and all processes, but not the nucleus, of neurons and oligodendrocytes [148]. Particularly numerous stathmin-immunoreactive neuronal cell bodies were found in the pyriform, cingulate, and neocortex, as well as in many cholinergic nuclei of the basal forebrain and brainstem, in the medial thalamus, in various brainstem nuclei, and choroid plexuses. Stathmin is highly expressed in the lateral nucleus (LA) of the amygdala as well as in the thalamic and cortical structures that send information to the LA about the conditioned (learned fear) and unconditioned stimuli (innate fear). Amygdala slices isolated from stathmin null mutant mice show deficits in spike-timing-dependent long-term potentiation (LTP) [149]. Null mutant mice also exhibit decreased memory in amygdala-dependent fear conditioning and fail to recognize danger in innately aversive environments. In a study that compared the ACC gray matter proteomes between schizophrenia and controls, *Stathmin1* was identified as altered in schizophrenia [150].

**4. *Cckbr* (Cholecystokinin B Receptor)**

**ABA review:** Moderate expression of *Cckbr* could be found throughout the cerebral cortex without any discernable enrichment of expression in any particular area. Somewhat sparser labeling was also observed in the granular and mitral layers of the main olfactory bulb. A similar pattern of expression was also observed in the anterior olfactory nucleus. Interestingly, low-to-moderate levels of staining were also found in the red nucleus and the ventral posteromedial nucleus of the thalamus of the midbrain and thalamus, respectively.

An independent review indicated that expression was observed in all of cortex (including anterior cingulated), from rostral to caudal and all levels. Additionally, amygdala is labeled, as well as all non-cortical territories including the hippocampus, striatum and the red nucleus.

Sagittal section Coronal section

**GENSAT:** No information available

**Literature:** This gene is also called CCK2 receptor. In the rat brain, unspliced precursor mRNA and the mature form were identified in the cerebral cortex, hypothalamus, and hippocampus in apparently differing proportions according to the region examined, suggesting that the expression of the *Cckbr* could be modulated at a post-transcriptional level [151]. *Cckbr* null mutant mice display gene dose-dependent changes in the activity of the dopaminergic system [152]. The sensitivity of presynaptic dopamine receptors was increased in heterozygous (+/-) and homozygous (-/-) animals, whereas the increase in sensitivity of post-synaptic dopamine receptors was apparent only in homozygous (-/-) mice. A null mutation of CCK2 receptor gene induces anxiolytic-like action in light-dark exploration, but not in fear conditioning test [153]. In situ hybridization revealed a 51% decrease of the full length *CCKBR* mRNA in the outer layers (II-III) of the frontal cortex of the schizophrenic brain [154]. The corresponding alterations for the truncated isoform were a 65% reduction in the outer layers and a 62% reduction in the inner layers (IV-VI) of the frontal cortex. Deletion of the CCK2 receptor gene reduces mechanical sensitivity and abolishes the development of hyperalgesia in mononeuropathic mice [155].

**5. *Adcy1* (Adenylate Cyclase 1)**

**ABA review:** Moderate levels of expression could be seen in layers 2/3, 4, 5, 6a of the cerebral cortex and in the medial zone of the posterior segment cortical amygdalar area. In the hippocampal formation there was very high labeling of Adcy1 in the granule cell layer of the dentate gyrus; slightly lower levels of staining were seen along the length of the pyramidal cell layer of Ammon’s horn. In the thalamus there was moderate expression in the reticular nucleus of the thalamus and the ventral group of the dorsal thalamus (ventral anterior-lateral complex, ventral medial nucleus, ventral posterior complex). Sparse moderate level staining could be seen in the midbrain and brainstem. Finally, moderate-to-high expression was present in the cerebellar Purkinje cell layer and the underlying granular cell layer.

An independent review indicated nice expression in all layers and regions of neocortex. There was also fairly rich expression in hippocampus, thalamus, cerebellum and lessor expression in other areas.

Sagittal section

**GENSAT:** No information available

**Literature:** Wild-type, adenylyl cyclase mutants AC1, AC8, or AC1 AC8 double knockout mice were indistinguishable in tests of acute pain, whereas behavioral responses to peripheral injection of two inflammatory stimuli, formalin and complete Freund's adjuvant, were reduced or abolished in AC1 and AC8 double null mutant mice. AC1 and AC8 are highly expressed in the anterior cingulate cortex (ACC) [156]. Intra-ACC administration of forskolin rescued behavioral allodynia defective in the AC1 and AC8 double null mutant mice. The loss of adenylyl cyclase I activity disrupts patterning of mouse somatosensory cortex [157]. AC1 immunoreactivity is significantly decreased in dementia of Alzheimer type (DAT) brains [158]. There is a significant correlation of AC1 immunoreactivity with Ca2+/CaM-sensitive AC activity. Ca2+/CaM-sensitive AC activity was significantly lower in DAT than in the control, indicating that impairment of Ca2+/CaM-sensitive AC1 is involved in the pathophysiology of DAT.

**Subanatomical region: Insula Cortex, Therapeutic interest: Pain alleviation**

The insular cortex is involved in the processing of visceral sensory, visceral motor, vestibular, attention, pain, emotion, verbal, motor information, inputs related to music and eating, in addition to gustatory, olfactory, visual, auditory, and tactile data [159]. Recent neuroimaging data revealed that the insular cortex was involved in various neuropsychiatric diseases such as mood disorders, panic disorders, obsessive-compulsive disorders, eating disorders, and schizophrenia. In the processing of pain stimuli, the insula cortex is part of the neuronal network that includes the somatosensory and ACC areas of the cortex. The insula has been proposed to be involved in the emotional aspects of pain and pain-related learning and memory.

**1. *Lxn* (Latexin)**

**ABA review:** According to one review of the ABA images, there are discretely positive and strong expression in neurons of hypoglossal nucleus, Purkinje cells, inferior olive, motor trigeminal, entorhinal and associated cortex, hippocampal pyramidal cells. In cortex proper, there was a tightly packed area of deep cortical cells in lateral cortex that is continuous with claustrum posteriorly and insula anteriorly. An independent review of the ABA images indicated that in the main olfactory bulb, there was low-to-moderate expression in the granular and glomerular cell layers. In the cortical subplate, there was high expression in the claustrum, basolateral amygdalar nucleus and the dorsal part of the endopiriform nucleus. To some extent there may also be high expression in the adjacent insula layer 6. Some strong punctate staining was also present in the deeper layers (6a) of the visceral and supplemental somatosensory regions. Within the hippocampal formation, there was moderate-to-high staining in the pyramidal layer of the CA1, CA2 and CA3 fields. In addition, there was high density, moderate expression in layer 6 of the hippocampal formation. In the retrohippocampal region, there was very high expression throughout the ventral and dorsal parts of the pyramidal layer of the subiculum. In the medulla, there was very strong expression in the lateral segment of the paragigantocellular reticular nucleus. Within the cerebellum, there was very high expression in the Purkinje cell layer. It should be noted that there was significant low-to-moderate punctate expression throughout the brain.

Sagittal section Coronal section Coronal (zoomed)

**GENSAT:** No information available

**Literature:** Latexin-immunoreactive neurons are restricted essentially to the infragranular layers of lateral cortical areas in the rat [160]. The overall density, laminar or sublaminar localization, and cell size distribution of latexin+ neurons differed substantially across cytoarchitectonic areas within lateral cortex. Numerous latexin+ neurons had the morphology of modified pyramidal cells especially of layer VI. In the rat brain, in situ hybridization showed that latexin mRNA is synthesized in a subset of neurons in the lateral but not the dorsal neocortex [161]. In another study, it was shown that latexin is expressed in a subset of neurons in the secondary somatosensory, agranular and granular insular cortex areas, and the claustrum of the wild-type, but not of Lxn-/- mice [162]. There is reduced pain sensitivity in mice lacking latexin, an inhibitor of metallocarboxypeptidases [162].

**2. *Ntng2* (NetrinG2)**

**ABA review:** In the cerebral cortex there was moderate expression throughout layer 2/3 and 5. In cortex layer 5 there was very high expression in the dorsal part of the agranular insular area and the auditory area, whereas in layer 2/3 there was very high expression in the taenia tecta. In addition, there was very high expression in layer 2 of the anterior olfactory nucleus. In the cortical subplate there was strong expression in the dorsal part of the endopiriform nucleus, claustrum, somatomotor areas, and the posterior portion of the basolateral amygdalar nucleus. In the hippocampal formation there was moderate-to-strong expression in the granular layer of the dentate gyrus and in the pyramidal layer of the CA3 field; somewhat lower levels of labeling were also observed in the CA1 and CA2 fields. Within the retrohippocampal region there was very strong expression throughout layer 2 of the entorhinal area. Similar levels of expression were also observed throughout the subiculum. In the hypothalamus there was strong and specific staining of the subthalamic nucleus. Moderate-to-strong punctate labeling was observed throughout the midbrain; in particular within this region there was significant staining in the superior colliculus. In the pons there was moderate-to-strong expression in the motor nucleus of trigeminal, tegmental reticular nucleus, laterodorsal tegmental nucleus and the sublaterodorsal nucleus. In the medulla there was high expression in the lateral reticular nucleus. Moderate-to-high levels of labeling are also present in the interpolar portion of the spinal nucleus of the trigeminal. In addition there was moderate expression in the hypoglossal, cuneate, gracile nuclei and dorsal/ventral cochlear nuclei. Within the cerebellum there was moderate-to-high punctate staining in the arbor vitae and the cerebellar peduncles.

An independent ABA review found label in deep layers in anterior cortex with the claustrum and endopiriform cortex nicely labeled. There was strong expression in Isles of Calleja and in the deep aspects of the insular cortex that extend into the deep layers of dorsal cortex.

Sagittal section Coronal section

**GENSAT:** BAC data is consistent with the literature and the in situ hybridization data from BGEM and Allen Brain Atlas. In the adult mouse brain, compared with Allen in situ data, the BAC produces much weaker expression in claustrum. In addition, the cerebellum is strongly labelled in BAC transgenic mice, whereas weak cerebellar expression is seen in the in situ hybridization data.

**Literature:** This gene is also called *Laminet2*. In the P20 mouse brain, *Laminet2* is expressed in the reticular thalamic nucleus, habenular nuclei, hippocampal pyramidal neurons, layers III and V of the parietal cortex, and the superficial layers of the retrosplenial cingulate cortex [163]. In the human brain, alternative splicing of the gene is suggested by the presence of two transcripts in all regions tested, with the possible exception of the cerebellum and medulla, where only the smaller sized transcript was present [164]. An association between *NTNG2* and schizophrenia was also observed with SNPs and haplotypes that clustered in the 5' region of the gene [165].

**3. *Nr4a2* (Nuclear Receptor Subfamily 4, Group A, Member 2)**

**ABA review:** In the subcortical plate there was high expression in the claustrum and the dorsal part of the endopiriform nucleus and potentially the adjacent insula layer 6. In addition, there was moderate-to-strong expression along the length of layer 6b. Like *Lxn*, there was also strong punctate staining in the deeper layers (6a) of the visceral and supplemental somatosensory regions. Moderate punctate staining could be found throughout the brain. An independent review indicated that there is a continuous patch of labeled cells that goes from dorsal endopiriform cortex to claustrum and deepest cortical layer. This is similar to *Lxn* above, and has some label in Purkinje cells but seems to be more discrete then *Lxn*.

Sagittal section Coronal section

**GENSAT:** BAC data matches both literature and BGEM in situ data in general. In adult BAC mice, correct expression is seen in the main olfactory bulb, neocortex, hippocampus, subiculum, claustrum, thalamus (including medial habenular nucleus), hypothalamus, cerebellum and substantia nigra reticular. However, there seems to be very weak or no expression in the ventral tegmentum area. In addition, there was staining in the olfactory tubercle and interpeduncular nucleus that has not been reported before. At P7, BAC mice show much weaker staining in the claustrum than the in situ data.

**Literature:** This gene is also called *Nurr1*. In the adult mouse brain, *Nurr1* is expressed in dopaminergic neurons present in the substantia nigra, ventral tegmental area, retrorubral field, central grey, linear nucleus raphe and olfactory bulb [166]. In the adult rat, *Nurr1* is co-expressed in latexin-expressing neurons located in layer V, sublayer VIa, and the white matter of the lateral sector of the neocortex, and also in latexin-negative early born neurons in sublayer VIb of the entire neocortex [167]. Mouse *nurr1* null mutants lack the ventral midbrain dopamine neurons and die soon after birth [168]. Abnormal cortical and subcortical dopaminergic activities are among the most consistent neuropathological findings in schizophrenia [169]. There is a reduction of dopamine-related transcription factors Nurr1 and NGFI-B in the prefrontal cortex in schizophrenia and bipolar disorders.

**4. *Fezf2* (FEZ Family Zinc Finger 2)**

**ABA review:** In the cerebral cortex there was moderate expression along the length of the layer 2/3. In addition, there was moderate-to-high staining throughout layer 5. Within this layer slightly higher density and levels of expression was observed in the posterior part of the agranular insular, visual, ectorhinal and in the infralimbic areas. In the cortical subplate there was moderate expression in layer 6b and throughout the basolateral amygdalar nucleus. In the main olfactory bulb there was moderate-to-high staining in glomerular, mitral and granular cell layers. Moderate-to-high expression was found in the CA1, CA2 and CA3 pyramidal cell layers of the hippocampal formation. In the retrohippocampal region there was high expression in the dorsal and ventral segments of the subiculum, pyramidal layer. There was strong expression in the granular layer but moderate punctate staining throughout the cerebellum.

An independent review indicated enrichment of mRNA in layer 5 cortical neurons and label in superficial layers 2-3. In amygdala and hippocampus-related cortex, specificity of this gene was most evident in medial sections where only the cerebral cortex was positive and layer 5 neurons therein.

Sagittal section Coronal section

**GENSAT:** Six BAC lines have matching expression at P7. The BAC data is consistent with both literature and BGEM in situ data in general. In the adult stages, cortical expression remains in BAC mice, which is consistent with the literature and the in situ data from ABA. No specific hybridization signal is observed in BGEM adult data.

**Literature:** This gene is also called *Zfp312, Fezl* and *Fezf2*. In the adult mouse brain, *Zfp312* is selectively expressed in layer V and VI subcortical projection pyramidal neurons and their progenitor cells [170]. Downregulating *Zfp312* expression with small interfering RNAs dramatically reduced the number of subcortical axonal projections from deep-layer pyramidal neurons. In *Fezl*-deficient mice, projection neurons in cortical layer 5 display molecular, morphological, and axonal targeting defects [171]. The corticospinal tract was absent, corticotectal and pontine projections were severely reduced, and *Fezl*-expressing neurons formed aberrant axonal projections. Fez-deficient mice also show several abnormalities in the olfactory system: impaired axonal projection of the olfactory sensory neurons, reduced size of the olfactory bulb, abnormal layer formation in the olfactory bulb, and aberrant rostral migration of the interneuron progenitors [172].

**5. Ttc9b (Tetratricopeptide Repeat Domain 9B)**

**ABA review:** In the cerebral cortex there was moderate expression along the length of the layer 2/3. In addition, there was moderate to high staining throughout layer 5 and 6a. There was moderate to strong labeling in the lateral, medial, and posteroventral segments of the anterior olfactory nucleus. Very high expression levels were found in layer 2 of the piriform area. Within the hippocampal formation there was moderate expression in the granular layer of the dentate gyrus and in the pyramidal layer of the CA1, CA2 and CA3 fields. In the pons moderate expression could be found in the magnocellular segment lateral reticular nucleus. In another set of observation it was noted that the expression was specific to the layer 6 of the cortex in all regions including insular cortex

Sagittal section Coronal section

**GENSAT:** No information available.

**Literature:** No brain expression studies have been reported.

**Subanatomical region: Somatosensory Cortex, Therapeutic interest: Pain alleviation**

Somatic sensation consists of the various sensory receptors that trigger the experiences labelled as touch or pressure (mechanoreception), temperature (thermoreception), and pain (nociception). The primary somatosensory area in the human cortex is located in the postcentral gyrus (parietal lobe).

**1. *Rspo1* (R-spondin Homolog)**

**ABA review:** Gene expression was foundd to be cortex specific; in lateral sections, expression was found in somatosensory cortex and relatively specific to layers 3-4. An independent review of the ABA images indicated that in layer 2/3 of the cortex there was moderate-to-high labeling in the primary somatosensory area. To a lesser extent, expression was also observed in the primary/secondary motor, anterior cingulate and retrosplenial areas (in layers 2/3). Cells lining the lateral and third ventricles also displayed moderate staining. Low-to-moderate expression was present throughout the glomerular layer of the main olfactory bulb. There was inconsistent, punctate, staining throughout the brainstem and granular cell layer of the cerebellum.

Sagittal section Coronal section Coronal (zoomed)

**GENSAT:** No information available

**Literature:** Northern blot revealed only low expression in cerebrum of adult mouse [173]. In situ hybridization showed its expression in the dorsal part of the neural tube on 10 and 12 days post-coitus.

**2. *Cyp39a1* (Cytochrome P450, Family 39, Subfamily a, Polypeptide 1)**

**ABA review:** In layer 2/3 and 4 of the cortex, moderate-to-high labeling was observed in the primary somatosensory area in the coronal section, but not in the sagittal views. Low-to-moderate expression was observed throughout the glomerular layer of the main olfactory bulb. Moderate-to-high staining was seen in the lateral and third ventricles. Within the midbrain there was moderate expression in the dorsal raphe, and to some extent, in the anterior tegmental nucleus. The pons also displayed low level, scattered

expression in the pontine reticular nucleus; similar diffuse punctate staining was also seen throughout the medulla.

Sagittal section Coronal section Coronal (zoomed)

**GENSAT:** No information available

**Literature:** No brain expression studies have been reported.

**3. *Cartpt* (CART prepropeptide)**

**ABA review:** Moderate-to-high expression was observed in layer 2/3 of the primary and supplemental somatosensory area. *Cartpt* was also found at high levels in layer 2 of the piriform area. The striatum displayed strong staining in the rostroventral lateral septal nucleus, nucleus accumbens and the pyramidal layer of the lateral septal nucleus. Similar expression levels were observed in the bed nuclei of the stria terminalis, posterior division, and the principal nucleus of the pallidum. In the caudoputamen there was significant expression in the medial amygdalar nucleus. In the hypothalamus there was moderate-to-strong staining in the paraventricular hypothalamic nucleus, arcuate nucleus, and the ventromedial hypothalamic nucleus. Within the main olfactory bulb there was moderate expression in the granule layer. Low-to-moderate labeling of cells was observed in the medial and lateral segments of the anterior olfactory nucleus. Very strong staining was observed in the Edinger-Westphal nucleus of the midbrain. Moderate staining was also seen in the superior colliculus, motor related, deep gray layer. In the medulla strong staining was observed in the hypoglossal nucleus, the dorsal motor nucleus of the vagus nerve, the nucleus raphé obscurus, magnocellular part of the lateral reticular nucleus. Additionally, strong punctate staining of cells in the medial and lateral segments of the nucleus of the solitary tract was observed.

An independent review indicated strong label in nucleus accumbens, hypothalamus, midbrain, basal forebrain, a specific layer in the colliculus and the piriform cortex. The label is layer-restricted but appeared to be rather specific to somatosensory cortex.

Sagittal section Coronal section Coronal (zoomed)

**GENSAT:** BAC data is consistent with the literature and Allen Brain in situ hybridization data with expression in hypothalamus, amygadala, midbrain and brainstem. However, in cortex, the *Cartpt* mRNA is expressed in somatosensory cortex barrel field.

**Literature:** The gene is also called *Cart*. In the rat central nervous system, *Cart* mRNA has been localized to the mitral and tufted cells of the olfactory bulb, layer IV of the cerebral cortex and barrel field neurons of the somatosensory cortex [174]. The two alternatively spliced *Cart* variants present in the rat brain have identical and overlapping distributions in the rat forebrain. Cart peptide staining was observed in the nucleus accumbens, basolateral amygdala, olfactory bulbs, the cortical barrels, thalamic nuclei, the lateral and dorsal horns of the spinal cord, and the nuclei of the solitary tract [175]. In the human brain, in situ hybridization detected the most abundant expression in the hypothalamus and the thalamus [176]. High mRNA expression levels are found in the cortex (piriform, dorsolateral prefrontal, lateral orbital prefrontal, medial orbitofrontal, and middle temporal cortex), nucleus accumbens, bed nucleus of the stria terminalis, amygdala (central, cortical, and medial nuclei), hippocampus, expression was also detected in the locus coeruleus and dorsal raphe. The rat Cart (55–102) peptide produces anti-nociception in the mouse formalin test, a persistent pain model [177]. The results suggest that Cart is involved in supraspinal pain transmission.

**4. *Col5a1* (Collagen, Type V, Alpha 1)**

**ABA review:** Moderate expression of *Col5a1* was found in the glomerular cell layer of the main olfactory bulb. Within the cortex moderate-to-high expression was found along the medial (infralimbic and prelimbic areas) and ventral (orbital area) parts of layer 6a. In the subcortical plate there was moderate-to-high staining in layer 6b, claustrum, and posterior amygdalar nucleus. Higher expression in this region could be seen in the endopiriform area. Within the hippocampal formation there was moderate labeling along the length of layer3 and in the pyramidal layer of the ventral portion of the subiculum. In the medulla moderate-to-high expression was found in the hypoglossal nucleus and the lateral segment of the paragigantocellular reticular nucleus. In the cerebellum, strong staining was observed in the Purkinje cell layer.

An independent review indicated rather selective expression in cortical layer 6, although superficial cells also displayed label. Expression appeared to be beyond the somatosensory cortex and throughout the extent of dorsal cortex. Cerebellar Purkinje cells were also nicely stained.

Sagittal section Coronal section Coronal (zoomed)

**GENSAT:** No information available

**Literature:** No brain expression studies have been reported.

**5. *Rorb* (RAR-related Orphan Receptor Beta)**

**ABA review:** There was strong expression in layer 4 of dorsal cortex (less in posterior and not in ventral/frontal regions). There was nice labeling of nuclei in thalamus and a few other regions.

Sagittal section Coronal section Coronal (zoomed)

**GENSTAT:** The BAC line in the database is one of low copy number. Overall expression is weak in BAC transgenic mice. Cortical expression (layer 3/4) in BAC mice matches the literature, but the data shows less staining in thalamus than the in situ hybridization data indicates. At E15-5, the BAC data has weak to undetectable expression in the cortical plate. The gene will be retargeted to obtain higher expressing lines.

**Literature:** Rorb is downregulated in the barrelless mutant relative to control mice by quantitative comparison of expression patterns in layer IV somatosensory cortex [178].

**6. Loc433228 (hypothetical gene supported by AK082257)**

**ABA review:** Moderate levels of expression could be found throughout layer 4 of cerebral cortex. However, staining appeared to be stronger in the primary and supplemental somatosensory area and to some extent the visceral area. In layers 2/3 moderate labeling of Loc433228 was found in the frontal pole, orbital area, primary and secondary motor area, and the dorsal segment of the agranular insular area. In the thalamus, similar levels of expression were present in the ventral posterior complex of the thalamus including the ventral posterolateral and posteromedial nuclei of the thalamus. Moderate labeling was also found in the midline group of the dorsal thalamus including the paraventricular nucleus of the thalamus and nucleus of reunions. In addition, moderate staining was also found in the anterior group of the dorsal thalamus. Within the midbrain moderate expression was observed in the superficial gray and zonal layers of the superior colliculus.

Sagittal section Coronal section

**Literature:** No brain expression studies have been reported.

**GENSAT:** No information available.

**7. GNB4 (Guanine Nucleotide Binding Protein, Beta 4)**

**ABA review:** Very high expression levels are present in the layer 6a of the cortex, claustrum and dorsal/ventral segments of the endopiriform area. There was also some high level staining in the adjacent insula area (layer 6) and the basolateral amygdalar nucleus. Moderate punctate staining could also be seen throughout the entorhinal area, piriform, piriform-amygdalar, and postpiriform transition areas. Finally, moderate-to-high expression could also be seen in the cerebellar Purkinje cell layer. In another set of observations it was reported that there is a low levels of staining throughout the brain with the exception of the region in rostral, lateral cortex designated by atlases as claustrum and endopiriform area.

Sagittal section Coronal section

**GENSAT:** No information available.

**Literature:** No brain expression studies have been reported

**Subanatomical region: Astroglia, Therapeutic Interest: Alzheimer Disease**

Astroglia, the most abundant glial subtype, are involved in a number of functions in the central nervous system including: guidance and support of neuronal migration during development, maintenance of the blood brain barrier, regulation of cerebral blood flow, modulation of immune reactions by serving as antigen-presenting cells, stabilization of cell–cell communications in the central nervous system, and maintenance of the extra cellular environment [179]. For example, astrocytes play a critical role in the maintenance of the neuronal environment by providing nutrient support to neurons, regulating ion concentration in the extracellular space, releasing or reabsorbing neurotransmitters at the synaptic cleft, and modulating synaptic activity [180]. Aberrations in astroglial activity have been shown to play a role in a number of neurodegerative disorders including epilepsy, Alexander, Alzheimer, Huntington and Parkinson disease [179, 180]

**1) *Gfap* (Glial Fibrillary Acidic Protein)**

**ABA review:** Moderate to high expression could be seen in the glomerular layer of the main olfactory bulb. However, low-to-moderate staining could also be seen in the granular layer. Very high expression was present in the rhinocele and subependymal zone. Similar levels of labeling could be seen along the cortical white matter tracts. In the cerebellum there was to low but specific expression in the Purkinje and underlying granular cell layers. Finally, it should be noted that sparse low expression was also found throughout the brain.

Sagittal section Coronal section Coronal (zoomed)

**GENSAT:** In the adult mouse strong to moderate expression is found in 20 brain regions: accessory olfactory bulb, amygdala, anterior commissure, cerebellum, corpus callosum, fornix, globus pallidus, hippocampus, hypothalamus, medulla, midbrain, olfactory bulb, optic tract, piriform cortex, pons, pyramidal tract, rostral migratory stream, spinal cord dorsal horn, spinal cord ventral horn, and substantia nigra. The BAC data matches both the literature and BGEM *in situ* hybridization data. In adult BAC mice, the glial limitans in cerebellum are labeled. At P7, Bergmann glia are stained. Only residual staining of radial glia in the cortex is visible, as these cells are transforming into astrocytes at this age.

**Literature:** *Gfap* mRNA and protein was shown to increase in old mice [181]. Major white fiber tracts (corpus callosum, fimbria, stria terminalis, and optic tract) were found to have increased Gfap immunostaining and mRNA. *Gfap* mRNA has been localized to the cell soma and processes of reactive and some non-reactive astrocytes in the adult [182]*.* *Gfap*-expressing cells in the postnatal subventricular zone display a unique glial phenotype intermediate between radial glia and astrocytes [183]*.*

**2) *S100B* (S100 Protein, Beta Polypeptide)**

**ABA review:** Images displayed very nicely labels for astrocytes throughout sagittally sectioned brain. There were some non-specific staining in layers 5-6 cortex, the CA2 region in the hippocampus and the brainstem nuclei.

Sagittal section Coronal section Coronal (zoomed)

**GENSAT:** No information available.

**Literature:** *S100b* is found in microglia in the form of a filamentous network as well as diffusely in the cytoplasm and associated with intracellular membranes [184]*.* *S100b* immunoreactivity in rat brain glial cultures is associated with both astrocytes and oligodendrocytes [185]*.* Examination of homozygous null mice suggests that glial protein *S100b* may modulate long-term neuronal synaptic plasticity [186]*.*

**3) *Slc1a2* (Solute Carrier Family 1 (Glial High Affinity Glutamate Transporter), Member 2)**

**ABA review:** Very high levels of staining could be seen throughout the brain. The density of expression was highest in all layers of the cerebral cortex. In the underlying cortical subplate there was very high expression in layer 6b. Similar levels of labeling were present in the cells lining the lateral ventricle. However, expression was very much reduced in most the white matter tracts including the corpus callosum, columns of the fornix, and the ventral hippocampal commissure. In some coronal sections there were much lower levels of expression in the hypothalamus and the medial septal nucleus of the pallidum. In the cerebellum there were high levels of staining only in the Purkinje cell layer.

Sagittal section Coronal section Coronal (zoomed)

**GENSAT:** In the adult mouse strong to moderate expression is found in 19 brain regions: amygdala, anterior olfactory nucleus, caudate putamen, cerebellum, cerebral cortex, dorsal horn, entorhinal cortex, hippocampus, hypothalamus, medulla, midbrain, olfactory bulb, piriform cortex, pons, septum, substantia nigra, thalamus, ventral horn, and ventral striatum. Two BAC lines have matching expression at P7. The EGFP reporter gene is expressed in glial cells throughout the brain, consistent with the published data and the *in situ* data from ABA. In E15-5 Bac embryos, the transgene is highly expressed in postmitotic cells in the cerebral cortex, instead of mitotic cells as suggested by the literature.

**Literature:** This gene is also called *Glt1* or *Eaat2*. In the rat brain, immunocytochemistry revealed that *Glt1* was localized only to astroglia and is distributed in astrocytes throughout the brain and spinal cord [187]. In the mouse forebrain at E16, *Glt1* is expressed in the globus pallidus, perirhinal cortex, lateral hypothalamus, hippocampus, and fimbria and the axonal pathways interconnecting the neocortex, basal ganglia, and thalamus. In the cerebral cortex, *Glt1* immunoreactivity is present in the subplate and along fiber bundles in the intermediate zone. *Glt1* immunoreactivity is double-labeled by GAP-43, suggesting that *Glt1* is expressed in neurons at E16 [188]. Homozygous mice deficient in *Glt1*, a widely distributed astrocytic glutamate transporter, showed lethal spontaneous seizures and increased susceptibility to acute cortical injury [189]. *Slc1a2* was identified by microarray analysis as differentially expressed across a variety of mouse strains [190]. Ingenuity Pathways Analysis highlighted the NMDA/glutamate signaling pathway, which contained *Slc1a2* among three correlated genes overexpressed in mouse strains exhibiting resistance to seizures.

**4) *Plaur* (Plasminogen Activator, Urokinase Receptor)**

**ABA review:** There was widespread moderate expression throughout the brain in what appears to be astrocytes.

Sagittal section Sagittal (zoomed)

**GENSAT:** No information available.

**Literature:** The gene is also called *uPAR*. Immunohistochemical studies in cerebral malaria patients reported focal accumulation of *uPAR* expressing macrophages/microglial cells in Durck's granulomas. In addition, staining was also found adjacent to petechial hemorrhages in both astrocytes and endothelial cells [191]*.* Cultured human microglia was found to express surface *uPAR*. Addition of lipopolysaccharide to microglial cultures enhanced the proportion of *uPAR* expression and shifted cell morphology to the elongated spindle or bipolar shape. When microglia was examined immediately ex vivo, *uPAR* surface expression could not be detected. Microglia isolated from a patient with multiple sclerosis displayed a large amount of *uPAR*+ cells. These cells were predominantly spindle or bipolar in nature. These findings suggested that *uPAR* surface expression was associated with microglial activation [192].

**5) *Gcm1* (Chorion-Specific Transcription Factor)**

**ABA review:** Diffuse moderate-level punctate staining for *Gcm1* could be found throughout the brain. However, in the cerebellum there was an enrichment of expression in the Purkinje cell and underlying granular cell layers. In addition, there was low level but high density expression in the glomerular cell layer and the olfactory nerve layer of main olfactory bulb.

Sagittal section Sagittal (zoomed)

**GENSAT:** No information available.

**Literature:** The Gene is also called *Gcma*. Introduction of the mouse *Gcma* gene in cultured embryonic brain cells resulted in the induction of an astrocyte lineage [193]. Ablation of mouse *Gcma* did not cause a significant decrease in Gfap+ cells in cultures from mutant brains. Thus, mouse *Gcma*-expressing cells maybe a subpopulation of glial cells, distinct from the major astrocyte cell type. It has been suggested that the mouse *Gcma* ablation defect could be compensated by mouse *Gcmb*, which also has the potential to induce astrocytes.

**6) *Gcm2* (Glial Cells Missing Homolog 2)**

**ABA review:** Moderate-level punctate staining for *Gcm2* was present throughout the brain. The highest expression levels could be seen in the CA1, CA2 and CA3 field of the pyramidal layer of the hippocampal formation. Moderate-to-high labeling was also found in throughout the dentate gyrus, granule cell layer. In addition, there were moderate levels of staining in the cerebellar Purkinje cell layer and to some extent the underlying granular cell layer. In the main olfactory bulb there was moderate in the expression in the granular, granular, mitral and cell layers. Similar levels of labeling were also seen in the olfactory nerve layer.

Sagittal section Sagittal (zoomed)

**GENSAT:** In the adult mouse strong to moderate expression is found in 9 brain regions: amygdala, caudate putamen, cerebral cortex, hippocampus, internal capsule, medulla, subicular cortex, ventral horn, and ventral striatum. The gene is proposed to be involved in hyperparathyroidism. Two BAC lines have similar expression patterns at P7 even though the confirmation line expresses at a lower level. The BAC data does not match BGEM *in situ* hybridization data. More expression sites are detected in the GENSAT BAC mice. The BAC consistently expresses in striatum, substantia nigra and dorsal spinal cord. In cerebellum, *Gcm2* mRNA is shown to be present near Bergmann glial cells, whereas the BAC labels scattered neurons in the internal granule layer. At E15-5, BGEM data indicates *Gcm2* mRNA is expressed in the ventricular zones, which is absent in the BAC data.

**Literature:** The gene is also referred to as *Gcmb*. Transcripts of human *GCMb* were detected by RT-PCR in fetal brain (specific region not indicated) [194]. Although *Gcm2* is expressed in neural tissues at a low level, the major sites of expression for mammalian genes *Gcm1* and *Gcm2* are non-neural, suggesting that the functions of the mammalian homologs have diverged and diversified [195]. However, when expressed ectopically, *Gcm1* could substitute functionally for Drosophila *Gcm* by transforming presumptive neurons into glia.

**7. SERPINA3 (Serine/Cysteine Peptidase Inhibitor, Clade A, Member 3N)**

**ABA review:**  Very sparse, moderate-level punctate staining was present throughout the brain. In the hippocampal formation low level staining could be seen in the CA2 and CA3 field of the pyramidal layer. In some non-consecutive slides moderate to high labeling was present throughout cerebellar Purkinje cell layer.

Sagittal section Coronal section

**GENSAT:** No information available.

**Literature:** Also called ACT (antichymotrypsin). A nuclear factor-1 (NFI) binding element, located within the -13-kb enhancer of the human ACT gene, is indispensable for the full basal transcriptional activity of the ACT gene [196]. NFI likely cooperates with AP-1 to mediate astrocyte-specific expression. A 14-kb long 5'-flanking region of the ACT gene contains adjacent NFI and AP-1 elements that colocalized with DNase I-hypersensitive sites found in astrocytes and glioma cells. Knock-down of NFI expression also specifically abrogates the expression of astrocyte-specific marker Gfap. ACT has been shown to be a major constituent of the plaques associated with Alzheimer's disease [197].

**Subanatomical region: Microglia (constitutive), Therapeutic Interest: Alzheimer Disease**

Microglia are a population of glial cells that are referred to as the resident immune cells of the central nervous system. Microglia are highly mobile cells that can move to sites to neuronal injury and remove the damaged cells by phagocytosis. Like other phagocytic cells, such as macrophages and dendritic cell, activated microglia are capable of releasing a number to cytotoxic molecules such as proinflammatory cytokines, reactive oxygen intermediates, proteinases and complement proteins. Although controversial, it has been speculated that chronic activation microglia may cause or exacerbate a number of brain disorders. Aberrant activity of the inflammatory pathway in the brain is thought to play a role in a number of neurodegenerative disorders including Alzheimer disease, Parkinson disease, prion diseases, multiple sclerosis and HIV-dementia [198].

**1) *Cx3cr1* (CX3C Chemokine Receptor 1)**

**ABA review:** Examination of AIBS images for *Cx3cr1* showed ubiquitous expression throughout the brain. Expression was highest in the main olfactory bulb and the cerebral cortex. In addition, there was some low level expression in the cerebellum. Examination of images at higher magnification indicated that no neurons were stained. Instead, small unidentified cells displayed labeling for *Cx3cr1*.

Coronal section Coronal (zoomed)

**GENSAT:** No information available.

**Literature:** No expression data for *Cx3cr1* was found in the literature.

**2) *Itgam* (Integrin Alpha M)**

**ABA review:** There was moderate expression of *Itgam* throughout the brain. In addition, there was also moderate enrichment of staining in layers 2-3 of the cerebral cortex.

Sagittal section Sagittal (zoomed)

**GENSAT:** No information available.

**Literature:** Two independent studies have proposed that Itgam may be a surface marker for microglia [199, 200].

**Subanatomical region: Microglia (activated), Therapeutic Interest: Alzheimer Disease, Amyptrophic Lateral Sclerosis**

**1) *Cd68* (Macrosialin Precursor)**

**ABA review:** There was abundant and relatively high level expression throughout the brain. Interestingly, expression was much reduced in the white matter tracts including the corpus callosum and corticospinal tract. In addition, within the cerebellum there was also reduced expression in the white matter tracts including arbor vitae, middle cerebellar peduncle, and vestibulocochlear nerve.

Sagittal section Sagittal (zoomed)

**GENSAT:** No information available.

**Literature:** *Cd68* is a 110-Kd transmembrane glycoprotein of unknown function that is highly expressed by human monocytes and tissue macrophages [201].

**2) *Aif1* (Anti-ionized calcium binding adaptor molecule 1)**

**ABA review:** *Aif1* expression was present at low levels throughout the brain. In an independent review of the ABA images, it was noted that the *Aif1* labeling was very similar to the activated microglia gene *Cd68*.

Sagittal section Coronal section Coronal (zoomed)

**GENSAT:** No information available.

**Literature:** *Aif1* expression is present in activated microglial/macrophage cells following spinal cord injury in rats (Schwab et al., 2001). Monoclonal antibodies against *Aif1* localized the protein to a minor rat monocyte subpopulation of lymphoid tissue [202].

**3) *P2rx7* (Purinergic receptor P2X, ligand-gated ion channel, 7)**

**ABA review:** Moderate but sparse expression of *P2rx7* was seen throughout the brain. In a few saggital sections there was a relative increase in labeling in the main olfactory bulb, hippocampal formation and to some extent the cerebellum. In an independent review of the ABA images, it was noted that *P2rx7* could be found in the CA3 field of the hippocampal pyramidal layer and in cortical cells.

Sagittal section Coronal section Coronal (zoomed)

**GENSAT:** In the adult mouse strong to moderate expression is found in 20 brain regions: amygdala, anterior olfactory nucleus, basal forebrain, caudate putamen, cerebellum, cerebral cortex, dorsal horn, entorhinal cortex, fornix, globus pallidus, hippocampus, medulla, midbrain, olfactory bulb, piriform cortex, pons, septum, substantia nigra, thalamus, ventral horn, and ventral striatum. The BAC data is reproducible and is consistent with the literature. BGEM *in situ* hybridization data is not informative. At P7, it may show expression in cerebellum.

**Literature:** The gene is also referred to as *P2x7*. *P2x7* receptor immunoreactive profile was confined to resting and activated microglia in the epileptic brain [203]. *P2x7*-immunoreactivity is observed in S100b+ astrocytes throughout the hippocampus. *P2x7* protein is primarily localized along astroglial processes [204]. Immunocytochemistry and RT-PCR confirmed the expression of *P2x1*, *P2x4*, and *P2x7* receptor mRNA and protein on primary cultures of rat microglial cells and on the N9 microglial cell line [205].

**4) *Sulf2* (Sulfatase 2)**

**ABA review:** There was very high and specific level of expression in layer 2/3 of the cerebral cortex. There were also high levels of expression in the hippocampal formation. In a few slides some low but unconvincing expression could be seen in the cerebellum. In an independent review of the ABA images, it was noted that there was significant neuronal staining in the deep cortex and the regio inferior segment of hippocampus. However, the staining in the main olfactory bulb the staining appeared to be non-neuronal.

Sagittal section Coronal section Coronal (zoomed)

**GENSAT:** In the adult mouse moderate-to-strong expression is found in 14 brain regions, namely amygdala, basal forebrain, caudate putamen cerebral cortex, dorsal horn, hippocampus, hypothalamus, medulla, midbrain, olfactory bulb, pons, thalamus, ventral horn, and ventral striatum. Two BAC lines have almost identical expression at P7. The BAC transgene is expressed in both neurons and glia. The neuronal expression is found in cortex and subiculum, consistent with ABA *in situ* hybridization data. It is not clear from the diaminobenzidine data whether some neurons in the hippocampus are stained. However, in BAC mice, small glial-like cells are stained throughout the central nervous system, which greatly masks the neuronal expression.

**Literature:** *Sulf2* was identified as a new CNS-resident macrophage subpopulation molecular marker for the discrimination with murine systemic macrophages (Donnou et al., 2005).

**Subanatomical region: Oligodendroglia, Therapeutic Interest: Alzheimer Disease, Multiple Sclerosis**

Oligodendroglia is a glial cell type whose main function is to provide support to axons and to produce the myelin sheath, which insulates axons. A single oligodendroglial cell can form segments of myelin sheaths for several neurons at once. In addition, there are also satellite oligodendroglia that do not interact with the myelin sheath but rather are perineuronal and thought to regulate the microenviroment around neurons [206]. Disruption of function and/or injury of oligodendroglia is associated with the demyelinating disease multiple sclerosis and leukodystrophies [207]. In addition, oligodendroglia dysfunction has been linked to major depression and bipolar disorder [208].

**1) *Olig1* (Oigodendrocyte Transcription Factor 1)**

**ABA review:** Moderate levels of expression could be seen throughout the brain. In some slides, *Olig1* was slightly more enriched in the white matter tracts.

Sagittal section Sagittal (zoomed)

**GENSAT:** In the adult mouse moderate-to-strong expression is found in 15 brain regions: amygdala, anterior commissure, anterior olfactory nucleus, basal forebrain, caudate putamen, cerebellum, cerebral cortex, corpus callosum, dorsal funiculus, dorsal horn, entorhinal cortex, fornix, globus pallidus, hippocampus, hypothalamus, lateral funiculus, medulla, midbrain, olfactory bulb, optic tract, piriform cortex, pons, septum, subicular cortex, substantia nigra, thalamus, ventral funiculus, ventral horn moderate-to-strong and ventral striatum. The BAC data is reproducible and consistent with the literature and BGEM *in situ* hybridization data. Our BAC mice detect *Olig1* expression in more brain regions that is not evident in BGEM *in situ* data. But the highest expression sites in P7 BAC mice are consistent with the *in situ* data, which include corpus callosum, deep cerebellar nuclei, thalamus and substantia nigra.

**Literature:** In the adult rat brain, *Olig1*-expressing cells are found preferentially in areas rich in oligodendrocytes, such as the corpus callosum and cerebellar white matter, but can also be detected throughout the central nervous system. Persistent expression of *Olig1* suggests possible functions in the survival, proliferation, and/or maturation of oligodendroglia precursors [209]. *In situ* hybridizations revealed that *Olig1* was expressed in the ventral spinal cord of the developing mouse embryo as early as E9.5. *Oligo1* expression declined to undetectable levels by E10.5 and reappeared at E12.0 [210]*.* Mouse studies have indicated that homozygotes for targeted null mutations for OLIG1 show that the bHLH transcription factors *Olig2* and *Olig1* couple neuronal and glial subtype specification [211]. This common developmental requirement for *Olig1* function points to a potential motor neuron/oligodendrocyte connection [212]*.*

**2) *Ugt8a* (UDP Galactosyltransferase 8A)**

**ABA review:** *Ugt8a* expression is very cleanly relegated to axonal tracks. Expression level is moderate and non-specific to any given track.

Sagittal section Coronal section Coronal (zoomed)

**GENSAT:** No information available.

**Literature:** In the rat brain *in situ* hybridization studies revealed that *Ugt8a* expression was restricted to the oligodendrocyte-containing cell layers of cerebrum and cerebellum [213]. Targeted disruption of the *Ugt8* gene in mice results in myelination in the absence of galactocerebroside and sulfatide that produces a normal structure with abnormal function and regional instability [84]. Furthermore, mutant mice also have a functional breakdown of the lipid bilayer of the myelin membrane in central and peripheral nervous system by disrupted galactocerebroside synthesis [214].

**3) *Cnp* (2', 3'-Cyclic-Nucleotide 3'-Phosphodiesterase)**

**ABA review:** In a very specific manner, all oligodendroglia were very nicely labeled for *Cnp*. White matter tracks such as corpus callosum, fimbria, etc were packed with *Cnp* stained cells with very little if any other cell types.

Sagittal section Coronal section Coronal (zoomed)

**GENSAT:** No information available.

**Literature:** Monoclonal antibody Rip specifically recognizes *Cnp* in oligodendrocytes [215]. Most of the *Cnp* mRNAs are associated with the perikarya of oligodendrocytes in the mouse brain [216].

**4) *Gjb1* (Gap Junction Membrane Channel Protein Beta 1)**

**ABA review:** In the cerebellum there was moderate expression within the arbor vitae and throughout the cerebellar peduncles. To a lower extent, *Gjb1* was also present in the corpus callosum and corticospinal tract. Interestingly, *Gjb1* was also highly expressed in the main olfactory bulb.

Sagittal section Sagittal (zoomed)

**GENSAT:** No information available.

**Literature:** The gene is also referred to as *Cx32*. In the CNS, *Cx32* could be found in oligodendroglia and their processes, but not in compact myelin, and the levels of *Cx32* protein and mRNA increased during development in parallel with those of the other myelin genes [217].

**5) *Klk6* (Kalikrein 6)**

**ABA review:** Moderate to high expression of *Klk6* was found in the white matter of the cerebellum including the arbor vitae and the cerebellar peduncles. Low levels of labeling for *Klk6* were seen in other regions of the brain. In an independent review, the gene was reported to have intense hybridization to white matter areas in cells that were presumably oligodendroglia. However, tracks like the corpus callosum were not endowed with major amounts of *Klk6*+ cells.

Sagittal section Sagittal (zoomed) Coronal section

**GENSAT:** No information available.

**Literature:** *In situ* hybridization histochemistry revealed that the *Klk6* transcripts were localized to the mature oligodendrocytes [218].

**6) *Mag* (Myelin-Associated Glycoprotein)**

**ABA review:** *Mag* was found to be enriched in the cerebellar white matter and other white matter tracts where it was present within oligodendroglia. An independent review of the ABA images noted that *Mag* staining could also be seen in regions where oligodendroglia was not normally found such as the molecular layers in hippocampus, cerebellum and cortex.

Sagittal section Sagittal (zoomed)

**GENSAT:** No information available.

**Literature:** *Mag* transcripts are found in white matter and in satellite and other oligodendrocytes of the gray matter [219]. Microarray analysis revealed enrichment of *Mag* in the heavily myelinated pyramidal tracts and other ventral myelinated pathways surrounding dopaminergic and GABAergic cell bodies of the adult rat substantia nigra [15]. *Mag* immunoreactivity is found in the periaxonal portion of the myelinated fibers and in a small number of oligodendroglia in the cortex, hippocampus, and the spinal cord. The sheath of Schwann cells in unmyelinated fibers and satellite cells in the spinal ganglia are also immunoreactive for *Mag* [220]. In Mag-deficient mice, the onset of myelination was delayed, and subtle morphological abnormalities were detected in that the content of oligodendrocyte cytoplasm at the inner aspect of most myelin sheaths was reduced and that some axons were surrounded by two or more myelin sheaths [221].

**7) *Apod* (Apolipoprotein D)**

**ABA review:** Very high levels of expression could be seen in the white matter tracts, which include the olfactory nerve layer, corpus callosum, hippocampal commisures, fornix system and the arbor vitae. Similar levels of expression could also be seen in the cerebellar Purkinje cell layer. However, there was also a high level of background staining throughout the brain. In addition, there was expression outlining the surface of the brain, which may be indicative of pia matter labeling. In an independent review, it was noted that there was intense staining of a number of blood vessels in brain.

Sagittal section Sagittal (zoomed)

**GENSAT:** In the adult mouse moderate-to-strong expression is found in 15 brain regions: amygdala, caudate putamen, cerebellum, cerebral cortex, dorsal horn, hippocampus, hypothalamus, medulla, midbrain, olfactory bulb, pons, septum, thalamus, ventral horn, and ventral striatum. Three BAC lines have identical expression at P7. The overall expression patterns observed in the BAC mice are consistent with the literature and BGEM *in situ* hybridization data.

**Literature:** *In situ* hybridization studies identified *Apod* expression in the hippocampal fimbria, corpus callosum and other white matter tracts within the brains of aged (26 months) mice [222]. Microarray analysis revealed enrichment of *Apod* in the heavily myelinated pyramidal tracts and other ventral myelinated pathways surrounding dopaminergic and GABAergic cell bodies of the adult rat substantia nigra [15]. In rabbits, abundant levels of *Apod* mRNA are found near blood vessels. White but not gray matter shows high mRNA levels throughout both the rabbit central nervous system and in the human brain [223].

**8) *Enpp2* (Ectonucleotide Pyrophosphatase/Phosphodiesterase 2)**

**ABA review:** Moderate labeling of *Enpp2* was present in all the white matter tracts. However, high expression was also seen in layer 2 of cortex as well as other non-glial cells in the cortex. It could also be found at low-to-moderate levels in the main olfactory bulb, midbrain and the internal granular layer of the cerebellum.

Sagittal section Sagittal (zoom)

**GENSAT:** No information available.

**Literature:** Expression of *Enpp2* is enriched in brain and spinal cord, where its mRNA can be detected in oligodendrocytes and in cells of the choroid plexus. Expression in the brain increases during development with an intermediate peak of expression around the time of active myelination and maximal expression in the adult [224].

**9) *Fa2h* (Fatty Acid 2-Hydroxylase)**

**ABA review:** There was moderate expression of *Fa2h* in the corpus callosum and corticospinal tract. Within the cerebellum, *Fa2h* was enriched within the arbor vitae and throughout the cerebellar peduncles.

Sagittal section Sagittal (zoomed) Coronal section

**GENSAT:** No information available.

**Literature:** *Fa2h* has been shown to be the hydroxylase responsible for the synthesis of alpha-hydroxylated ceramide in myelinating oligodendrocytes [225].

**10) *Mal* (Myelin and Lymphocyte Protein)**

**ABA review:** *Mal* was highly expressed in the white matter tracts where it clearly labeled oligodendrocytes. However, a number nonconsecutive of slides indicated that *Mal* was expressed in significant amount throughout the brain.

Sagittal section Sagittal (zoomed)

**GENSAT:** No information available.

**Literature:** *Mal* is expressed in oligodendrocytes and Schwann cells. Microarray analysis revealed enrichment of *Mal* in the heavily myelinated pyramidal tracts and other ventral myelinated pathways surrounding dopaminergic and GABAergic cell bodies of the adult rat substantia nigra [15]. In the central nervous system, *Mal* is expressed during late steps of myelination. Mal protein appears approximately 3-5 days later than myelin basic protein and proteolipid protein. In contrast, in the peripheral nervous system, *Mal* transcript and protein expression is detected prior to the onset of myelination, as early as embryonic day 17. These results indicate that *Mal* is differentially expressed in oligodendrocytes and Schwann cells, likely reflecting different functions of the *Mal* proteolipid [226]. Mal is required for maintenance of proper axon-glia interactions in the central nervous system, and genetic ablation of Mal results in abnormal myelination [227].

**11) *Mbp* (Myelin Basic Protein)**

**ABA review:** Very high level of expression could be seen along the white matters tracts of the brain. However, significant *Mbp* staining was also observed throughout the brain.

Sagittal section Sagittal (zoomed) Coronal section

**GENSAT:** No information available.

**Literature:** *Mbp* mRNA is localized to oligodendrocytes [182]. *In situ* hybridization indicates that *Mbp* expression is predominantly localized over the nucleus of oligodendrocytes in white matter regions [228]. Microarray analysis revealed enrichment of *Mbp* in the heavily myelinated pyramidal tracts and other ventral myelinated pathways surrounding dopaminergic and GABAergic cell bodies of the adult rat substantia nigra [15]. The myelin basic protein (MBP) gene encodes two families of proteins, the classic MBP constituents of myelin and the golli-MBPs, the function of which is less well understood [229]. Although golli-Mbp null mutant mice did not display an overt dysmyelinating phenotype, they did exhibit delayed and/or hypomyelination in the visual cortex and the optic nerve. Ultrastructural analysis of the mutants confirmed both the delay and hypomyelination and revealed abnormalities in myelin structure and in some oligodendrocytes.

**12) *Mobp* (Myelin-Associated Oligodendrocytic Basic Protein)**

**ABA review:** *Mobp* displayed strong staining of oligodendroglia in the cerebellar white matter as well other white matter tracts of the brain. However, significant *Mobp* expression could also be seen many other regions of the brain.

Sagittal section

**GENSAT:**  *Mobp* is expressed exclusively in oligodendrocytes in the central nervous system. The BAC data matches the literature and BGEM *in situ* hybridization data. The BAC correctly expresses in oligodendrocytes.

**Literature:** In the postnatal central nervous system of the mouse, *Mobp* mRNA is located initially in the cell bodies of oligodendrocytes, but moves distally into their processes as myelination proceeds [230]. Mice homozygous for one null allele show abnormal myelin arrangements, in that while the formation of compact myelin was normal, the radial component was abnormally straight (instead of zig-zag as in wild type) [231]. Mice homozygous for another null allele contain normal myelin [232].

**13) *Mog* (Myelin-Oligodendrocyte Glycoprotein Precursor)**

**ABA review:** Moderate-to-high expression in the white matter tracts. *Mog* was also found at significant levels throughout the brain. At higher magnification expression was specific to oligodendroglia.

Sagittal section Sagittal section (zoomed)

**GENSAT:** No information available.

**Literature:** *In vitro* footprinting and electrophoretic mobility shift assays demonstrated that a 657 bp of the 5'-flanking sequence of the murine *Mog* gene induces the highest level of transcription in an oligodendroglial cell line [233].

**14) *Olig2* (Oligodendrocyte transcription factor 2)**

**ABA review:** *Olig2* was highly expressed in the white matter tracts. However, significant background staining could also be seen throughout the brain.

Sagittal section Coronal section Coronal (zoomed)

**GENSAT:** In the adult mouse moderate-to-strong expression is found in 18 brain regions: amygdala, anterior olfactory nucleus, basal forebrain, caudate putamen, cerebellum, cerebral cortex, dorsal horn, hippocampus, hypothalamus, medulla, midbrain, olfactory bulb, pons, septum, substantia nigra, thalamus, ventral horn, and ventral striatum. The BAC data is reproducible and matches both the literature and BGEM *in situ* hybridization data. The EGFP transgene expresses in small glial cells throughout the central nervous system especially concentrated in white matter tract.

**Literature:** In the post-natal brain, *Olig2*-expressing cells are present preferentially in white matter such as corpus callosum and cerebellar medulla and spread out as they mature [234].

**15) *Pllp* (Plasmolipin)**

**ABA review:** High levels of *Pllp* expression were present throughout the brain. Slightly higher levels of staining could also be seen in the hippocampus. *Pllp* expression did not appear to be significantly enriched within white matter tracts.

Sagittal section Coronal section Coronal - zoom

**GENSAT:** No information available.

**Literature:** Plasmolipin is to be restricted to oligodendrocytes of the rat brain [235].

**16) *Plp1* (Proteolipid Protein 1)**

**ABA review:** High levels of expression were found in the in the white matter tracts throughout the brain. However, there was significant background staining present throughout the brain. In particular, very levels of labeling could be found throughout the main olfactory bulb, brain stem and the granular cell layer of the cerebellum.

Sagittal section Coronal section Coronal (zoomed)

**GENSAT:** No information available.

**Literature:** Microarray analysis revealed enrichment of *Plp* in the heavily myelinated pyramidal tracts and other ventral myelinated pathways surrounding dopaminergic and GABAergic cell bodies of the adult rat substantia nigra [15]. Two proteolipid proteins, *Plp* and *Dm20*, are major membrane components of central nervous system myelin [236].

**17) *Sox10* (SRY-Box Containing Gene 10)**

**ABA review:** Low levels of expression were present within the cerebellar white matter. There did not seem to be much expression in the corpus callosum nor the corticospinal tract. However, there was also relatively high expression in the Purkinje cells of the cerebellum, hippocampal pyramidal cells and cortical neurons. These findings suggested that *Sox10* may be expressed in both oligodendroglia and neurons.

Sagittal section Sagittal (zoomed) Coronal section

**GENSAT:** In the adult mouse moderate-to-strong expression is found in 15 brain regions: amygdala, caudate putamen, cerebellum, cerebral cortex, dorsal horn, hippocampus, hypothalamus, medulla, midbrain, olfactory bulb, pons, septum, thalamus, ventral horn, and ventral striatum. Two BAC lines have identical expression at P7. The BAC data is consistent with the literature. The expression of the EGFP reporter gene is only detected in glial cells.

**Literature:** In the adult rat brain, *Sox10* transcripts are confined to glial precursors and later in oligodendrocytes [237].

**18) *Tmem63a* (Transmembrane Protein 63a)**

**ABA review:** Relatively high expression was observed in the white matter of the cerebellum including the arbor vitae and throughout the cerebellar peduncles. There was also relatively high expression in the rest of the brain including the corpus callosum, and corticospinal tract. In an independent review of the ABA images it was noted that *Tmem63a* expression was fairly strong in all areas where there are accumulations of nerve fibers.

Sagittal section Sagittal section (zoomed)

**GENSAT:** No information available.

**Literature:** SAGE analysis revealed regional enrichment of *Tmem63a* in the substantia nigra of the adult C57BL/6 mouse [10].

**Subanatomical region: HiPPOcampus, Therapeutic interest: Alzheimer Disease, Adult Neurogenesis, Depression, Plasticity**

The term hippocampal formation generally applies to the dentate gyrus, the Cornu Ammonis fields CA1-CA3 (and CA4, frequently called the hilus and considered part of the dentate gyrus), and the subiculum. The CA1, CA2 and CA3 fields make up the hippocampus proper. The hippocampus forms part of the limbic system and plays a role in memory formation. Additionally, the hippocampus dentate gyrus is also the site of neurogenesis in adults. Abnormalities of the hippocampus cause epilepsy, Alzheimer disease, depression and schizophrenia.

1**.**  ***Htr1a* (5-Hydroxytryptamine 1A Receptor)**

**ABA review:** Within the hippocampal formation there was moderate-to-high expression in the pyramidal cell layer of the dentate gyrus crest. Slightly lower levels of expression were present in the granule cell layer of the CA1, CA2, and CA3 fields. Layer 2 of the entorhinal area contained high levels of *Htr1a* labeling. Within the pallidum moderate expression was observed in the diagonal band nucleus and in the medial septal nucleus. Moderate expression was present in the interfascicular nucleus raphe, superior central nucleus raphe, medial part and the interpeduncular nucleus. Low diffuse staining could be seen in layers 2/3 and 4 of the cerebral cortex, and layers 1 and 2 of the olfactory tubercule.

Sagittal section Coronal section Coronal (zoomed)

**GENSAT:** BAC data is consistent with the literature and BGEM in situ hybridization data in general. In situ data shows stronger expression in upper layers of the visual cortex at P7, which is not obvious in the BAC data. In the P7 cerebellum, *Htr1a* mRNA is transiently expressed in cells near the Purkinje cell layer. The BAC produces expression in interneurons in the internal granule layer. In the adult, scattered cells are labeled in dentate gyrus in BAC transgenic mice.

**Literature:** In the rat brain, in situ hybridization detected highest expression in the dorsal raphe nucleus, septum, hippocampus, entorhinal cortex, and interpeduncular nucleus [238]. Other areas include olfactory bulb, cerebral cortex, some thalamic and hypothalamic nuclei, brainstem nuclei, and the dorsal horn of the spinal cord. In another study, the 5-HT1A receptor transcript is expressed densely in the mouse E14.5-16.5 thalamus, in hippocampus, and in a medial-to-lateral gradient in the cortex [239]. Expression of the 5HT1A receptor emerges during the initial stages of embryonic hippocampal development. Most, if not all, hippocampal neurons begin to express 5HT1A shortly upon completion of their terminal mitosis [240].

**2. *Tgfb2* (Transforming Growth Factor, Beta 2)**

**ABA review:** Moderate-to-high expression in the dentate gyrus and high levels of expression in CA2 and the CA3 (stratum lucid) regions were observed. Cells lining the third, fourth and lateral ventricles also express *Tgfb2* at moderate-to-high levels. Low-to-moderate levels of staining were present in the anterior and posterior segments of the biomedical amygdalar nucleus. There was also moderate expression in the cerebellar Purkinje cell layer.

An independent review indicated very nice label in all of regio inferior pyramidal cells and light label in dentate granule cells. Additional label was found in the Isle of Calleja, rostromedial thalamus, choroid plexus, and discrete nuclei in amygdala. Neurons in mamillary nucleus in hypothalamus and Purkinje cells were also labeled.

Sagittal section Sagittal (zoomed)

**GENSAT:** Two BAC transgenic lines have very similar expression patterns at P7. The Bac data is correct but very incomplete. The confirmed expression sites are choroid plexus, epedymal cells lining the lateral ventricles and brainstem. In situ hybridization data also shows localization of *Tgfb2* mRNA in the thalamus, hippocampus (CA3 and DG) and amygadala. Better BACs will be chosen and the gene will be retargeted.

**Literature:** Tgfb2 is an essential signal for differentiation of midbrain progenitors toward neuronal fate and dopaminergic phenotype [241]. Microarray analysis revealed *Tgfb2* is regionally enriched in the hippocampus [11]. *Tgfb2* is expressed principally by radial glia and astrocytes in developing rats; in the adult, it is expressed in glia and neurons [242].

**3. *Gria1* (Glutamate Receptor, Ionotropic, AMPA 1)**

**ABA review:** Moderate expression was found in the glomerular layer of the main olfactory bulb. Similar levels of staining could be seen in 2 and 3 layers of the dorsal/ventral taenia tecta and the medial part of the anterior olfactory nucleus. Moderate *Gria1* labeling was present in the lateral septal complex. In the hippocampal formation there was very high expression in the CA1 and CA2 pyramidal cell layer whereas moderate-to-high expression could be seen in the CA3 field. In addition, there was moderate-to-high staining along the granular cell layer of the dentate gyrus. Finally, low-to-moderate expression could be found in the cerebellar Purkinje cell layer.

Coronal section Coronal (zoomed)

**GENSAT:** No information available

**Literature:** This gene is also called *Glur1* or *GluRA*. Strong *Glur1* immunostaining was found in all CA1 except the stratum pyramidale [243]. In area CA3, *Glur1* immunoreactivity was weaker in the strata oriens and radiatum than in the CA1, with the stratum lucidum showing the weakest labelling. GluR1-immunopositive products were also found in the soma, dendrites and dentritic spine of neurons in the hilus of the dentate gyrus and in the molecular layer of the dentate gyrus. A human cDNA clone detected an RNA transcript in human cerebral cortex, hippocampus and cerebellum, similar to that seen in rat [244]. In situ hybridization experiments showed that human *Glur1* mRNA is present in granule and pyramidal cells in the hippocampal formation and that there is no apparent difference of distribution between control patient and patient with Alzheimer disease. In hippocampal CA1 pyramidal neurons, *GluRA* (-/-) mice showed a reduction in functional AMPA receptors, with the remaining receptors preferentially targeted to synapses [245]. In adult *GluRA*-/- mice, associative long-term potentiation (LTP) is absent in CA3 to CA1 synapses, but spatial learning in the water maze was not impaired. The results suggest that CA1 hippocampal LTP is controlled by the number or subunit composition of AMPA receptors and show a dichotomy between LTP in CA1 and acquisition of spatial memory.

**4. *Nr3c2* (Nuclear Receptor Subfamily 3, Group C, Member 2)**

**ABA review:** Expression was found only in the hippocampal region. There was also moderate expression in the induseum griseum of the dentate gyrus. In addition, there was moderate-to-high expression along the granular cell layer of the dentate gyrus. Slightly lower levels of staining were observed in the pyramidal cells of Ammon’s horn. A small population is this region displayed very strong labeling for *Nr3c2*.

Sagittal section Coronal section Coronal (zoomed)

**GENSAT:** No information available

**Literature:** In the rat hippocampus, neurons in CA1, CA2, and the dentate gyrus express *Nr3c2* at high levels; mRNA was found in all pyramidal cell fields (CA1-4) of the hippocampal formation and the granular neurons of the dentate gyrus [246]. Microarray analysis revealed *Nr3c2* is regionally enriched in the adult rat hippocampus [9]. The gene is also called MR**,** and expression of rat MR is regulated by at least three promoters, and at least three mRNA subtypes differing in their 5’-untranslated regions have been demonstrated [247, 248]. In human post-mortem brain, MR mRNA was also very highly expressed in hippocampus, with significantly higher levels in dentate gyrus and CA2, CA3 and CA4 than CA1 [249].

**Subanatomical region: HiPPOcampus, Ammon’s horn. Therapeutic interest: Alzheimer Disease, Adult Neurogenesis, Depression, Plasticity**

(See description of Hippocampus under Subanatomical region: Hippocampus)

**1. *Fibcd1*** **(Fibrinogen C Domain Containing 1)**

**ABA review:** Cells that were labeled include larger cells in the olfactory bulb glomeruli, cells of the taenia tecta and many cells in cortex and septal nucleus. Additionally, robustly labeled cells could be found in the lateral preoptic area, amygdala and regio superior pyramidal cells. An independent review of the ABA images indicated very high expression in hippocampus CA1 and CA2. Some low and sparse expression could also be seen in the dentate gyrus. In the retrohippocampal region, there was moderate-to-high expression in layer 6 of the lateral portion of the entorhinal area. Moderate expression could be seen in the glomerular layer of the main olfactory bulb, in the 3rd ventricle, and throughout the cerebral cortex. However, expression density was somewhat higher in the ventral and dorsal taenia tecta and throughout layer 1. In the cortical subplate, slightly higher levels of expression are found in the posterior amygdalar nucleus. In the pons, the laterodorsal tegmental nucleus and sublaterodorsal nucleus appeared to have moderate punctuate expression. In the medulla, similar levels of labeling are also present in the dorsal motor nucleus of the vagus nerve.

Sagittal section Coronal section Coronal (zoomed)

**GENSAT:** No information available

**Literature:** No brain expression studies have been reported.

**2. *Neurod6* (Neurogenic Differentiation 6)**

**ABA review:** Within the hippocampal formation, there was very intense expression throughout the pyramidal cell layer. There was moderate, but very significant, expression in the cerebral cortex layer 1, 2, 3, and 4, and similar levels of expression in the posteroventral part of the anterior olfactory nucleus in the main olfactory bulb. An independent review of the ABA images indicated that there was expression in all of dorsal cortex (deep layers, in particular), the cells of the basolateral amygdala, all of the hippocampal pyramidal cells, and the polymorphic cells.

Coronal section Coronal (zoomed)

**GENSAT**: Twenty-two regions of moderate-to-strong expression are described for the adult. BAC data is reproducible in multiple lines and matches the literature.

**Literature:** *Neurod6* is also called *Nex1*. Microarray analysis revealed *Neurod6* is regionally enriched in the adult mouse hippocampus [11]. SAGE analysis revealed regional enrichment of *Neurod6* in the prelimbic cortex of adult C57BL/6 mice [10].

Rodent *Nex1* gene induction coincides with the generation of post-mitotic neurons and parallels overt neuronal differentiation and synaptogenesis. High levels of mRNA are sustained in mature pyramidal neurons of the hippocampus, cerebellum and several neocortical areas. When ectopically expressed in PC12 cells, Nex1 trans-activates its own promoter [250]. Nex1 is a critical effector of the NGF pathway and promotes neuronal differentiation. In another study, it was shown that a subset of progenitor cells within the subventricular zone of the mouse neocortex can be defined by Cre recombinase expression under control of the *Nex* locus [251]. Nex-deficient mice have no obvious developmental defect, and central nervous system neurons appear fully differentiated. It has been suggested that Nex gene function in neuronal differentiation in mutant mice is compensated for by neuroD and NDRF [252].

**3. *Pkp2* (Plakophilin 2)**

**ABA review:** One review of the ABA images indicated that there was nice discrete label in all hippocampal pyramidal cells, with a lighter signal in most granule cells and thalamic and inferior olive neurons with nothing else labeled. In an independent, it was found that the entire hippocampal pyramidal cells displayed moderate-to-high expression. However, the stratum lucidum region of CA3 had slightly higher levels of labeling. In addition, in the thalamus, there was low-to-moderate expression in the dorsal part of the lateral geniculate complex.

Sagittal section Coronal section Coronal (zoomed)

**GENSAT:** No information available

**Literature:** Mutations of plakophilin-2 (*Pkp2*) have been associated with the cardiac abnormality - arrhythmogenic right ventricular cardiomyopathy [253]. Null mice exhibit lethal alterations in heart morphogenesis and stability at mid-gestation [254]. No brain expression studies have been reported.

**4. *Slc9a2* (Solute Carrier Family 9, Member 2)**

**ABA review:** Within the hippocampal formation there was moderate-to-high expression throughout the pyramidal cell layer. In the main olfactory bulb there was moderate expression in the mitral layer. In sagittal sections there appeared to be moderate expression throughout the cerebral cortex and the cerebellum. An independent assessment indicated that the label is very similar to *Pkp2* but not as discrete and in many nuclei/regions in the brain.

Sagittal section Coronal section Coronal (zoomed)

**GENSAT:** No information available

**Literature**: This gene is also called *Nhe2*. Northern analysis of mouse tissues did not detect any expression in the brain [255]. No expression was detected in the human brain either [256].

**5. *Gpr161* (G Protein-Coupled Receptor 161)**

**ABA review:** Very intense labeling was observed in CA1 and CA2 within the hippocampal formation and throughout the length of the subiculum. In the main olfactory bulb there was moderate expression in the mitral layer and to a lesser extent also in the glomerular layer. Like *Fibcd1*, there appeared to be moderate expression in the ventral and dorsal taenia tecta. However, there was significantly less expression in the rest of the cerebral cortex. In the cerebellum moderate expression was observed in the Purkinje cell layer. There appeared to be moderate expression in the compact part of the substantia nigra.

An independent review indicated that the hippocampal regio superior pyramids were very strongly labeled; CA3a was less dramatically labeled and the rest of the pyramidal cells, and some cells in dentate, even less so. There was expression in the taenia tecta and layer V of dorsal cortex. Additionally, there was some label in some cerebellar Purkinje cells, but not much above background label elsewhere.

Sagittal section Coronal section Coronal (zoomed)

**GENSAT:** No information available

**Literature:** No brain expression studies have been described.

**6. *Arl15* (ADP-Ribosylation Factor-Like 15)**

**ABA review:** In the hippocampal formation, there was high expression in the pyramidal cell layer of the CA1 and CA2 fields. Moderate-to-high levels of expression could also be seen in the CA3 field. In addition, very sparse moderate expression was found in the cerebellar granular cell layer. In sagittal sections, there was moderate expression along the length of layer 2/3 of the cerebral cortex and low level staining was also observed in the caudoputamen.

Sagittal section Coronal section Coronal (zoomed)

**GENSAT:** No information available

**Literature:** No brain expression studies have been described.

**7. *C630041L24Rik* (Hypothetical Protein)**

**ABA review:** In the hippocampal formation, there was high expression in the pyramidal cell layer of the CA1 and CA2 fields. Moderate levels of staining were also present in the pyramidal layer of the ventral segment of the subiculum. Moderate-to-high levels of expression could be found in the induseum griseum. In addition, moderate labeling was observed in the anterodorsal part of the medial amygdalar nucleus and the posterior part of the basomedial amygdalar nucleus.

Sagittal section Coronal section Coronal (zoomed)

**GENSAT:** No information available

**Literature:** This gene is also known as *Spink8*. No brain expression studies have been described.

**8. *Hunk* (Hormonally Upregulated Neu-Associated Kinase)**

**ABA review:** Within the hippocampal formation there was high expression in CA1 while the level in the CA2 region was slightly lower. In the main olfactory bulb there was moderate labeling in the mitral layer and to a lesser extent also in the glomerular layer. There was significant low-to-moderate expression throughout the cerebral cortex with no obvious regional enrichment. Within the cerebral nuclei there was moderate expression in the rostroventral portion of the lateral septal nucleus. There was moderate expression in the cerebellar Purkinje cell layer.

An independent review indicated there are positive cells in the cortex and not much else except in the hippocampus where there was very nice label in the hippocampal. The strongest label was found in the regio superior pyramids and also in some inferior pyramids and the polymorphic neurons in the hilus.

Sagittal section Coronal section Coronal (zoomed)

**GENSAT:** No information available

**Literature**: This gene is also called *Mak-V*. Northern blot analysis detected transcript in the mouse midbrain, olfactory bulb, frontal, posterior and entorhinal cortex, with the highest level in the hippocampus and the cerebellum [257].

**9. *Klk8* (Kallikrein-Related Peptidase 8)**

**ABA review:** *Klk8* is expressed solely in the hippocampal formation. There was a moderate-to-high expression in CA1, CA2 and CA3. An independent review indicated that this gene is expressed very nicely and specifically in all of the hippocampal pyramidal cells with the exception of CA2 and CA4 (the polymorphic cells in the dentate hilus). Other sites of expression include the basomedial and lateral cells of the amygdala and some cell groups in the pontine region.

Sagittal section Coronal (zoomed)

**GENSAT:** No information available

**Literature:** Other aliases for this gene include brain serine protease 1, neuropsin and ovasin. Neuropsin has a functional role in neural plasticity [258]. Northern and in situ hybridization demonstrated that neuropsin mRNA is expressed specifically in the limbic system of adult mouse brain and is localized at highest concentration in pyramidal neurons of the hippocampal CA1-3 subfields [189]. Alternative splicing of the human transcript generates 2 isoforms; type 2 neuropsin is predominantly expressed in the adult brain and hippocampus [259]. Dot blot showed that neuropsin is expressed in various regions of adult brain, including the hippocampus and cerebral cortex and also in various fetal tissues. These results suggest that human type 2 neuropsin may be important to the adult brain plasticity. In neuropsin null mice, electron microscopic analysis revealed that a number of asymmetrical synapses were significantly decreased in the stratum radiatum, the major terminal field of Schaffer-collaterals, whereas free boutons still holding synaptic vesicles (but with no synaptic specialization) were increased [260]. An increased number of parvalbumin-immunoreactive cells (known as fast spiking cells) in mutant mice were also observed. These results strongly suggest that neuropsin is involved in connectivity of a group of CA1 synapses and consequently in the hippocampal networking. In another study it was shown that mutant mice are viable and overtly normal; they display normal hippocampal long-term synaptic potentiation (LTP) and exhibited no deficits in spatial navigation (water maze) [261]. Nevertheless, electrophysiological studies revealed that the hippocampus of such mice possessed an increased susceptibility for hyperexcitability (polyspiking) in response to repetitive afferent stimulation.

**10. *Sstr4* (Somatostatin Receptor 4)**

**ABA review**: In hippocampus, the strongest label is in regio superior pyramids but also label in region inferior to lesser extent; the dentate gyrus is unlabeled. The deep cortex (layers 5 and 6) is also labeled as are the subicular and entorhinal cortex. While the rostral forebrain has much label, the midbrain, hindbrain and cerebellum are largely unlabeled.

Sagittal section Coronal section Coronal (zoomed)

**GENSAT:** The published in situ hybridization and immunocytochemical data are not completely in agreement. The consensus is that the gene is strongly expressed in cortex (layer IV-VI), hippocampus (CA1-3). It is also expressed at lower levels in other areas, including anterior olfactory nucleus, septum and amygdala. The BAC is expressed in above regions at P7. However, the database line is a low copy line and the expression in BAC mice is quite weak. In cortex, the neurons in layer IV are labeled, instead of neurons in layer IV-VI as both literature and BGEM in situ indicated. At E15.5, both BAC data and in situ hybridization data show little expression of Sstr4 inside brain except that the in situ reveals weak expression in DRG. Moderate-to-strong expression was found in the amygdala, hippocampus and the subicular cortex of an adult mouse brain.

**Literature:** In situ hybridization indicated that somatostatin receptor subtype 4 is found mainly in the hippocampus CA1, CA2 and CA3 pyramidal cells and in the pyramidal cells in layers (IV-VI) of the cerebral cortex [262]. In the human brain, intermediate Sst4 receptor mRNA signals were observed in the dentate gyrus of the hippocampus and several medullary nuclei while an intense expression was found in the granule and Purkinje cell layer of cerebellum [263].

**Subanatomical region: HiPPOcampus, Dentate gyrus. Therapeutic interest: Alzheimer Disease, Adult Neurogenesis, Depression, Plasticity**

(See description of Hippocampus under Subanatomical region: Hippocampus)

**1. *Lct* (Lactase)**

**ABA review:**  One review of the ABA images indicated label in dentate granule cells and regio superior pyramidal cells, and almost no label anywhere else in areas surveyed. In an independent review, it was found that *Lct* is expressed at high levels along the length in the granule cell layer of the dentate gyrus. In addition, moderate-to-high levels of labeling could be observed in the CA1 and CA2 pyramidal cells; very little expression could be observed in other regions of the brain.

Sagittal section Coronal section Coronal (zoomed)

**GENSAT:** No information available

**Literature:** *Lct* is also called *Lph* (Lactase-phlorizin hydrolase). No brain expression studies have been reported.

**2. *Crlf1* (Cytokine Receptor-Like Factor 1)**

**ABA review:** Specific expression in descending levels was observed in: dentate granule cells, polymorphs in hilus, and CA3/regio inferior pyramidal cells. Additionally, the hippocampus-related structures of taenia tecta and induseum griseum are also labeled. There was very low expression elsewhere in the brain. An independent review found high expression along the length of the granule cell layer of the hippocampus dentate gyrus. The pyramidal cells displayed moderate-to-high expression in the CA2 and CA3 regions. The cerebral cortex contained moderate labels in the ventral taenia tecta.

Sagittal section Coronal section Coronal (zoomed)

**GENSAT:** No information available

**Literature:** Microarray analysis revealed *Crlf1* is regionally enriched in the adult mouse hippocampus [11].

**3. *Tdo2* (Tryptophan 2,3-dioxygenase)**

**ABA review:** There was nice label in many, but not all, dentate granule cells; some sporadic layer V cortical pyramidal cells and cerebellar Purkinje cells are also labeled, but there was very little signal in the rest of brain. An independent review of the ABA images indicated that moderate-to-high expression along the length of the hippocampus dentate gyrus. In the cerebral cortex, there appeared to be moderate expression in the dorsal taenia tecta. The cerebellar pyramidal cell layer also displayed moderate labeling.

Sagittal section Coronal section Coronal (zoomed)

**Literature:** Tdo2 is expressed in the rat brainstem, cerebellum, cortex and hypothalamus [264]. Expression of the kynurenine pathway enzyme tryptophan 2,3 dioxygenase is increased in the frontal cortex of individuals with schizophrenia [265].

**4. *A330019N05Rik* (Hypothetical Protein)**

**ABA review:** In the hippocampal formation there was very high expression the granule cell layer of the dentate gyrus. In the Ammon’s there was moderate-to-high labeling in CA2 pyramidal cell layer; however, sparse moderate expression could also be observed in the CA1 and CA3 fields. In the main olfactory bulb there was moderate-to-high staining in the mitral layer and low-to-moderate expression in the glomerular layer. In the motor-related pons there were moderate levels of expression in the supratrigeminal nucleus; slightly lower amount of labeling in the hypoglossal nucleus of the motor-related region of the medulla.

Sagittal section Coronal section Coronal section (zoomed)

**GENSAT:** No information available

**Literature:** No brain expression studies have been reported.

**5. *Gabrd* (Gamma-Aminobutyric Acid (GABA) A Receptor, Delta)**

**ABA review:** There was high and discrete expression in dentate granule cells and entorhinal cortex with almost no label elsewhere, with the exception of SVZ/RMS cells which appeared nicely labeled. An independent review indicated moderate-to-high expression (coronal sections) throughout the dentate gyrus. The rest of the brain displayed very little expression. However, in sagittal sections, the dentate gyrus showed very high levels of staining. There was very high expression in the layers 2 and 3 of the dorsal and ventral zones of the medial part of the entorhinal area. In addition, there was some sparse punctuate expression throughout the brain.

Sagittal section Coronal section Coronal (zoomed)

**GENSAT:** No information available

**Literature:** In the rat brain, mRNA levels are highest in the cerebellar granule cells and secondarily in hippocampus and thalamus [266]. A 6.4 kb genomic fragment of the *Gabrd* gene faithfully directed *lacZ* expression in the cerebral cortex, hippocampal formation, thalamus, and brainstem of transgenic mice [267]. This was consistent with the expression of the endogenous delta subunit protein in both developing and mature brain. Northern blot detected expression of the human gene in the cerebellum, cerebral cortex, medulla, temporal, frontal and occipital lobes, and putamen [268]. A *Gabrd* deletion in mice prevents neurosteroid modulation of inhibitory synaptic currents in cerebellar neurons [269]. The uncompetitive, glutamate receptor antagonist MK-801 reduces Gabrd mRNA levels in rat dentate gyrus, but not in other brain regions quantitated [270].

**6*. Prox1* (Homeobox Prospero-Like Protein PROX1)**

**ABA review:** Very high expression could be seen in the granule cell layer of the dentate gyrus. In addition, there was moderate expression throughout the thalamus. In the sagittal sections, there was also moderate expression throughout the brain particularly in the midbrain, brainstem and cerebellum.

Sagittal section Coronal section Coronal (zoomed)

**GENSAT:** No information available

**Literature:** Prenatally, *Prox1* is expressed in the subventricular zone or in early differentiating regions of the murine brain [271]. At these stages, Prox1 mRNA, but not Prox1 protein, is also detected in several regions of the prethalamus and hypothalamus. At an early postnatal stage, Prox1 expression is mainly detected in several nuclei of the thalamus, the cerebellum, and the hippocampus. In adulthood, *Prox1* expression remains only in the hippocampus dentate gyrus and cerebellum. The *Prox1* +/ *Prox1*::*lacZ* mice have an insertion of the *lacZ* gene into the *Prox1* locus, and *Prox1* promoter driven-galactosidase expression recapitulates the expression pattern of the endogenous *Prox1* gene [272].

**7*. Dsp* (Desmoplakin)**

**ABA review:** Very high expression was observed in the granule cell layer of the dentate gyrus. There was some low-to-moderate expression in the lateral ventricle, 4th ventricle and lateral recess in what appeared to be the choroid plexus.

Sagittal section Coronal section Coronal (zoomed)

**GENSAT:** Expression in the hippocampus is confirmed by in situ hybridization data from the Allen Brain Atlas. The BAC data also reveals expression in restricted nuclei in brainstem, which is reproducible among other BAC transgenic lines.

**Literature:** No brain expression studies have been reported.

**8*. C78409* (Expressed Sequence C78409)**

**ABA review:** There was very high expression in the granule cell layer of the dentate gyrus. Low-to-moderate labeling could also be seen in the caudoputamen. In some sagittal sections, there was moderate expression in the choroid plexus.

Sagittal section Coronal section Coronal (zoomed)

**GENSAT:** No information available

**Literature:** No brain related expression studies have been reported.

**9*. Lrrtm4* (Leucine Rich Repeat Transmembrane Neuronal 4)**

**ABA review:** Moderate-to-high expression could be observed throughout the hippocampus dentate gyrus. A relatively small number of cells in hippocampus CA2 expressed *Lrrtm4* at low-to-moderate levels. No other region in the brain showed obvious staining for this gene. An independent review indicate light-to-moderate, specific label in the dentate granule cells, CA2, and anterior olfactory nuclei.

Sagittal section Sagittal (zoomed)

**GENSAT:** No information available

**Literature:** Human *LRRTM4* mRNA is prominently expressed in the cerebellum, spinal cord, amygdala, caudate nucleus, corpus callosum, hippocampus and thalamus. In the adult mouse brain, *LRRTM4* is expressed in the olfactory bulb, olfactory tubercle, cerebral cortex, striatum, and inferior colliculi. In the hippocampus, high expression was seen in the granular layer of dentate gyrus and CA3 pyramidal layer. Low levels are found in Purkinje cell layer and brainstem regions [273].

**10. *Htr4* (5-Hydroxytryptamine (Serotonin) Receptor 1A)**

**ABA review:** The islands of Calleja and associated structures like olfactory tubercle and the ventral pallidum were nicely labeled. Sporadic cells in striatum labeled and there was about the same pattern in the dentate gyrus.

Sagittal section Coronal section Coronal (zoomed)

**GENSAT:** There are two splice forms of Htr4 receptors with slightly different regional distributions in the central nervous system. According to literature, both splice forms are present in striatum, colliculi, hippocampus, and substantia nigra; one of the splice forms is also found in cerebellum and the other in the thalamus, main olfactory bulb and brainstem. Two BAC transgenic lines have matching expression at P7. Almost all the sites mentioned by the literature are seen in the BAC transgenic mice. In addition, the BAC reveals staining in cortex and hypothalamus, which are confirmed by in situ. There are 25 regions of strong to moderate expression in the adult: amygdala, anterior olfactory nucleus, basal forebrain, caudate putamen, cerebellum, cerebral cortex, entorhinal cortex, fasciculus retroflexus, globus pallidus, hippocampus, hypothalamus, medulla, midbrain, nigrostriatal bundle, olfactory bulb, piriform cortex, pons, septum, spinal cord dorsal horn, spinal cord ventral horn, stria terminalis, subicular cortex, substantia nigra, thalamus, and ventral striatum.

**Literature:** This gene is also called *5-HT4*. Oligonucleotide probes that recognize two cloned splice variants (*5-HT4S* and *5-HT4L*) of 5-HT4 receptors used in situ hybridization revealed high levels of transcripts in olfactory tubercle, some components of the basal ganglia (caudate putamen, ventral striatum), medial habenula and hippocampal formation [274]. In sections of post-mortem human brain, in situ hybridization indicated the highest levels of *5-HT4* receptor mRNA in caudate nucleus, putamen, nucleus accumbens, and in the hippocampal formation [275].

**11. *Tspan18* (Tetraspanin 18)**

**ABA review:** There was strong label in regio inferior pyramidal cells and dentate granule cells (deeper ones seem to be labeled a bit stronger than superficial ones), but otherwise everywhere else there was low-to-no signal. There was very strong signal also in the habenula and nice label in inferior olivary neurons.

An independent review indicated strongest region of expression is not the hippocampus but a region centrally located between the hippocampus wings. Expression is mainly moderate in the dentate gyrus and CA. There also is some off-target expression in olfactory region.

Sagittal section Coronal section Coronal (zoomed)

**GENSAT:** No information available

**Literature:** No brain expression studies have been reported.

**Subanatomical region: Hypothalamus, Therapeutic Interest: Cancer**

The hypothalamus is located below the thalamus and adjacent to the mammillary bodies, the third ventricle, and the optic chiasm. The hypothalamus plays an essential role in regulating the autonomic nervous system. In addition, the paraventricular and supraoptic nuclei of the hypothalamus project large neurons to the posterior pituitary gland and thereby link the nervous system to the endocrine system [74]. Through its autonomic and endocrine effector pathways the hypothalamus modulates fluid and electrolyte balance, food ingestion and energy balance, reproduction, thermoregulation, and immune activity, and responsiveness to pain stimuli [276]. Hypothalamic dysfunctions are associated with stress/ mood disorders [277], sleep disturbances [278], and headaches [276].

**1) *Hcrt*** **(Hypocretin)**

**ABA review:** Very high expression was present in the lateral hypothalamic area. Moderate staining was also present in layer 2 of the cerebral cortex. In the hippocampal formation, there was moderate staining in the pyramidal cell layer of Ammon’s horn and the granule cell layer of the dentate gyrus. Moderate-to-high expression was also observed in the cerebellar Purkinje cell layer. It should be noted that there was significant background staining in the slides. In an independent review of the ABA images, it was reported that there was very specific *Hcrt* staining in the anterior/posterior regions of the hypothalamus. In addition, there was also labeling of cells in the superficial layer and molecular region of piriform cortex.

Sagittal section Coronal section

**GENSAT:** In the adult mouse strong to moderate expression is found in 3 brain regions: basal forebrain, cerebral cortex, and hypothalamus. The adult BAC data correlate well with published expression data and BGEM *in situ* hybridization data. However, *in situ* data shows the presence of *Hcrt* mRNA in hypothalamus at P7, which is not observed in P7 BAC mice.

**Literature:** No brain expression studies have been reported.

**2) *Gpx3*** **(Glutathione Peroxidase 3)**

**ABA review:** High levels of expression were observed in the paraventricular hypothalamic nucleus. In sagittal sections similar levels of staining could be seen in the arcuate nucleus. Moderate levels of labeling were present in the medial preoptic nucleus. Low-to-moderate expression could be found throughout the rest of the hypothalamus. In the thalamus, moderate staining was present in the paraventricular nucleus of the thalamus. Within the midbrain, the dorsal nucleus raphe displayed moderate-to-high labeling. In the pons, there was high expression in the locus coeruleus. In addition, there are some moderately labeled cells in the dorsal part of the nucleus of the lateral lemniscus and the lateral division of the parabrachial nucleus. In the medulla, there was high expression in the area postrema but moderate levels of labeling could be seen in the central and medial portions of the nucleus of the solitary tract.

In an independent review of the ABA images it was noted that there was nice labeling in several hypothalamic regions including the mamillary, arcuate, ventromedial and dorsomedial nuclei as well as the preoptic and anterior hypothalamic regions. There was also labeling in the paraventricular nucleus of the thalamus. Finally, there was some staining of cells in the locus coeruleus region and parts of the substantia nigra.

Sagittal section Coronal section

**GENSAT:** No information available.

**Literature:** Microarray analysis revealed *Gpx3* is regionally enriched in the adult mouse hypothalamus [11].

**3) *Trh* (Thyrotropin Releasing Hormone)**

**ABA review:** High level of expression was present in the periaqueductal gray. In addition, there was very high staining in the cortical amygdalar area, posterior part, medial zone and in the ventral zone of the medial part of the entorhinal area. There was also moderate-to-high labeling within the hypothalamus and in the inferior olivary complex region of the medulla. In an independent review of the ABA images, it was reported there was nice staining of neurons in hypothalamus including the periventricular, paraventricular, ventromedial nuclei as well as the medial and posterior segments of the arcuate nucleus. The reticular nucleus of the thalamus and subfornical organ were also well-labeled. In addition, there was expression of *Trh* in the medial/ventral amygdaloid region and in layer 6 of the cerebral cortex. In particular, there was significant expression in the posteromedial cortical amygdaloid nucleus. Finally, there was intense labeling of ventral posterior cells of the raphe (obscurus, pallidus) and the olfactory bulb glomeruli.

Sagittal section Coronal section

**GENSAT:** In the adult mouse brain there were no regions with strong to moderate expression. Two BAC lines have almost identical expression at P7. The BAC data is correct but incomplete. Compared with the adult *in situ* hybridization data from ABA, the BAC does not produce expression in posteromedial amygadaloid nuclei and reticular thalamic nuclei.

**Literature:** In the human brain, immunocytochemistry detected expression in the dorsocaudal paraventricular nucleus (PVN), suprachiasmatic nucleus (SCN), and in the sexually dimorphic nucleus (SDN) [279]. Dense *TRH*-containing fiber networks were present not only in the median eminence, but also in a number of other hypothalamic areas, suggesting a physiological function of *TRH* as a neuromodulator or neurotransmitter in the human brain, in addition to its neuroendocrine role in pituitary secretion of thyroid-stimulating hormone. *In situ* hybridization detected heavily labeled neurons in the caudal part of the PVN, while some cells were present in the SCN and in the perifornical area. PCR detected characteristic expression of the *Trh* mRNA solely in the mouse hypothalamus [280].

**4) *Fezf1* (FEZ Family Zinc Finger 1)**

**ABA review:** *Fezf1* was discretely expressed in the ventromedial hypothalamic nucleus. It was also expressed in significant amounts in the glomerular layer of the main olfactory bulb. In an independent review of the ABA images, it was noted that there was discrete label within the ventromedial nucleus of the hypothalamus.

Sagittal section Coronal section

**GENSAT:** No information available.

**Literature:** No brain expression studies have been reported.

**5) *Agrp* (Agouti-Related Protein Precursor)**

**ABA review:** *Agrp* was found to be exclusively expressed in the arcuate hypothalamic nucleus. Very little to no expression was present in other brain regions. An independent review of the ABA images confirmed that there was very nice and specific label for *Agrp* in the arcuate nucleus with no expression elsewhere in the brain.

Sagittal section Coronal section

**GENSAT:** No information available.

**Literature:** *Agrp* mRNA-expressing cells were limited to the arcuate nucleus, representing a major subpopulation (95%) of the neuropeptide-Y (Npy) neurons in mice [281]. Additionally, Agrp-immunoreactive terminals all contained Npy and were observed in many brain regions extending from the rostral telencephalon to the pons, including the parabrachial nucleus.

**6) *Calcr* (Calcitonin Receptor Precursor)**

**ABA review:** Moderate-to-high expression was present throughout the hypothalamus. However, staining seemed to be somewhat higher in the paraventricular and supreachiasmatic nuclei. Similar levels of labeling were found in the posterodorsal region of the medial amygdalar nucleus. Moderate-to-high expression was seen in the dorsal raphe and the periaqueductal gray regions of the midbrain. In the pons there was high expression in the pontine central gray and in the laterodorsal tegmental, subceruleus and sublaterodorsal nuclei. In another set of observation it was noted that there was nice labeling of *Calc* in several hypothalamic regions including: arcuate, dorsomedial, anterior and medial preoptic areas.

Sagittal section Coronal section

**GENSAT:** No information available.

**Literature:** The mouse gene is also called *Clr* or *mCTR*. In the mouse brain, *in situ* hybridizations detected *Calcr* expression in the preoptic area, dorsomedial hypothalamic nucleus, lateral hypothalamic area, periaqueductal gray, dorsal raphe nucleus, locus coeruleus, lateral parabrachial nucleus, gigantocellular reticular nucleus alpha part, lateral paragigantocellular nucleus, raphe magnus nucleus and solitary tract nucleus [282]. Microarray analysis revealed *Calcr* is regionally enriched in the adult mouse hypothalamus [11]. In the human brain, specific calcitonin (CT) binding was measured with ^125^I-labeled CT appeared highest in homogenates of the posterior hypothalamus and the median eminence, and was less than 12% of hypothalamic binding in all of the other regions of the brain examined [283]. Like the human gene, mouse *Calcr* is imprinted in a tissue-specific manner, with a predominant expression from the maternal allele in the brain [284].

**7) *Ghrh* (Growth Hormone-Releasing Factor)**

**ABA review:** In the hypothalamus there was moderate expression in the arcuate nucleus. In addition, there was also moderate staining in a hypothalamic region adjacent to the nucleus of reunions of the thalamus. No expression was observed in other areas of the brain. It should be noted that the sagittal sections did not show any convincing staining. In an independent review of the ABA images, it was noted that was very light and discrete label in the arcuate nucleus and medial tuberal region of the hypothalamus.

Coronal section

**GENSAT:** No information available.

**Literature:** In the mouse brain, RNA blot analysis detected *Ghrh* expression in hypothalamus and in situ hybridization indicated that *Ghrh* mRNA is localized predominantly to the arcuate nucleus of the hypothalamus [285]. Microarray analysis revealed *Ghrh* is regionally enriched in the adult mouse hypothalamus [11]. *Ghrh* mRNA in the hypothalamus of the adult male rat is increased by testosterone [286]. *Ghrh* null mice display isolated growth hormone deficiency, pituitary hyperplasia, growth retardation and weight loss [287].

**8) *Npy* (Neuropeptide Y Precursor)**

**ABA review:** In the hypothalamus there was very strong labeling of the cells in the arcuate nucleus. In the main olfactory bulb there was moderate-to-high expression in the olfactory nerve layer as well as moderate punctate staining in the granular layer. Finally, moderate-to-high punctate staining was observed throughout the cerebal cortex. In an independent review of the ABA images, it was reported *Npy* label in scattered interneuronal-like cells within the cortex and striatum. Very strong staining was seen in the suprachiasmatic region of hypothalamus and in the reticular nucleus of thalamus.

Sagittal section Coronal section

**GENSAT:** In the adult mouse strong to moderate expression is found in 20 brain regions: amygdala, anterior olfactory nucleus, basal forebrain, caudate putamen, cerebellum, cerebral cortex, entorhinal cortex, hippocampus, hypothalamus, mammillothalamic tract, medulla, midbrain, olfactory bulb, olfactory nerve layer, piriform cortex, pons, stria terminalis, substantia nigra, ventral horn, and ventral striatum. The BAC data is consistent with previously published results. Confirmed expression is found in cerebral cortex, hippocampus, hypothalamus, caudate putamen, basal forebrain and midbrain. It should be noted that fewer striatal interneurons are labeled in the BAC data. In addition, the BAC detects extra expression in cerebellum; because tau-EGFP is used as a reporter processes are also labeled, making it hard to distinguish positive cells from stained fibers in adult BAC mice.

**Literature:** In the rat and mouse brain, *in situ* hybridizations detected a strong signal in the arcuate nucleus of the hypothalamus [288]. Scattered *Npy*-mRNA-containing cell bodies were seen throughout the cerebral cortex. Immunohistochemistry detected a similar distribution of Npy peptide.  *NPY* mRNA is weakly expressed in the caudate nucleus, putamen and nucleus accumbens in normal individuals with a scattered labelling of neurons [289]. Increased *NPY* mRNA expression is observed in the striatum in Parkinson disease.

**9) *Pmch* (Pro-Melanin-Concentrating Hormone)**

**ABA review:** High and specific expression was observed in the lateral hypothalamic area. There did not appear to be much expression in other regions of the brain. In an independent review of the ABA images, it was noted that a set of neurons are labeled in ventromedial and lateral hypothalamus. *Pmch* label was not found in any other brain regions.

Sagittal section Coronal section

**GENSAT:** In the adult mouse strong to moderate expression is found in 19 brain regions: amygdala, anterior olfactory nucleus, basal forebrain, caudate putamen, cerebral cortex, entorhinal cortex, globus pallidus, hippocampus, hypothalamus, medulla, midbrain, olfactory bulb, piriform cortex, pons, septum, subicular cortex, substantia nigra, thalamus, and ventral striatum. The literature shows the *Mch* mRNA is prominently expressed in cells in the lateral hypothalamic area and zona incerta and the Mch peptide is localized in neurons in both areas. Fibers stained for Mch antibodies are distributed throughout the central nervous system in patterns that are conformed to known projection fields of the lateral hypothalamic area; BAC data is consistent with these findings. Because a tau::EGFP marker is used as a reporter, all the projection fields of the EGFP expressing cells are also nicely stained; distribution of the stained fibers is in agreement with the previously published data.

**Literature:** The rat *Pmch* mRNA encodes multiple putative neuropeptides coexpressed in the dorsolateral hypothalamus [290]. Northern blot analysis demonstrated that *Pmch* mRNA was found predominantly if not exclusively within the hypothalamus [291].

**10) *Pomc* (Pro-Opiomelanocortin Alpha)**

**ABA review:** Strong and specific expression was found in the arcuate hypothalamic nucleus; there did not appear to be much expression in other regions of the brain. In an independent review, it was noted that there was very specific staining in the arcuate nucleus and median eminence region of the hypothalamus.

Sagittal section Coronal section

**GENSAT:** In the adult mouse strong to moderate expression is found in 10 brain regions: amygdala, cerebellum, entorhinal cortex, hippocampus, hypothalamus, medulla, olfactory nerve layer, pons, ventral horn, and ventral striatum. The BAC expresses in medial basal hypothalamus, amygadala and brainstem, which are consistent with the published data. It also shows staining in hippocampus and cerebellum, which has not been previously reported for the endogenous *Pomc* mRNA.

**Literature:** In C57BL/6J mice, *Pomc* is specifically expressed in the hypothalamic arcuate nucleus [292]. Microarray analysis revealed *Pomc1* is regionally enriched in the adult mouse hypothalamus [11].

**Subanatomical region: Hypothalamus, Paraventricular nucleus, Therapeutic Interest: Pain**

(See hypothalamus for description)

**1) *Avp* (Arginine Vasopressin)**

**ABA review:** In the hypothalamus there was very high expression in the paraventricular hypothalamic nucleus. Similar levels of expression could also be found in the supraoptic nucleus. In the ventrolateral preoptic nucleus there was moderate labeling of cells. However, the most ventral portion of this region expressed *Avp* at very high levels. Somewhat lower levels of staining could also be observed in the anteroventral periventricular nucleus. Finally, in some slides there was inconsistent high expression in the optic chiasm.

Sagittal section Coronal section

**GENSAT:** In the adult mouse moderate-to-strong expression is found in 3 brain regions: hypothalamus, paraventricular nucleus, and supraoptic nucleus. The gene is expressed in hypothalamus, particularly in paraventricular nucleus, supraoptic nucleus and suprachiasmatic nucleus. The BAC data matches both literature and *in situ* hybridization data from ABA.

**Literature:** Microarray analysis revealed *Avp* is regionally enriched in the adult mouse hypothalamus [11]. In the mouse brain, *in situ* hybridization detected prominent groups of *Avp* mRNA expressing cells in the region between the paraventricular and suprachiasmatic nuclei, forming the distinct mouse accessory nucleus, and a periventricular group that merges with the paraventricular neurons [293]. In the supraoptic nucleus, a 60% increase of vasopressin mRNA expression was found in depressed compared with control subjects. In the melancholic subgroup, *Avp* mRNA expression was significantly increased in both the supraoptic nucleus and the paraventricular nucleus compared with control subjects (Meynen et al., 2006). In transgenic rats expressing an *Avp*-EGFP fusion gene, eGFP exp was observed in the hypothalamic supraoptic nucleus, the paraventricular nucleus, and the suprachiasmatic nucleus. Expression of eGFP in rats recapitulates that of the endogenous gene (Ueta et al., 2005).

**2) *Oxt* (Oxytocin-Neurophysin 1 Precursor)**

**ABA review:** In the hypothalamus there was very high expression in the paraventricular hypothalamic nucleus. Similar levels of expression could also be observed in the supraoptic, suprachiasmatic, and arcuate nuclei. Somewhat lower levels of expression were clearly present in the intermediate part of the periventricular hypothalamic nucleus. In an independent review of the ABA images, it was found that *Oxt* was very specific to the hypothalamus: supraoptic, suprachiasmatic and all aspects of the paraventricular nucleus, anterior hypothalamus.

Sagittal section Coronal section

**GENSAT:** No information available.

**Literature:** Microarray analysis revealed *Oxt* is regionally enriched in the adult mouse hypothalamus [11]. In humans, *Oxt* mRNA is found in the supraoptic nucleus, paraventricular nucleus, accessory magnocellular nucleus and, less frequently, in neurons of the lateral hypothalamus [294].

**Subanatomical region: Locus Coeruleus, Therapeutic Interest: Serotonin System, Depression**

Locus coeruleus (LC) is a compact nucleus of noradrenergic neurons located in the

dorsal pons that provides major ascending projections to the thalamus and cerebral cortex as well as descending outputs to the brainstem, cerebellum and spinal cord. LC plays an important role alertness and responsiveness to novel stimuli in the environment [20]. Alterations in the activity of the LC has been associated with a number of clinical disorders including attention-deficit hyperactivity disorder, stress disorders, and emotional and affective disorders [295].

**1) *Dbh* (Dopamine-β-Hydroxylase)**

**ABA review:** High levels of *Dbh* labeling were present in the locus coeruleus, the laterodorsal tegmental nucleus and the sublaterodorsal nucleus. Slightly lower levels were seen in the motor nucleus of trigeminal and supratrigeminal nucleus of the pons. Similar staining amounts were seen within the nucleus prepositus of the medulla.

Sagittal section Coronal section

**GENSAT:** No expression data is given for the adult mouse brain. However, it is reported that the BAC data shows correct expression in the locus coeruleus.

**Literature:** Dbh+ cells are found to be concentrated in the nucleus locus coeruleus and the nucleus subcoeruleus in the pons of the human brain stem [296]*.* *Dbh* immunoreactivity, visualized by the peroxidase-antiperoxidase method, is observed in the somata and proximal processes of locus coeruleus neurons and in the distal axons of several noradrenergic terminal fields in the rat central nervous system [297].

**2) *Maoa* (Monoamine Oxidase type A)**

**ABA review:** *Maoa* labeling was high and very specific to the locus coeruleus. There was very little staining in any other brain regions.

Sagittal section Coronal section

**GENSAT:** No information available.

**Literature:** In the rat brain, *Maoa* mRNA is present in major monoaminergic cell groups, such as the dorsal vagal complex, the C1/A1 groups, the locus ceruleus, the raphe nuclei, the substantia nigra, and the ventral tegmental area. *Maoa* mRNA is also found in forebrain structures, such as the cortex, the hippocampus, the thalamus, and the hypothalamus [298]. A spontaneous point mutation produced monoamine oxidase a/b knock-out mice with greatly elevated monoamines and anxiety-like behavior. The affected anatomical systems are homeostasis, behavior, nervous system, cardiovascular system [299]*.*  In addition, mice lacking *Maoa* have altered amounts of brain serotonin and norepinephrine and displayed aggressive behavior [300].

**3) *Slc6a2* (Solute carrier family 6 (neurotransmitter transporter, noradrenalin), member 2)**

**ABA review:** There was high and specific expression of *Slc6a2* in the locus coeruleus. Virtually no labeling was observed in other brain regions.

Sagittal section Coronal section

**GENSAT:** In the adult mouse strong to moderate expression is found in 3 brain regions: locus coeruleus, medulla, and pons. The BAC data is consistent with the literature. The locus coeruleus and laterodorsal tegmental nucleus are correctly labeled as well as their projections. The adult BAC data is also confirmed by the *in situ* hybridization data from ABA.

**Literature:** The gene is also called *Net*. *Slc6a2* is expressed mainly in the locus coeruleus of the rat brain stem, [301]. In the adult rat brain, all norepinephrine-containing cell bodies in the brainstem (locus coeruleus and lateral tegmentum) appear to express the *Net* mRNA [302]*.* Norepinephrine homeostasis is abnormal in homozygous mutant mice. In addition to displaying altered behavior, mutant mice are also hypersensitive to psychostimulants [303].

**4) *Slc18a2* (Solute carrier family 18 (vesicular monoamine), member)**

**ABA review:** High levels of expression of *Slc18a2* were present in the locus coeruleus. Similar levels of labeling were also observed in the ventral tegmental area and substantia nigra (pars compacta). Slightly lower levels of staining were observed in the dorsal raphe nucleus, and superior central nucleus raphe (medial part) significant and possibly the trochlear nucleus. Within the medulla, similar levels of expression were found in the laterodorsal tegmental nucleus, sublaterodorsal nucleus, nucleus raphe magnus and nucleus of the trapezoid body. Finally, scattered labeling was seen throughout the cerebral nuclei.

Sagittal section Coronal section

**GENSAT:** In the adult mouse moderate-to-strong expression is found in 18 brain regions: amygdala, basal forebrain, caudate putamen, cerebral cortex, entorhinal cortex, globus pallidus, hippocampus, hypothalamus, locus coeruleus, medulla, midbrain, olfactory bulb, piriform cortex, pons, septum, substantia nigra, ventral striatum, and ventral tegmental area. The BAC data shows correct expression in substantia nigra, ventral tegmental area, raphe nucleus and locus coeruleus. Expression in basal forebrain, pons and thalamus is also supported by the literature. Extra expression is detected in caudate putamen, nucleus accumbens and superior colliculus in BAC mice. However, the neuronal expression in these areas is only seen at E15-5 and P7 time points. In adult BAC mice, only labeled fibers are found in these areas and are probably projections from the substantia nigra, ventral tegmental area and optic nerve.

**Literature:** The gene is also called vesicular monoamine transporter 2 (*Vmat2*). In the adult rodent brain *Vmat2* expression is strongest in the locus coeruleus, substantia nigra and ventral tegmental area. Somewhat lower expression can also be seen in the hypothalamus and ventral thalamus [304]. Homozygous *Vmat2* null mice exhibit neonatal death and supersensitivity to cocaine and amphetamine [305]. In addition, mice with very low expression of the *Vmat2* gene may serve as a potential animal model for Parkinsonism [306]*.*

**Subanatomical region: Neurogenic regions, Therapeutic Interest: Adult Neurogenesis**

Neurogenesis is the process by which new neurons are generated. In adults, the majority of neurogenesis occurs in the subgranular zone of the dentate gyrus of hippocampus [307] and in the subgranular zone lining the lateral ventricles [308]. In addition, some neurogenesis has also been observed in the neocortex [309] and the substantia nigra [310]. It has been suggested that adult neurogenesis may be a mechanism for the replacement of lost neurons. It has also been demonstrated that neurogenesis plays a role in learning and memory [311].

**1) *Dcx* (Doublecortin)**

**ABA review:** High expression levels could be seen in the subependymal zone and rhinocele. Slightly lower levels of labeling were also present in the lateral, 3rd, 4th ventricles and in the rostral migratory stream. Moderate-to-high levels of expression were also found in the glomerular and granular cell layers of the main olfactory bulb. Within the hippocampal formation, there was moderate-to-high labeling of the granular cell of the dentate gyrus. In some slides there was sparse punctate staining throughout the brainstem and cerebellum. In an independent review, it was noted that *Dcx* labeled migrating neurons in the adult rostral migratory stream and subgranular zone of the hippocampus.

Sagittal section

**GENSAT:** In the adult mouse strong to moderate expression is found in 16 brain regions: amygdala, anterior olfactory nucleus, cerebellum, cerebral cortex, dorsal funiculus, dorsal horn, hippocampus, hypothalamus, lateral funiculus, midbrain, olfactory bulb, piriform cortex, rostral migratory stream, thalamus, ventral funiculus, and ventral horn. Two BAC lines share the same expression at adult. The BAC data matches the literature and BGEM *in situ* hybridization data. At adult, weak expression remains in most of the expression areas observed at P7 (probably due to the stability of EGFP protein), whereas BGEM data shows expression only in main olfactory bulb.

**Literature:** Expression of *Dcx* is high in certain areas of the adult mammalian brain, including the dentate gyrus and the lateral ventricle wall in conjunction with the rostral migratory stream and olfactory bulb [312]. In the adult rat brain, *Dcx* immunoreactive cells with the characteristic morphology of migrating neuroblasts can be found in the subventricular zone, rostral migratory stream and the main and accessory olfactory bulbs [313].

**2) *Mki67* (Antigen KI-67)**

**ABA review:** Moderate-to-high expression could be seen in the subependymal zone and rhinocele. Similar levels of labeling were also present in the lateral ventricle and in the rostral migratory stream. There was not much off-target expression of *Mk167*. However, in some slides low levels expression could be found throughout the cerebellum. In an independent review, *Mk167* staining was found to be very specific to the proliferative populations in rostral migratory stream and a small group of cells in the subgranular zone.

Sagittal section Coronal section

**GENSAT:** No information available.

**Literature:** Expression of the human *KI67* protein is strictly associated with cell proliferation. The KI67 protein is present during all active phases of the cell cycle, but absent from resting cells, suggests that it may be an excellent marker for proliferating cells [314]. *Mki67* is expressed exclusively in the pyknotic but not in the non-pyknotic nuclei in the hippocampus [315]. Immunohistochemistry for *Mki67* and 5-bromo-2-deoxyuridine (BrdU) labeling showed that cell proliferation occurred mainly in the hilus and partly in the subgranular zone of postnatal rat dentate gyrus [316].

**3) *Vim* (Vimentin)**

**ABA review:** Moderate-to-high expression of *Vim* was present in cells lining the lateral ventricle, third ventricle, and the lateral recess. Similar levels of labeling could also be seen in the Purkinje cell layer. Moderate levels of labeling were also observed in the olfactory nerve layer of main olfactory bulb. Low-to-moderate punctuate expression could also be found throughout the brain. In an independent review of the ABA images, it was that *Vim* was found to be specific for ependyma and pia (and blood vessels) as well as the adult neurogenic population. Limited staining was present elsewhere with the exception of very nice label in cells of the Purkinje cell layer in cerebellum.

Sagittal section Coronal section

**GENSAT:** In the adult mouse strong to moderate expression is found in 16 brain regions: amygdala, anterior olfactory nucleus, cerebellum, cerebral cortex, choroid plexus, dorsal horn, ependymal layer, hippocampus, leptomeninges, medulla, olfactory nerve layer, pons, rostral migratory stream, and ventral horn. The BAC expresses in both neurons and glia at P7. The cerebellar expression is correct. However, level of expression is greatly decreased in adult cerebellum. Only a subset of the Bergmann glial is labeled. The overall expression pattern in adult BAC mice is consistent with *in situ* hybridization data from ABA.

**Literature:** Entorhinal cortex lesioning increases *Vim* mRNA expression in the ipsilatera1 hippocampus, the ipsilateral cortex, and in the outer molecular layer of the dentate gyrus [317]. In unlesioned controls, *Vim* mRNA is detected in ependymal and endothelial cells but not in the hippocampal parenchyma indicating that there is expression of Vim in subpopulations of both microglia and astrocytes. In *Vim* null mice, reactive astrocytes that normally express both GFAP and vimentin do not exhibit GFAP immunoreactivity. GFAP fails to assemble into a filamentous network in the absence of *Vim* [318]. Therefore, Vim appears to be necessary for the stabilization of GFAP filaments and consequently network formation. In the granular layer of the dentate gyrus, cell proliferation/survival and neurogenesis is increased in GFAP(-/-)*Vim*(-/-) mice compared to wildtype controls [319].

**4) *Dlx2* (Distal-Less Homeobox 2)**

**ABA review:** Moderate-to-high expression was present in the subependymal zone and rhinocele. Similar levels of labeling were found in the lateral ventricle and in the rostral migratory stream. There was low-to-moderate expression in the glomerular, granular cell layers and the olfactory nerve layer of main olfactory. There did not appear to be much off-target expression of *Dlx2*. In an independent review of the ABA images, it was reported that there was no expression in the dentate subgranular zone. However there was specific expression in the cerebellar nuclei neurons and in the Purkinje cell layer.

Sagittal section Coronal section

**GENSAT:** In the adult mouse strong to moderate expression is found in 5 brain regions: amygdala, hypothalamus, olfactory bulb, septum, and supraoptic nucleus. Three BAC lines have overlapping expression at P7. The BAC data is consistent with the literature. Additional expression sites are observed in midbrain, cerebellum (at P7) and brainsstem. In the adult brain, compared with the *in situ* hybridization data from ABA and GenePaint.org, the subventricular zone is not stained in the BAC data. *Dlx2* is expressed in cortical interneurons with highest levels of expression during embryogenesis [320]. The BAC only shows weak expression in the cortical plate at E15-5. The postnatal in situ data from both ABA and GenePaint failed to detect any specific hybridization signals in the cortex.

**Literature:** In the adult mouse brain the entire extent of the rostral migratory stream (RMS) from the anterior subventricular zone to the olfactory bulb contains precursor cells heavily that were labeled for both *Dlx2* mRNAs [321]. Levels of mRNA decrease along the migratory route, with the strongest labeling in the RMS, moderate levels of mRNA in the granule cell layer, and relatively weak label in periglomerular cells. *Dlx2* is also expressed in the ependymal area adjacent to the lateral ventricle, the cortex, ventral forebrain, thalamus, hippocampus, and hypothalamus. *Dlx1* and *Dlx2* are required for down regulating Notch signaling during specification and differentiation steps of 'late' progenitors (P3) [322].

**5) *Nes* (Nestin)**

**ABA review:** Moderate-to-high expression could be seen in the rostral migratory stream. Some cells in the hippocampal CA3, pyramidal layer also expressed *Nes* at high levels. Moderate expression could be seen in the Purkinje cell and the granular cellular layer of the cerebellum. In some slides there appeared to be some expression in the olfactory tubercule and the lateral segment of the paragigantocellular reticular nucleus. In coronal sections there was significant punctate, moderate expression throughout the brain. Additionally, *Nes* levels were found to be lower in the cerebral white matter and the cerebral nuclei. In an indepent review of the ABA images, it was noted that there was only light and not very specific expression in the neurogenic regions.

Sagittal section Coronal section

**GENSAT:** In the adult mouse strong to moderate expression is found in 21 brain regions: amygdala, anterior olfactory nucleus, basal forebrain, caudate putamen, cerebellum, cerebral cortex, dorsal horn, entorhinal cortex, globus pallidus, hippocampus, hypothalamus, medulla, midbrain, olfactory bulb, piriform cortex, pons, septum, substantia nigra, thalamus, ventral horn, and ventral striatum. BAC data is consistent with the literature and BGEM *in situ* hybridization data in general. In E15-5 BAC embryos, expression is observed largely in the developing cerebellum and in the telecephalon. The *in situ* data shows higher *Nes* expression in the ventricular zone. During the postnatal development, expression persists in the BAC mice even though the published data suggests a down-regulation of nestin expression. Labelled cells are restricted to glial cells and vascular cells. The expression in glial cells in postnatal BAC mice is in agreement with the previous findings.

**Literature:** *Nes* represents a characteristic marker of multi-lineage progenitor cells and suggests that its presence in cells may indicate multi-potentiality and regenerative potential [323]. In the adult mouse brain, *Nes* mRNA expression is detected in the membranes between cerebellum and cortex and near hippocampus, the recess of the third ventricle and the choroid plexus [324]. Transgenic mice were generated that expressed GFP under the control of the neural precursor-specific form of *Nes* [325]. Adult expression of GFP was seen only in known areas of adult neurogenesis, including, the subventricular zone and the dentate gyrus. In transgenic mice, an enhancer in the second intron of the nestin gene was shown to be able to drive neural precursor specific expression [326]. The 5.4 kb upstream nestin promoter and the 700-bp second intron direct expression to neural precursor cells [327].

**6) *Dlx1* (Distal-Less Homeobox 1)**

**ABA review:** Very high expression of *Dlx1* was observed in the subependymal zone and rhinocele. In addition, high levels of labeling were present in the lateral ventricle and in the rostral migratory stream. Slightly lower levels of expression were present in the glomerular granular and mitral layers of the main olfactory bulb. There was moderate expression in the rostroventral part of the lateral septal nucleus. In the pallidum there were similar levels of expression in the diagonal band and medial septal nuclei. In the caudoputamen moderate labeling was present in the anterodorsal segment of the medial amygdalar nucleus. In the hypothalamus, moderate-to-high expression was present in the suprachiasmatic and arcuate nuclei. In the cerebellum there was moderate expression in the Purkinje cell layer. Finally, sparse punctate staining could also be observed throughout the cerebral cortex. In an independent review of the ABA images, it was found that Dlx1 labeled cells of the rostral migratory stream, but with no specific label in the subgranular zone. In addition, many other cells throughout brain have above-background label for *Dlx1*.

Sagittal section Coronal section

**GENSAT:** In the adult mouse strong to moderate expression is found in 15 brain regions: amygdala, anterior olfactory nucleus, basal forebrain, caudate putamen, cerebral cortex, entorhinal cortex, globus pallidus, hippocampus, hypothalamus, olfactory bulb, piriform cortex, rostral migratory stream, stria terminalis, subicular cortex, and ventral striatum. Two BAC lines are identical at P7. These BAC lines show restricted expression in the forebrain, and almost no expression in midbrain and hindbrain, except a few scattered stained fibers. The BAC data is consistent with the published data and *in situ* hybridization data.

**Literature:** In the adult mouse brain, the entire extent of the rostral migratory stream from the anterior subventricular zone to the olfactory bulb contained precursor cells is heavily labeled for *Dlx1* mRNAs [321]. High mRNA levels are found in all regions of the olfactory bulb, including the ependymal zone and granule and periglomerular cells. *Dlx1* is also expressed in the ependymal area adjacent to the lateral ventricle, the cortex, ventral forebrain, thalamus, hippocampus, and hypothalamus.

**7) *Fabp7* (Fatty Acid Binding Protein 7, Brain)**

**ABA review:** Moderate-to-high labeling of *Fabp7* was observed in the Purkinje cells. In some slides, there also was strong expression in the olfactory nerve layer of the main olfactory bulb and the glomerular layer of the accessory olfactory bulb. In an independent review of the ABA images, it was also noted that there was nice and very specific staining in cerebellum in what are likely Bergmann glia. In addition, there was also expression in the cells of the adult neurogenic regions of the subgranular zone of the dentate and rostral migratory stream.

Sagittal section Coronal section

**GENSAT:** In the adult mouse strong to moderate expression is found in the cerebellum and olfactory bulb. Our data matches BGEM *in situ* data and the literature. BGEM data only detects cerebellar expression at P7, whereas the BAC data shows low levels of expression in other areas of the brain consistent with the literature.

**Literature:** Also referred to as *B-Fabp* or *Blbp* (brain lipid-binding protein). *In situ* hybridizations identified low levels of *fabp7* mRNA throughout the brain and higher levels within the glial limiting membranes surrounding ventricles of the mouse brain [328]. Expression is widespread in the 11-day-old rat brain [329]. The highest levels of expression can be found in fiber tracts, to a lesser degree, in most gray matter areas, including the anterior olfactory nuclei, striatum, hippocampal formation, thalamus, and cerebral cortex. *B-Fabp* mRNA was abundant in the ependymal and subependymal layers of lateral ventricles. EGFP, EYFP, and dsRed2 transgenic mice under the control of the *Blbp* promoter were generated to develop mouse models for the visualization and study of radial glia [330]. In these transgenic lines, fluorescent protein expression was restricted to radial glia in the embryonic cortex and to astrocytes in the adult brain.

**8) *Igfbpl1* (Insulin-Like Growth Factor Binding Protein-Like 1)**

**ABA review:** Very broad moderate-level expression could be seen throughout the brain. However, in the hippocampal formation there was moderate-to-high expression in the CA1, CA2, CA3 fields and somewhat lower expression in the dentate gyrus. In an independent review of the ABA images, it was noted that there was non-specific staining throughout the brain. In particular, the highest number of Igfbpl1+cells could be found basal forebrain.

Sagittal section Coronal section

**GENSAT:** No information available.

**Literature:** Using FACS sorting to enrich neural progenitor cells from the adult mouse brain and Affymetrix microarrays, genes specifically expressed in these cells were identified [331]. *In situ* hybridization confirmed that *Igfbpl1* was expressed in the subventricular region of central nervous system germinal zones at E13, E17 and in the adult.

**9) *Lrrn1* (Leucine Rich Repeat Protein 1, Neuronal)**

**ABA review:** Moderate expression of *Lrrn1* was present in the cells lining the lateral ventricle. In some slides, there appeared to be moderate expression in the subependymal zone and rhinocele. In the main olfactory bulb there were similar levels of staining in the mitral layer and low punctate labeling in the granular layer. Somewhat lower amount of staining could be seen throughout the anterior olfactory nucleus. There was moderate-to-high labeling in the molecular layer of the piriform area and the adjacent piriform-amygdalar area. In the pallidum medium levels of expression was present in the diagonal band and medial septal nuclei. In the hippocampal formation, very high expression could be found throughout the granular layer of the dentate gyrus and in the pyramidal cell layers in CA1 and CA2. Within the thalamus there was moderate expression in the parafascicular nucleus. In much of the midbrain there was sparse moderate expression of *Lrrn1*. However, somewhat higher levels were observed in the anterior pretectal nucleus midbrain reticular nucleus. In the medulla there was high expression in the magnocellular segment of the lateral reticular nucleus. Within the pons there were similar levels of expression in the pontine gray and the nucleus of the lateral lemniscus. Finally, moderate but specific labeling could be seen in the cerebellar Purkinje cell layer.

In an independent review of the ABA images, it was reported that many neurons are labeled throughout the brain and there did not appear to be specific to the rostral migratory stream and subgranular zone. *Lrrn1*+ staining was found in the Purkinje cells, regio superior pyramidal cells, dentate granule cells and layer 5 cortical neurons.

Sagittal section Coronal section

**GENSAT:** In the adult mouse strong to moderate expression is found in 22 brain regions: amygdala, anterior olfactory nucleus, basal forebrain, caudate putamen, cerebellum, cerebral cortex, dorsal horn, entorhinal cortex, globus pallidus, hippocampus, hypothalamus, medulla, midbrain, olfactory bulb, piriform cortex, pons, septum, subicular cortex, substantia nigra, thalamus, ventral horn, and ventral striatum. Two BAC lines have matching expression at P7. The BAC data grossly matches BGEM *in situ* hybridization data.

**Literature:** FACS sorting and Affymetrix microarrays identified *Lrrn1* as being specifically expressed in adult mouse brain neuronal progenitor cells [331]. *In situ* hybridizations confirmed that *Lrrn1* was expressed strongly in adult dentate gyrus, but was not detectable above background in the subventricular zone.

**10) *Rrm1* (Ribonucleotide Reductase M1 Polypeptide)**

**ABA review:** High expression of *Rrm1* was found in the rostral migratory stream. In addition, there was moderate expression in the lateral and 4th ventricle and the lateral recess. Significant, moderate and punctuate expression could also be observed throughout the midbrain and the brainstem. In particular, some slides displayed high density of moderate expression in the pontine gray, medial vestibular nucleus, spinal vestibular nucleus, and the lateral portion of the paragigantocellular reticular nucleus. An independent review of the ABA images, reported that there was nice discrete label in the rostral migratory stream and scattered cells in dentate granule cells that likely includes a few intensely labeled cells in the subgranular zone. In fact, the type of label seen in dentate was also observed in many neurons within the forebrain.

Sagittal section

**GENSAT:** No information available.

**Literature:** Immunocytochemical studies detected *Rrm1* in neural stem cells within the subgranular layer in the hippocampal dentate gyrus [332]. Most *Rrm1* immunoreactive cells were bipolar to multipolar, and had a large cell body and long processes. Two different populations of *Rrm1* expressing cells were visualized in the subventricular zone in the forebrain, one dominated by small, bipolar cells extending into the rostral migratory stream, while the other was formed by large multipolar cells, adjacent to the ependyma, with processes extending to the lateral ventricle.

**11) *Sox2* (Transcription factor SOX-2)**

**ABA review:** High levels of *Sox2* expression were present in the cells lining the lateral ventricle. Similar levels of staining were also present in the rostral migratory stream and what appears to be the subependymal zone and rhinocele. In the main olfactory bulb, there was high expression in the glomerular cell layer. Slightly lower levels were present in the granular cell layer. Moderate-to-high labeling could be seen in the granular cell layer of the dentate gyrus. High expression was found in the cerebellar Purkinje cell layer. Significant moderate punctuate expression could be observed throughout the brain. In particular, some slides displayed high density of moderate expression in the central amygdalar area, and layers 1, 2/3 of the cerebral cortex. In an independent review, it was noted there was very nice labeling in subgranular zone and in rostral migratory stream. In addition, there was some staining of neurons in cortex and cerebellar Purkinje cell layer.

Sagittal section

**GENSAT:** No information available.

**Literature:** 5.7 kb of *Sox2* flanking sequence drove β-geo reporter activity in ventricular zone cells of the mouse embryo and to periventricular cells of the adult brain [333]. Using *Sox2::*EGFP transgenic mice, it was shown that EGFP was expressed in the core of the olfactory bulb [334]. Highest EGFP expression was found in regions enriched in neural stem cells, the subventricular zone (SVZ) and the subgranular layer (SGL) of the hippocampus. Along the anterior lateral ventricle, EGFP expression was seen in more than 50% of the cells comprising the ependymal layer and the immediately subjacent SVZ. EGFP was also pronounced within the hippocampus, localizing to the hilus, the SGL of the dentate gyrus, and the ependymal and subependymal layers of the fimbria. Scattered cells within the cortex in both young adult and middle-aged animals were EGFP+, as were small cells proximal to the Purkinje cells of the cerebellum. In the adult mouse brain, *Sox2* deficiency results in neurodegeneration and impaired neurogenesis [335]. Precursor cell proliferation and the generation of new neurones in adult neurogenic regions are greatly decreased, and GFAP/nestin+ hippocampal cells, which include the earliest neurogenic precursors are also strikingly diminished.

**12) *Thbs4* (Thrombospondin 4)**

**ABA review:** High expression levels of *Thbs4* were found in the subependymal/rhinocele. Similar amounts of labeling were also present in the lateral ventricle. Interestingly, a large number of cells in the corpus callosum and anterior commissure, olfactory limb also expressed *Thbs4* at moderate-to-high levels. In the hippocampal formation some cells in the subiculum were also labeled. In an independent review of the ABA images, it was observed that the gene was specifically expressed, and at rather high levels, in the adult neurogenic regions of the rostral migratory stream and the subgranular zone of the dentate gyrus.

Sagittal section Coronal section

**GENSAT:** In the adult mouse strong to moderate expression is found in 15 brain regions: amygdala, caudate putamen, cerebellum, cerebral cortex, dorsal horn, hippocampus, hypothalamus, medulla, midbrain, olfactory bulb, pons, septum, thalamus, ventral horn, and ventral striatum. Two BAC lines have identical expression patterns at P7. The BAC data needs to be confirmed by *in situ* hybridizations studies.

**Literature:** *Thbs4* transcript can be detected in both the developing and adult nervous system [336]. *Thbs4* immunoreactivity is present at some neuronal cell bodies, e.g., granule cell and pyramidalneurons in the hippocampus, Purkinje cells in the cerebellum, and subpopulations of pyramidal neurons in the cerebral cortex. *Thbs4* is a neuronal extracellular matrix protein associated with certain synapse-rich structures in the adult, and may be involved in local signaling in the developing and adult nervous system. *Thbs4* is expressed by vascular cells and influences the vessel wall by modulating the proliferation of human endothelial cells and smooth muscle cells [337]. Using microarray expression analysis, it was shown that *Thbs4* has an expression profile characteristic of grade I astrocytomas [338].

**13) *Nr2e1* (Orphan Nuclear Receptor NR2E1)**

**ABA review:** Images are not available for *Nr2e1*.

**GENSAT:** No information available.

**Literature:** Also referred to as fierce, *Tll*, and *Tlx*. Taking advantage of a β- galactosidase reporter knocked into the *Tlx* locus, the *Tlx* expression pattern was examined in adult brains of heterozygote mice [339]. Beta-galactosidase staining was distributed sparsely throughout the cortex, but indicated high but dispersed expression of *Tlx* in the subgranular layer of the dentate gyri and clustered expression in the subventricular zone - two main sites where adult neural stem cells (NSCs) are located. *Tlx* mutant mice showed a loss of cell proliferation and reduced labelling of nestin in neurogenic areas in the adult brain. *Tlx* -/- mice have a dramatic reduction in retina thickness and enhanced generation of S-cones, and developed severe early onset retinal dystrophy [340].

**14) *Dscam* (Down Syndrome Cell Adhesion Molecule Precursor)**

**ABA review:** Moderate expression could be seen in layers 2/3, 5 and 6 of the cerebral cortex. In addition similar levels of labeling were present in the glomerular, mitral and granular layers of the main olfactory bulb. Within the hippocampal formation, moderate-to-high expression was found along Ammon's horn and slightly higher levels were seen in the granule cell layer of the dentate gyrus. Moderate-to-high staining was also seen in the lateral ventricle in what appears to be the choroid plexus. Finally, sparse, punctate, moderate expression was seen throughout the midbrain and brainstem.

Sagittal section

**GENSAT:** No information available.

**Literature:** In the adult mouse brain, *Dscam* is expressed in the molecular layer of the cerebellum, granule cells in the hippocampal dentate gyrus, pyramidal cells in the CA3 and CA1 regions, granule cells in the olfactory bulb, and some regions in the cerebral cortex [341]. *Dscam* is involved in the adult neurogenesis of primate hippocampus after cerebral ischemia. In the subgranular zone, cerebral ischemia leads to a marked increase of Dscam+ cells with upregulation seen in two cell types: immature neurons and astrocytes positive for S100beta. Young astrocytes are in intimate contact with newly generated neurons in the subgranular zone [342].

**Subanatomical region: Raphe nuclei, Therapeutic interest: Serotonin system, Depression**

The raphe nuclei is cluster of nuclei containing serotonergic neurons found in the brain stem and releases serotonin to the rest of the brain [343]. Serotonin, also called 5-hydroxy-tryptamine (5-HT), is implicated in many neurological disorders such as anorexia, depression, and sleep disorders. Selective serotonin reuptake inhibitor (SSRI) antidepressants are believed to act in these nuclei, as well as at their targets [344].

**1*. Fev* (ETS-Domain Transcription Factor Pet-1)**

**ABA review**: There was low-to-moderate expression in the periaqueductal gray region. Moderate-to-high expression was seen in the cerebellar granular layer. Strong labeling was seen in the glomerular, granular and mitral layers of the main olfactory bulb. Low-to-moderate expression was also found in layer 1 of the cerebral cortex and throughout the pons and medulla.

Sagittal section Sagittal section (zoomed)

**GENSAT:** No information available

**Literature:** *Fev/Pet1* is exclusively expressed in the midline part of the human brainstem containing raphe nuclei, which also specifically expressed 5-HT transporter (*Sert*) and tryptophan hydroxylase (Tph2), two markers of the 5-HT neurotransmitter system [345]. In the rat brain, *Pet1* expression is restricted to, and marks, the entire rostrocaudal extent of rat serotonergic hindbrain raphe nuclei [346]. *Pet1* expression in the mouse brain is in agreement with the rat data, with expression detected in central serotonergic neurons located in the mes-/metencephalic raphe nuclei from E11 on until adulthood [347]. In these regions gene expression co-localizes precisely with *Sert*. In *Pet1* mutant mice, majority of 5-HT neurons fail to differentiate and the remaining neurons show deficient expression of genes required for 5-HT synthesis, uptake, and storage [348]. There is heightened anxiety-like and aggressive behavior in adult mice. A 40 kb upstream fragment of the *Fev* promoter was used to generate transgenic mice in which transgene expression was correctly targeted to serotonergic neurons and displayed little to no ectopic expression [349]. Enhancer sequences within the 40 kb upstream fragment are responsible for the directed transgene expression. Moreover, virtually all of the 5-HT neurons in the adult were LacZ+ in all of the lines examined. Expression was maintained when the 40 kb fragment was truncated on its 5' end to either 12- or 1.8 kb, although position independence was then lost. Analysis of transgene expression in *Pet1* null mice indicated that Pet1 was required to maintain the activity of the *Pet1* enhancer region in a subset of 5-HT neurons. Pet1 plays a critical role in 5-HT neuron development and is required for normal anxiety-like and aggressive behavior [348].

**2*. Gchfr* (GTP Cyclohydrolase I Feedback Regulator)**

**ABA review:** There was very specific expression in the raphe nuclei with limited expression in rostral brain and brainstem. An independent review indicated specific and high level of expression in the dorsal nucleus raphe. Slightly lower levels of expression were present in the medial part of the superior central nucleus raphe. There was also significant scattered expression in the piriform area.

Sagittal section Sagittal section (zoomed)

**GENSAT:** No information available

**Literature:** This gene is also called *Gfrp*. In the rat brain *Gfrp* mRNA expression is abundant in serotonin neurons of the dorsal raphe nucleus, but is undetectable in dopamine neurons of the midbrain or norepinephrine neurons of the locus coeruleus [350].

**3.  *Slc6a4* (Solute Carrier Family 6, Member 4; Serotonin Transporter)**

**ABA review:** There was high expression in the dorsal raphe nucleus and superior central nucleus raphe (medial part), and possibly the trochlear nucleus.

Sagittal section Sagittal (zoomed)

**GENSAT**: There are 22 regions of moderate-to-strong expression in the adult. The BAC data has correct expression in raphe nuclei. Fiber projections are stained across the central nervous system, giving an impression that EGFP reporter gene is widely expressed inside. In fact, labeled cell bodies are only observed in a few brain areas at P7, including raphe nuclei. Other sites, including main olfactory bulb, thalamus and cortex, are confirmed by literature and BGEM *in situ* data.

**Literature:** The gene is also called *Sert*. In the rat brain *Sert* is mainly expressed in the raphe nuclei. *In situ* hybridization studies established the specific localization of *Sert* gene expression to the raphe nuclei in both mouse and rat brains [304, 351]. A knock-in strategy was used to generate a line of mice expressing Cre recombinase under the transcriptional control of the serotonin transporter promoter (*Sert*-*cre* mice) [352]. Immunohistochemical staining of adult transgenic mouse brain sections revealed Cre recombinase expression in the raphe nuclei. A 2.2 kb promoter fragment of the mouse serotonin transporter drove expression of the luciferase reporter gene in a 5-HTT-expressing cell line and in serotonergic raphe neurons derived from embryonic rat brainstem [353].

**4. *Slc17a8* (Sodium-Dependent Inorganic Phosphate Cotransporter)**

**ABA review:** There was significant label in terms of intensity and numbers of cells in all of the raphe neuronal groups. Additionally, there was diffuse but nicely labeled sets of cells in anterior cortex, layer Va, striatum (a very nice minority subpopulation), bed nucleus, and central nuclei of thalamus. Sporadic, but nicely labeled cells, could be found in hippocampus pyramidal cell dendritic territory

An independent review indicated very high expression in the lateral and medial parts of the superior central nucleus raphe, the dorsal nucleus raphe and the tectospinal pathway. High expression was also seen in the lateral septal nucleus, ventral part, and moderate expression was found throughout layer 4 of cerebral cortex. There was moderate labeling of the medial segments of the thalamus, including the inter-anteromedial nucleus of the thalamus and rhomboid nucleus, nucleus of reunions and central medial nucleus of the thalamus. Sparse punctuate staining was present in the caudo-putamen and the nucleus accumbens. Some moderate scattered staining was present in the pontine central gray and in the tegmental reticular nucleus.

Sagittal section Sagittal section (zoomed)

**GENSAT:** No information available

**Literature:** This gene is also called *Vglut3*. *Vglut3* mRNA expression is restricted to a small number of neurons scattered in the striatum, hippocampus, cerebral cortex, and raphe nuclei in the rat brain [354]. In the mouse brain, Vglut3 is expressed in interneurons in cortex and hippocampus, in the striatum and in mesopontine raphe nuclei [355].

**5. *Tph2* (Tryptophan hydroxylase 2)**

**ABA review:** This gene expression is high and specific in the medial part of the superior central nucleus raphe and in the dorsal nucleus raphe. Moderate-to-high labeling could also be seen in the ventral portion of the pontine reticular nucleus. An independent review indicated that specific and strong expression in all components of the raphe nuclei, including median, dorsal, paramedian, magnus, pallidus, and obscurus.

Sagittal section Sagittal (zoomed)

**GENSAT:** There are 25 regions of moderate-to-strong expression in the adult. BAC data shows strong expression in raphe nucleus, which correlates well with published biochemical studies and BGEM *in situ* data. The BAC data also indicates additional expression sites that are not observed in the *in situ* data.

**Literature:** In transgenic mice, a 6.1 kb mouse *Tph2* promoter was able to direct the *lacZ* expression to serotonergic tissues in the pineal gland, as well as a moderate level of *lacZ* expression in serotonergic brain regions such as the median and dorsal raphe nuclei, the nuclei raphe magnus and raphe pallidus [356]. Additionally, *in situ* hybridization demonstrated specific labeling of Tph within the dorsal raphe nucleus of the rat brain; higher levels are present in pineal gland [357]. Tph antiserum localized immunoreactive cells in the dorsal raphe nucleus of rat and mouse brains. Moderate immunoreactivity was also observed in the medial raphe nuclei [358]. The existence of the two isoforms Tph1 and Tph2 was identified fortuitously after discovering that the Tph (-/-) mice still produced serotonin in the brainstem, with ablated synthesis in the peripheral tissues [359]. Only *Tph2* is expressed in the brain (mainly in the brainstem); *Tph1* is expressed in peripheral tissues like gut. Tph2 is required for brain serotonin synthesis [360, 361]. A loss-of-function mutation in human *TPH2* is an important risk factor for unipolar major depression [361].

**6. *Maob* (Monoamine Oxidase B)**

**ABA review:** High levels of expression were present in the dorsal nucleus raphe and in the medial part of the superior central nucleus raphe. Moderate-to-high labeling was also found in the midline group of the dorsal thalamus that includes the parataenial nucleus, paraventricular nucleus of the thalamus, and nucleus of reunions. Slightly lower levels of expression could also be seen in the central medial nucleus of the thalamus. In some sagittal sections sparse staining could be seen throughout the brainstem.

An independent review indicated specific but limited expression in the raphe nuclei populations that were labeled, particularly the median and dorsal nuclei. Additonally, there was label in some anterior forebrain cells, midline thalamic nuclei, and cells of the choroids plexus.

Sagittal section Sagittal section (zoomed)

**GENSAT:** No information available

**Literature:** In the mouse brain, using *in situ* hybridization and histochemistry expression was detected in the serotoninergic neurons of the raphe from developmental stages E12 to P7, remaining stable during postnatal life [362]. Expression was also found in intermediary thalamic nuclei and in a variety of non-neuronal cells, the choroid plexus, the ependyma, and astrocytes. In the rat brain, high expression was found only in the area postrema, the subfornical organ, and the dorsal raphe [298].

**7. *Esr2* (Estrogen Receptor 2)**

**ABA review:** There appeared to be low-to-moderate expression in the dorsal nucleus raphe. However, there was high expression in the stria terminalis, the optic tract and the medial amygdalar nucleus. In addition, there was also moderate scattered expression interfascicular nucleus of the bed nuclei of the stria terminalis and to some extent the medial preoptic area. An independent review of the ABA images indicated that labeling of raphe nuclei is modest and limited; there also was label in the bed nucleus and anterior hypothalamus.

Sagittal section Sagittal section – zoomed

**GENSAT:** The BAC data is consistent with the literature. In the adult hippocampus, the EGFP transgene expression is confined in subiculum; this needs to be further confirmed by *in situ* hybridization.

**Literature:** This gene is also called *ERbeta*. ERbeta immunoreactivity was primarily localized to cell nuclei within the dorsal raphe, olfactory bulb, cerebral cortex, septum, preoptic area, bed nucleus of the stria terminalis, amygdala, paraventricular hypothalamic nucleus, thalamus, ventral tegmental area, substantia nigra, locus coeruleus, and cerebellum [363]. Extranuclear immunoreactivity was detected in olfactory bulb, CA3 stratum lucidum, and CA1 stratum radiatum of the hippocampus and cerebellum.

**Subanatomical region: Striatum, Therapeutic interest: Huntington Disease, Parkinson Disease, Plasticity in Depression**

The striatum, consisting of the caudate and the putamen, is a subcortical part of the telencephalon and the major input station of the basal ganglia system. Parkinson disease results in loss of dopaminergic innervation to the striatum (and other basal ganglia). A striatal lesion is also involved in the Huntington disease.

**1. *Adora2a* (Adenosine A2a Receptor)**

**ABA review:** There was moderate-to-high expression in the caudoputamen and the nucleus accumbens. Similar levels of labeling were found in layer 1 of the olfactory tubercle. Very little expression of *Adora2a* was found in other brain regions.

Sagittal section Coronal section

**GENSAT:** There are 13 regions of strong to moderate expression in the adult: amygdala, basal forebrain, caudate putamen, dorsal funiculus, globus pallidus, lateral funiculus, locus coeruleus, olfactory bulb, pons, spinal cord, dorsal horn, spinal cord, ventral horn, ventral funiculus, and ventral striatum. BAC data matches both literature and BGEM *in situ* hybridization data.

**Literature:** This gene is also called *A2ar*. SAGE analysis revealed regional enrichment of *Adora2a* in the caudate-putamen of the adult C57BL/6 mouse [10]. Microarray analysis revealed *Adora2a* is regionally enriched in the adult mouse striatum [11]. In the rat central nervous system, specific expression is found in the caudate putamen, nucleus accumbens, olfactory tubercle, lateral septum and in some cerebellar Purkinje cells [364]. In normal human brain, *ADORA2A* mRNA is detected in striatal (nucleus accumbens, caudate nucleus and putamen) and extrastriatal (globus pallidus, substantia nigra) brain regions. A significant decrease in the level of adenosine A2A receptor mRNA is found in the anterior and posterior caudate nucleus and anterior dorsal putamen, whereas a significant increase is observed in the substantia nigra pars reticulata of the Parkinsonian brain when compared to age-matched controls [365].

**2. *Gpr88* (G Protein-Coupled Receptor 88)**

**ABA review:** There was specific expression in the striatum/accumbens. Additionally, cells that appeared to be in layer Va of cortex and basal pontine nuclei (reticular, raphe magnus, superior olive) were nicely labeled.

Sagittal section Coronal section

**GENSAT:** There are seven regions of moderate-to- strong expression in the adult, including caudate putamen, cerebellum, pons, spinal cord dorsal horn, spinal cord ventral horn, substantia nigra, and ventral striatum. BAC data is consistent with both literature and BGEM *in situ* data. In postnatal BAC transgenic mice, correct expression is observed in cortex, caudate putamen, ventral striatum, entorhinal cortex and inferior olive. However, the BAC also produces extra expression in medial habenular nuclei, preoptic area and hippocampus. At E15-5, the BAC data shows less expression in caudate putamen than what *in situ* hybridization data indicated, and additional expression in cerebellum.

**Literature:** SAGE analysis revealed regional enrichment of *Gpr88* in the caudate-putamen of adult C57BL/6 mice [10]. *GPR88* is highly expressed in both caudate nucleus and putamen, and is very faintly detected in medulla of the human brain [366]. Similarly, in the mouse brain, highest levels of expression are detected in the caudate-putamen. Signals were distinctly detected in nucleus accumbens and olfactory tubercle and less intensively in the inferior olive nucleus.

**3*. Drd1a* (Mouse Dopamine Receptor D1A)**

**ABA review:** In one review it was found that this gene is relatively specific for the extended striatum that includes the nucleus accumbens. Additionally, there was expression, albeit light, in some regions of basal amygdala and endopiriform cortex. Expression is largely limited in all other regions of the brain. This is the most widespread of dopamine receptors. An independent review of the ABA images indicated moderate expression throughout the caudoputamen and the nucleus accumbens. Similar levels of expression could be seen in layers 1 and 2 of the olfactory tubercle.

Sagittal section Coronal section

**GENSAT:** There are 22 regions of moderate-to-strong expression in the adult. GENSAT BAC clone not available on UCSC (just one end available through NCBI clone registry). BAC data correlates well with the published expression studies in embryonic and post-natal animals. Bergmann glial cells in adult BAC transgenic mice are labeled, which is inconsistent with the literature. The difference could be explained either by sensitivity of the BAC method, or the species-specific difference between rat and mouse brains (almost all the studies were performed in rat brains).

**Literature:** SAGE analysis revealed regional enrichment of *Drd1* in the caudate-putamen of the adult C57BL/6 mouse [10]. *DRD1* gene expression is detected at high levels in the caudate and putamen, as well as in the nucleus accumbens, in both the human and rat brain [367]. Microarray analysis revealed *Drd1a* is regionally enriched in the adult rat striatum [9, 15]. In the rat brain, mRNA was also abundant in the olfactory tubercles and several thalamic nuclei. *D1A*-/- mutants are growth retarded and die shortly after weaning age unless their diet is supplemented with hydrated food [368]. Neurologically, *D1A*-/- mice exhibit normal coordination and locomotion, although they display a significant decrease in rearing behavior. D1A receptor binding was absent in striatal sections from *D1A*-/- mice.

**4. *Drd2* (Dopamine receptor D2)**

**ABA review:** The expression pattern appeared to be very similar to *Drd1*. There was moderate-to-high expression throughout the caudoputamen and the nucleus accumbens. High levels of expression could be seen in layers 1 and 2 of the olfactory tubercle. However, there was also high expression in the ventral tegmental area of the midbrain. In addition, in coronal sections there appeared to be high expression in the subthalamic nucleus. An independent review indicated nice but more discrete label for striatum and accumbens. Other cell populations that were labeled are the substantia nigra and hypothalamus, and some dopaminergic populations that would be expected.

Sagittal section Coronal section

**GENSAT:** BAC data correlates well with the literature. There are 28 regions of moderate-to-strong expression in the adult: accumbens nucleus, amygdala, basal forebrain, caudate putamen, cerebellum, cerebral cortex, endopiriform nucleus, entorhinal cortex, globus pallidus, hippocampus, hypothalamus, medulla, midbrain, olfactory bulb, olfactory nerve layer, pons, retrosplenial cortex, septum, solitary nucleus, spinal cord dorsal horn, spinal cord ventral horn, subicular cortex, substantia nigra, thalamus, trapezoid body, ventral pallidum, ventral striatum and ventral tegmental area.

**Literature:** SAGE analysis revealed regional enrichment of *Drd2* in the caudate-putamen of the adult C57BL/6 mouse [10]. Microarray analysis revealed *Drd2* is regionally enriched in the adult mouse and rat striatum [9, 11, 15]. In the human brain, high expression is detected in caudate putamen, accumbens nuclei, the olfactory tubercle and the anterior lobe of pituitary gland; additionally in substantia nigra pars compacta and ventral tegmental area [367]. In the rat, expression is mainly detected in the Islands of Calleja and at lower levels in the anterior nucleus accumbens, the medial mammillary nucleus as well as in the bed nucleus of the stria terminalis. Expression is high in the intermediate lobe of the pituitary gland. High expression is detected in caudate, putamen, and pituitary of the human brain [369]. Moderate levels were detected in regions of catecholamine-containing cell bodies and in the amygdala.

**5. *Gpr6* (G Protein-Coupled Receptor 6)**

**ABA review:** Beautiful moderate-to-high levels of expression were present throughout the striatum. Slightly higher levels of labeling were present in the olfactory tubercule. In addition, there appeared to be low-to-moderate expression throughout the main olfactory and the brainstem. Similar levels of expression could also be observed in the cerebellar Purkinje cell layer.

Sagittal section Coronal section

**GENSAT:** Multiple BAC lines are identical at developmental stage P7. The BAC data matches BGEM *in situ* data post-natally. The E15-5 *in situ* data shows no specific hybridization signals.

**Literature:** SAGE analysis revealed regional enrichment of *Gpr6* in the nucleus accumbens of the adult C57BL/6 mouse [10]. In the rat brain, *in situ* hybridization detected dense expression in the caudate putamen, nucleus accumbens and olfactory tubercle. Lower levels were found in the hypothalamus, posterior cingulate, retrosplenial cortex, and substantia nigra compacta. Labelling is faint in the hippocampus [370]. Microarray analysis revealed *Gpr6* is regionally enriched in the adult rat striatum [9]. Northern blot revealed that human *GPR6* RNA is abundant in the putamen and to a lesser extent in the frontal cortex, hippocampus, and hypothalamus [371].

**6. *Rgs9* (Regulator of G-protein Signaling 9)**

**ABA review:** There was very high expression throughout the caudoputamen, the nucleus accumbens and layers 1 and 2 of the olfactory tubercle. The posterior part of the periventricular hypothalamic nucleus showed low-to-moderate labeling. In the pons there was moderate expression in the locus coeruleus. Within the medulla there appeared to be moderate expression in medial part of the nucleus of the solitary tract. Sparse diffuse label could be seen throughout the cerebral cortex.

An independent review indicated nice expression in the striatum/accumbens. There was specific expression in the suprachiasmatic and paraventricular nuclei of the hypothalamus. Some positive cells wrap down to the amygdaloid nucleus but avoid the lateral amygdala. Two discrete regions in brainstem also seem to be positive: nucleus ambiguous and a component of the solitary nucleus. These are important autonomic relay stations in the central nervous system.

Sagittal section Coronal section

**GENSAT**: The BAC data matches the literature and BGEM *in situ* data. At P7, the BAC is expressed in many areas of the central nervous system, in contrary to the more restricted expression pattern seen at adult, but grossly matches P7 *in situ* hybridization data. This may reflects the dynamic expression of *Rgs9* during the development. There are 28 regions of moderate-to-strong expression in the adult: amygdala, anterior olfactory nucleus, basal forebrain, caudate putamen, cerebellum, cerebral cortex, entorhinal dortex, fasciculus retroflexus, globus pallidus, hippocampus, hypothalamus, medulla, midbrain, nigrostriatal bundle, olfactory bulb, optic tract, piriform cortex, pons, rostral migratory stream, septum, solitary nucleus, spinal cord dorsal horn, stria terminalis, subicular cortex, substantia nigra, supraoptic nucleus, thalamus, and ventral striatum.

**Literature:** SAGE analysis revealed regional enrichment of *Rgs9* in the caudate-putamen of adult C57BL/6 mice [10]. Microarray analysis revealed *Rgs9* is regionally enriched in the adult rat striatum [9]. In the rat brain, *Rgs9* expression is extremely dense in caudoputamen, nucleus accumbens, and olfactory tubercle regions of the striatum; relatively dense in medial hypothalamus [372]. Lower levels of expression can be found in the amygdala as well. Alternative splicing of transcripts gives rise to two isoforms: a brain and a retinal isoform. In the human brain, using the antibody to the brain isoform (Rgs9-2), immunostaining was observed in both putamen and caudate basal ganglia as clusters of vesicles surrounding interneuron cell bodies [373]. *Rgs9* null mice develop abnormal involuntary movements resembling drug-induced dyskinesia when inhibition of dopaminergic transmission is followed by activation of D2-like dopamine receptors [374].

**7. *Adcy5* (Adenylate Cyclase 5)**

**ABA review:** There was very strong expression in the striatum and accumbens, but also in numerous other brain regions such as mitral cells of olfactory bulb, CA2 of hippocampus.

Sagittal section Coronal section

**GENSAT:** No information available

**Literature:** SAGE analysis revealed regional enrichment of *Adcy5* in the caudate-putamen of adult C57BL/6 mice [10]. *Adcy5* is selectively concentrated in the rat corpus striatum as detected by Northern analysis, *in situ* hybridization [375] and microarray analysis [15]. Expression of *Adcy5* is reduced in R6/1 Huntington disease transgenic mice as assessed by Affymetrix array analysis [376]. Disruption of *Adcy5* led to a major loss of adenylyl cyclase activity in a striatum-specific manner [377]. Null mice exhibited Parkinsonian-like motor dysfunction, i.e. abnormal coordination and bradykinesia.

**8. *Crym* (Crystallin, Mu)**

**ABA review:** There was high expression throughout the nucleus accumbens, layers 1 and 2 of the olfactory tubercle and the medial portion of the caudoputamen. However, very high expression could be observed throughout the cerebral cortex. In addition, very high expression could be seen in the hippocampal formation. Moderate-to-high expression could be seen in the mitral layer of the main olfactory bulb. There was high expression in lateral, medial and dorsal portions of the anterior olfactory nucleus.

Sagittal section Coronal section

**GENSAT**: The gene is restricted to the dorsal and temporal side of retina. The BAC data is reproducible and needs to be confirmed by BGEM *in situ* data. The adult BAC data is consistent with *in situ* hybridization data from Allen Brain Atlas. Few cells in the layer 6 of the cortex seem to be labeled in the BAC DAB images, but confocal images indicates many pyramidal neurons in that layer are EGFP+, which agrees with Allen *in situ* hybridization data. BGEM data reveals strong expression of *Crym* mRNA in caudate putamen. The BAC produces expression in caudate putamen, but at much lower levels. There are 28 regions of moderate-to-strong expression in the adult: amygdala, anterior olfactory nucleus, anterior commissure, basal forebrain, caudate putamen, cerebral cortex, cerebral peduncle, entorhinal cortex, fornix, globus pallidus, hippocampus, hypothalamus, indusium griseum, internal capsule, longitudinal fasciculus of pons, medulla, midbrain, olfactory bulb, olfactory nerve layer, piriform cortex, pons, pyramidal tract, septum, spinal cord dorsal horn, spinal cord ventral horn, stria terminalis, thalamus, and ventral striatum.

**Literature:** This gene is also referred to as NADP-regulated thyroid-hormone binding protein or Cytosolic T(3)-binding protein in the literature [378]. SAGE analysis revealed regional enrichment of *Crym* in the nucleus accumbens of the adult C57BL/6 mouse [10]. High but heterogeneous expression was observed in the brain [379]. Sites of highest expression included cerebral cortex, cerebellum, amygdala, caudate nucleus, hippocampus, putamen and accumbens. Significantly lower expression was found in pons, corpus callosum, medulla oblongata, substantia nigra, thalamus and spinal cord.

**9. *Foxp1* (Forkhead box P1)**

**ABA review:** There was moderate expression throughout the caudoputamen, the nucleus accumbens and layers 1 and 2 of the olfactory tubercle. Similar levels of expression were present in layers 1, 2/3 and 4 of the cerebral cortex. Within the thalamus there was moderate expression in the paraventricular, anteromedial, parafascicular nuclei.

An independent review indicated that there was strong expression in the striatum, and moderately dense, but high, general background.

Sagittal section Coronal section

**GENSAT:** No information available

**Literature:** Microarray analysis revealed *Foxp1* is regionally enriched in the adult mouse striatum [11].

**10. *Lpl* (Lipoprotein lipase)**

**ABA review:** There was moderate-to-high expression throughout the caudoputamen, the nucleus accumbens and layers 1 and 2 of the olfactory tubercle. There was high expression could be seen in the hippocampal formation. In the cortex there was moderate expression in the cortical amygdalar area, posterior part, lateral zone. In addition, low-to-moderate expression could be seen in the dorsal part of the taenia tecta. Moderate-to-high labeling could be seen in the posteroventral part of the medial amygdalar nucleus. Moderate expression could be seen in the posterior part of the basomedial amygdalar nucleus and possibly the ventral portion of the basolateral amygdalar nucleus.

An independent review indicated that there was moderate expression in the striatum, some expression in the cortex, and highest but patchy expression in hippocampus. Coronal section displayed mainly in the hippocampus and no signal in striatum at all.

Sagittal section Coronal section

**GENSAT:** No information available

**Literature:** In newborn mice, *Lpl* mRNA is strongly expressed in the pyramidal neurons of the hippocampus; in adult mice, the cRNA probe for *Lpl* hybridized to the hippocampus [380]. In the mouse brain there is widespread expression of *Lpl* mRNA mainly in pyramidal cells of the hippocampus (CA1, CA2 and CA3 areas), in the striatum and in several cortical areas [381].

**11. *Pde1b1* (Phosphodiesterase 1B, Calmodulin-Dependent)**

**ABA review:** Most ventral cortical cells (base of layer VI) were labeled and a thin sheet of layer Va. There was strong and specific label in striatum/accumbens and also cells strewn in the basal amygdaloid region. Dentate granule cells were positive, as were cells in the postero-ventral cortex. Low expression could be found in several nuclei.

Sagittal section

**GENSAT**: Two BAC lines have matching expression at adult and the adult BAC data is consistent with the literature. The highest level of expression was observed in caudate putamen, olfactory tubercle and nucleus accumbens. At P7, scattered glial cells are transiently labeled in brainstem area. The E15-5 Bac data matches the *in situ* data from Genepaint. There are 24 regions of moderate-to-strong expression in the adult: amygdala, anterior olfactory nucleus, basal forebrain, caudate putamen, cerebellum, cerebral cortex, entorhinal cortex, globus pallidus, hippocampus, hypothalamus, inferior cerebellar peduncle, medulla, midbrain, olfactory bulb, optic tract, piriform cortex, pons, septum, spinal cord dorsal horn, spinal cord ventral horn, stria medullaris thalami, substantia nigra, thalamus, and ventral striatum.

**Literature:**  This gene is also called *Pde1b* or *Hspde1b1* in the literature. SAGE analysis revealed regional enrichment of *Pde1b* in the caudate-putamen of adult C57BL/6 mice [10]. *In situ* hybridization demonstrated high levels of *Pde1b1* mRNA in the caudate-putamen, nucleus accumbens, and olfactory tubercle in the mouse brain [382]. Moderate mRNA levels were observed in dentate gyrus, cerebral cortex, medial thalamic nuclei, and brainstem of mouse brain. Immunocytochemistry showed that majority of protein was localized to the caudate-putamen, nucleus accumbens, and olfactory tubercle. Microarray analysis revealed *Pde1b1* is regionally enriched in the adult mouse striatum [11]. In the human brain, the only detectable signals for *PDE1B1* were in the caudate nucleus and putamen; lower levels of signal were observed in other brain regions [383]. Null mutant mice exhibit exaggerated locomotor hyperactivity and phosphorylation of DARPP-32 (a signal transduction pathway component) in striatal slices in response to dopamine agonists, and display impaired spatial learning [384].

**12. *Pdyn* (Prodynorphin)**

**ABA review:** Moderate-to-high labeling could be seen throughout the caudoputamen. A slightly higher level of labeling was present in the nucleus accumbens and layers 1 and 2 of the olfactory tubercle. Very high levels of expression could be seen in the lateral segment of the central amygdalar nucleus and in the ventomedial hypothalamic nucleus. Sparse moderate-to-high expression could also be seen in the zona incerta. Moderate expression was also present in layers 2/3 and 4 of the cerebral cortex. There also appeared to be similar levels of expression in the granular cell layer of the cerebellum. It should also be noted that significant background expression could be seen throughout the brain. An independent review indicated strong expression in the striatum, and moderately dense, but high general background.

Sagittal section Coronal section

**GENSAT**: Two BAC lines share identical expression at P7. The data is consistent with the literature and *in situ* hybridization data.

**Literature:** An abundance of mRNAs is found in the nucleus accumbens and striatum of mouse brain [385]. In the human neostriatum there is heterogenous expression: high in the patch, but low in the matrix compartment. The prodynorphin patch/matrix mRNA expression was elevated in the caudate nucleus of suicide subjects as compared to normal controls [386].

**13. *Rarb* (Retinoic Acid Receptor, Beta)**

**ABA review:** There was nice specific label in the striatum/accumbens. Some specific sets of cortical neurons were labeled such as in piriform cortex, but overall this is quite specific to striatum.

Sagittal section Coronal section

**GENSAT:** No information available

**Literature:** *Rarb* mRNA is enriched in the striatum of adult mice [387]. Homozygous null mice are growth-deficient, but are fertile and have normal longevity [388]. They display homeotic transformations and malformations of cervical vertebrae and a retrolenticular membrane. In another study it was shown that null mutations of *Rarb* gene resulted in reduction of striatal-enriched tyrosine phosphatase (STEP) mRNA in the striatum of mutant mice [389].

**14*. Rasd2* (GTP-Binding Protein Rhes)**

**ABA review:** In the main olfactory bulb there was moderate expression in the glomerular layer, but moderate-to-high levels were observed in the mitral layer. Sparse punctate staining could be seen in layers 2/3 and 4 of the cerebral cortex. Very high expression was present in layer 2 of the olfactory tubercule. Moderate-to-high labeling was present in the nucleus accumbens and caudoputamen and in the reticular nucleus of the thalamus

In the hippocampal formation there was moderate expression along the length of the Ammon’s horn. Sparse moderate level labeling could also be seen in the midbrain and brainstem. In particular, there was a higher density of expression in the ventral segments of the medulla. An independent review indicated that this gene is expressed in numerous cells at reasonable levels, highest in striatum and accumbens and mitral cells of olfactory bulb and hippocampal pyramidal cells.

Sagittal section Coronal section

**GENSAT:** No information available

**Literature:** This gene is also referred to as *Rhes* and *Se6c* in the literature. SAGE analysis revealed regional enrichment of *Rasd2* in the caudate-putamen of adult C57BL/6 mice [10]. In the rat brain, *Se6c* mRNA was prominently detected in caudate nucleus, putamen, olfactory tubercle, and additionally, in parietal cortex (layers II, III, IV, and VI) [390]. Full gene expression in rat striatum is dependent upon thyroid hormone availability [391]. Western blot showed dimunition of rhes protein in striatal extracts of null mutant mice compared to WT mice [392]. Homozygous *Rhes* null mice show behavioral abnormalities, displaying a gender-dependent increase in anxiety levels and a clear motor coordination deficit but no learning or memory impairment [392].

**15. *Tgfa* (Transforming Growth Factor, Alpha)**

**ABA review:** In the main olfactory bulb there was moderate expression in the glomerular layer and moderate-to-high levels in the mitral layer. Sparse punctate staining was present in the cerebral cortex. Moderate-to-high labeling for *Tgfa* was found in the nucleus accumbens and caudoputamen. An independent review indicated nice and specific, albeit low, expression in the striatum/accumbens with a specific subset of cells that were labeled. Mitral cells in olfactory bulb and dentate gyrus granule cells were also labeled.

Sagittal section

**GENSAT:** No information available

**Literature:** In the rat forebrain, expression is found in the olfactory bulb, caudate-putamen, nucleus accumbens, olfactory tubercle, ventral pallidum, amygdala, hippocampal stratum granulosum and CA3 stratum pyramidale, and piriform, entorhinal, and retrosplenial cortices. Additionally, expression is detected in thalamic nuclei, suprachiasmatic, dorsomedial, and ventromedial nuclei of the hypothalamus. In addition, labeled cells were present in regions of white matter including the corpus callosum, anterior commissure, internal and external capsules, optic tract, and lateral olfactory tract [393]. Enzyme immunoassays demonstrated that there are significantly higher concentrations of TGF-alpha and other cytokines in the dopaminergic striatal regions in parkinsonian patients than in controls. This suggests that these cytokines may be produced as compensatory responses in the nigrostriatal dopaminergic regions in Parkinson disease [394].

**Subanatomical region: substantia nigra, Therapeutic interest: dopamine system, parkinson disease**

The substantia nigra lies in the midbrain and is a major element of the basal ganglia system. The substantia nigra compacta and ventral tegmental area are responsible for dopamine production in the brain, and therefore play a vital role in reward and addiction. Dopamine, normally synthesized by the dopaminergic neurons of the substantia nigra, passes via axoplasmic flow to the nerve terminals in the striatum, where it is released as a transmitter. Dopaminergic neuronal death in the SN leads to Parkinson disease.

**1*. Ddc* (Dopa Decarboxylase)**

**ABA review:** Very discrete expression was found in catecholaminergic neurons (dorsal raphe, locus coeruleus), including ventral tegmental area and substantia nigra. It was also nicely expressed in some hypothalamic nuclei, paraventricular and others, lateral habenula. In an independent review, it was indicated that there was strong expression in the substantia nigra (pars compacta), the ventral tegmental area, and parasubthalamic nucleus. Somewhat lower levels were also observed in the dorsal and central linear raphe nuclei and potentially the laterodorsal tegmental nucleus.

Sagittal section Coronal section Coronal section (zoomed)

**GENSAT:** *In situ* hybridization data from Allen Brain Atlas supports the literature and reveals some other expression sites, such as hypothalamus, main olfactory bulb and brainstem. Two BAC transgenic lines are identical at P7. The BAC data produces expression in the above areas. Blood vessel staining is also confirmed by the literature. However, the BAC detects additional expression in caudate putamen, ventral striatum and cerebellum. Staining in the cortex and hippocmpus is predominantly in fibers

**Literature:** SAGE analysis revealed regional enrichment of *Ddc* in the ventral tegmental area of the adult C57BL/6 mouse [10]. Microarray analysis revealed regional enrichment of *Ddc* in the adult rat substantia nigra [15]. A 3.6 kb human *AADC* (aromatic amino acid decarboxylase; another synonym for *Ddc*) promoter drives *lacZ* gene expression in substantia nigra, ventral tegmental area, and the dorsal, medial and pontine raphe nuclei of transgenic mice [395].

**2*. Slc6a3* (Solute Carrier Family 6, Member 3; Dopamine Transporter)**

**ABA review:** There was strong and specific expression in both DA cells in ventral tegmental area and substantia nigra. In an independent review it was found that expression is in midbrain motor-related areas, including, superior colliculus, substantia nigra, ventral tegmental area, midbrain reticular nucleus, retrorubral area, periaqueductal grey, red nucleus oculormotor nucleus, and in midbrain behavioural related areas such as substantia nigra, pedunculopontine and midbrain raphe nuclei.

Sagittal section Coronal section

**GENSAT:** No information available

**Literature:** This gene is also called *Dat*. SAGE analysis revealed regional enrichment of *Slc6a3* in the ventral tegmental area of the adult C57BL/6 mouse [10]. *In situ* hybridization revealed intense labeling in the dopamine cell body regions of the substantia nigra pars compacta/ventral tegmental area in the rat brain [396]. Microarray analysis revealed *Slc6a3* is regionally enriched in the adult mouse midbrain [11] and the adult rat substantia nigra [15]. A knock-in strategy has been used to generate mice expressing Cre recombinase under the control of the mouse dopamine transporter promoter (*Dat*-cre mice) [352]. In *Dat*-*cre* mice, immunocytochemical staining revealed that virtually all dopaminergic neurons in the ventral midbrain expressed *Cre*. In control human brains, there is intense *DAT* mRNA expression in the ventral midbrain with no significant difference in mRNA concentrations among the four regions studied [397]. In the Parkinson disease brains, there is an overall decrease in the intensity of *DAT* mRNA expression in the surviving dopaminergic neurons.

**3. *Ntsr1* (Neurotensin Receptor 1)**

**ABA review:** Besides few sets of neurons in diagonal band of Broca rostrally and medulla posteriorly, there was very nice expression in the ventral tegmental area and the substantia nigra. An independent review indicated high and specific expression in the substantia nigra (pars compacta) and ventral tegmental area. Slightly lower labeling was also seen in midbrain reticular nucleus (retrorubral area).

Sagittal section Coronal section

**GENSAT:** BAC data is consistent with the literature in general, partially matches the BGEM *in situ* data, and reveals strong Ntsr expression in thalamus during postnatal development. However, confocal images show that fiber projections are mostly stained.

**Literature:** *Ntsr1* is expressed in the mouse periaqueductal gray and the rostral ventral medulla [398]. In another study, *in situ* hybridization revealed expression in hippocampus, amygdala, hypothalamus, and cortex; moderate expression is observed in the thalamic region [399]. No hybridization signal was detected in any of the sections from null mutant mice.

**4. *Pitx3* (Paired-like Homeodomain Transcription Factor 3)**

**ABA review:** No ABA images available.

**GENSAT:** No information available

**Literature:** In the rat brain, *Pitx3* expression completely overlapped with that in TH+ cells indicating that *Pitx3* is expressed in dopaminergic neurons of the mesencephalic dopaminergic system [400]. The number of *Pitx3*-expressing neurons is reduced in Parkinson patients and these neurons are absent from 6-hydroxy-dopamine-lesioned rats, an animal model for this disease. It is noteworthy that there is early developmental failure of substantia nigra dopamine neurons in aphakia mutant mice lacking this gene [401]. In aphakia mutant mice, a deletion of 652 bp located 2.5 kb upstream of the 5' UTR sequence of *Pitx3* results in diminished expression at all sites [402].

**5. *Aldh1a1* (Aldehyde Dehydrogenase 1 Family, Member A1)**

**ABA review:** There was high and specific expression in the ventral tegmental area and the substantia nigra (compacta and reticular parts). Slightly lower labeling was also seen in midbrain reticular nucleus (retrorubral area). In addition, there was low-to-moderate expression in the cortical subplate (claustrum) and in the cerebellar Purkinje cells.

Another review indicated very strong and specific expression in the ventral tegmental area and substantia nigra. There was only very light and discrete signals elsewhere, such as a few cells deep in the rostral cortex and the cerebellar Bergmann glia.

Sagittal section Coronal section Coronal section (zoomed)

**GENSAT:** No information available

**Literature:** This gene is also called *Aldh1*. The promoter region of the mouse *Aldh1* shows high sequence similarity with human *ALDH1* [403]. SAGE analysis revealed regional enrichment of *Aldh1a1* in the ventral tegmental area of the adult C57BL/6 mouse [10]. No *Aldh1* expression has been detected in mouse brain by northern analysis. *ALDH1* was found to be expressed highly and specifically in dopaminergic cells of both substantia nigra and the ventral tegmental area of the human brain [404].

**6. *Chrna6* (Cholinergic Receptor, Nicotinic, Alpha Polypeptide 6)**

**ABA review:** There was high expression in the substantia nigra (pars compacta) and the ventral tegmental area. Interestingly similar levels were also observed in the locus ceruleus and in the pontine central gray. Somewhat lower levels were also observed in the central linear raphe nuclei and the midbrain reticular nucleus (retrorubral area).

Another review indicated that there was label in superficial layers of superior colliculus, dopaminergic neurons of substantia nigra and ventral tegmental area, and layer 5 pyramids in cortex.

Sagittal section Coronal section Coronal (zoomed)

**GENSAT:** There are six regions of moderate-to-strong expression in the adult: caudate putamen, midbrain, optic tract, substantia nigra, thalamus, and ventral tegmental area. The BAC data is in agreement with the literature and BGEM *in situ* data. One discrepancy between the BAC data and the literature is that the locus coeruleus is not labeled in adult BAC mice. The BAC also shows additional expression in striatum. The BAC used by GENSAT (RP23-133K10) includes both *Chrna6* and *Chrnb3*.

**Literature:** SAGE analysis revealed regional enrichment of *Chrna6* in the ventral tegmental area of the adult C57BL/6 mouse [10]. Microarray analysis revealed *Chrna6* regional enrichment in the adult rat substantia nigra [15]. In the rat brain, expression is restricted to and high in the locus coeruleus, ventral tegmental area and substantia nigra; lower levels are found in reticular thalamic nucleus, supramammillary nucleus and mesencephalic V nucleus; some cells of medial habenula (medioventral part) and of the interpeduncular nucleus (central and lateral parts) also labeled [405]. It has also been reported that in the human brain, immunoreactivity is found in the cerebellum [406].

**7. *Chrnb3* (Cholinergic Receptor, Nicotinic, Beta 3)**

**ABA review:** There was expression in the substantia nigra (pars compacta), interpeduncular nucleus, ventral tegmental area, also in midbrain reticular nucleus (retrorubral area), pontine central gray, and medial habenula in the epithalamus. There was also low-to-moderate expression in the dorsal region of the superior colliculus.

An independent review indicated that there was a strong signal in the cells of the medial habenula, a one-cell thick set of cells in superficial layer 5 of cortex, superficial cell layer of superior colliculus, cells of the substantia nigra, and in more posterior sections in the ventral tegmental area. In the brainstem, cells appeared positive in either the locus coeruleus or Barrington's nucleus.

Sagittal section Coronal section Coronal (zoomed)

**GENSAT:** The BAC used by GENSAT (RP24-149I12) includes both *Chrna6* and *Chrnb3*. The BAC data is reproducible, matches the BGEM *in situ* data, and reveals extra expression in optic chiasm and superior colliculus. Expression in the superior colliculus is confirmed by ABA *in situ* hybridization data. However, the BAC misses expression in locus coeruleus

**Literature:** There is restricted distribution of expression in the substantia nigra and ventral tegmental area of the rat brain [407]. In another study, it was reported that within the substantia nigra of the rat brain, almost all dopamine neurons express this gene [408]. A third publication reported that antibodies to Chrnb3 subunit detect receptor-binding activity in the striatum [409]. Deficits in striatal binding are associated with major losses of Chrnb3 subunits (by around 75%) in Parkinson disease. In *Chrnb3* (+/+) mice strong expression of *Chrnb3* mRNA was detected in the substantia nigra, ventral tegmental area, and medial habenula, with weaker labeling in the superior colliculi and a subset of the medial vestibular nuclei [410]. On the other hand decreased expression was observed in *Chrnb3* (+/-) mice, whereas no specific labeling was detected in *Chrnb3* (-/-) mice, verifying disruption of the gene.

**8. *Th* (Tyrosine Hydroxylase)**

**ABA review**: High levels of labeling were found in the substantia nigra (pars compacta), the ventral tegmental area, parasubthalamic nucleus, locus ceruleus and parabrachial nucleus. Somewhat lower levels were also observed in the dorsal and central linear raphe nuclei, posterior hypothalamic nucleus and the nucleus of Darkschewitsch. In addition, very high expression was observed in the main olfactory bulb (especially in the glomerular and granular layers and molecular and pyramidal layers of the nucleus of the lateral olfactory tract. Low diffuse expression could be seen throughout the cerebral nuclei, white matter tracts and layer 6a of the cerebral cortex.

An independent review indicated expression of this gene in all classic TH+ cells such as locus coeruleus, olfactory bulb, medulla, hypothalamus, ventral tegmental area and substantia nigra, including ectopic expression in P cells.

Sagittal section Coronal section Coronal (zoomed)

**GENSAT:** There are 20 regions of strong to moderate expression in the adult. The BAC data is consistent with the literature and BGEM *in situ* data. The confirmed expression sites include main olfactory bulb, striatum, substantia nigra, ventral tegmental area and raphe nuclei. One paper reports the presence of *Th* mRNA in amygdala and ventral thalamus. These expression sites also appear in the BAC data. The BAC detects extra expression sites such as hypothalamus and ventral striatum, which are not confirmed by the *in situ* data, but are reproducible among BAC lines.

**Literature:** Tyrosine hydroxylase is a rate-limiting enzyme in catecholamine biosynthesis. SAGE analysis revealed regional enrichment of *Th* in the ventral tegmental area of the adult C57BL/6 mouse [10]. In adult transgenic mice, 4.5 kb of the rat *Th* promoter drove human placental alkaline phosphatase (AP) expression in essentially all *Th*-expressing cell groups throughout development and in adults [411]. In another study, in transgenic mice bearing 9.0 kb of rat *Th* 5' flanking sequence fused to *lacZ* high level expression of beta-galactosidase were found at levels equivalent to the endogenous *Th* in central catecholaminergic cells [412].

**Subanatomical region: Subthalamic nucleus, Therapeutic Interest: Pain**

The subthalamic nucleus (STN) is a part of the basal ganglia which also includes the substantia nigra, striatum, external segment of the globus pallidus, and internal segment of the globus pallidus [413]. In humans, the STN contains primarily glutamatergic neurons with a smaller population of GABAergic interneurons. The STN plays an important role in the planning, execution and motivational/ emotional aspects of movement. Lesions in this brain region have been shown to result in hemiballism which is characterized by violent involuntary movements of the contralateral limbs [414]. Currently, deep brain stimulation of the STN is the most common therapeutic strategy for patients with Parkinson disease with failed medical management [415].

**1) *Pitx2* (Pituitary Homeobox 2)**

**ABA review:** Examination of images indicated that there was moderate expression of *Pitx2* in the subthalamic nucleus. In the hypothalamus there was low level expression in the supramammillary nucleus, medial mammillary nucleus posterior hypothalamic nucleus and in the posterior portion of the periventricular hypothalamic nucleus. Within the midbrain there moderate labeling was present in the superior colliculus, motor related, deep gray layer. In some slides moderate levels of expression could be found in the olfactory nerve layer of main olfactory bulb. Finally, scattered low level expression was observed throughout the cerebellum.

Sagittal section Coronal section

**GENSAT:** No information available.

**Literature:** The gene is also called *Ptx2* in the literature. All *Ptx2* transcript variants display an identical and markedly restricted expression in the anterior and intermediate lobes of the pituitary gland, the subthalamic nucleus, the posterior hypothalamic nucleus, the mammillary bodies, the red nucleus, and the deep gray layer of the superior colliculus [416]*.* In the adult mouse brain, *Ptx2* is coexpressed with Lmx1b in the subthalamic nucleus, posterior hypothalamus and some, but not all, mammillary nuclei [417]. *Pitx2* is required for normal development of neurons in the mouse subthalamic nucleus and midbrain. However, closer examination of the expression data did not uphold this account because many neurons are labeled in the anterior brain, hippocampal cells and some select hypothalamic nuclei [418].

**2) *Lmx1b* (LIM Homeobox Transcription Factor 1 Beta)**

**ABA review:** Moderate expression of *Lmx1B* can be found in the subthalamic nucleus. Within the pons moderate levels of expression can be seen in the superior central nucleus raphe. Similar levels of labeling can be observed in the posterior portion of the periventricular hypothalamic nucleus. Weak but specific expression is present in the midbrain dorsal nucleus raphe and the central linear nucleus raphe.

Sagittal section Coronal section

**GENSAT:** No information available.

**Literature:** *In situ* hybridization studies in the adult mouse brain detected *Lmx1b* in the subthalamic nucleus, the posterior hypothalamus, premammillary nucleus, and supramammillary nucleus, substantia nigra, ventral tegmental area; the interpeduncular nucleus contained *Lmx1b*-expressing cells, raphe nuclei, parabrachial nucleus and the pontine reticular nucleus, trigeminal nucleus and the dorsal horn [35]. *Lmx1b* knockout mice fail to induce the mesencephalic dopamine system-specific homeodomain gene Ptx3 in TH+ neurons. Eventually, this small set of TH+ neurons are lost during embryonic maturation [419].

**Subanatomical region: Thalamus, Therapeutic interest: Huntington Disease**

The thalamus constitutes the main part of the diencephalon and is deeply situated in the forebrain. It has sensory and motor functions, and axons from every sensory system (except olfaction) synapse here, as the last relay site before the information reaches the cerebral cortex. The thalamus functions as a translator where various sensory inputs are processed into a form readable by the cortex. Abnormalities within the thalamus result in schizophrenia, memory deficits, and Huntington disease (indirectly due to decreased activity of basal ganglia output) etc

**1. *Ramp3* (Receptor (Calcitonin) Activity Modifying Protein 3)**

**ABA review:** There was moderate-to- high expression throughout the thalamus. Expression appeared to be slightly higher in the dorsal region of the thalamus. In addition, there was moderate expression throughout layer 2 and the prelimbic region of layer 6a in the cerebral cortex. An independent review found gene expression in upper and lower cortex and area postrema. There was very strong expression in thalamus, including the reticular nucleus. This seemed to be the most intense and complete of labels in the posterior thalamus.

Sagittal section Coronal section

**GENSAT:** The BAC data is consistent with the literature and the *in situ* hybridization data from Allen Brain Atlas. The BAC produces high expression in the cerebellum at E15-5, but in scattered cerebellar cells at P7. The hippocampus is reproducibly stained in different Bac lines, mainly at P7. Adult *in situ* hybridization data from Allen database reveals the presence of *Ramp3* mRNA in deep layers of cortex and the adult BAC data has a few scattered cells weakly labeled in the area.

**Literature:** SAGE analysis revealed regionally enrichment of *Ramp3* in the thalamus of the adult C57BL/6 mouse [10]. Microarray analysis revealed *Ramp3* is regionally enriched in the adult mouse midbrain [11]. *Ramp3* is restrictively expressed in thalamic nuclei of the adult rat brain [420].

**2. *Rgs16* (Regulator of G-Protein Signalling 16)**

**ABA review:** There was strong expression in the suprachiasmatic nucleus, but very specific expression in thalamus (but not reticular nucleus). An independent review of the ABA images indicated very specific expression throughout the thalamus, but reduced expression along the midline regions (medial/lateral habenula, paraventricular nucleus, intermediodorsal nucleus, central medial nucleus, rhomboid nucleus, and nucleus of reunions).

Sagittal section Coronal section

**GENSAT:** There are 21 regions of strong to moderate expression in the adult. BAC data correlates with the literature in major expression sites. In adult BAC transgenic mice, strong expression is observed in thalamus, superior colliculus, basal forebrain, main olfactory bulb and hypothalamus. Weaker expression is also detected in cortex and cerebellum.

**Literature:** SAGE analysis revealed regional enrichment of *Rgs16* in the thalamus of the adult C57BL/6 mouse [10]. *Rgs16* is expressed predominantly in the thalamic midline/intralaminar and principal relay nuclei, and the hypothalamic suprachiasmatic nucleus of the rat brain [421].

**3. *Slitrk6* (SLIT and NTRK-Like Family, Member 6)**

**ABA review:** There was moderate expression in the lateral geniculate nucleus and other thalamic nuclei, with rather discrete, low background cell label. In an independent review, moderate expression was found throughout the thalamus. Slightly higher expression was observed in the ventral posterior complex and medial geniculate complex of the dorsal thalamus.

Sagittal section Coronal section

**GENSAT:** Two BAC transgenic mouse lines have identical expression at P7. The BAC produces expression broadly across the CNS and the overall expression pattern is consistent with BGEM *in situ* data. Compared to the BAC data, *in situ* hybridization indicated that in the cerebellum *Slitrk6* mRNA is expressed in granule cells in external granule cell layer and internal granule cell layer.

**Literature:** SAGE analysis revealed regional enrichment of *Slitrk6* in the thalamus of the adult C57BL/6 mouse [10]. It has been reported that in the E17 mouse brain, *Slitrk6* expression is restricted to the ventral thalamus and lateral geniculate nucleus, and expression is strong in the suprafascicular nucleus [422]. *Slitrk6* expression in the dorsal thalamus persists in the adult stage from the embryonic mouse stage [423]. In the normal human brain, *SLITRK6* expression is strongest in the putamen [424].

**4. *Tnnt1* (Troponin T Type 1; skeletal, slow)**

**ABA review:** Besides specific expression in the thalamic, other domains with strong expression included the Islands of Calleja and the superficial grey layer of superior colliculus. An independent review indicated high levels of expression in the thalamus, with somewhat higher expression in the ventral region. Within the pons, there was some weak, but specific labeling of cells, in the superior olivary complex and facial motor nucleus. Some diffuse expression was also observed throughout the midbrain.

Sagittal section

**GENSAT:** No information available

**Literature:** SAGE analysis revealed regional enrichment of *Tnnt1* in the thalamus of the adult C57BL/6 mouse [10]. Microarray analysis revealed *Tnnt1* is regionally enriched in the adult mouse midbrain [11].

**5*. 1110069I04Rik* (Hypothetical Protein)**

**ABA review:** There was some limited and light label in the Purkinje cell layer and cochlear nucleus, hippocampus, striatum and cortex. The most robust *in situ* signal was present in most thalamic areas from rostral to caudal, but not in the reticular nucleus.  In an independent review, it was noted that *1110069I04Rik* transcript is not enriched within the thalamus. There are moderate-to-high levels throughout the cerebral cortex, hippocampal formation, main olfactory bulb and cerebellum. Expression appeared to be lower in the cerebral nuclei and pallidum.

Sagittal section Coronal section

**GENSAT:** No information available

**Literature:** *In situ* hybridization and immunohistochemistry revealed a high constitutive expression of synaptopodin mRNA in the hippocampal formation [425].

**6. *Amotl1* (Angiomotin-Like Protein 1)**

**ABA review:** There was nice moderate-to-high expression throughout the thalamus. However, there appeared to be somewhat higher expression was observed in the ventral region. An independent review that gene expression is very specific to the thalamus including the antero-dorsal thalamus but these regions are less obvious in posterior thalamus.

Sagittal section Coronal section

**GENSAT:** No information available

**Literature:** SAGE analysis revealed regional enrichment of *Amotl1* in the thalamus of the adult C57BL/6 mouse [10].

**7. *Rab37* (RAB37, Member of RAS Oncogene Family)**

**ABA review:**  Within the thalamus *Rab37* appeared to be expressed in the medial and ventral segments and the subparafascicular/peripeduncular nuclei. In the medulla there was scattered expression in the external cuneate nucleus, the lateral reticular nucleus magnocellular part and to some extent the hypoglossal nucleus. In the pons there was significant labeling in the pontine gray, pontine reticular nucleus and tegmental reticular nucleus. Some expression was also present in the cerebellar white matter.

An independent review indicated some brainstem staining in XII nucleus, lateral reticular nucleus, and internal granule layer of cerebellum, Purkinje cells of parafloccular lobe, neurons in the pontine region and the red nucleus. The thalamic label was seen posteriorly in the medial geniculate, lateral geniculate and was very similar to *Lef1* but it decreases in the anterior thalamus. It also appeared to be absent in the reticular nucleus but present in ventro-posterior thalamus.

Sagittal section Coronal section

**GENSAT:** No information available

**Literature:** Microarray analysis revealed *Lef1* is regionally enriched in the adult mouse midbrain [11].

**8. *Sh3d19* (SH3 Domain Protein D19)**

**ABA review:** There was moderate-to-high expression throughout the thalamus. There also appeared to be specific expression in the pyramidal layer of the olfactory tract nucleus and scattered (but lower) expression throughout the cerebral cortex. An independent review indicated that this is a thalamus-specific gene with expression mainly in the anterior nuclei.

Sagittal section Coronal section

**GENSAT:** No information available

**Literature:** No relevant brain expression studies have been reported.

**9. *Grid2ip* (Glutamate Receptor, Ionotropic, Delta 2 (Grid2) Interacting Protein)**

**ABA review:** There was moderate-to-high expression throughout the thalamus and significant expression in the lateral septal nucleus and the main olfactory bulb. Purkinje cells are also very strongly labeled.

An independent review indicated strong expression in thalamic territory in the rostral brain, with the exception of the reticular nucleus. There was specific and strong label in all other thalamic nuclei from rostral to caudal extent. Other regions of expression include the ependyma of the olfactory extension of the ventricle, a peppering of cells in the olfactory bulb, a layer of neurons above the rhinal fissure in the orbital cortex, and the dorsal peduncular nuclei that spreads into the cells of the dorsal part of the lateral septal nuclei. Additionally, cells in layer 3-4 of cortex, cerebellar Purkinje cells, discrete brainstem nuclei in pons and superior olive are also labeled.

Sagittal section Coronal section

**GENSAT:** The gene is highly expressed in cerebellar Purkinje cells. Two BAC lines have very similar expression at P7. The BAC data matches both the literature and *in situ* hybridization data from Allen Brain Atlas. In the adult, cerebellar Purkinje cells are strongly stained in BAC transgenic mice. The BAC also produces expression in cortex, which is not obvious in ABA data. The lateral septum appears to be not expressed in BAC mice. In the P7 cerebellum, the BAC produces transient expression in the granule cells. In the E15-5 mouse spinal cord, the EGFP transgene is only expressed in lower spinal cord; the same data is obtained from two embryos.

**Literature:** *Grid2ip* is also referred to as delphilin. Delphilin is identified as a glutamate receptor delta2 (GluRdelta2) subunit interacting protein that is selectively expressed in cerebellar Purkinje cells [426]. *In situ* hybridization demonstrated the highest expression of delphilin mRNA in Purkinje cells of the adult mouse brain [427]. Weak but detectable expression was also observed in the cerebrum and the brainstem.

**10. *Lef1* (Lymphoid Enhancer Binding Factor 1)**

**ABA review:** There was moderate but very specific expression throughout the thalamus. An independent review indicated positive cells are rather limited in brain and are found in the medial geniculate, lateral geniculate and lateral posterior nuclei. Expression is relatively absent in posterior thalamus, the ventro-posterior cell groups, and also cells of the reticular nucleus throughout the extent of the thalamus.

Sagittal section Coronal section

**GENSAT:** Two BAC transgenic lines have overlapping expression at P7. The overall expression pattern correlates with the literature and BGEM *in situ* data. Confirmed expression sites include hypothalamus, hippocampus, cortex, thalamus and superior colliculus. In the thalamus, both the literature and BGEM data suggest that *Lef1* mRNA is present in most of the thalamic nuclei. The EGFP transgene is mainly confined to the laterodorsal group. In addition, the BAC reproducibly produces expression in blood vessels in two targeting events.

**Literature:** There is very specific expression in the dorsal thalamus [428, 429].

**11. *Plekhg1* (Pleckstrin Homology Domain Containing, Family G)**

**ABA review:** There was moderate-to-high expression throughout the thalamus and very little off-target labeling in other regions of the brain. An independent review indicated very strong and nice label in mid-thalamic region with the exception of the reticular nucleus. There was diminished staining in anterior and posterior thalamic nuclei.

Sagittal section Coronal section

**GENSAT:** No information available

**Literature:** No relevant brain expression studies have been reported.

**12. *Syt9* (Synaptotagmin 9)**

**ABA review:** There was moderate-to-high expression throughout the thalamus. There was labeling of cells in the mitral and glomerular layers of the main olfactory bulb, as well as in the cerebellar cortex. An independent review indicated uniform expression throughout thalamus (including the reticular nucleus); other positive areas include the amygdala and the medial habenula.

Sagittal section Coronal section

**GENSAT:** No information available

**Literature:** No relevant brain expression studies have been reported.

**13. *Tcf7l2* (Transcription Factor 7-Like 2)**

**ABA review:** There was strong expression in all nuclei throughout thalamus with the exception of reticular nucleus. There was also nice label in various regions of the periaqueductal gray. An independent review of the ABA images indicated high levels of expression in the thalamus. *Tcf7l2* is also expressed at similar levels in regions of the midbrain including inferior colliculus, anterior and posterior pretectal nuclei, nucleus of the optic tract, and the rostral potion of the midbrain reticular nucleus. In addition, the glomerular and mitral layers of the main olfactory bulb were also labeled.

Sagittal section Coronal section

**GENSAT:** No description available

**Literature:** This gene is also called *Tcf4* in the literature. High levels of *Tcf4* expression were particularly evident in the developing central nervous system. In the adult mouse, the gene was described as being expressed mainly in the cortex [430]. SAGE analysis revealed regional enrichment of *Tcf7l2* in the thalamus of the adult C57BL/6 mouse [10]. Microarray analysis revealed *Tcf7l2* is enriched in the adult mouse midbrain [11].

**14. Gm804 (gene model 804, (NCBI))**

**ABA review:** One review indicated moderate-to-high expression throughout the thalamus. Similar levels of expression could be seen in the cerebellar Purkinje cell layer, the glomerular, mitral and granular cell layers of the main olfactory bulb. Another review indicated strong expression in the thalamic nuclei, but there also is expression particularly in the cortex.

Sagittal section

**GENSAT:** No description available.

**Literature:** SAGE analysis revealed regional enrichment of Gm804 in the thalamus of the adult C57BL/6 mouse [10].

**15. *Gja7* (Gap junction alpha-7 protein)**

**ABA review:** One review indicated moderate levels of expression almost exclusively in the thalamus within the anteroventral nucleus (dorsal segment), anteromedial and reticular nuclei. Similar expression levels were also present throughout the ventral posterior complex of the thalamus. There also appeared to be strong staining in the granule layer of the accessory olfactory nucleus. Another review indicated expression that is very specific to thalamic nuclei; label is moderately heavy and is in many nuclei, including both geniculate bodies, VPL/M and anterior nuclei. Background expression was found in small cells in cortex and striatu

Sagittal section Coronal section

**GENSAT:** No description available

**Literature:** This gene is also caled Connexin45. In mice, the connexin45 gene is highly expressed during embryogenesis and up to 2 weeks after birth in nearly all brain regions. Afterward its expression is restricted to the thalamus, the CA3 region of hippocampus and the cerebellum. In adult mouse brain, the pattern of LacZ-staining in combination with the analysis of different neuronal and glial marker proteins strongly suggests that connexin45 is expressed in neurons [431]. Double immunofluorescent staining using specific antibodies to connexin45 and connexin32 paired with cell-type specific marker proteins revealed that connexin45 and connexin32 were co-expressed and colocalized in oligodendrocytes of the rat hippocampus [432].

**16. Socs6 (Suppressor of cytokine signaling 6)**

**ABA review:** One review indicated low-to-moderate level expression throughout the brain. However, labeling of SOCS6 was markedly reduced in the white matter tracts. Moderate but dense staining was present throughout the thalamus. Similar levels of expression were present in the cells lining the ventricles. Low-to-moderate expression was observed throughout the pons and medulla; however, expression was somewhat higher in the inferior olivary complex. There was also moderate-to-high labeling in the cerebellar Purkinje cell layer. Another review indicated background expression, but standing out above background was label in most nuclei of thalamus.

Sagittal section Coronal section

**GENSAT:** No description available

**Literature:** No expression studies were found.

**17. Vangl1 (vang, van gogh-like 1 (Drosophila)**

**ABA review:** Low-to-moderate expression was found in the ventral group of the dorsal thalamus including the ventral anterior-lateral complex of the thalamus and ventral medial nucleus of the thalamus. A similar level of labeling was found in the geniculate group of the dorsal thalamus which included the dorsal part of the lateral geniculate complex and medial geniculate complex. Low levels of less dense punctate staining of were located in the ventral posterior complex of the thalamus, reticular nucleus of the thalamus and the medial and lateral groups of the dorsal thalamus. Virtually no expression could be found in the intralaminar nuclei of the dorsal thalamus and the midline group of the dorsal thalamus. Vangl1 staining was absent in the rest of the brain with the exception of a few positive cells in the layer 2 of the cerebral cortex.

Sagittal section Coronal section

**GENSAT:** No description available

**Literature:** No expression studies were found.

**References**

1. Amunts K, Kedo O, Kindler M, Pieperhoff P, Mohlberg H, Shah NJ, Habel U, Schneider F, Zilles K: **Cytoarchitectonic mapping of the human amygdala, hippocampal region and entorhinal cortex: intersubject variability and probability maps**. *Anat Embryol (Berl)* 2005, **210**(5-6):343-352.

2. Abu-Abed S, MacLean G, Fraulob V, Chambon P, Petkovich M, Dolle P: **Differential expression of the retinoic acid-metabolizing enzymes CYP26A1 and CYP26B1 during murine organogenesis**. *Mech Dev* 2002, **110**(1-2):173-177.

3. Li SH, Yu ZX, Li CL, Nguyen HP, Zhou YX, Deng C, Li XJ: **Lack of huntingtin-associated protein-1 causes neuronal death resembling hypothalamic degeneration in Huntington's disease**. *J Neurosci* 2003, **23**(17):6956-6964.

4. Dragatsis I, Dietrich P, Zeitlin S: **Expression of the Huntingtin-associated protein 1 gene in the developing and adult mouse**. *Neurosci Lett* 2000, **282**(1-2):37-40.

5. Sugino K, Hempel CM, Miller MN, Hattox AM, Shapiro P, Wu C, Huang ZJ, Nelson SB: **Molecular taxonomy of major neuronal classes in the adult mouse forebrain**. *Nat Neurosci* 2006, **9**(1):99-107.

6. Persohn E, Malherbe P, Richards JG: **Comparative molecular neuroanatomy of cloned GABAA receptor subunits in the rat CNS**. *The Journal of comparative neurology* 1992, **326**(2):193-216.

7. Honda S, Kagoshima M, Wanaka A, Tohyama M, Matsumoto K, Nakamura T: **Localization and functional coupling of HGF and c-Met/HGF receptor in rat brain: implication as neurotrophic factor**. *Brain research* 1995, **32**(2):197-210.

8. Jung W, Castren E, Odenthal M, Vande Woude GF, Ishii T, Dienes HP, Lindholm D, Schirmacher P: **Expression and functional interaction of hepatocyte growth factor-scatter factor and its receptor c-met in mammalian brain**. *The Journal of cell biology* 1994, **126**(2):485-494.

9. Stansberg C, Vik-Mo AO, Holdhus R, Breilid H, Srebro B, Petersen K, Jorgensen HA, Jonassen I, Steen VM: **Gene expression profiles in rat brain disclose CNS signature genes and regional patterns of functional specialisation**. *BMC genomics* 2007, **8**:94.

10. Brochier C, Gaillard MC, Diguet E, Caudy N, Dossat C, Segurens B, Wincker P, Roze E, Caboche J, Hantraye P *et al*: **Quantitative gene expression profiling of mouse brain regions reveals differential transcripts conserved in man and affected in disease models**. *Physiological genomics* 2008.

11. Zapala MA, Hovatta I, Ellison JA, Wodicka L, Del Rio JA, Tennant R, Tynan W, Broide RS, Helton R, Stoveken BS *et al*: **Adult mouse brain gene expression patterns bear an embryologic imprint**. *Proceedings of the National Academy of Sciences of the United States of America* 2005, **102**(29):10357-10362.

12. Garcia MM, Cusick CG, Harlan RE: **Protein kinase C-delta in rat brain: association with sensory neuronal hierarchies**. *The Journal of comparative neurology* 1993, **331**(3):375-388.

13. Chesselet MF, Weiss L, Wuenschell C, Tobin AJ, Affolter HU: **Comparative distribution of mRNAs for glutamic acid decarboxylase, tyrosine hydroxylase, and tachykinins in the basal ganglia: an in situ hybridization study in the rodent brain**. *The Journal of comparative neurology* 1987, **262**(1):125-140.

14. Davidson S, Miller KA, Dowell A, Gildea A, Mackenzie A: **A remote and highly conserved enhancer supports amygdala specific expression of the gene encoding the anxiogenic neuropeptide substance-P**. *Molecular psychiatry* 2006, **11**(4):323, 410-321.

15. Fang H, Tong W, Shi L, Jakab RL, Bowyer JF: **Classification of cDNA array genes that have a highly significant discriminative power due to their unique distribution in four brain regions**. *DNA and cell biology* 2004, **23**(10):661-674.

16. Shumyatsky GP, Tsvetkov E, Malleret G, Vronskaya S, Hatton M, Hampton L, Battey JF, Dulac C, Kandel ER, Bolshakov VY: **Identification of a signaling network in lateral nucleus of amygdala important for inhibiting memory specifically related to learned fear**. *Cell* 2002, **111**(6):905-918.

17. Su BY, Cai WQ, Zhang CG, Martinez V, Lombet A, Perbal B: **The expression of ccn3 (nov) RNA and protein in the rat central nervous system is developmentally regulated**. *Mol Pathol* 2001, **54**(3):184-191.

18. Lopes da Silva S, Cox JJ, Jonk LJ, Kruijer W, Burbach JP: **Localization of transcripts of the related nuclear orphan receptors COUP-TF I and ARP-1 in the adult mouse brain**. *Brain research* 1995, **30**(1):131-136.

19. Cano G, Card JP, Rinaman L, Sved AF: **Connections of Barrington's nucleus to the sympathetic nervous system in rats**. *J Auton Nerv Syst* 2000, **79**(2-3):117-128.

20. Sved AF, Cano G, Passerin AM, Rabin BS: **The locus coeruleus, Barrington's nucleus, and neural circuits of stress**. *Physiol Behav* 2002, **77**(4-5):737-742.

21. Rouzade-Dominguez ML, Pernar L, Beck S, Valentino RJ: **Convergent responses of Barrington's nucleus neurons to pelvic visceral stimuli in the rat: a juxtacellular labelling study**. *The European journal of neuroscience* 2003, **18**(12):3325-3334.

22. Pavcovich LA, Yang M, Miselis RR, Valentino RJ: **Novel role for the pontine micturition center, Barrington's nucleus: evidence for coordination of colonic and forebrain activity**. *Brain Res* 1998, **784**(1-2):355-361.

23. Keegan CE, Herman JP, Karolyi IJ, O'Shea KS, Camper SA, Seasholtz AF: **Differential expression of corticotropin-releasing hormone in developing mouse embryos and adult brain**. *Endocrinology* 1994, **134**(6):2547-2555.

24. Weickert CS, Kittell DA, Saunders RC, Herman MM, Horlick RA, Kleinman JE, Hyde TM: **Basic fibroblast growth factor and fibroblast growth factor receptor-1 in the human hippocampal formation**. *Neuroscience* 2005, **131**(1):219-233.

25. Belluardo N, Wu G, Mudo G, Hansson AC, Pettersson R, Fuxe K: **Comparative localization of fibroblast growth factor receptor-1, -2, and -3 mRNAs in the rat brain: in situ hybridization analysis**. *The Journal of comparative neurology* 1997, **379**(2):226-246.

26. Sarter M, Bruno JP, Givens B: **Attentional functions of cortical cholinergic inputs: what does it mean for learning and memory?** *Neurobiol Learn Mem* 2003, **80**(3):245-256.

27. Dubelaar EJ, Mufson EJ, ter Meulen WG, Van Heerikhuize JJ, Verwer RW, Swaab DF: **Increased metabolic activity in nucleus basalis of Meynert neurons in elderly individuals with mild cognitive impairment as indicated by the size of the Golgi apparatus**. *J Neuropathol Exp Neurol* 2006, **65**(3):257-266.

28. Del Tredici K, Rub U, De Vos RA, Bohl JR, Braak H: **Where does parkinson disease pathology begin in the brain?** *J Neuropathol Exp Neurol* 2002, **61**(5):413-426.

29. Gentleman SM, Falkai P, Bogerts B, Herrero MT, Polak JM, Roberts GW: **Distribution of galanin-like immunoreactivity in the human brain**. *Brain Res* 1989, **505**(2):311-315.

30. Counts SE, Chen EY, Che S, Ikonomovic MD, Wuu J, Ginsberg SD, Dekosky ST, Mufson EJ: **Galanin fiber hypertrophy within the cholinergic nucleus basalis during the progression of Alzheimer's disease**. *Dement Geriatr Cogn Disord* 2006, **21**(4):205-214.

31. Mufson EJ, Bothwell M, Hersh LB, Kordower JH: **Nerve growth factor receptor immunoreactive profiles in the normal, aged human basal forebrain: colocalization with cholinergic neurons**. *The Journal of comparative neurology* 1989, **285**(2):196-217.

32. Mufson EJ, Counts SE, Ginsberg SD: **Gene expression profiles of cholinergic nucleus basalis neurons in Alzheimer's disease**. *Neurochem Res* 2002, **27**(10):1035-1048.

33. Marksteiner J, Sperk G, Krause JE: **Distribution of neurons expressing neurokinin B in the rat brain: immunohistochemistry and in situ hybridization**. *The Journal of comparative neurology* 1992, **317**(4):341-356.

34. Chawla MK, Gutierrez GM, Young WS, 3rd, McMullen NT, Rance NE: **Localization of neurons expressing substance P and neurokinin B gene transcripts in the human hypothalamus and basal forebrain**. *The Journal of comparative neurology* 1997, **384**(3):429-442.

35. Asbreuk CH, van Schaick HS, Cox JJ, Kromkamp M, Smidt MP, Burbach JP: **The homeobox genes Lhx7 and Gbx1 are expressed in the basal forebrain cholinergic system**. *Neuroscience* 2002, **109**(2):287-298.

36. Zhao Y, Marin O, Hermesz E, Powell A, Flames N, Palkovits M, Rubenstein JL, Westphal H: **The LIM-homeobox gene Lhx8 is required for the development of many cholinergic neurons in the mouse forebrain**. *Proceedings of the National Academy of Sciences of the United States of America* 2003, **100**(15):9005-9010.

37. Fragkouli A, Hearn C, Errington M, Cooke S, Grigoriou M, Bliss T, Stylianopoulou F, Pachnis V: **Loss of forebrain cholinergic neurons and impairment in spatial learning and memory in LHX7-deficient mice**. *The European journal of neuroscience* 2005, **21**(11):2923-2938.

38. Valdenaire O, Richards JG, Faull RL, Schweizer A: **XCE, a new member of the endothelin-converting enzyme and neutral endopeptidase family, is preferentially expressed in the CNS**. *Brain research* 1999, **64**(2):211-221.

39. Kiryu-Seo S, Sasaki M, Yokohama H, Nakagomi S, Hirayama T, Aoki S, Wada K, Kiyama H: **Damage-induced neuronal endopeptidase (DINE) is a unique metallopeptidase expressed in response to neuronal damage and activates superoxide scavengers**. *Proceedings of the National Academy of Sciences of the United States of America* 2000, **97**(8):4345-4350.

40. Ohba N, Kiryu-Seo S, Maeda M, Muraoka M, Ishii M, Kiyama H: **Transgenic mouse overexpressing the Akt reduced the volume of infarct area after middle cerebral artery occlusion**. *Neurosci Lett* 2004, **359**(3):159-162.

41. Salehi A, Verhaagen J, Dijkhuizen PA, Swaab DF: **Co-localization of high-affinity neurotrophin receptors in nucleus basalis of Meynert neurons and their differential reduction in Alzheimer's disease**. *Neuroscience* 1996, **75**(2):373-387.

42. Holtzman DM, Kilbridge J, Li Y, Cunningham ET, Jr., Lenn NJ, Clary DO, Reichardt LF, Mobley WC: **TrkA expression in the CNS: evidence for the existence of several novel NGF-responsive CNS neurons**. *J Neurosci* 1995, **15**(2):1567-1576.

43. Pardridge WM: **Molecular biology of the blood-brain barrier**. *Mol Biotechnol* 2005, **30**(1):57-70.

44. Aktas O, Ullrich O, Infante-Duarte C, Nitsch R, Zipp F: **Neuronal damage in brain inflammation**. *Arch Neurol* 2007, **64**(2):185-189.

45. Akiyama H, Barger S, Barnum S, Bradt B, Bauer J, Cole GM, Cooper NR, Eikelenboom P, Emmerling M, Fiebich BL *et al*: **Inflammation and Alzheimer's disease**. *Neurobiol Aging* 2000, **21**(3):383-421.

46. Bendayan R, Ronaldson PT, Gingras D, Bendayan M: **In situ localization of P-glycoprotein (ABCB1) in human and rat brain**. *J Histochem Cytochem* 2006, **54**(10):1159-1167.

47. Jodoin J, Demeule M, Fenart L, Cecchelli R, Farmer S, Linton KJ, Higgins CF, Beliveau R: **P-glycoprotein in blood-brain barrier endothelial cells: interaction and oligomerization with caveolins**. *J Neurochem* 2003, **87**(4):1010-1023.

48. Maynard TM, Haskell GT, Peters AZ, Sikich L, Lieberman JA, LaMantia AS: **A comprehensive analysis of 22q11 gene expression in the developing and adult brain**. *Proceedings of the National Academy of Sciences of the United States of America* 2003, **100**(24):14433-14438.

49. Chen Z, Zandonatti M, Jakubowski D, Fox HS: **Brain capillary endothelial cells express MBEC1, a protein that is related to the Clostridium perfringens enterotoxin receptors**. *Lab Invest* 1998, **78**(3):353-363.

50. Morita K, Sasaki H, Furuse M, Tsukita S: **Endothelial claudin: claudin-5/TMVCF constitutes tight junction strands in endothelial cells**. *The Journal of cell biology* 1999, **147**(1):185-194.

51. Frelin C, Ladoux A, Marsault R, Vigne P: **Function of vasoactive factors in the cerebral microcirculation**. *J Cardiovasc Pharmacol* 1992, **20 Suppl 12**:S94-96.

52. Matsuo Y, Mihara S, Ninomiya M, Fujimoto M: **Protective effect of endothelin type A receptor antagonist on brain edema and injury after transient middle cerebral artery occlusion in rats**. *Stroke* 2001, **32**(9):2143-2148.

53. Schlachetzki F, Zhu C, Pardridge WM: **Expression of the neonatal Fc receptor (FcRn) at the blood-brain barrier**. *J Neurochem* 2002, **81**(1):203-206.

54. Deane R, Sagare A, Hamm K, Parisi M, LaRue B, Guo H, Wu Z, Holtzman DM, Zlokovic BV: **IgG-assisted age-dependent clearance of Alzheimer's amyloid beta peptide by the blood-brain barrier neonatal Fc receptor**. *J Neurosci* 2005, **25**(50):11495-11503.

55. Pongrac JL, Middleton FA, Peng L, Lewis DA, Levitt P, Mirnics K: **Heat shock protein 12A shows reduced expression in the prefrontal cortex of subjects with schizophrenia**. *Biol Psychiatry* 2004, **56**(12):943-950.

56. Steagall RJ, Rusinol AE, Truong QA, Han Z: **HSPA12B is predominantly expressed in endothelial cells and required for angiogenesis**. *Arteriosclerosis, thrombosis, and vascular biology* 2006, **26**(9):2012-2018.

57. Sugiyama T, Kumagai H, Morikawa Y, Wada Y, Sugiyama A, Yasuda K, Yokoi N, Tamura S, Kojima T, Nosaka T *et al*: **A novel low-density lipoprotein receptor-related protein mediating cellular uptake of apolipoprotein E-enriched beta-VLDL in vitro**. *Biochemistry* 2000, **39**(51):15817-15825.

58. Kim DH, Iijima H, Goto K, Sakai J, Ishii H, Kim HJ, Suzuki H, Kondo H, Saeki S, Yamamoto T: **Human apolipoprotein E receptor 2. A novel lipoprotein receptor of the low density lipoprotein receptor family predominantly expressed in brain**. *The Journal of biological chemistry* 1996, **271**(14):8373-8380.

59. Clatworthy AE, Stockinger W, Christie RH, Schneider WJ, Nimpf J, Hyman BT, Rebeck GW: **Expression and alternate splicing of apolipoprotein E receptor 2 in brain**. *Neuroscience* 1999, **90**(3):903-911.

60. Korschineck I, Ziegler S, Breuss J, Lang I, Lorenz M, Kaun C, Ambros PF, Binder BR: **Identification of a novel exon in apolipoprotein E receptor 2 leading to alternatively spliced mRNAs found in cells of the vascular wall but not in neuronal tissue**. *The Journal of biological chemistry* 2001, **276**(16):13192-13197.

61. Mackic JB, Stins M, McComb JG, Calero M, Ghiso J, Kim KS, Yan SD, Stern D, Schmidt AM, Frangione B *et al*: **Human blood-brain barrier receptors for Alzheimer's amyloid-beta 1- 40. Asymmetrical binding, endocytosis, and transcytosis at the apical side of brain microvascular endothelial cell monolayer**. *J Clin Invest* 1998, **102**(4):734-743.

62. Deane R, Du Yan S, Submamaryan RK, LaRue B, Jovanovic S, Hogg E, Welch D, Manness L, Lin C, Yu J *et al*: **RAGE mediates amyloid-beta peptide transport across the blood-brain barrier and accumulation in brain**. *Nat Med* 2003, **9**(7):907-913.

63. Donahue JE, Flaherty SL, Johanson CE, Duncan JA, 3rd, Silverberg GD, Miller MC, Tavares R, Yang W, Wu Q, Sabo E *et al*: **RAGE, LRP-1, and amyloid-beta protein in Alzheimer's disease**. *Acta Neuropathol (Berl)* 2006, **112**(4):405-415.

64. Li JY, Boado RJ, Pardridge WM: **Cloned blood-brain barrier adenosine transporter is identical to the rat concentrative Na+ nucleoside cotransporter CNT2**. *J Cereb Blood Flow Metab* 2001, **21**(8):929-936.

65. Guillen-Gomez E, Calbet M, Casado J, de Lecea L, Soriano E, Pastor-Anglada M, Burgaya F: **Distribution of CNT2 and ENT1 transcripts in rat brain: selective decrease of CNT2 mRNA in the cerebral cortex of sleep-deprived rats**. *J Neurochem* 2004, **90**(4):883-893.

66. Cornford EM, Hyman S, Swartz BE: **The human brain GLUT1 glucose transporter: ultrastructural localization to the blood-brain barrier endothelia**. *J Cereb Blood Flow Metab* 1994, **14**(1):106-112.

67. Morgello S, Uson RR, Schwartz EJ, Haber RS: **The human blood-brain barrier glucose transporter (GLUT1) is a glucose transporter of gray matter astrocytes**. *Glia* 1995, **14**(1):43-54.

68. Evans JE, Frostholm A, Rotter A: **Embryonic and postnatal expression of four gamma-aminobutyric acid transporter mRNAs in the mouse brain and leptomeninges**. *The Journal of comparative neurology* 1996, **376**(3):431-446.

69. Takanaga H, Ohtsuki S, Hosoya K, Terasaki T: **GAT2/BGT-1 as a system responsible for the transport of gamma-aminobutyric acid at the mouse blood-brain barrier**. *J Cereb Blood Flow Metab* 2001, **21**(10):1232-1239.

70. Matsuo H, Tsukada S, Nakata T, Chairoungdua A, Kim DK, Cha SH, Inatomi J, Yorifuji H, Fukuda J, Endou H *et al*: **Expression of a system L neutral amino acid transporter at the blood-brain barrier**. *Neuroreport* 2000, **11**(16):3507-3511.

71. Kageyama T, Nakamura M, Matsuo A, Yamasaki Y, Takakura Y, Hashida M, Kanai Y, Naito M, Tsuruo T, Minato N *et al*: **The 4F2hc/LAT1 complex transports L-DOPA across the blood-brain barrier**. *Brain Res* 2000, **879**(1-2):115-121.

72. Pizzagalli F, Hagenbuch B, Stieger B, Klenk U, Folkers G, Meier PJ: **Identification of a novel human organic anion transporting polypeptide as a high affinity thyroxine transporter**. *Molecular endocrinology (Baltimore, Md* 2002, **16**(10):2283-2296.

73. Sugiyama D, Kusuhara H, Taniguchi H, Ishikawa S, Nozaki Y, Aburatani H, Sugiyama Y: **Functional characterization of rat brain-specific organic anion transporter (Oatp14) at the blood-brain barrier: high affinity transporter for thyroxine**. *The Journal of biological chemistry* 2003, **278**(44):43489-43495.

74. Kandel ER, Schwartz JH, Thomas M. Jessel TM: *Principles of Neural Science*. Fourth edition. New York: McGraw Hill; 2000.

75. Liu QR, Lopez-Corcuera B, Mandiyan S, Nelson H, Nelson N: **Cloning and expression of a spinal cord- and brain-specific glycine transporter with novel structural features**. *The Journal of biological chemistry* 1993, **268**(30):22802-22808.

76. Laube B, Maksay G, Schemm R, Betz H: **Modulation of glycine receptor function: a novel approach for therapeutic intervention at inhibitory synapses?** *Trends Pharmacol Sci* 2002, **23**(11):519-527.

77. Naciff JM, Kaetzel MA, Behbehani MM, Dedman JR: **Differential expression of annexins I-VI in the rat dorsal root ganglia and spinal cord**. *The Journal of comparative neurology* 1996, **368**(3):356-370.

78. Greenberg DS: **National Institutes of Health moves ahead with "PubMed Central"**. *Lancet* 1999, **354**(9183):1009.

79. Lee MY, Choi JS, Lim SW, Cha JH, Chun MH, Chung JW: **Expression of osteopontin mRNA in developing rat brainstem and cerebellum**. *Cell Tissue Res* 2001, **306**(2):179-185.

80. Shughrue PJ, Lane MV, Merchenthaler I: **Comparative distribution of estrogen receptor-alpha and -beta mRNA in the rat central nervous system**. *The Journal of comparative neurology* 1997, **388**(4):507-525.

81. Nomura M, Akama KT, Alves SE, Korach KS, Gustafsson JA, Pfaff DW, Ogawa S: **Differential distribution of estrogen receptor (ER)-alpha and ER-beta in the midbrain raphe nuclei and periaqueductal gray in male mouse: Predominant role of ER-beta in midbrain serotonergic systems**. *Neuroscience* 2005, **130**(2):445-456.

82. Trieu M, Ma A, Eng SR, Fedtsova N, Turner EE: **Direct autoregulation and gene dosage compensation by POU-domain transcription factor Brn3a**. *Development* 2003, **130**(1):111-121.

83. Quina LA, Pak W, Lanier J, Banwait P, Gratwick K, Liu Y, Velasquez T, O'Leary DD, Goulding M, Turner EE: **Brn3a-expressing retinal ganglion cells project specifically to thalamocortical and collicular visual pathways**. *J Neurosci* 2005, **25**(50):11595-11604.

84. Coetzee T, Fujita N, Dupree J, Shi R, Blight A, Suzuki K, Suzuki K, Popko B: **Myelination in the absence of galactocerebroside and sulfatide: normal structure with abnormal function and regional instability**. *Cell* 1996, **86**(2):209-219.

85. Zheng T, Santi MR, Bovolin P, Marlier LN, Grayson DR: **Developmental expression of the alpha 6 GABAA receptor subunit mRNA occurs only after cerebellar granule cell migration**. *Brain Res Dev Brain Res* 1993, **75**(1):91-103.

86. Funfschilling U, Reichardt LF: **Cre-mediated recombination in rhombic lip derivatives**. *Genesis* 2002, **33**(4):160-169.

87. Nusser Z, Ahmad Z, Tretter V, Fuchs K, Wisden W, Sieghart W, Somogyi P: **Alterations in the expression of GABAA receptor subunits in cerebellar granule cells after the disruption of the alpha6 subunit gene**. *The European journal of neuroscience* 1999, **11**(5):1685-1697.

88. Miura E, Iijima T, Yuzaki M, Watanabe M: **Distinct expression of Cbln family mRNAs in developing and adult mouse brains**. *The European journal of neuroscience* 2006, **24**(3):750-760.

89. Pang Z, Zuo J, Morgan JI: **Cbln3, a novel member of the precerebellin family that binds specifically to Cbln1**. *J Neurosci* 2000, **20**(17):6333-6339.

90. Zhang XM, Ng AH, Tanner JA, Wu WT, Copeland NG, Jenkins NA, Huang JD: **Highly restricted expression of Cre recombinase in cerebellar Purkinje cells**. *Genesis* 2004, **40**(1):45-51.

91. Anderson GW, Larson RJ, Oas DR, Sandhofer CR, Schwartz HL, Mariash CN, Oppenheimer JH: **Chicken ovalbumin upstream promoter-transcription factor (COUP-TF) modulates expression of the Purkinje cell protein-2 gene. A potential role for COUP-TF in repressing premature thyroid hormone action in the developing brain**. *The Journal of biological chemistry* 1998, **273**(26):16391-16399.

92. Vandaele S, Nordquist DT, Feddersen RM, Tretjakoff I, Peterson AC, Orr HT: **Purkinje cell protein-2 regulatory regions and transgene expression in cerebellar compartments**. *Genes & development* 1991, **5**(7):1136-1148.

93. Hayase Y, Higashiyama S, Sasahara M, Amano S, Nakagawa T, Taniguchi N, Hazama F: **Expression of heparin-binding epidermal growth factor-like growth factor in rat brain**. *Brain Res* 1998, **784**(1-2):163-178.

94. Lin X, Antalffy B, Kang D, Orr HT, Zoghbi HY: **Polyglutamine expansion down-regulates specific neuronal genes before pathologic changes in SCA1**. *Nat Neurosci* 2000, **3**(2):157-163.

95. Bergo MO, Leung GK, Ambroziak P, Otto JC, Casey PJ, Gomes AQ, Seabra MC, Young SG: **Isoprenylcysteine carboxyl methyltransferase deficiency in mice**. *The Journal of biological chemistry* 2001, **276**(8):5841-5845.

96. Wu KD, Lee WS, Wey J, Bungard D, Lytton J: **Localization and quantification of endoplasmic reticulum Ca(2+)-ATPase isoform transcripts**. *Am J Physiol* 1995, **269**(3 Pt 1):C775-784.

97. Baba-Aissa F, Raeymaekers L, Wuytack F, Callewaert G, Dode L, Missiaen L, Casteels R: **Purkinje neurons express the SERCA3 isoform of the organellar type Ca(2+)-transport ATPase**. *Brain research* 1996, **41**(1-2):169-174.

98. Volpe P, Gorza L, Brini M, Sacchetto R, Ausoni S, Clegg DO: **Expression of the calsequestrin gene in chicken cerebellum Purkinje neurons**. *Biochem J* 1993, **294 ( Pt 2)**:487-490.

99. Frank KF, Mesnard-Rouiller L, Chu G, Young KB, Zhao W, Haghighi K, Sato Y, Kranias EG: **Structure and expression of the mouse cardiac calsequestrin gene**. *Basic Res Cardiol* 2001, **96**(6):636-644.

100. Zhao R, Lawler AM, Lee SJ: **Characterization of GDF-10 expression patterns and null mice**. *Dev Biol* 1999, **212**(1):68-79.

101. Soderstrom S, Ebendal T: **Localized expression of BMP and GDF mRNA in the rodent brain**. *J Neurosci Res* 1999, **56**(5):482-492.

102. Takao M, Hino J, Takeshita N, Konno Y, Nishizawa T, Matsuo H, Kangawa K: **Identification of rat bone morphogenetic protein-3b (BMP-3b), a new member of BMP-3**. *Biochem Biophys Res Commun* 1996, **219**(2):656-662.

103. Araki K, Meguro H, Kushiya E, Takayama C, Inoue Y, Mishina M: **Selective expression of the glutamate receptor channel delta 2 subunit in cerebellar Purkinje cells**. *Biochem Biophys Res Commun* 1993, **197**(3):1267-1276.

104. Doughty ML, De Jager PL, Korsmeyer SJ, Heintz N: **Neurodegeneration in Lurcher mice occurs via multiple cell death pathways**. *J Neurosci* 2000, **20**(10):3687-3694.

105. Sasai Y, Kageyama R, Tagawa Y, Shigemoto R, Nakanishi S: **Two mammalian helix-loop-helix factors structurally related to Drosophila hairy and Enhancer of split**. *Genes & development* 1992, **6**(12B):2620-2634.

106. Hirata H, Ohtsuka T, Bessho Y, Kageyama R: **Generation of structurally and functionally distinct factors from the basic helix-loop-helix gene Hes3 by alternative first exons**. *The Journal of biological chemistry* 2000, **275**(25):19083-19089.

107. Furuyama T, Inagaki S, Iwahashi Y, Wanaka A, Tohyama M: **Localization of mRNAs for Rlim-1, the rat Xlim-1 homolog, in the developing rat brain**. *Brain research* 1996, **36**(1):152-156.

108. Besco J, Popesco MC, Davuluri RV, Frostholm A, Rotter A: **Genomic structure and alternative splicing of murine R2B receptor protein tyrosine phosphatases (PTPkappa, mu, rho and PCP-2)**. *BMC genomics* 2004, **5**(1):14.

109. Koop EA, Lopes SM, Feiken E, Bluyssen HA, van der Valk M, Voest EE, Mummery CL, Moolenaar WH, Gebbink MF: **Receptor protein tyrosine phosphatase mu expression as a marker for endothelial cell heterogeneity; analysis of RPTPmu gene expression using LacZ knock-in mice**. *The International journal of developmental biology* 2003, **47**(5):345-354.

110. Kolbinger F, Streiff MB, Katopodis AG: **Cloning of a human UDP-galactose:2-acetamido-2-deoxy-D-glucose 3beta-galactosyltransferase catalyzing the formation of type 1 chains**. *The Journal of biological chemistry* 1998, **273**(1):433-440.

111. Hennet T, Dinter A, Kuhnert P, Mattu TS, Rudd PM, Berger EG: **Genomic cloning and expression of three murine UDP-galactose: beta-N-acetylglucosamine beta1,3-galactosyltransferase genes**. *The Journal of biological chemistry* 1998, **273**(1):58-65.

112. Baird JW, Nibbs RJ, Komai-Koma M, Connolly JA, Ottersbach K, Clark-Lewis I, Liew FY, Graham GJ: **ESkine, a novel beta-chemokine, is differentially spliced to produce secretable and nuclear targeted isoforms**. *The Journal of biological chemistry* 1999, **274**(47):33496-33503.

113. Kondo Y, Nakanishi T, Takigawa M, Ogawa N: **Immunohistochemical localization of connective tissue growth factor in the rat central nervous system**. *Brain Res* 1999, **834**(1-2):146-151.

114. Heuer H, Christ S, Friedrichsen S, Brauer D, Winckler M, Bauer K, Raivich G: **Connective tissue growth factor: a novel marker of layer VII neurons in the rat cerebral cortex**. *Neuroscience* 2003, **119**(1):43-52.

115. Chan CH, Godinho LN, Thomaidou D, Tan SS, Gulisano M, Parnavelas JG: **Emx1 is a marker for pyramidal neurons of the cerebral cortex**. *Cereb Cortex* 2001, **11**(12):1191-1198.

116. Jin XL, Guo H, Mao C, Atkins N, Wang H, Avasthi PP, Tu YT, Li Y: **Emx1-specific expression of foreign genes using "knock-in" approach**. *Biochem Biophys Res Commun* 2000, **270**(3):978-982.

117. Iwasato T, Nomura R, Ando R, Ikeda T, Tanaka M, Itohara S: **Dorsal telencephalon-specific expression of Cre recombinase in PAC transgenic mice**. *Genesis* 2004, **38**(3):130-138.

118. Kudo LC, Karsten SL, Chen J, Levitt P, Geschwind DH: **Genetic Analysis of Anterior-Posterior Expression Gradients in the Developing Mammalian Forebrain**. *Cereb Cortex* 2006.

119. Gunther T, Poli C, Muller JM, Catala-Lehnen P, Schinke T, Yin N, Vomstein S, Amling M, Schule R: **Fhl2 deficiency results in osteopenia due to decreased activity of osteoblasts**. *Embo J* 2005, **24**(17):3049-3056.

120. Jamali S, Bartolomei F, Robaglia-Schlupp A, Massacrier A, Peragut JC, Regis J, Dufour H, Ravid R, Roll P, Pereira S *et al*: **Large-scale expression study of human mesial temporal lobe epilepsy: evidence for dysregulation of the neurotransmission and complement systems in the entorhinal cortex**. *Brain* 2006, **129**(Pt 3):625-641.

121. Yajima S, Lammers CH, Lee SH, Hara Y, Mizuno K, Mouradian MM: **Cloning and characterization of murine glial cell-derived neurotrophic factor inducible transcription factor (MGIF)**. *J Neurosci* 1997, **17**(22):8657-8666.

122. Ait-Ghezala G, Abdullah L, Crescentini R, Crawford F, Town T, Singh S, Richards D, Duara R, Mullan M: **Confirmation of association between D10S583 and Alzheimer's disease in a case--control sample**. *Neurosci Lett* 2002, **325**(2):87-90.

123. Quadro L, Blaner WS, Salchow DJ, Vogel S, Piantedosi R, Gouras P, Freeman S, Cosma MP, Colantuoni V, Gottesman ME: **Impaired retinal function and vitamin A availability in mice lacking retinol-binding protein**. *Embo J* 1999, **18**(17):4633-4644.

124. Pignot V, Hein AE, Barske C, Wiessner C, Walmsley AR, Kaupmann K, Mayeur H, Sommer B, Mir AK, Frentzel S: **Characterization of two novel proteins, NgRH1 and NgRH2, structurally and biochemically homologous to the Nogo-66 receptor**. *J Neurochem* 2003, **85**(3):717-728.

125. Lauren J, Airaksinen MS, Saarma M, Timmusk T: **Two novel mammalian Nogo receptor homologs differentially expressed in the central and peripheral nervous systems**. *Mol Cell Neurosci* 2003, **24**(3):581-594.

126. Botta A, Sangiuolo F, Calza L, Giardino L, Potenza S, Novelli G, Dallapiccola B: **Expression analysis and protein localization of the human HPC-1/syntaxin 1A, a gene deleted in Williams syndrome**. *Genomics* 1999, **62**(3):525-528.

127. Inoue A, Akagawa K: **Neuron specific expression of a membrane protein, HPC-1: tissue distribution, and cellular and subcellular localization of immunoreactivity and mRNA**. *Brain research* 1993, **19**(1-2):121-128.

128. Itoh TJ, Fujiwara T, Shibuya T, Akagawa K, Hotani H: **Inhibition of microtubule assembly by HPC-1/syntaxin 1A, an exocytosis relating protein**. *Cell Struct Funct* 1999, **24**(5):359-364.

129. Molnar M, Potkin SG, Bunney WE, Jones EG: **MRNA expression patterns and distribution of white matter neurons in dorsolateral prefrontal cortex of depressed patients differ from those in schizophrenia patients**. *Biol Psychiatry* 2003, **53**(1):39-47.

130. Bulfone A, Smiga SM, Shimamura K, Peterson A, Puelles L, Rubenstein JL: **T-brain-1: a homolog of Brachyury whose expression defines molecularly distinct domains within the cerebral cortex**. *Neuron* 1995, **15**(1):63-78.

131. Kolk SM, Whitman MC, Yun ME, Shete P, Donoghue MJ: **A unique subpopulation of Tbr1-expressing deep layer neurons in the developing cerebral cortex**. *Mol Cell Neurosci* 2006, **32**(1-2):200-214.

132. Hill JM, Ades AM, McCune SK, Sahir N, Moody EM, Abebe DT, Crnic LS, Brenneman DE: **Vasoactive intestinal peptide in the brain of a mouse model for Down syndrome**. *Exp Neurol* 2003, **183**(1):56-65.

133. Cirelli C, Tononi G: **Gene expression in the brain across the sleep-waking cycle**. *Brain Res* 2000, **885**(2):303-321.

134. Naeve GS, Vana AM, Eggold JR, Verge G, Ling N, Foster AC: **Expression of rat insulin-like growth factor binding protein-6 in the brain, spinal cord, and sensory ganglia**. *Brain research* 2000, **75**(2):185-197.

135. Bienvenu G, Seurin D, Le Bouc Y, Even P, Babajko S, Magnan C: **Dysregulation of energy homeostasis in mice overexpressing insulin-like growth factor-binding protein 6 in the brain**. *Diabetologia* 2005, **48**(6):1189-1197.

136. Matsuoka H, Iwata N, Ito M, Shimoyama M, Nagata A, Chihara K, Takai S, Matsui T: **Expression of a kinase-defective Eph-like receptor in the normal human brain**. *Biochem Biophys Res Commun* 1997, **235**(3):487-492.

137. Gurniak CB, Berg LJ: **A new member of the Eph family of receptors that lacks protein tyrosine kinase activity**. *Oncogene* 1996, **13**(4):777-786.

138. Schwartz F, Ota T: **The 239AB gene on chromosome 22: a novel member of an ancient gene family**. *Gene* 1997, **194**(1):57-62.

139. Dan C, Nath N, Liberto M, Minden A: **PAK5, a new brain-specific kinase, promotes neurite outgrowth in N1E-115 cells**. *Molecular and cellular biology* 2002, **22**(2):567-577.

140. Pandey A, Dan I, Kristiansen TZ, Watanabe NM, Voldby J, Kajikawa E, Khosravi-Far R, Blagoev B, Mann M: **Cloning and characterization of PAK5, a novel member of mammalian p21-activated kinase-II subfamily that is predominantly expressed in brain**. *Oncogene* 2002, **21**(24):3939-3948.

141. Szemes M, Gyorgy A, Paweletz C, Dobi A, Agoston DV: **Isolation and characterization of SATB2, a novel AT-rich DNA binding protein expressed in development- and cell-specific manner in the rat brain**. *Neurochem Res* 2006, **31**(2):237-246.

142. Reim K, Wegmeyer H, Brandstatter JH, Xue M, Rosenmund C, Dresbach T, Hofmann K, Brose N: **Structurally and functionally unique complexins at retinal ribbon synapses**. *The Journal of cell biology* 2005, **169**(4):669-680.

143. Zhuo M: **Molecular mechanisms of pain in the anterior cingulate cortex**. *J Neurosci Res* 2006, **84**(5):927-933.

144. Tsai JC, Liu L, Cooley BC, DiChiara MR, Topper JN, Aird WC: **The Egr-1 promoter contains information for constitutive and inducible expression in transgenic mice**. *Faseb J* 2000, **14**(13):1870-1872.

145. Herms J, Zurmohle U, Schlingensiepen R, Brysch W, Schlingensiepen KH: **Developmental expression of the transcription factor zif268 in rat brain**. *Neurosci Lett* 1994, **165**(1-2):171-174.

146. Thomas KL, Hall J, Everitt BJ: **Cellular imaging with zif268 expression in the rat nucleus accumbens and frontal cortex further dissociates the neural pathways activated following the retrieval of contextual and cued fear memory**. *The European journal of neuroscience* 2002, **16**(9):1789-1796.

147. Ko SW, Vadakkan KI, Ao H, Gallitano-Mendel A, Wei F, Milbrandt J, Zhuo M: **Selective contribution of Egr1 (zif/268) to persistent inflammatory pain**. *J Pain* 2005, **6**(1):12-20.

148. Peschanski M, Hirsch E, Dusart I, Doye V, Marty S, Manceau V, Sobel A: **Stathmin: cellular localization of a major phosphoprotein in the adult rat and human CNS**. *The Journal of comparative neurology* 1993, **337**(4):655-668.

149. Shumyatsky GP, Malleret G, Shin RM, Takizawa S, Tully K, Tsvetkov E, Zakharenko SS, Joseph J, Vronskaya S, Yin D *et al*: **stathmin, a gene enriched in the amygdala, controls both learned and innate fear**. *Cell* 2005, **123**(4):697-709.

150. Clark D, Dedova I, Cordwell S, Matsumoto I: **A proteome analysis of the anterior cingulate cortex gray matter in schizophrenia**. *Molecular psychiatry* 2006, **11**(5):459-470, 423.

151. Jagerschmidt A, Popovici T, O'Donohue M, Roques BP: **Identification and characterization of various cholecystokinin B receptor mRNA forms in rat brain tissue and partial determination of the cholecystokinin B receptor gene structure**. *J Neurochem* 1994, **63**(4):1199-1206.

152. Koks S, Volke V, Veraksits A, Runkorg K, Sillat T, Abramov U, Bourin M, Huotari M, Mannisto PT, Matsui T *et al*: **Cholecystokinin2 receptor-deficient mice display altered function of brain dopaminergic system**. *Psychopharmacology (Berl)* 2001, **158**(2):198-204.

153. Raud S, Innos J, Abramov U, Reimets A, Koks S, Soosaar A, Matsui T, Vasar E: **Targeted invalidation of CCK2 receptor gene induces anxiolytic-like action in light-dark exploration, but not in fear conditioning test**. *Psychopharmacology (Berl)* 2005, **181**(2):347-357.

154. Zachrisson O, de Belleroche J, Wendt KR, Hirsch S, Lindefors N: **Cholecystokinin CCK(B) receptor mRNA isoforms: expression in schizophrenic brains**. *Neuroreport* 1999, **10**(16):3265-3268.

155. Kurrikoff K, Koks S, Matsui T, Bourin M, Arend A, Aunapuu M, Vasar E: **Deletion of the CCK2 receptor gene reduces mechanical sensitivity and abolishes the development of hyperalgesia in mononeuropathic mice**. *The European journal of neuroscience* 2004, **20**(6):1577-1586.

156. Wei F, Qiu CS, Kim SJ, Muglia L, Maas JW, Pineda VV, Xu HM, Chen ZF, Storm DR, Muglia LJ *et al*: **Genetic elimination of behavioral sensitization in mice lacking calmodulin-stimulated adenylyl cyclases**. *Neuron* 2002, **36**(4):713-726.

157. Abdel-Majid RM, Leong WL, Schalkwyk LC, Smallman DS, Wong ST, Storm DR, Fine A, Dobson MJ, Guernsey DL, Neumann PE: **Loss of adenylyl cyclase I activity disrupts patterning of mouse somatosensory cortex**. *Nature genetics* 1998, **19**(3):289-291.

158. Yamamoto M, Ozawa H, Saito T, Hatta S, Riederer P, Takahata N: **Ca2+/CaM-sensitive adenylyl cyclase activity is decreased in the Alzheimer's brain: possible relation to type I adenylyl cyclase**. *J Neural Transm* 1997, **104**(6-7):721-732.

159. Nagai M, Kishi K, Kato S: **Insular cortex and neuropsychiatric disorders: A review of recent literature**. *Eur Psychiatry* 2007.

160. Arimatsu Y, Kojima M, Ishida M: **Area- and lamina-specific organization of a neuronal subpopulation defined by expression of latexin in the rat cerebral cortex**. *Neuroscience* 1999, **88**(1):93-105.

161. Hatanaka Y, Uratani Y, Takiguchi-Hayashi K, Omori A, Sato K, Miyamoto M, Arimatsu Y: **Intracortical regionality represented by specific transcription for a novel protein, latexin**. *The European journal of neuroscience* 1994, **6**(6):973-982.

162. Jin M, Ishida M, Katoh-Fukui Y, Tsuchiya R, Higashinakagawa T, Ikegami S, Arimatsu Y: **Reduced pain sensitivity in mice lacking latexin, an inhibitor of metallocarboxypeptidases**. *Brain Res* 2006, **1075**(1):117-121.

163. Yin Y, Miner JH, Sanes JR: **Laminets: laminin- and netrin-related genes expressed in distinct neuronal subsets**. *Mol Cell Neurosci* 2002, **19**(3):344-358.

164. Meerabux JM, Ohba H, Fukasawa M, Suto Y, Aoki-Suzuki M, Nakashiba T, Nishimura S, Itohara S, Yoshikawa T: **Human netrin-G1 isoforms show evidence of differential expression**. *Genomics* 2005, **86**(1):112-116.

165. Aoki-Suzuki M, Yamada K, Meerabux J, Iwayama-Shigeno Y, Ohba H, Iwamoto K, Takao H, Toyota T, Suto Y, Nakatani N *et al*: **A family-based association study and gene expression analyses of netrin-G1 and -G2 genes in schizophrenia**. *Biol Psychiatry* 2005, **57**(4):382-393.

166. Backman C, Perlmann T, Wallen A, Hoffer BJ, Morales M: **A selective group of dopaminergic neurons express Nurr1 in the adult mouse brain**. *Brain Res* 1999, **851**(1-2):125-132.

167. Arimatsu Y, Ishida M, Kaneko T, Ichinose S, Omori A: **Organization and development of corticocortical associative neurons expressing the orphan nuclear receptor Nurr1**. *The Journal of comparative neurology* 2003, **466**(2):180-196.

168. Werme M, Hermanson E, Carmine A, Buervenich S, Zetterstrom RH, Thoren P, Ogren SO, Olson L, Perlmann T, Brene S: **Decreased ethanol preference and wheel running in Nurr1-deficient mice**. *The European journal of neuroscience* 2003, **17**(11):2418-2424.

169. Xing G, Zhang L, Russell S, Post R: **Reduction of dopamine-related transcription factors Nurr1 and NGFI-B in the prefrontal cortex in schizophrenia and bipolar disorders**. *Schizophr Res* 2006, **84**(1):36-56.

170. Chen JG, Rasin MR, Kwan KY, Sestan N: **Zfp312 is required for subcortical axonal projections and dendritic morphology of deep-layer pyramidal neurons of the cerebral cortex**. *Proceedings of the National Academy of Sciences of the United States of America* 2005, **102**(49):17792-17797.

171. Chen B, Schaevitz LR, McConnell SK: **Fezl regulates the differentiation and axon targeting of layer 5 subcortical projection neurons in cerebral cortex**. *Proceedings of the National Academy of Sciences of the United States of America* 2005, **102**(47):17184-17189.

172. Hirata T, Nakazawa M, Yoshihara S, Miyachi H, Kitamura K, Yoshihara Y, Hibi M: **Zinc-finger gene Fez in the olfactory sensory neurons regulates development of the olfactory bulb non-cell-autonomously**. *Development* 2006, **133**(8):1433-1443.

173. Kamata T, Katsube K, Michikawa M, Yamada M, Takada S, Mizusawa H: **R-spondin, a novel gene with thrombospondin type 1 domain, was expressed in the dorsal neural tube and affected in Wnts mutants**. *Biochim Biophys Acta* 2004, **1676**(1):51-62.

174. Couceyro PR, Koylu EO, Kuhar MJ: **Further studies on the anatomical distribution of CART by in situ hybridization**. *J Chem Neuroanat* 1997, **12**(4):229-241.

175. Koylu EO, Couceyro PR, Lambert PD, Kuhar MJ: **Cocaine- and amphetamine-regulated transcript peptide immunohistochemical localization in the rat brain**. *The Journal of comparative neurology* 1998, **391**(1):115-132.

176. Hurd YL, Fagergren P: **Human cocaine- and amphetamine-regulated transcript (CART) mRNA is highly expressed in limbic- and sensory-related brain regions**. *The Journal of comparative neurology* 2000, **425**(4):583-598.

177. Damaj MI, Martin BR, Kuhar MJ: **Antinociceptive effects of supraspinal rat cart (55-102) peptide in mice**. *Brain Res* 2003, **983**(1-2):233-236.

178. Carson JP, Ju T, Lu HC, Thaller C, Xu M, Pallas SL, Crair MC, Warren J, Chiu W, Eichele G: **A digital atlas to characterize the mouse brain transcriptome**. *PLoS Comput Biol* 2005, **1**(4):e41.

179. Montgomery DL: **Astrocytes: form, functions, and roles in disease**. *Vet Pathol* 1994, **31**(2):145-167.

180. Maragakis NJ, Rothstein JD: **Mechanisms of Disease: astrocytes in neurodegenerative disease**. *Nat Clin Pract Neurol* 2006, **2**(12):679-689.

181. Kohama SG, Goss JR, Finch CE, McNeill TH: **Increases of glial fibrillary acidic protein in the aging female mouse brain**. *Neurobiol Aging* 1995, **16**(1):59-67.

182. Landry CF, Watson JB, Kashima T, Campagnoni AT: **Cellular influences on RNA sorting in neurons and glia: an in situ hybridization histochemical study**. *Brain research* 1994, **27**(1):1-11.

183. Liu X, Bolteus AJ, Balkin DM, Henschel O, Bordey A: **GFAP-expressing cells in the postnatal subventricular zone display a unique glial phenotype intermediate between radial glia and astrocytes**. *Glia* 2006, **54**(5):394-410.

184. Adami C, Sorci G, Blasi E, Agneletti AL, Bistoni F, Donato R: **S100B expression in and effects on microglia**. *Glia* 2001, **33**(2):131-142.

185. Richter-Landsberg C, Heinrich M: **S-100 immunoreactivity in rat brain glial cultures is associated with both astrocytes and oligodendrocytes**. *J Neurosci Res* 1995, **42**(5):657-665.

186. Nishiyama H, Knopfel T, Endo S, Itohara S: **Glial protein S100B modulates long-term neuronal synaptic plasticity**. *Proceedings of the National Academy of Sciences of the United States of America* 2002, **99**(6):4037-4042.

187. Rothstein JD, Martin L, Levey AI, Dykes-Hoberg M, Jin L, Wu D, Nash N, Kuncl RW: **Localization of neuronal and glial glutamate transporters**. *Neuron* 1994, **13**(3):713-725.

188. Matsugami TR, Tanemura K, Mieda M, Nakatomi R, Yamada K, Kondo T, Ogawa M, Obata K, Watanabe M, Hashikawa T *et al*: **From the Cover: Indispensability of the glutamate transporters GLAST and GLT1 to brain development**. *Proceedings of the National Academy of Sciences of the United States of America* 2006, **103**(32):12161-12166.

189. Chen ZL, Yoshida S, Kato K, Momota Y, Suzuki J, Tanaka T, Ito J, Nishino H, Aimoto S, Kiyama H *et al*: **Expression and activity-dependent changes of a novel limbic-serine protease gene in the hippocampus**. *J Neurosci* 1995, **15**(7 Pt 2):5088-5097.

190. Letwin NE, Kafkafi N, Benjamini Y, Mayo C, Frank BC, Luu T, Lee NH, Elmer GI: **Combined application of behavior genetics and microarray analysis to identify regional expression themes and gene-behavior associations**. *J Neurosci* 2006, **26**(20):5277-5287.

191. Fauser S, Deininger MH, Kremsner PG, Magdolen V, Luther T, Meyermann R, Schluesener HJ: **Lesion associated expression of urokinase-type plasminogen activator receptor (uPAR, CD87) in human cerebral malaria**. *J Neuroimmunol* 2000, **111**(1-2):234-240.

192. Washington RA, Becher B, Balabanov R, Antel J, Dore-Duffy P: **Expression of the activation marker urokinase plasminogen-activator receptor in cultured human central nervous system microglia**. *J Neurosci Res* 1996, **45**(4):392-399.

193. Iwasaki Y, Hosoya T, Takebayashi H, Ogawa Y, Hotta Y, Ikenaka K: **The potential to induce glial differentiation is conserved between Drosophila and mammalian glial cells missing genes**. *Development* 2003, **130**(24):6027-6035.

194. Kanemura Y, Hiraga S, Arita N, Ohnishi T, Izumoto S, Mori K, Matsumura H, Yamasaki M, Fushiki S, Yoshimine T: **Isolation and expression analysis of a novel human homologue of the Drosophila glial cells missing (gcm) gene**. *FEBS letters* 1999, **442**(2-3):151-156.

195. Kim J, Jones BW, Zock C, Chen Z, Wang H, Goodman CS, Anderson DJ: **Isolation and characterization of mammalian homologs of the Drosophila gene glial cells missing**. *Proceedings of the National Academy of Sciences of the United States of America* 1998, **95**(21):12364-12369.

196. Gopalan SM, Wilczynska KM, Konik BS, Bryan L, Kordula T: **Nuclear factor-1-X regulates astrocyte-specific expression of the alpha1-antichymotrypsin and glial fibrillary acidic protein genes**. *The Journal of biological chemistry* 2006, **281**(19):13126-13133.

197. Abraham CR, Selkoe DJ, Potter H: **Immunochemical identification of the serine protease inhibitor alpha 1-antichymotrypsin in the brain amyloid deposits of Alzheimer's disease**. *Cell* 1988, **52**(4):487-501.

198. Dheen ST, Kaur C, Ling EA: **Microglial activation and its implications in the brain diseases**. *Curr Med Chem* 2007, **14**(11):1189-1197.

199. Rock RB, Gekker G, Hu S, Sheng WS, Cheeran M, Lokensgard JR, Peterson PK: **Role of microglia in central nervous system infections**. *Clin Microbiol Rev* 2004, **17**(4):942-964, table of contents.

200. Guillemin GJ, Brew BJ: **Microglia, macrophages, perivascular macrophages, and pericytes: a review of function and identification**. *J Leukoc Biol* 2004, **75**(3):388-397.

201. Holness CL, Simmons DL: **Molecular cloning of CD68, a human macrophage marker related to lysosomal glycoproteins**. *Blood* 1993, **81**(6):1607-1613.
[truncated: 52,207 more chars]
